# Iterative Assembly of Macrocyclic Lactones using Successive Ring Expansion Reactions

**DOI:** 10.1002/chem.201803064

**Published:** 2018-08-19

**Authors:** Thomas C. Stephens, Aggie Lawer, Thomas French, William P. Unsworth

**Affiliations:** ^1^ University of York York YO10 5DD UK

**Keywords:** lactones, macrocycles, macrolides, medium-sized rings, ring expansion

## Abstract

Macrocyclic lactones can be prepared from lactams and hydroxyacid derivatives via an efficient 3‐ or 4‐atom iterative ring expansion protocol. The products can also be expanded using amino acid‐based linear fragments, meaning that macrocycles with precise sequences of hydroxy‐ and amino acids can be assembled in high yields by “growing” them from smaller rings, using a simple procedure in which high dilution is not required. The method should significantly expedite the practical synthesis of diverse nitrogen containing macrolide frameworks.

## Introduction

Nature routinely makes use of exquisitely selective assembly line‐type processes^1^ to construct molecules vital to life, such as DNA and polyketide metabolites,^2^ and artificial synthetic methods based on similar principles have long been known for the synthesis of peptides^3^ and oligonucleotides,^4^ thus transforming synthetic biology and its associated fields. Indeed, the value of assembly line type approaches is increasingly being recognised for the preparation of other compound classes: seminal methods include those for the synthesis of sugars,^5^ polyketide derivatives,^6^ sp^3^‐rich hydrocarbons,^7^ polyenes,^8^ cyclic ethers,^9^ polyaromatics^10^ and various others.^11^

**Figure 1:**
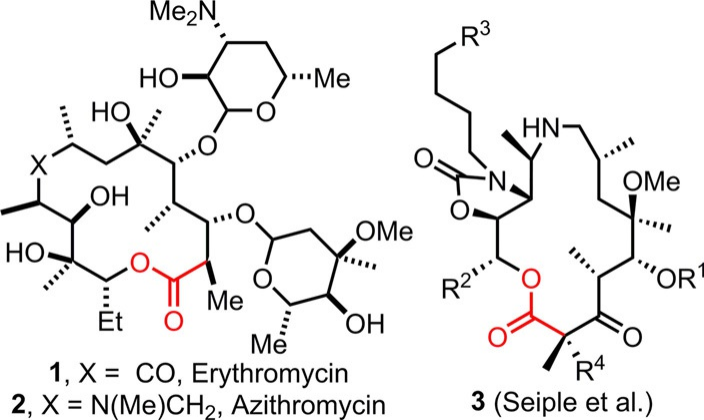
Macrolide antibiotics **1**–**3**.

This manuscript concerns our efforts to develop a practical, iterative method for the assembly of macrocyclic lactones. Medicinal interest in macrocycles has risen markedly in recent years,^12^, ^13^ with macrocyclic lactones (especially macrolide antibiotics)^14^ featuring heavily in medicinally oriented research. Naturally occurring macrolides such as erythromycin **1** 14a have long been used as antibiotics, while analogues prepared via semi‐synthesis (e.g. azithromycin **2**)14b as well as fully synthetic analogues (e.g. **3**)14f,14g have since been developed to address the challenge of rising anti‐microbial resistance (Figure 1).^15^ Macrocyclic lactones (and indeed most macrocycles) are usually difficult to make, largely due to the energetic barriers that must be overcome to promote the end‐to‐end cyclisation of a linear precursor.^16^ Nonetheless, several powerful strategies have emerged over the years to address this,^17^, ^18^ with ring closure via the lactone C−O bond (e.g. the Yamaguchi macrolactonisation reaction) and ring closing metathesis amongst the most popular.^18^ However, such methods usually rely on high dilution conditions to favour macrocyclisation over competing dimerisation or oligomerisation pathways, and this impacts their practicality.^16^ Furthermore, macrocyclisation reactions are typically highly sensitive to structural and conformational changes in the cyclisation precursors. This means that generalised, building block approaches to prepare macrocyclic lactones are rare, although a notable exception is the excellent work of Seiple, Zhang, Myers and co‐workers, in which a modular platform for the efficient synthesis of >300 macrolide antibiotic candidates (*c.f*. **3**, Figure 1) is described.14f

**Figure 2:**
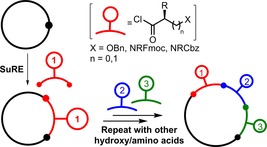
Iterative assembly of macrocyclic lactones using successive ring expansion reactions (SuRE).

In terms of developing a general, practical route to macrocyclic lactones, ring expansion strategies have much potential,^19^, ^20^ as the end‐to‐end cyclisation step that hampers conventional macrocyclisation methods is completely avoided. Thus, in this manuscript, we describe the development of a high yielding, iterative strategy for the synthesis of macrocyclic lactones using Successive Ring Expansion (SuRE) reactions.^21^ The new synthetic protocols reported enable a broad array of functionalised lactone‐ and lactam‐containing macrocycles (10–24‐membered) to be prepared in high yields by the iterative insertion of both hydroxy acid and amino acid‐based linear fragments into lactams (Figure 2).

## Results and Discussion

**Scheme 1:**
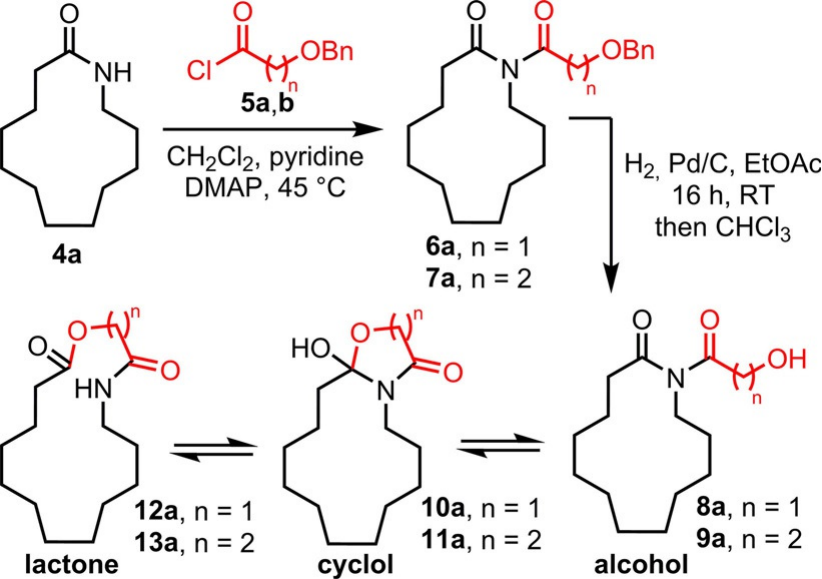
Ring expansion sequence to macrocyclic lactones **12 a** and **13 a**.

Previous research in our group has focused on the development of ring expansion routes to macrocyclic lactams,^21^, ^22^ although we recently uncovered two examples of lactone‐forming reactions that operate via a similar strategy.21c Thus, following *N*‐acylation of lactam **4 a**, the resulting imides (**6**/**7 a**) were shown to undergo hydrogenolysis (to form alcohols **8**/**9 a**) and rearrange via cyclols **10**/**11 a** to furnish ring expanded lactones **12 a** and **13 a** (Scheme 1). Unlike in our previous lactam work, ring expansion did not take place spontaneously following protecting group cleavage (an equilibrating mixture of isomers **8**/**9 a**, **10**/**11 a**, and **12**/**13 a** was formed in each case) but stirring this mixture in chloroform was sufficient to drive the equilibrium towards ring expanded macrocyclic lactones **12 a** and **13 a**, which were isolated in 88 and 47 % yields, respectively.

**Scheme 2:**
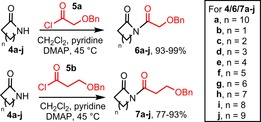
*N*‐acylation of lactams **4 a–j**.

To establish whether these proof‐of‐concept results could be expanded into a general method, we began by evaluating the effect of the ring size of the starting lactam on the reaction outcome. Ring size was predicted to have a major impact on the alcohol/cyclol/lactone equilibrium shown in Scheme 1; in particular, the expansion of normal ring sizes (5–7‐membered) into medium‐sized rings (8–11‐membered) was expected to be challenging, in view of the well‐known difficulties associated of making medium‐sized rings.^23^ To facilitate this, a total of 20 *N*‐acylated derivatives **6 a**–**j** and **7 a**–**j** were prepared, using 4–13‐membered lactams **4 a**–**j** and α‐ and β‐hydroxyacid derivatives **5 a** and **5 b**, which were coupled using a high yielding, lactam *N*‐acylation procedure summarised in Scheme 2.

**Scheme 3:**
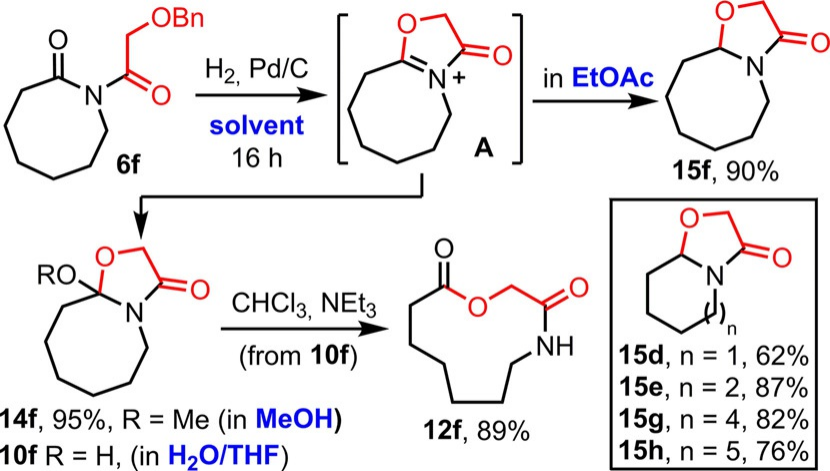
Solvent dependent fates of intermediate **14**.

We then moved on to examine their ring expansion reactions, starting with the α‐hydroxyacid derivatives **6 a**–**j**. Although we had already shown that ring‐expanded lactone **12 a** could be made in high yield from **6 a**, literature precedent suggested that other ring sizes would not be so easy; for example, imides **6 c**–**6 e** have been described previously in separate studies by Shemayakin, Antonov and co‐workers24a and Griot and co‐workers,24b but in their hands were found to produce mixtures of alcohol (**8**) and cyclol (**10**) products following hydrogenolysis, with no evidence for having undergone ring expansion. However, when our hydrogenolysis conditions were applied to novel imide **6 f**, *N*,*O*‐acetal **15 f** was unexpectedly formed in 87 % yield, presumably via reduction of a dehydrated intermediate of the form **A** (Scheme 3). The same process also operates on other ring sizes, with *N*,*O*‐acetals **15 d**–**h** all being formed similarly, from their respective imides **6 d**–**h** (Scheme 3 box).^25^

While this discovery represents an interesting way to prepare cyclic *N*,*O*‐acetals,^26^ it was problematic in the context of generating ring‐expanded lactones. A solution was found by changing the hydrogenolysis solvent; thus, if the hydrogenolysis was carried out in methanol, intermediate **A** was trapped by the solvent to form a methanol adduct (**14 f**) that is stable with respect to over‐reduction and was isolated in 95 % yield. We then considered that by exchanging methanol for water, a water‐trapped adduct (i.e. cyclol **10 f**) would form similarly, and serve as an intermediate towards the desired ring expansion lactone. Pleasingly this idea worked well; thus, the hydrogenolysis was performed in a mixed THF/water solvent system, and following filtration, the reaction mixture (which at this stage was largely comprised of cyclol **10 f**) was stirred in chloroform/NEt_3_ at RT, which promoted clean isomerisation to the desired ring expansion product **12 f** in 89 % yield (Scheme 3).

**Scheme 4:**
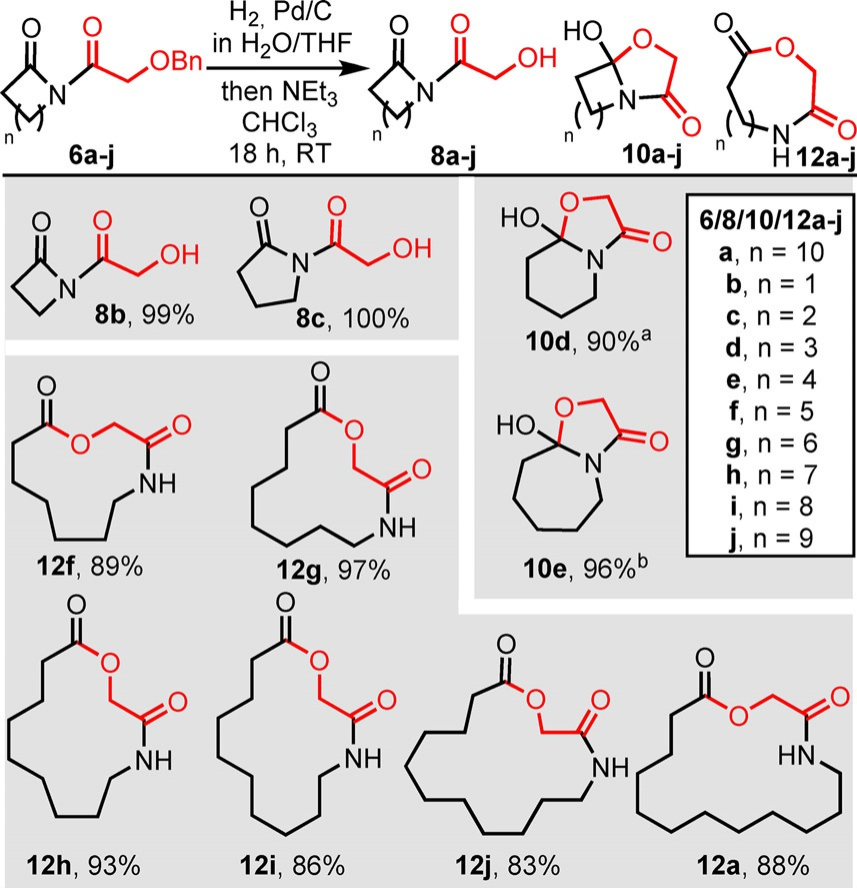
Ring expansion of α‐hydroxyacid derivatives **6 a**–**j**. [a] For simplicity, the major isomeric form of **10 d** is drawn, but in CDCl_3_ solution, this compounds exists as a 3:2 ratio of **10 d**:**8 d**. [b] For simplicity, the major isomeric form of **10 e** is drawn, but in CDCl_3_ solution, this compounds exists as a 69:13:1 ratio of **10 e**:**8 e**:**12 e**.

Having established a viable hydrogenolysis method in which over‐reduction can be avoided, this procedure was applied to all of the 4–13‐membered cyclic imide precursors **6 a**–**j** (Scheme 4). From these experiments, a clear trend emerged linking the size of the cyclic starting material **6** to the reaction outcome. Thus, in the cases of 4‐ and 5‐membered cyclic imides **6 a** and **6 b**, debenzylation proceeded smoothly, but isomerisation did not occur following stirring in chloroform/NEt_3_, with imides **8 b** and **8 c** being isolated in high yields. Conversely, under the same conditions, 6‐ and 7‐membered imides **8 d** and **8 e** only partially rearranged; cyclol isomers **10 d** and **10 e** were formed as the major products in CDCl_3_ solution, although the corresponding imide and ring expanded isomeric forms were also visible in their ^1^H NMR spectra. Finally, all the cyclic imides from 8‐membered **6 f** to 13‐membered **6 a** underwent hydrogenolysis and ring expansion as desired, to deliver ring expanded products **12 f**–**j** and **12 a** in high yields (83–97 %).

**Scheme 5:**
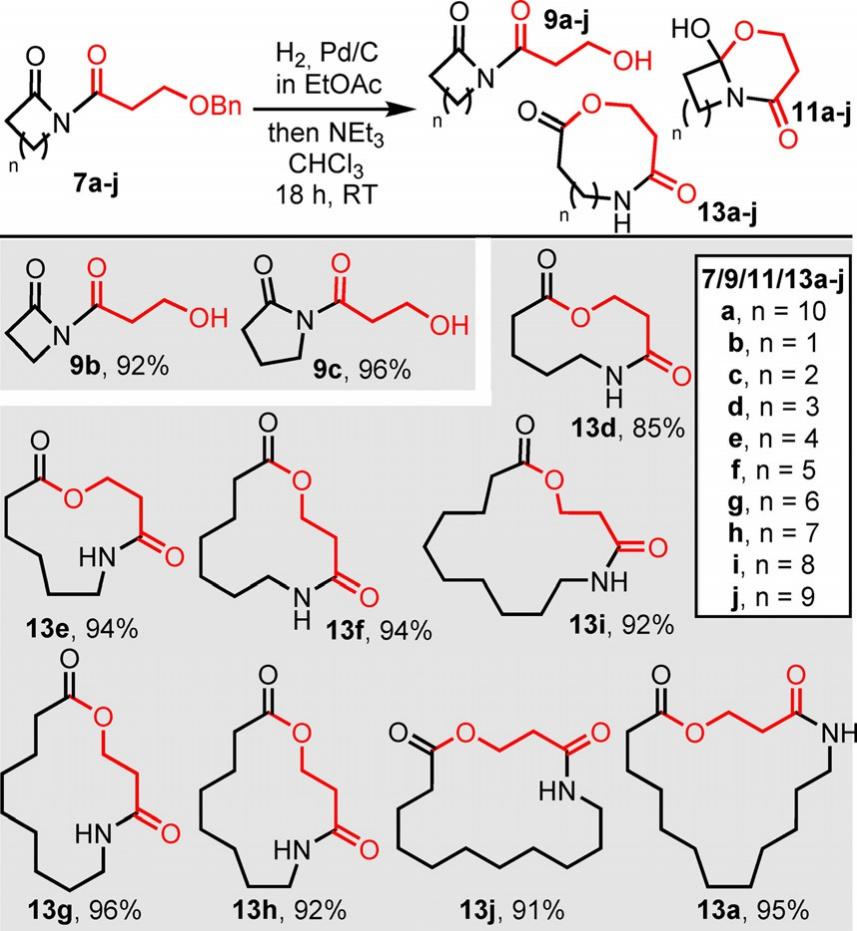
Ring expansion of β‐hydroxyacid derivatives **7 a**–**j**.

We then examined β‐hydroxyacid derivatives **7 a**–**j**. Helpfully, in this series over‐reduction was not observed, hence hydrogenolysis could be performed in ethyl acetate, and was followed by stirring in chloroform/NEt_3_ as before. Again, clear ring size trends emerged; as in the α‐hydroxyacid series, the 4‐ and 5‐membered ring starting materials **7 b** and **7 c** failed to undergo ring expansion following hydrogenolysis, with alcohols **9 b** and **9 c** being isolated instead, but all cyclic imides from 6‐membered **7 d** to 13‐membered **7 a** were successfully converted into the desired ring expansion products **13 d**–**j** and **13 a** in high yields (85–96 %, Scheme 5).

Thus, both the α‐ and β‐hydroxyacid series have a clear point at which the ring expansion reactions “switch on”, that is ≥8‐membered rings expand effectively in the α‐hydroxyacid series, and ≥6‐membered for their β‐hydroxyacid analogues. Of course, there will likely be some substrate specific variation, but these ring size guidelines should be helpful in predicting the viability of ring expansion processes on related systems. We believe that these are thermodynamic outcomes, and that following hydrogenolysis, the three isomeric forms **8**, **10** and **12** (or **9**, **11** and **13**) equilibrate upon stirring in chloroform/NEt_3._ The observed results are consistent with what we know about the difficulties associated with medium ring system,^23^ (which typically suffer from ring strain and/or destabilizing transannular interactions) and are supported by a relatively simple computational study, using Density Functional Theory (DFT),^27^ which drew inspiration from a related study on lactam‐forming ring expansions by Yudin and co‐workers.^22^ Thus, the relative Gibbs free energies of isomeric imide (**8**/**9**), cyclol (**10**/**11**) and ring expanded products (**12**/**13**) were calculated for the four reaction systems which lie on the borderline of undergoing ring expansion or remaining as the imide form (i.e. those leading to the formation of **8 c**, **12 f**, **9 c** and **13 d**) with these results summarised in Table 1. Pleasingly, the calculations agree with the synthetic outcomes; thus, for the α‐hydroxyacid series, the 5‐membered ring imide form **8 c** was calculated to be significantly lower in energy than either the cyclol or ring expanded isomers, whereas the ring expanded form **12 f** was calculated to have the lowest Gibbs free energy for the 8‐membered starting material. A similar trend was also seen for the β‐hydroxyacid series, in which the switch in the reaction outcome between the 5‐ and 6‐membered starting materials observed synthetically was predicted by the DFT calculations.^27^, ^28^ Full details of the computational methods can be found in the Supplementary Information (SI). Also included in the SI are calculations for the two reaction systems which produced mixtures of products (**8 d**/**10 d**/**12 d** and **8 e**/**10 e**/**12 e**). In these cases, the three isomeric forms **8**/**10**/**12** were found to be much closer in Gibbs free energy in comparison to those shown in Table 1, with no isomer being >3 kcal mol^−1^ lower in Gibbs free energy than each of the other two, hence it is not surprising that a mixture of products was obtained in the synthetic reactions; indeed, these results further corroborate the notion that the reactions are under thermodynamic control.

**Table 1:** DFT [B3LYP/6‐31G*] calculated relative Gibbs free energy values (in vacuum) for isomeric imides (**8**/**9**), cyclols (1**0**/**11**) and lactones (**12**/**13**).^27^

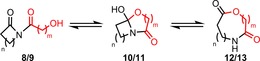					
n	m	Ring sizes	**8/9**	**10/11**	**12/13**
			Δ*G*°_rel_ (kcal/mol)		
2	1	5→8	0.0 (**8 c**)	13.4 (**10 c**)	10.3 (**12 c**)
5	1	8→11	6.3 (**8 f**)	8.9 (**10 f**)	0.0 (**12 f**)
2	2	5→9	0.0 (**9 c**)	11.7 (**11 c**)	4.1 (**13 c**)
3	2	6→10	2.4 (**9 d**)	8.1 (**11 d**)	0.0 (**13 d**)

**Scheme 6:**
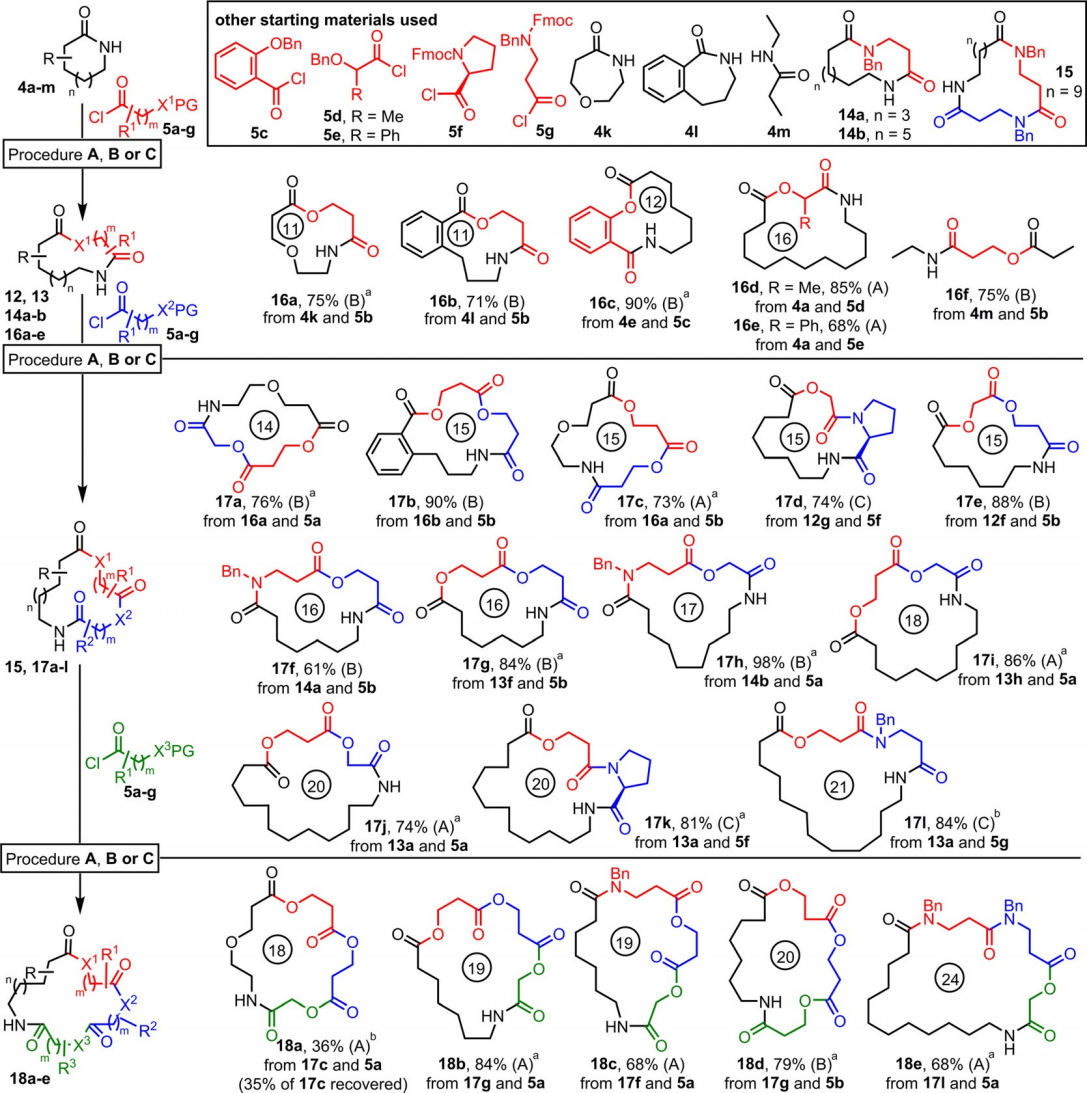
Successive ring expansion reactions. For all Procedures A–C: i) Lactam (1 equiv), Pyridine (6 equiv), DMAP (0.1 equiv), ROCl **5 a**‐**g** (1.5 equiv), CH_2_Cl_2_ (0.1 m), 18 h, 45 °C, then: Procedure A: ii) H_2_, Pd/C in H_2_O/THF; iii) NEt_3_, CHCl_3_ 18 h, RT (for XPG=OBn, *m*=1) Procedure B: ii) H_2_, Pd/C in EtOAc; iii) NEt_3_, CHCl_3_ 18 h, RT (for XPG=OBn, *m*=2) Procedure C: ii) DBU (10 equiv), CH_2_Cl_2_ 18 h, RT (for XPG=NRFmoc, *m*=1 or 2). [a] An additional 1.5 equiv of ROCl **5** was used in the *N*‐acylation (step i) to help ensure complete conversion; [b] An additional 4.5 equiv of ROCl **5** was used in the *N*‐acylation (step i) to help ensure complete conversion.

We next went on to examine more complex reaction systems and test whether the products could be further elaborated in successive ring expansion reactions (Scheme 6). Additional starting materials were required for this phase of work, all of which were either commercially available, or easily prepared via literature routes, with further details included in the Supporting Information (Scheme 6 box). The yields given in Scheme 6 refer to the overall acylation/deprotection/ring expansion sequence and are all real synthetic yields of purified products following column chromatography. Some examples (indicated with a superscripted “a” or “b”) required more than the standard 1.5 equivalents of acid chloride for the N‐acylation to proceed to completion, but otherwise, all reactions were performed using the standard sets of conditions.

First, examples of the ring expansion were performed with lactams containing an ether linkage (**16 a**) and a benzannulated system (**16 b**), with both proceeding in good yield using the standard protocol. The high yielding synthesis of 12‐membered ring **16 c** is an interesting case, as this shows that the ring expansion can be performed using phenol nucleophiles, whilst branched hydroxyacid derivatives are also well tolerated (**16 d** and **16 e**). Also, whilst not a ring expansion reaction, the insertion of acid chloride **5 b** into linear amide **4 m** to make **16 f** shows that the rearrangement is not restricted to cyclic amides.

We then went on to examine successive ring expansion reactions. In total, 12 macrocyclic lactones in a range of ring sizes (14–21‐membered rings) were prepared in consistently high yields via the expansion of lactams for a second time (**17 a**–**l**, 61–98 %), involving the installation of various combinations of α‐ and β‐hydroxyacid derived linear fragments. We were especially pleased to discover that the new methods and products are compatible with our published lactam SuRE method; macrocycles were formed which involved the insertion of amino acid‐based linear fragments in lactams before (**17 f**,**h**) and after (**17 d**,**k**,**l**) ring expansion using a hydroxy acid derivative. The ability to install both lactone and lactam motifs into the ring expanded products (in any order) is important, as this significantly increases the freedom with which functional macrocycles can be designed and prepared using the SuRE method; for example, this could have important implications for its use in the preparation of azaketolide‐type antibiotics (e.g. **2** and **3**, Figure 1).

We also prepared 5 macrocyclic lactones (18–24‐membered rings) that demonstrate that the rings can be expanded for a third time (**18 a**–**e**). Triple ring‐expanded product **18 a** was formed in a relatively modest 36 % yield, with 35 % of the starting lactam **17 c** being recovered from the reaction due to incomplete *N*‐acylation in this case, even after adding additional doses of acid chloride **5 a**. Whilst the yield in this example was somewhat disappointing, it is perhaps inevitable that there will be some variation in the efficiency of the *N*‐acylation step, especially in larger ring systems where the conformation of the starting material may impact upon the ease with which the acid chloride approaches the lactam. Nonetheless, we were pleased that once formed, the *N*‐acylated material underwent hydrogenolysis and ring expansion as expected, enabling the isolation of the highly oxygenated trilactone **18 a**. Furthermore, we were delighted to discover that the reactions proceeded more smoothly for the preparation of products **18 b**–**e**, which were formed in much higher yields (68–84 %) using both α‐ and β‐hydroxyacid derived linear fragments, and including examples which had previously been expanded with amino acid derivatives to form mixed lactam/lactone macrocycles **18 c** and **18 e**.

**Scheme 7:**
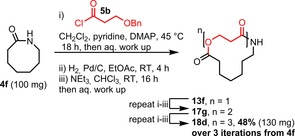
Telescoped triple ring expansion of lactam **4 f** into macrocycle **18 d**.

Finally, to further demonstrate the ease and practicality of the SuRE method, we performed the preparation of one of the triple ring expanded products (**18 d**) without chromatographic purification at any of the intermediate stages. To help ensure complete *N*‐acylation in each iteration, three equivalents of acid chloride **5 b** were used in this telescoped reaction sequence (rather than the usual 1.5 equivalents), but otherwise, no changes were made to the standard protocol other than not performing any chromatography until after the final iteration. Thus, lactam **4 f** was *N*‐acylated with acid chloride **5 b**, and following a short aqueous work up, taken on directly to hydrogenolysis with palladium on carbon in ethyl acetate. Following this, filtration, a solvent switch (ethyl acetate to CHCl_3_), stirring overnight with triethylamine and aqueous work completed the first iteration. This furnished crude product **13 f**, which was simply reacted in the same way (to form crude **17 g**) and then again, to form crude **18 d**, which was finally purified by column chromatography and isolated in 48 % overall yield over the three complete iterations (Scheme 7).

## Conclusion

In summary, our new lactone‐forming SuRE reaction system has been demonstrated in a range of high yielding ring successive ring expansion reactions. It has also been shown to be compatible with our published lactam‐forming SuRE method, enabling mixed lactam and lactone‐containing macrocycles using a versatile, practical protocol. Crucially, none of the methods rely on specialised reaction conditions or high dilution at any stage, with all the reactions described in this manuscript having been performed at 0.1 m concentration. Whilst all the steps in the overall SuRE process are relatively simple when considered individually (and indeed, conceptually related lactone forming ring expansion processes have been described previously),^[20^ we know of no other study in which such an array of complex ring expanded lactones can be assembled with the ease described in this manuscript, and none in which the ring expansion reactions can be performed iteratively. Thus, we view the relative simplicity of our SuRE method to be a key strength. The freedom to install precise sequences of lactone and lactam containing linear fragments into macrocycles in this way, and the ability to scale up the reactions if required,^[29^ is expected to be of high value in the myriad scientific fields that rely on the design and synthesis of functionalised macrocycles.

## Experimental Section

Full synthetic detail and spectroscopic data for all compounds are provided in the Supporting Information. General procedures A, B and C (Scheme 6) are also included below:

### Procedure A

A mixture of lactam (1 mmol), DMAP (0.1 mmol) and pyridine (6 mmol) in DCM (7 mL) under an argon atmosphere was stirred at RT for 5 mins. Next, a solution of acid chloride **5** in DCM (3.5 mL) was added and the resulting mixture was heated, at reflux, at 50 °C for 16 h. The solvent was concentrated in vacuo, loaded onto a short silica plug and eluted with hexane:ethyl acetate, to remove the majority of excess carboxylic acid and pyridine residues, and concentrated in vacuo. This material was re‐dissolved in THF (10 mL) and placed under an argon atmosphere. Palladium on carbon (100 mg, Pd 10 % on carbon) and water (2 mL) was then added and the reaction vessel was backfilled with hydrogen (via balloon) several times, then stirred at RT under a slight positive pressure of hydrogen (balloon). The reaction was then purged with argon, filtered through Celite, washed with methanol where the solvent was removed in vacuo. The crude material was then re‐dissolved in chloroform (10 mL) and triethylamine (1.5 mmol) added, and stirred at RT for 16 h, then reduced in vacuo and purified by flash column chromatography.

### Procedure B

A mixture of lactam (1 mmol), DMAP (0.1 mmol) and pyridine (6 mmol) in DCM (7 mL) under an argon atmosphere was stirred at RT for 5 mins. Next, a solution of acid chloride **5** in DCM (3.5 mL) was added and the resulting mixture was heated, at reflux, at 50 °C for 16 h. The solvent was concentrated in vacuo, loaded onto a short silica plug and eluted with hexane:ethyl acetate, to remove the majority of excess carboxylic acid and pyridine residues, and concentrated in vacuo. This material was re‐dissolved in ethylacetate (10 mL) and placed under an argon atmosphere. Palladium on carbon (100 mg, Pd 10 % on carbon) was then added and the reaction vessel was backfilled with hydrogen (via balloon) several times, then stirred at RT under a slight positive pressure of hydrogen (balloon). The reaction was then purged with argon, filtered through Celite, washed with methanol where the solvent was removed in vacuo. The crude material was then re‐dissolved in chloroform (10 mL) and triethylamine (1.5 mmol) added, and stirred at RT for 16 h, then reduced in vacuo and purified by flash column chromatography.

### Procedure C

A mixture of lactam (1 mmol), DMAP (0.1 mmol) and pyridine (6 mmol) in DCM (7 mL) under an argon atmosphere was stirred at RT for 5 mins. Next, a solution of acid chloride **5** (1.5 mmol) in DCM (3.5 mL) was added and the resulting mixture was heated, at reflux, at 50 °C for 16 h. The solvent was then concentrated in vacuo, loaded onto a short silica plug and eluted with 2:1 hexane:ethyl acetate, to remove the majority of excess carboxylic acid and pyridine residues, and concentrated in vacuo. This material was re‐dissolved in DCM (10 mL) and placed under an argon atmosphere. DBU (10 mmol) was then added and stirred at RT for 16 h, then reduced in vacuo and purifiied by flash column chromatography.

## Conflict of interest

The authors declare no conflict of interest.

## Supporting information

As a service to our authors and readers, this journal provides supporting information supplied by the authors. Such materials are peer reviewed and may be re‐organized for online delivery, but are not copy‐edited or typeset. Technical support issues arising from supporting information (other than missing files) should be addressed to the authors.

Supplementary
