# Supplementary material for: Iterative Assembly of Macrocyclic Lactones using Successive Ring Expansion Reactions
Source: Chemistry. 2018 Aug 19;24(52):13947–53. doi: 10.1002/chem.201803064 (PMC6334170; doi:10.1002/chem.201803064)
Supplement: Supplementary file 1 — Supplementary [file CHEM-24-13947-s001.pdf]

CHEMISTRY
A European Journal

Supporting Information

Iterative Assembly of Macrocyclic Lactones using Successive Ring Expansion Reactions

Thomas C. Stephens, Aggie Lawer, Thomas French, and William P. Unsworth*[a]

chem_201803064_sm_miscellaneous_information.pdf

Table of Contents

Table of Contents	Page
General information	1
List of starting materials	2
General procedure for acid chloride formation	3
Characterisation data and procedures	3–54
^{1}H and ^{13}C NMR spectra	55–120
Computational chemistry	121–139
References	140

General Information

Except where stated, all reagents were purchased from commercial sources and used without further purification. Except where stated, all experimental procedures were carried out under an atmosphere of argon. Anhydrous CH_2Cl_2 was obtained from an Innovative Technology Inc. PureSolv® solvent purification system. ^{1}H NMR and ^{13}C NMR spectra were recorded on a JEOL ECX400 or JEOL ECS400 spectrometer, operating at 400 MHz and 100 MHz, respectively. All spectral data was acquired at 295 K unless stated. Chemical shifts (δ) are quoted in parts per million (ppm). The residual solvent peak, δ_H 7.26 and δ_C 77.0 for $CDCl_3$ was used as a reference. Coupling constants (*J*) are reported in Hertz (Hz) to the nearest 0.1 Hz. The multiplicity abbreviations used are: s singlet, d doublet, t triplet, q quartet, m multiplet. Signal assignment was achieved by analysis of DEPT, COSY, NOESY, HMBC and HSQC experiments where required. Infrared (IR) spectra were recorded on a PerkinElmer UATR two spectrometer as a thin film. Mass-spectra (low and high-resolution) were obtained by the University of York Mass Spectrometry Service, using electrospray ionisation (ESI) on a Bruker Daltonics, Micro-tof spectrometer. Melting points were determined using Gallenkamp apparatus and are uncorrected. Thin layer chromatography was carried out on Merck silica gel 60F254 pre-coated aluminium foil sheets and were visualised using UV light (254 nm) and stained with basic aqueous potassium permanganate. Flash column chromatography was carried out using slurry packed Fluka silica gel (SiO_2), 35–70 µm, 60 Å, under a light positive pressure, eluting with the specified solvent system.

List of Starting Materials

All the starting materials used in this publication are listed below. Commercially available starting materials (denoted with a *) were used as supplied, those with a reference number are known compounds prepared via the cited literature method, while for all others, preparative details and spectroscopic characterisation data are provided.

Lactams/amide 4a-m, 14a-b and 15

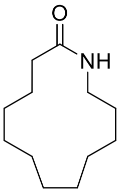

4a*

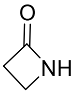

4b*

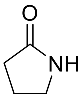

4c*

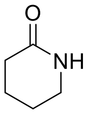

4d*

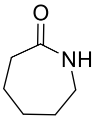

4e*

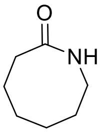

4f*

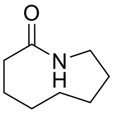

4g[1]

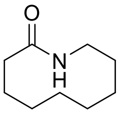

4h[1]

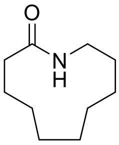

4i[1]

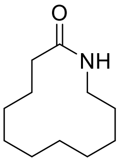

4j*

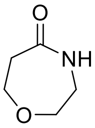

4k[2]

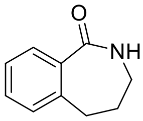

4l*

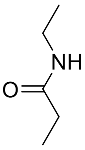

4m*

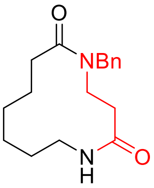

14a[1]

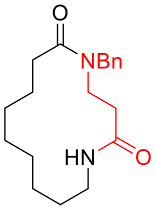

14b[1]

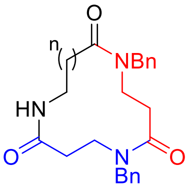

15, n=9[1]

Linear carboxylic acids S1–S7 used to make acid chlorides 5a-g

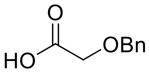

S1[1]
(→ ROCl **5a**)

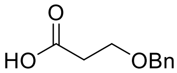

S2[1]
(→ ROCl **5b**)

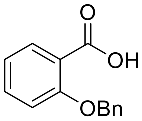

S3*
(→ ROCl **5c**)

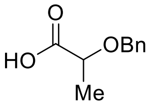

S4[3]
(→ ROCl **5d**)

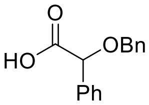

S5[4]
(→ ROCl **5e**)

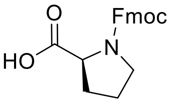

S6*
(→ ROCl **5f**)

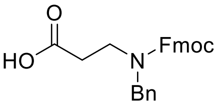

S7[1]
(to make ROCl **5g**)

General procedure for acid chloride formation[1]

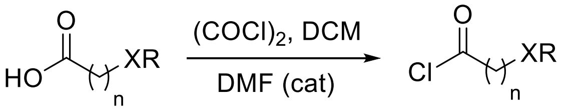

Oxalyl chloride (3 mmol) was added to a suspension of carboxylic acid (1 mmol) in DCM (5 mL), followed by a catalytic amount of DMF (1 drop/mmol of carboxylic acid). The resulting mixture was stirred at RT for 1 h and concentrated *in vacuo* to remove all the solvent and excess oxalyl chloride.

Characterisation data and procedures: *N*-acylation reactions

Details of the *N*-acylation reactions to prepare imides **6a**, **6i** and **6j**, can be found within the procedures for their ring-expanded derivatives **12a**, **12i** and **12j** respectively, as in these three cases, the imides **6a**, **6i** and **6j**, did not require chromatographic purification and hence were taken on directly to hydrogenolysis and ring expansion without purification.

1-(2-(Benzyloxy)acetyl)azetidin-2-one (6b)

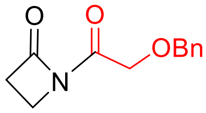

A mixture of 2-azetidinone (56 mg, 0.786 mmol), DMAP (10 mg, 0.0786 mmol) and pyridine (380 µL, 4.72 mmol) in DCM (5.5 mL) under an argon atmosphere was stirred at RT for 5 mins. Next, a solution of acid chloride (1.18 mmol, 1.50 eqv prepared using the general procedure) in DCM (3 mL) was added and the resulting mixture was heated, at reflux, at 50 °C for 16 h. The mixture was allowed to cool before the solvent was removed *in vacuo*. Purification by flash column chromatography (SiO_2, 5:1 hexane:ethyl acetate → 2:1 hexane:ethyl acetate) afforded the *title compound* as a colourless oil (145 mg, 87%); R_f 0.66 (ethyl acetate); ν_{max}/cm^{-1} (thin film) 2981, 2870, 1781, 1706; δ_H (400 MHz, $CDCl_3$) 7.42–7.22 (5H, m, Ph), 4.63 (2H, s, C**H$_2$**Ph), 4.42 (2H, s, C**H$_2$**OBn), 3.55 (2H, t, *J* = 5.3 Hz, C**H$_2$**N), 2.99 (2H, t, *J* = 5.3 Hz, C**H$_2$**CO); δ_C (100 MHz, $CDCl_3$) 168.1 (**C**O), 164.7 (**C**O), 137.1 (**C**), 128.6 (**C**H), 128.2 (**C**H), 128.1 (**C**H), 73.6 (**C**H$_2$Ph), 69.7 (**C**H$_2$OBn), 36.2 (**C**H$_2$), 36.0 (**C**H$_2$); HRMS (ESI): calcd. for $C_{12}H_{13}NNaO_3$, 242.0788. Found: $[MNa]^+$, 242.0787 (0.7 ppm error).

1-(2-(Benzyloxy)acetyl)pyrrolidin-2-one (6c)

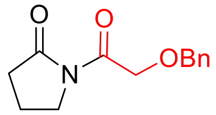

A mixture of pyrrolidin-2-one (60 µL, 0.786 mmol), DMAP (10 mg, 0.0786 mmol) and pyridine (380 µL, 4.72 mmol) in DCM (5.5 mL) under an argon atmosphere was stirred at RT for 5 mins. Next, a solution of acid chloride (1.18 mmol, 1.50 equiv. prepared using the general procedure) in DCM (3 mL) was added and the resulting mixture was heated, at reflux, at 50 °C for 16 h. The mixture was allowed to cool before the solvent was removed *in vacuo*. Purification by flash column chromatography (SiO_2, 2:1 hexane:ethyl acetate) afforded the *title compound* as a colourless oil (137 mg, 75%); Data consistent with those previously reported in the literature.[5]

1-(2-(Benzyloxy)acetyl)piperidin-2-one (6d)

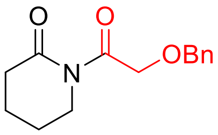

A mixture of δ-valerolactam (78 mg, 0.786 mmol), DMAP (10 mg, 0.0786 mmol) and pyridine (380 µL, 4.72 mmol) in DCM (5.5 mL) under an argon atmosphere was stirred at RT for 5 mins. Next, a solution of acid chloride (1.18 mmol, 1.50 equiv. prepared using the general procedure) in DCM (3 mL) was added and the resulting mixture was heated, at reflux, at 50 °C for 16 h. The mixture was allowed to cool before the solvent was removed *in vacuo*. Purification by flash column chromatography (SiO_2, 2:1 hexane:ethyl acetate) afforded the *title compound* as a yellow oil (193 mg, 99%); R_f 0.65 (ethyl acetate); ν_{max}/cm^{-1} (thin film) 2950, 1690; δ_H (400 MHz, $CDCl_3$) 7.47–7.20 (5H, m, Ph), 4.63 (2H, s, C**H$_2$**O), 4.62 (2H, s, C**H$_2$**O), 3.80–3.66 (2H, m, C**H$_2$**NH), 2.59–2.45 (2H, m, C**H$_2$**CO), 1.90–1.71 (4H, m, 2 × C**H$_2$**); δ_C (100 MHz, $CDCl_3$) 174.3 (**C**O), 173.3 (**C**O), 137.8 (**C**), 128.5 (**C**H), 128.1 (**C**H), 127.9 (**C**H), 73.3 (**C**H$_2$O), 73.0 (**C**H$_2$O), 44.1 (**C**H$_2$NH), 34.5 (**C**H$_2$CO), 22.3 (**C**H$_2$), 20.2 (**C**H$_2$); HRMS (ESI): calcd. for $C_{14}H_{17}NNaO_3$, 270.1101. Found: $[MNa]^+$, 270.1105 (−1.8 ppm error).

1-(2-(Benzyloxy)acetyl)azepan-2-one (6e)

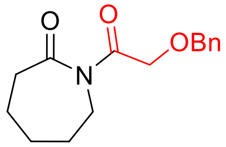

A mixture of ε-caprolactam (90 mg, 0.786 mmol), DMAP (10 mg, 0.0786 mmol) and pyridine (380 μL, 4.72 mmol) in DCM (5.5 mL) under an argon atmosphere was stirred at RT for 5 mins. Next, a solution of acid chloride (1.18 mmol, 1.50 equiv. prepared using the general procedure) in DCM (3 mL) was added and the resulting mixture was heated, at reflux, at 50 °C for 16 h. The mixture was allowed to cool before the solvent was removed *in vacuo*. Purification by flash column chromatography (SiO_2, 2:1 hexane:ethyl acetate) afforded the *title compound* as a yellow oil (203 mg, 98%); R_f 0.73 (ethyl acetate); ν_{max}/cm^{-1} (thin film) 2931, 2859, 1694; δ_H (400 MHz, $CDCl_3$) 7.48–7.19 (5H, m, Ph), 4.63 (2H, s, C**H₂**Ph), 4.60 (2H, s, C**H₂**OBn), 3.98–3.85 (2H, m, C**H₂**NH), 2.74–2.62 (2H, m, C**H₂**CO), 1.82–1.62 (6H, m, 3 × C**H₂**); δ_C (100 MHz, $CDCl_3$) 177.8 (**C**O), 173.5 (**C**O), 137.8 (**C**), 128.5 (**C**H), 128.1 (**C**H), 127.9 (**C**H), 73.3 (**C**H_2Ph), 72.9 (**C**H_2OBn), 43.1 (**C**H_2NH), 39.5 (**C**H_2CO), 29.2 (**C**H_2), 28.5 (**C**H_2), 23.6 (**C**H_2); HRMS (ESI): calcd. for $C_{15}H_{19}NNaO_3$, 284.1257. Found: $[MNa]^+$, 284.1254 (1.5 ppm error).

1-(2-(Benzyloxy)acetyl)azocan-2-one (6f)

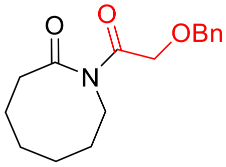

A mixture of azocan-2-one (100 mg, 0.786 mmol), DMAP (10 mg, 0.0786 mmol) and pyridine (380 μL, 4.72 mmol) in DCM (5.5 mL) under an argon atmosphere was stirred at RT for 5 mins. Next, a solution of acid chloride (1.18 mmol, 1.50 equiv. prepared using the general procedure) in DCM (3 mL) was added and the resulting mixture was heated, at reflux, at 50 °C for 16 h. The mixture was allowed to cool before the solvent was removed *in vacuo*. Purification by flash column chromatography (SiO_2, 2:1 hexane:ethyl acetate) afforded the *title compound* as a yellow oil (216 mg, 98%); R_f 0.74 (ethyl acetate); ν_{max}/cm^{-1} (thin film) 2925, 2859, 1687; δ_H (400 MHz, $CDCl_3$) 7.47–7.19 (5H, m, Ph), 4.63 (2H, s, C**H₂**Ph), 4.61 (2H, s, C**H₂**OBn), 4.00–3.86 (2H, m, C**H₂**NH), 2.68–2.57 (2H, m, C**H₂**CO), 1.91–1.79 (2H, m, C**H₂**), 1.77–1.67 (2H, m, C**H₂**), 1.61–1.52 (2H, m, C**H₂**), 1.48–1.39 (2H, m, C**H₂**); δ_C (100 MHz, $CDCl_3$) 178.2 (**C**O), 173.9 (**C**O), 137.8 (**C**), 128.5 (**C**H), 128.1 (**C**H), 127.9 (**C**H), 73.3

(CH_2Ph), 73.0 (CH_2OBn), 43.4 (CH_2NH), 37.0 (CH_2CO), 29.3 (CH_2), 29.0 (CH_2), 26.3 (CH_2), 24.1 (CH_2); HRMS (ESI): calcd. for $C_{16}H_{21}NNaO_3$, 298.1414. Found: $[MNa]^+$, 298.1417 (−0.9 ppm error).

1-(2-(Benzyloxy)acetyl)azonan-2-one (6g)

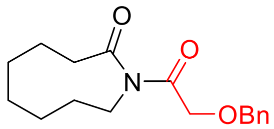

A mixture of azonan-2-one (111 mg, 0.786 mmol), DMAP (10 mg, 0.0786 mmol) and pyridine (380 μL, 4.72 mmol) in DCM (5.5 mL) under an argon atmosphere was stirred at RT for 5 mins. Next, a solution of acid chloride (1.18 mmol, 1.50 equiv. prepared using the general procedure) in DCM (3 mL) was added and the resulting mixture was heated, at reflux, at 50 °C for 16 h. The mixture was allowed to cool before the solvent was removed *in vacuo*. Purification by flash column chromatography (SiO_2, 2:1 hexane:ethyl acetate) afforded the *title compound* as a yellow oil (213 mg, 93%); R_f 0.77 (ethyl acetate); ν_{max}/cm^{-1} (thin film) 3030, 2926, 2864, 1687; δ_H (400 MHz, $CDCl_3$) 7.46–7.17 (5H, m, Ph), 4.62 (2H, s, C**H$_2$**O), 4.58 (2H, s, C**H$_2$**O), 3.94–3.82 (2H, m, C**H$_2$**N), 2.68–2.57 (2H, m, C**H$_2$**CO), 1.91–1.81 (2H, m, C**H$_2$**), 1.80–1.72 (2H, m, C**H$_2$**), 1.65–1.55 (2H, m, C**H$_2$**), 1.54–1.46 (2H, m, C**H$_2$**), 1.45–1.37 (2H, m, C**H$_2$**); δ_C (100 MHz, $CDCl_3$) 179.2 (**C**O), 173.6 (**C**O), 137.7 (**C**), 128.5 (**C**H), 128.1 (**C**H), 127.9 (**C**H), 73.4 (**C**H$_2$O), 72.5 (**C**H$_2$O), 45.0 (**C**H$_2$N), 38.4 (**C**H$_2$CO), 28.8 (**C**H$_2$), 27.6 (**C**H$_2$), 25.7 (**C**H$_2$), 25.4 (**C**H$_2$), 21.7 (**C**H$_2$); HRMS (ESI): calcd. for $C_{17}H_{23}NNaO_3$, 312.1570. Found: $[MNa]^+$, 312.1576 (−2.8 ppm error).

1-(2-(Benzyloxy)acetyl)azecan-2-one (6h)

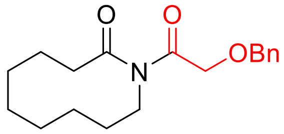

A mixture of azecan-2-one (122 mg, 0.786 mmol), DMAP (10 mg, 0.0786 mmol) and pyridine (380 μL, 4.72 mmol) in DCM (5.5 mL) under an argon atmosphere was stirred at RT for 5 mins. Next, a solution of acid chloride (1.18 mmol, 1.50 equiv. prepared using the general procedure) in DCM (3 mL) was added and the resulting mixture was heated, at reflux, at 50 °C for 16 h. The mixture was allowed to cool before the solvent was removed *in vacuo*. Purification by flash column chromatography (SiO_2, 2:1 hexane:ethyl acetate) afforded the *title compound* as a yellow oil (229 mg, 96%); R_f 0.78 (ethyl acetate); ν_{max}/cm^{-1} (thin film) 2929,

2869, 1687; δ_H (400 MHz, $CDCl_3$) 7.44–7.21 (5H, m, Ph), 4.61 (2H, s, C**H$_2$**O), 4.55 (2H, s, C**H$_2$**O), 3.96–3.85 (2H, m, C**H$_2$**N), 2.80–2.70 (2H, m, C**H$_2$**CO), 1.91–1.79 (2H, m, C**H$_2$**), 1.76–1.67 (2H, m, C**H$_2$**), 1.52–1.38 (6H, m, 3 × C**H$_2$**), 1.37–1.23 (2H, m, C**H$_2$**); δ_C (100 MHz, $CDCl_3$) 178.2 (**C**O), 174.1 (**C**O), 137.6 (**C**), 128.5 (**C**H), 128.1 (**C**H), 127.9 (**C**H), 73.4 (**C**H-$_2$O), 72.5 (**C**H_2O), 43.9 (**C**H_2N), 34.4 (**C**H_2CO), 26.6 (**C**H_2), 26.2 (**C**H_2), 25.3 (**C**H_2), 25.2 (**C**H_2), 22.3 (**C**H_2), 20.2 (**C**H_2); HRMS (ESI): calcd. for $C_{18}H_{25}NNaO_3$, 326.1727. Found: $[MNa]^+$, 326.1728 (−0.6 ppm error).

1-(3-(Benzyloxy)propanoyl)azacyclotridecan-2-one (7a)

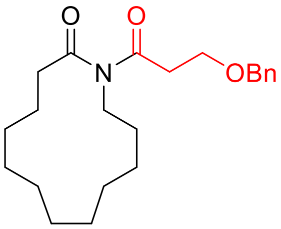

A mixture of laurolactam (155 mg, 0.786 mmol), DMAP (10 mg, 0.0786 mmol) and pyridine (380 µL, 4.72 mmol) in DCM (5.5 mL) under an argon atmosphere was stirred at RT for 5 mins. Next, a solution of acid chloride (1.18 mmol, 1.50 equiv. prepared using the general procedure) in DCM (3 mL) was added and the resulting mixture was heated, at reflux, at 50 °C for 16 h. The mixture was allowed to cool then diluted with DCM (50 mL), washed with 10% $HCl_{(aq)}$ (15 mL) and $NaHCO_{3(aq)}$ (2 × 20 mL), dried over $MgSO_4$ and concentrated *in vacuo*. Purification by flash column chromatography (SiO_2, 10:1 hexane:ethyl acetate → 5:1 hexane:ethyl acetate → 2:1 hexane:ethyl acetate) afforded the *title compound* as a white foam (214 mg, 79%); R_f 0.56 (dichloromethane); ν_{max}/cm^{-1} (thin film) 2929, 2860, 1690; δ_H (400 MHz, $CDCl_3$) 7.39–7.19 (5H, m, Ph), 4.52 (2H, s, C**H$_2$**Ph), 3.80 (2H, t, J = 6.0 Hz, C**H$_2$**OBn), 3.68 (2H, t, J = 7.3 Hz, C**H$_2$**NH), 3.07 (2H, t, J = 6.0 Hz, C**H$_2$**CH_2OBn), 2.62–2.53 (2H, m, C**H$_2$**CO), 1.81–1.70 (2H, m, C**H$_2$**), 1.69–1.60 (2H, m, C**H$_2$**), 1.49–1.23 (14H, m, 7 × C**H$_2$**); δ_C (100 MHz, $CDCl_3$) 177.0 (**C**O), 174.5 (**C**O), 138.4 (**C**), 128.5 (**C**H), 127.8 (**C**H), 127.7 (**C**H), 73.3 (**C**H_2Ph), 66.1 (**C**H_2OBn), 43.1 (**C**H_2NH), 39.2 (**C**$H_2$$CH_2$OBn), 36.2 (**C**$H_2$CO), 26.0 (**C**$H_2$), 25.9 (**C**$H_2$), 25.7 (**C**$H_2$), 25.1 (**C**$H_2$), 24.8 (**C**$H_2$), 24.7 (**C**$H_2$), 24.2 (**C**$H_2$), 24.1 (**C**$H_2$), 23.8 (**C**$H_2$); HRMS (ESI): calcd. for $C_{22}H_{33}NNaO_3$, 382.2353. Found: $[MNa]^+$, 382.2358 (−1.9 ppm error).

1-(3-(Benzyloxy)propanoyl)azetidin-2-one (7b)

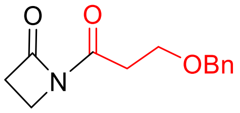

A mixture of 2-azetidinone (56 mg, 0.786 mmol), DMAP (10 mg, 0.0786 mmol) and pyridine (380 μL, 4.72 mmol) in DCM (5.5 mL) under an argon atmosphere was stirred at RT for 5 mins. Next, a solution of acid chloride (1.18 mmol, 1.50 equiv. prepared using the general procedure) in DCM (3 mL) was added and the resulting mixture was stirred at RT for 16 h. The mixture was then diluted with DCM (50 mL), washed with 10% $HCl_{(aq)}$ (15 mL) and $NaHCO_{3(aq)}$ (2 × 20 mL), dried over $MgSO_4$ and concentrated *in vacuo*. Purification by flash column chromatography (SiO_2, 2:1 hexane:ethyl acetate) afforded the *title compound* as a yellow oil (167 mg, 91%); R_f 0.64 (ethyl acetate); ν_{max}/cm^{-1} (thin film) 1784, 1698; δ_H (400 MHz, $CDCl_3$) 7.42–7.19 (5H, m, Ph), 4.53 (2H, s, C**H**$_2$Ph), 3.80 (2H, t, *J* = 6.1 Hz, C**H**$_2$NH), 3.57 (2H, t, *J* = 5.3 Hz, C**H**$_2$OBn), 3.06–2.94 (4H, m, C**H**$_2$CH$_2$OBn and C**H**$_2$CON); δ_C (100 MHz, $CDCl_3$) 168.9 (**C**O), 165.1 (**C**O), 138.2 (**C**), 128.5 (**C**H), 127.8 (**C**H), 127.7 (**C**H), 73.1 (**C**H$_2$Ph), 64.7 (**C**H$_2$OBn), 37.1 (**C**H$_2$), 36.7 (**C**H$_2$), 36.0 (**C**H$_2$); HRMS (ESI): calcd. for $C_{13}H_{15}NNaO_3$, 256.0944. Found: $[MNa]^+$, 256.0945 (−1.1 ppm error).

1-(3-(Benzyloxy)propanoyl)pyrrolidin-2-one (7c)

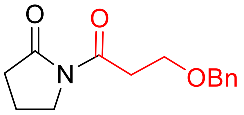

A mixture of pyrrolidinone (60 μL, 0.786 mmol), DMAP (10 mg, 0.0786 mmol) and pyridine (380 μL, 4.72 mmol) in DCM (5.5 mL) under an argon atmosphere was stirred at RT for 5 mins. Next, a solution of acid chloride (1.18 mmol, 1.50 equiv. prepared using the general procedure) in DCM (3 mL) was added and the resulting mixture was heated, at reflux, at 50 °C for 16 h. The mixture was allowed to cool before the solvent was removed *in vacuo*. Purification by flash column chromatography (SiO_2, 5:1 hexane:ethyl acetate → 2:1 hexane:ethyl acetate) afforded the *title compound* as a colourless oil (149 mg, 77%). R_f 0.65 (ethyl acetate); ν_{max}/cm^{-1} (thin film); 2870, 1734, 1687; δ_H (400 MHz, $CDCl_3$) 7.38–7.17 (5H, m, Ph), 4.51 (2H, s, C**H**$_2$Ph), 3.87–3.68 (4H, m, C**H**$_2$N and C**H**$_2$OBn), 3.19 (2H, t, *J* = 6.1 Hz, (CH$_2$)$_2$C**H**$_2$CO), 2.52 (2H, t, *J* = 8.0 Hz, C**H**$_2$CH$_2$OBn), 2.05–1.88 (2H, m, C**H**$_2$CH$_2$N); δ_C (100 MHz, $CDCl_3$) 175.6 (**C**O), 172.0 (**C**O), 138.4 (**C**), 128.4 (**C**H), 127.8 (**C**H), 127.7 (**C**H), 73.1 (**C**H$_2$Ph), 65.1 (**C**H$_2$OBn), 45.5 (**C**H$_2$N), 37.4 (**C**H$_2$CO), 33.7 (**C**H$_2$CO), 17.2 (**C**H$_2$CH$_2$N); HRMS (ESI): calcd. for $C_{14}H_{18}NO_3$, 248.1281. Found: $[MH]^+$, 248.1285 (−1.0 ppm error).

1-(3-(Benzyloxy)propanoyl)piperidin-2-one (7d)

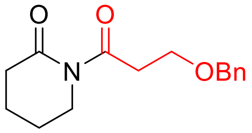

A mixture of 2-piperidinone (78 mg, 0.786 mmol), DMAP (10 mg, 0.0786 mmol) and pyridine (380 µL, 4.72 mmol) in DCM (5.5 mL) under an argon atmosphere was stirred at RT for 5 mins. Next, a solution of acid chloride (1.18 mmol, 1.50 equiv. prepared using the general procedure) in DCM (3 mL) was added and the resulting mixture was heated, at reflux, at 50 °C for 16 h. The mixture was allowed to cool before the solvent was removed *in vacuo*. Purification by flash column chromatography (SiO_2, 5:1 hexane:ethyl acetate → 2:1 hexane:ethyl acetate) afforded the *title compound* as a colourless oil (170 mg, 83%). R_f 0.63 (ethyl acetate); ν_{max}/cm^{-1} (thin film) 2872, 1692; δ_H (400 MHz, $CDCl_3$) 7.40–7.21 (5H, m, Ph), 4.53 (2H, s, C**H2**Ph), 3.79 (2H, t, *J* = 6.1 Hz, C**H2**OBn), 3.74–3.67 (2H, m, C**H2**N), 3.22 (2H, t, *J* = 6.1 Hz, C**H2**CH2OBn), 2.57 (2H, m, C**H2**), 1.87–1.73 (4H, m, 2 × C**H2**); δ_C (100 MHz, $CDCl_3$) 174.8 (**C**O), 173.5 (**C**O), 138.4 (**C**), 128.5 (**C**H), 127.8 (**C**H), 127.7 (**C**H), 73.2 (**C**H2Ph), 65.8 (**C**H2OBn), 44.1 (**C**H2N), 40.2 (**C**H2), 34.9 (**C**H2), 22.5 (**C**H2), 20.4 (**C**H2); HRMS (ESI): calcd. for $C_{15}H_{20}NO_3$, 262.1438. Found: $[MH]^+$, 262.1441 (−2.0 ppm error).

1-(3-(Benzyloxy)propanoyl)azepan-2-one (7e)

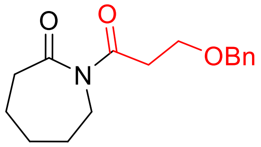

A mixture of ε-caprolactam (90 mg, 0.786 mmol), DMAP (10 mg, 0.0786 mmol) and pyridine (380 µL, 4.72 mmol) in DCM (5.5 mL) under an argon atmosphere was stirred at RT for 5 mins. Next, a solution of acid chloride (1.18 mmol, 1.50 equiv. prepared using the general procedure) in DCM (3 mL) was added and the resulting mixture was heated, at reflux, at 50 °C for 16 h. The mixture was allowed to cool before the solvent was removed *in vacuo*. Purification by flash column chromatography (SiO_2, 5:1 hexane:ethyl acetate → 2:1 hexane:ethyl acetate) afforded the *title compound* as a colourless oil (194 mg, 90%); R_f 0.70 (ethyl acetate); ν_{max}/cm^{-1} (thin film) 2930, 2860, 1694; δ_H (400 MHz, $CDCl_3$) 7.41–7.20 (5H, m, Ph), 4.52 (2H, s, C**H2**Ph), 3.94–3.84 (2H, m, C**H2**N), 3.79 (2H, t, *J* = 6.1 Hz, C**H2**OBn), 3.19 (2H, t, *J* = 6.1 Hz, C**H2**CH2OBn), 2.71–2.65 (2H, m, C**H2**), 1.80–1.60 (6H, m, 3 × C**H2**); δ_C (100 MHz, $CDCl_3$) 177.9 (**C**O), 174.1 (**C**O), 138.4 (**C**), 128.4 (**C**H), 127.8 (**C**H), 127.7 (**C**H), 73.2 (**C**H2Ph), 66.0 (**C**H2OBn), 43.4 (**C**H2N), 39.83 (**C**H2CO), 39.75 (**C**H2CO), 29.3

($\mathbf{C}H_2$), 28.6 ($\mathbf{C}H_2$), 23.7 ($\mathbf{C}H_2$); HRMS (ESI): calcd. for $C_{16}H_{22}NO_3$, 276.1594. Found: $[MH]^+$, 276.1598 (−1.4 ppm error).

1-(3-(Benzyloxy)propanoyl)azocan-2-one (7f)

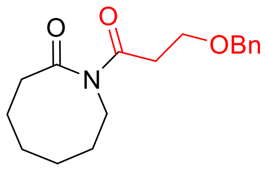

A mixture of azocan-2-one (100 mg, 0.786 mmol), DMAP (10 mg, 0.0786 mmol) and pyridine (380 μL, 4.72 mmol) in DCM (5.5 mL) under an argon atmosphere was stirred at RT for 5 mins. Next, a solution of acid chloride (1.18 mmol, 1.50 equiv. prepared using the general procedure) in DCM (3 mL) was added and the resulting mixture was heated, at reflux, at 50 °C for 16 h. The mixture was allowed to cool before the solvent was removed *in vacuo*. Purification by flash column chromatography (SiO_2, 5:1 hexane:ethyl acetate → 2:1 hexane:ethyl acetate) afforded the *title compound* as a colourless oil (206 mg, 91%); R_f 0.72 (ethyl acetate); ν_{max}/cm^{-1} (thin film) 2927, 2859, 1688; δ_H (400 MHz, $CDCl_3$) 7.38–7.20 (5H, m, Ph), 4.52 (2H, s, $C\mathbf{H_2}$Ph), 3.93–3.84 (2H, m, $C\mathbf{H_2}$N), 3.79 (2H, t, J = 6.1 Hz, $C\mathbf{H_2}$OBn), 3.17 (2H, t, J = 6.1 Hz, $C\mathbf{H_2}CH_2$OBn), 2.66–2.58 (2H, m, $C\mathbf{H_2}$CO), 1.90–1.81 (2H, m, $C\mathbf{H_2}$), 1.74–1.64 (2H, m, $C\mathbf{H_2}$), 1.60–1.53 (2H, m, $C\mathbf{H_2}$), 1.47–1.39 (2H, m, $C\mathbf{H_2}$); δ_C (100 MHz, $CDCl_3$) 178.5 ($\mathbf{C}$O), 174.7 ($\mathbf{C}$O), 138.4 ($\mathbf{C}$), 128.4 ($\mathbf{C}$H), 127.8 ($\mathbf{C}$H), 127.7 ($\mathbf{C}$H), 73.2 ($\mathbf{C}H_2$Ph), 66.0 ($\mathbf{C}H_2$OBn), 43.5 ($\mathbf{C}H_2$N), 39.9 ($\mathbf{C}H_2CH_2$OBn), 37.1 ($\mathbf{C}H_2$CO), 29.7 ($\mathbf{C}H_2$), 29.2 ($\mathbf{C}H_2$), 26.3 ($\mathbf{C}H_2$), 24.0 ($\mathbf{C}H_2$); HRMS (ESI): calcd. for $C_{17}H_{24}NO_3$, 290.1751. Found: $[MH]^+$, 290.1756 (−1.8 ppm error).

1-(3-(Benzyloxy)propanoyl)azonan-2-one (7g)

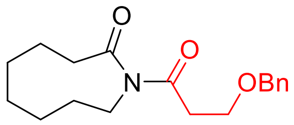

A mixture of azonan-2-one (111 mg, 0.786 mmol), DMAP (10 mg, 0.0786 mmol) and pyridine (380 μL, 4.72 mmol) in DCM (5.5 mL) under an argon atmosphere was stirred at RT for 5 mins. Next, a solution of acid chloride (1.18 mmol, 1.50 equiv. prepared using the general procedure) in DCM (3 mL) was added and the resulting mixture was heated, at reflux, at 50 °C for 16 h. The mixture was allowed to cool before the solvent was removed *in vacuo*. Purification by flash column chromatography (SiO_2, 5:1 hexane:ethyl acetate → 2:1

hexane:ethyl acetate) afforded the *title compound* as a colourless oil (222 mg, 93%); R_f 0.77 (ethyl acetate); ν_{max}/cm^{-1} (thin film) 2927, 2867, 1688; δ_H (400 MHz, $CDCl_3$) 7.38–7.20 (5H, m, Ph), 4.51 (2H, s, C**H₂**Ph), 3.89–3.82 (2H, m, C**H₂**N), 3.79 (2H, t, *J* = 6.1 Hz, C**H₂**OBn), 3.09 (2H, t, *J* = 6.1 Hz, C**H₂**CH_2OBn), 2.67–2.58 (2H, m, C**H₂**CO), 1.92–1.81 (2H, m, C**H₂**), 1.79–1.70 (2H, m, C**H₂**), 1.69–1.59 (2H, m, C**H₂**), 1.52–1.43 (2H, m, C**H₂**), 1.42–1.33 (2H, m, C**H₂**); δ_C (100 MHz, $CDCl_3$) 180.1 (**C**O), 174.1 (**C**O), 138.4 (**C**), 128.4 (**C**H), 127.8 (**C**H), 127.7 (**C**H), 73.3 (**C**H_2Ph), 66.1 (**C**H_2OBn), 44.8 (**C**H_2N), 39.02 (**C**H_2CO), 38.98 (**C**H_2CO), 28.8 (**C**H_2), 28.0 (**C**H_2), 26.0 (**C**H_2), 25.5 (**C**H_2), 21.2 (**C**H_2); HRMS (ESI): calcd. for $C_{18}H_{26}NO_3$, 304.1907. Found: $[MH]^+$, 304.1908 (−0.2 ppm error).

1-(3-(Benzyloxy)propanoyl)azecan-2-one (7h)

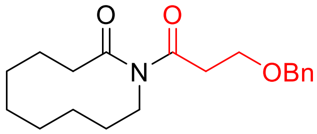

A mixture of azecan-2-one (122 mg, 0.786 mmol), DMAP (10 mg, 0.0786 mmol) and pyridine (380 μL, 4.72 mmol) in DCM (5.5 mL) under an argon atmosphere was stirred at RT for 5 mins. Next, a solution of acid chloride (1.18 mmol, 1.50 equiv. prepared using the general procedure) in DCM (3 mL) was added and the resulting mixture was heated, at reflux, at 50 °C for 16 h. The mixture was allowed to cool then diluted with DCM (50 mL), washed with 10% $HCl_{(aq)}$ (15 mL) and $NaHCO_{3(aq)}$ (2 × 20 mL), dried over $MgSO_4$ and concentrated *in vacuo*. Purification by flash column chromatography (SiO_2, 5:1 hexane:ethyl acetate → 2:1 hexane:ethyl acetate) afforded the *title compound* as a yellow oil (212 mg, 87%); R_f 0.74 (ethyl acetate); ν_{max}/cm^{-1} (thin film) 2927, 2870, 1686; δ_H (400 MHz, $CDCl_3$) 7.40–7.20 (5H, m, Ph), 4.52 (2H, s, C**H₂**Ph), 3.91–3.85 (2H, m, C**H₂**N), 3.82 (2H, t, *J* = 6.1 Hz, C**H₂**OBn), 2.99 (2H, t, *J* = 6.1 Hz, C**H₂**CH_2OBn), 2.84–2.76 (2H, m, $(CH_2)_7$C**H₂**CO), 1.86–1.77 (2H, m, C**H₂**), 1.75–1.67 (2H, m, C**H₂**), 1.52–1.33 (8H, m, 4 × C**H₂**); δ_C (100 MHz, $CDCl_3$) 179.6 (**C**O), 174.5 (**C**O), 138.3 (**C**), 128.5 (**C**H), 127.8 (**C**H), 127.7 (**C**H), 73.4 (**C**H_2Ph), 66.2 (**C**H_2OBn), 44.5 (**C**H_2N), 38.4 (**C**H_2CH_2OBn), 37.0 (**C**H_2CO), 25.6 (**C**H_2), 25.4 (**C**H_2), 25.0 (**C**H_2), 24.3 (**C**H_2), 23.7 (**C**H_2), 22.2 (**C**H_2); HRMS (ESI): calcd. for $C_{19}H_{27}NNaO_3$, 340.1883. Found: $[MNa]^+$, 340.1885 (−0.4 ppm error).

1-(3-(Benzyloxy)propanoyl)azacycloundecan-2-one (7i)

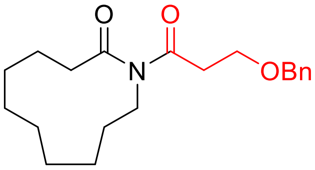

A mixture of azacycloundecan-2-one (133 mg, 0.786 mmol), DMAP (10 mg, 0.0786 mmol) and pyridine (380 µL, 4.72 mmol) in DCM (5.5 mL) under an argon atmosphere was stirred at RT for 5 mins. Next, a solution of acid chloride (1.18 mmol, 1.50 equiv. prepared using the general procedure) in DCM (3 mL) was added and the resulting mixture was heated, at reflux, at 50 °C for 16 h. The mixture was allowed to cool then diluted with DCM (50 mL), washed with 10% $HCl_{(aq)}$ (15 mL) and $NaHCO_{3(aq)}$ (2 × 20 mL), dried over $MgSO_4$ and concentrated *in vacuo*. Purification by flash column chromatography (SiO_2, 5:1 hexane:ethyl acetate → 2:1 hexane:ethyl acetate) afforded the *title compound* as a yellow oil (193 mg, 77%); R_f 0.74 (ethyl acetate); ν_{max}/cm^{-1} (thin film) 3031, 2929, 2866, 1738, 1689; δ_H (400 MHz, $CDCl_3$) 7.42–7.18 (5H, m, Ph), 4.53 (2H, s, C**H$_2$**Ph), 3.91–3.79 (4H, m, C**H$_2$**NH and C**H$_2$**OBn), 2.94 (2H, t, *J* = 6.4 Hz, C**H$_2$**CH$_2$OBn), 2.84–2.74 (2H, m, C**H$_2$**CO), 1.85–1.75 (2H, m, C**H$_2$**), 1.73–1.65 (2H, m, C**H$_2$**), 1.48–1.17 (10H, m, 5 × C**H$_2$**); δ_C (100 MHz, $CDCl_3$) 179.1 (**C**O), 174.5 (**C**O), 138.2 (**C**), 128.5 (**C**H), 127.8 (**C**H), 127.7 (**C**H), 73.4 (**C**H$_2$Ph), 66.2 (**C**H$_2$OBn), 44.1 (**C**H$_2$NH), 38.1 (**C**H$_2$CO), 36.8 (**C**H$_2$CO), 26.3 (**C**H$_2$), 25.5 (**C**H$_2$), 25.2 (**C**H$_2$), 25.1 (**C**H$_2$), 23.8 (**C**H$_2$), 23.0 (**C**H$_2$), 22.7 (**C**H$_2$); HRMS (ESI): calcd. for $C_{20}H_{29}NNaO_3$, 354.2040. Found: $[MNa]^+$, 354.2040 (−0.1 ppm error).

1-(3-(Benzyloxy)propanoyl)azacyclododecan-2-one (7j)

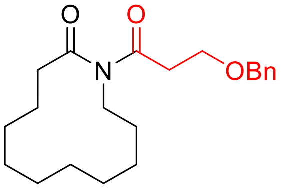

A mixture of azacycloundecan-2-one (133 mg, 0.786 mmol), DMAP (10 mg, 0.0786 mmol) and pyridine (380 µL, 4.72 mmol) in DCM (5.5 mL) under an argon atmosphere was stirred at RT for 5 mins. Next, a solution of acid chloride (1.18 mmol, 1.50 equiv. prepared using the general procedure) in DCM (3 mL) was added and the resulting mixture was heated, at reflux, at 50 °C for 16 h. The mixture was allowed to cool then diluted with DCM (50 mL), washed with 10% $HCl_{(aq)}$ (15 mL) and $NaHCO_{3(aq)}$ (2 × 20 mL), dried over $MgSO_4$ and concentrated *in vacuo*. Purification by flash column chromatography (SiO_2, 5:1 hexane:ethyl acetate → 2:1

hexane:ethyl acetate) afforded the *title compound* (as a mixture of two rotamers determined by ^{13}C NMR resonances) as a colourless oil (209 mg, 77%); R_f 0.78 (ethyl acetate); ν_{max}/cm^{-1} (thin film) 2928, 2863, 1688; δ_H (400 MHz, $CDCl_3$) 7.39–7.18 (10H, m, Ph, both rotamers), 4.52 (4H, s, C**H₂**Ph, both), 3.88–3.72 (8H, m, C**H₂**OBn and C**H₂**NH, both), 2.96–2.88 (4H, m, C**H₂**CH₂OBn, both), 2.83 (4H, m, C**H₂**CO, both), 1.83–1.15 (32H, m, 8 × C**H₂**, both); δ_C (100 MHz, $CDCl_3$) data for the major rotamer only: 178.2 (**C**O), 174.3 (**C**O), 138.2 (**C**), 128.5 (**C**H), 127.8 (**C**H), 127.7 (**C**H), 73.4 (**C**H₂Ph), 66.2 (**C**H₂OBn), 43.6 (**C**H₂NH), 38.1 (**C**H₂CO), 37.3 (**C**H₂CO), 25.7 (**C**H₂), 25.6 (**C**H₂), 24.8 (**C**H₂), 24.5 (**C**H₂), 24.0 (**C**H₂), 23.8 (**C**H₂), 23.4 (**C**H₂), 23.2 (**C**H₂). Diagnostic ^{13}C NMR resonances for the minor rotamer: 179.1 (**C**O), 174.5 (**C**O), 44.1 (**C**H₂NH), 36.8 (**C**H₂CO), 26.3 (**C**H₂), 25.5 (**C**H₂), 25.2 (**C**H₂), 25.1 (**C**H₂), 23.7 (**C**H₂), 23.0 (**C**H₂), 22.7 (**C**H₂); HRMS (ESI): calcd. for $C_{21}H_{31}NNaO_3$, 368.2196. Found: $[MNa]^+$, 368.2195 (0.1 ppm error).

Characterisation data and procedures: Over-Reduced Products

Tetrahydro-5H-oxazolo[3,2-a]pyridin-3(2H)-one (15d)

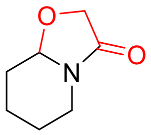

1-(2-(Benzyloxy)acetyl)piperidin-2-one (54 mg, 0.217 mmol) was dissolved in ethyl acetate (3.2 mL) and placed under an argon atmosphere. Palladium on carbon (22 mg, Pd 10% on carbon) was then added and the reaction vessel was backfilled with hydrogen (via balloon) several times, then stirred at RT under a slight positive pressure of hydrogen (balloon) for 16 h. The reaction was then purged with argon, filtered through Celite, washed with methanol. The solvent was then removed *in vacuo* to afford the *title compound* as a colourless oil (25 mg, 82%); R_f 0.30 (ethyl acetate); ν_{max}/cm^{-1} (thin film) 3477, 2947, 2862, 1694; δ_H (400 MHz, $CDCl_3$) 4.98 (1H, d, J = 9.2 Hz, C**H**), 4.23 (2H, s, C**H₂**CO), 4.13 (1H, dd, J = 13.1, 4.1 Hz, C**H**H′N), 2.74 (1H, td, J = 13.1, 3.1 Hz, CH**H′**N), 2.16–2.04 (1H, m, C**H**H′CH), 1.98–1.88 (1H, m, CH**H′**CH), 1.73–1.62 (2H, m, C**H₂**), 1.49–1.30 (2H, m, C**H₂**); δ_C (100 MHz, $CDCl_3$) 168.2 (**C**O), 89.5 (**C**H), 67.7 (**C**H₂CO), 39.6 (**C**H₂N), 32.9 (**C**H₂CH), 24.0 (**C**H₂), 21.4 (**C**H₂); HRMS (ESI): calcd. for $C_7H_{12}NO_2$, 142.0863. Found: $[MH]^+$, 142.0863 (2.3 ppm error).

Hexahydrooxazolo[3,2-a]azepin-3(2H)-one (15e)

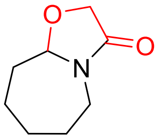

1-(2-(Benzyloxy)acetyl)azepan-2-one (120 mg, 0.459 mmol) was dissolved in ethyl acetate (4.6 mL) and placed under an argon atmosphere. Palladium on carbon (46 mg, Pd 10% on carbon) was then added and the reaction vessel was backfilled with hydrogen (via balloon) several times, then stirred at RT under a slight positive pressure of hydrogen (balloon) for 16 h. The reaction was then purged with argon, filtered through Celite, washed with methanol. The solvent was then removed *in vacuo* to afford the *title compound* as a colourless oil (62 mg, 87%); R_f 0.37 (ethyl acetate); ν_{max}/cm^{-1} (thin film) 2928, 2855, 1698; δ_H (400 MHz, $CDCl_3$) 5.34–5.20 (1H, m, C**H**), 4.21 (1H, d, *J* = 13.7 Hz, C**H**H′O), 4.12 (1H, d, *J* = 13.7 Hz, CH**H′**O), 3.82–3.66 (1H, m, C**H**H′N), 2.99–2.82 (1H, m, CH**H′**N), 2.02–1.31 (8H, m, 4 × C**H$_2$**); δ_C (100 MHz, $CDCl_3$) 170.4 (**C**O), 91.7 (**C**H), 68.0 (**C**H_2O), 40.8 (**C**H_2N), 35.5 (**C**H_2), 29.6 (**C**H_2), 28.1 (**C**H_2), 22.0 (**C**H_2); HRMS (ESI): calcd. for $C_8H_{13}NNaO_2$, 178.0838. Found: $[MNa]^+$, 178.0840 (−1.0 ppm error).

Hexahydro-5H-oxazolo[3,2-a]azocin-3(2H)-one (15f)

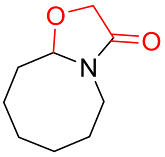

1-(2-(Benzyloxy)acetyl)azocan-2-one (120 mg, 0.436 mmol) was dissolved in ethyl acetate (4.4 mL) and placed under an argon atmosphere. Palladium on carbon (44 mg, Pd 10% on carbon) was then added and the reaction vessel was backfilled with hydrogen (via balloon) several times, then stirred at RT under a slight positive pressure of hydrogen (balloon) for 16 h. The reaction was then purged with argon, filtered through Celite, washed with methanol. The solvent was then removed *in vacuo* to afford the *title compound* as a colourless oil (66 mg, 90%); R_f 0.42 (ethyl acetate); ν_{max}/cm^{-1} (thin film) 2925, 2856, 1699; δ_H (400 MHz, $CDCl_3$) 5.17–5.10 (1H, m, C**H**), 4.26 (1H, dd, *J* = 13.0, 1.5 Hz, C**H**H′O), 4.13 (1H, dd, *J* = 13.0, 1.5 Hz, CH**H′**O), 3.90–3.77 (1H, m, C**H**H′N), 2.81–2.69 (1H, m, CH**H′**N), 1.92–1.75 (3H, m, C**H**H′CH and C**H$_2$**), 1.65–1.35 (7H, m, CH**H′**CH and 3 × C**H$_2$**); δ_C (100 MHz, $CDCl_3$) 170.7 (**C**O), 91.7 (**C**H), 68.0 (**C**H_2O), 39.9 (**C**H_2N), 31.0 (**C**H_2), 27.2 (**C**H_2), 27.0 (**C**H_2), 24.1 (**C**H_2),

20.2 (**C**H_2); HRMS (ESI): calcd. for $C_9H_{15}NNaO_2$, 195.0995. Found: $[MNa]^+$, 195.0999 (−2.6 ppm error).

Octahydrooxazolo[3,2-a]azonin-3(2H)-one (15g)

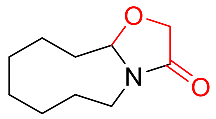

1-(2-(Benzyloxy)acetyl)azonan-2-one (64 mg, 0.219 mmol) was dissolved in ethyl acetate (3.2 mL) and placed under an argon atmosphere. Palladium on carbon (22 mg, Pd 10% on carbon) was then added and the reaction vessel was backfilled with hydrogen (via balloon) several times, then stirred at RT under a slight positive pressure of hydrogen (balloon) for 16 h. The reaction was then purged with argon, filtered through Celite, washed with methanol and the solvent was removed *in vacuo*. Purification by flash column chromatography (SiO_2, 2:1 hexane:ethyl acetate → ethyl acetate) afforded the *title compound* as a colourless oil (32 mg, 82%); R_f 0.49 (ethyl acetate); ν_{max}/cm^{-1} (thin film) 3424, 2924, 2857, 1694; δ_H (400 MHz, $CDCl_3$) 5.18 (1H, br s, C**H**), 4.34 (1H, d, J = 13.7 Hz, C**H**H′CO), 4.18 (1H, d, J = 13.7 Hz, CH**H′**CO), 3.86–3.73 (1H, m, C**H**H′N), 2.87–2.74 (1H, m, CH**H′**N), 2.01–1.31 (12H, m, 6 × C**H_2**); δ_C (100 MHz, $CDCl_3$) 171.7 (**C**O), 92.7 (**C**H), 68.2 (**C**H_2CO), 41.3 (**C**H_2N), 30.7 (**C**H_2), 25.9 (**C**H_2), 25.5 (**C**H_2), 25.3 (**C**H_2), 24.3 (**C**H_2), 19.8 (**C**H_2); HRMS (ESI): calcd. for $C_{10}H_{18}NO_2$, 184.1332. Found: $[MH]^+$, 184.1331 (0.7 ppm error).

Octahydro-5H-oxazolo[3,2-a]azecin-3(2H)-one (15h)

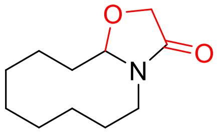

1-(2-(Benzyloxy)acetyl)azecan-2-one (27 mg, 0.090 mmol) was dissolved in ethyl acetate (0.9 mL) and placed under an argon atmosphere. Palladium on carbon (9 mg, Pd 10% on carbon) was then added and the reaction vessel was backfilled with hydrogen (via balloon) several times, then stirred at RT under a slight positive pressure of hydrogen (balloon) for 16 h. The reaction was then purged with argon, filtered through Celite, and washed with methanol. The solvent was then removed *in vacuo* to afford the *title compound* as a colourless oil (13 mg, 76%); R_f 0.62 (ethyl acetate); ν_{max}/cm^{-1} (thin film) 2919, 2864, 1701; δ_H (400 MHz, $CDCl_3$) 5.25 (1H, br s, C**H**), 4.32 (1H, dd, J = 13.7, 2.1 Hz, C**H**H′CO), 4.18 (1H, d, J = 13.7 Hz, CH**H′**CO), 3.92–3.79 (1H, m, C**H**H′N), 2.99–2.87 (1H, m, CH**H′**N), 1.98–1.34 (14H, m, 7 ×

C**H₂**); δ_C (100 MHz, $CDCl_3$) 172.5 (**C**O), 93.5 (**C**H), 68.1 (**C**H_2CO), 40.8 (**C**H_2N), 30.2 (**C**H_2), 26.3 (**C**H_2), 25.5 (**C**H_2), 23.5 (**C**H_2), 22.1 (**C**H_2), 21.6 (**C**H_2), 18.7 (**C**H_2); HRMS (ESI): calcd. for $C_{11}H_{20}NO_2$, 198.1489. Found: $[MH]^+$, 198.1494 (−2.9 ppm error).

10a-Methoxyhexahydro-5H-oxazolo[3,2-a]azocin-3(2H)-one (14f)

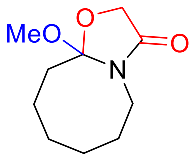

1-(2-(Benzyloxy)acetyl)azocan-2-one (120 mg, 0.436 mmol) was dissolved in methanol (4.4 mL) and placed under an argon atmosphere. Palladium on carbon (44 mg, Pd 10% on carbon) was then added and the reaction vessel was backfilled with hydrogen (via balloon) several times, then stirred at RT under a slight positive pressure of hydrogen (balloon) for 1 h. The reaction was then purged with argon, filtered through Celite, washed with methanol where the solvent was removed *in vacuo* to afford the *title compound* as a colourless oil (82 mg, 95%); R_f 0.52 (ethyl acetate); ν_{max}/cm^{-1} (thin film) 2928, 2859, 1712; δ_H (400 MHz, $CDCl_3$) 4.29 (2H, s, C**H₂**O), 3.74 (1H, dt, *J* = 13.7, 5.0 Hz, C**H**H′N), 3.03 (3H, s, OC**H₃**), 2.81 (1H, ddd, *J* = 13.7, 9.2, 4.5 Hz, CH**H′**N), 2.05–1.85 (2H, m, C**H₂**C), 1.63–1.30 (8H, m, 4 × C**H₂**); δ_C (100 MHz, $CDCl_3$) 170.5 (**C**O), 115.1 (**C**OMe), 68.5 (**C**H_2O), 48.2 (**C**H_3O), 39.0 (**C**H_2N), 34.5 (**C**H_2C), 27.0 (**C**H_2), 26.2 (**C**H_2), 23.8 (**C**H_2), 21.5 (**C**H_2); HRMS (ESI): calcd. for $C_{10}H_{17}NNaO_3$, 222.1101. Found: $[MNa]^+$, 222.1105 (−1.3 ppm error).

Characterisation data and procedures: Ring Expansion Reactions

1-Oxa-4-azacyclohexadecane (12a)

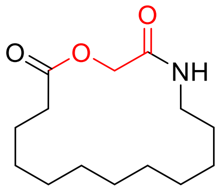

A mixture of laurolactam (155 mg, 0.786 mmol), DMAP (10 mg, 0.0786 mmol) and pyridine (380 μL, 4.72 mmol) in DCM (5.5 mL) under an argon atmosphere was stirred at RT for 5 mins. Next, a solution of acid chloride (1.18 mmol, 1.50 equiv. prepared using the general procedure) in DCM (3 mL) was added and the resulting mixture was heated, at reflux, at 50 °C for 16 h. The solvent was concentrated *in vacuo*, loaded onto a short silica plug and eluted with 2:1 hexane:ethyl acetate, to remove the majority of excess carboxylic acid and pyridine

residues, and concentrated *in vacuo*. This material (**4a**) was re-dissolved in THF (7.8 mL) and placed under an argon atmosphere. Palladium on carbon (78 mg, Pd 10% on carbon) and water (1.42 mL, 78.6 mmol) was then added and the reaction vessel was backfilled with hydrogen (via balloon) several times, then stirred at RT under a slight positive pressure of hydrogen (balloon) for 1 h. The reaction was then purged with argon, filtered through Celite, washed with methanol where the solvent was removed *in vacuo*. The crude material was then re-dissolved in chloroform (7.8 mL) and triethylamine (165 µL, 1.18 mmol) added, and stirred at RT for 16 h, then reduced *in vacuo*. Purification by flash column chromatography (SiO_2, 2:1 hexane:ethyl acetate → ethyl acetate) afforded the *title compound* as a white solid (177 mg, 88%). Data consistent with those previously reported in the literature.[1]

1-(2-Hydroxyacetyl)azetidin-2-one (8b)

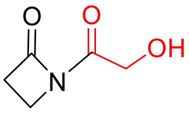

1-(3-(Benzyloxy)propanoyl)azetidin-2-one (147 mg, 0.671 mmol) was dissolved in ethyl acetate (6.7 mL) and placed under an argon atmosphere. Palladium on carbon (67 mg, Pd 10% on carbon) was then added and the reaction vessel was backfilled with hydrogen (via balloon) several times, then stirred at RT under a slight positive pressure of hydrogen (balloon) for 2 h. The reaction was then purged with argon, filtered through Celite, washed with methanol where the solvent was removed *in vacuo*. The crude material was then re-dissolved in chloroform (6.7 mL) and triethylamine (140 µL, 1.01 mmol) added, and stirred at RT for 16 h, then reduced *in vacuo*. Purification by flash column chromatography (SiO_2, 2:1 hexane:ethyl acetate → ethyl acetate → 9:1 ethyl acetate:methanol) afforded the *title compound* as a colourless oil (86 mg, 99%); R_f 0.26 (ethyl acetate); ν_{max}/cm^{-1} (thin film) 3425, 2913, 1778, 1694; δ_H (400 MHz, $CDCl_3$) 4.44 (2H, s, C**H**$_2$OH), 3.62 (2H, t, *J* = 5.3 Hz, C**H**$_2$N), 3.22 (1H, s, O**H**), 3.08 (2H, t, *J* = 5.3 Hz, C**H**$_2$CON); δ_C (100 MHz, $CDCl_3$) 170.9 (**C**O), 164.9 (**C**O), 63.4 (**C**H$_2$OH), 36.5 (**C**H$_2$N), 36.3 (**C**H$_2$CON); HRMS (ESI): calcd. for $C_5H_8NO_3$, 130.0499. Found: $[MH]^+$, 130.0498 (0.2 ppm error).

1-(2-Hydroxyacetyl)pyrrolidin-2-one (8c)

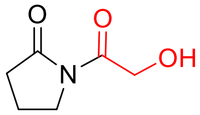

1-(2-(Benzyloxy)acetyl)pyrrolidin-2-one (135 mg, 0.579 mmol) was dissolved in ethyl acetate (5.8 mL) and placed under an argon atmosphere. Palladium on carbon (58 mg, Pd 10% on carbon) was then added and the reaction vessel was backfilled with hydrogen (via balloon) several times, then stirred at RT under a slight positive pressure of hydrogen (balloon) for 2 h. The reaction was then purged with argon, filtered through Celite, washed with methanol where the solvent was removed *in vacuo*. The crude material was then re-dissolved in chloroform (5.8 mL) and triethylamine (121 µL, 0.869 mmol) added, and stirred at RT for 16 h, then reduced *in vacuo*. Purification by flash column chromatography (SiO_2, 2:1 hexane:ethyl acetate → ethyl acetate) afforded the *title compound* as a white oil (83 mg, 100%); R_f 0.31 (ethyl acetate); ν_{max}/cm^{-1} (thin film) 3456, 2905, 1732, 1686; δ_H (400 MHz, $CDCl_3$) 4.56 (2H, s, C**H**$_2$OH), 3.77 (2H, t, *J* = 7.3 Hz, C**H**$_2$N), 3.30 (1H, br s, O**H**), 2.53 (2H, t, *J* = 8.0 Hz, C**H**$_2$CON), 2.12–2.01 (2H, m, C**H**$_2$CH$_2$N); δ_C (100 MHz, $CDCl_3$) 176.0 (**C**O), 174.4 (**C**O), 64.2 (**C**H$_2$OH), 45.0 (**C**H$_2$N), 32.9 (**C**H$_2$CO), 18.0 (**C**H$_2$); HRMS (ESI): calcd. for $C_6H_9NNaO_3$, 166.0475. Found: $[MNa]^+$, 166.0477 (−0.9 ppm error).

8a-Hydroxytetrahydro-5H-oxazolo[3,2-a]pyridin-3(2H)-one and 1-(2-hydroxyacetyl)piperidin-2-one (10d and 8d)

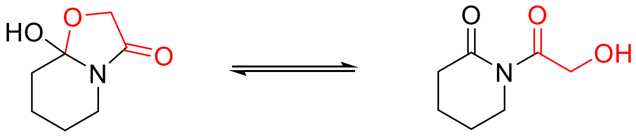

1-(2-(Benzyloxy)acetyl)piperidin-2-one (188 mg, 0.768 mmol) was dissolved in THF (7.7 mL) and placed under an argon atmosphere. Palladium on carbon (77 mg, Pd 10% on carbon) and water (1.38 mL, 76.8 mmol) was then added and the reaction vessel was backfilled with hydrogen (via balloon) several times, then stirred at RT under a slight positive pressure of hydrogen (balloon) for 2 h. The reaction was then purged with argon, filtered through Celite, washed with methanol where the solvent was removed *in vacuo*. The crude material was then re-dissolved in chloroform (7.7 mL) and triethylamine (161 µL, 1.15 mmol) added, and stirred at RT for 16 h, then reduced *in vacuo*. Purification by flash column chromatography (SiO_2, 2:1 hexane:ethyl acetate → ethyl acetate) afforded an inseparable mixture of the *title compounds* (as a 10:6.8 mixture, **10d**:**8d**) as a colourless oil (109 mg, 90%); R_f 0.39 (ethyl acetate);

ν_{max}/cm^{-1} (thin film) 3375, 2947, 2874, 1688. Data for *8a-hydroxytetrahydro-5H-oxazolo[3,2-a]pyridin-3(2H)-one*: δ_H (400 MHz, $CDCl_3$) 4.91 (1H, br s, O**H**), 4.37 (1H, d, J = 13.9 Hz, C**H**H′O), 4.17 (1H, d, J = 13.9 Hz, CH**H′**O), 3.97 (1H, dd, J = 13.3, 5.0 Hz, C**H**H′N), 3.02–2.90 (1H, m, CH**H′**N), 2.19–2.11 (1H, m, C**H**H′COH), 1.91–1.26 (5H, m, CH**H′**COH and 2 × C**HH′**); δ_C (100 MHz, $CDCl_3$) 168.1 (**C**O), 109.2 (**C**OH), 66.8 (**C**H_2O), 38.4 (**C**H_2N), 36.2 (**C**H_2COH), 24.3 (**C**H_2), 21.3 (**C**H_2). Data for *1-(2-hydroxyacetyl)piperidin-2-one*: 4.61 (1H, s, C**H₂**OH), 3.82–3.72 (2H, m, C**H₂**N), 3.44 (1H, br s, O**H**), 2.60–2.52 (2H, m, C**H₂**CO), 1.94–1.26 (4H, m, 2 × C**H₂**); δ_C (100 MHz, $CDCl_3$) 177.5 (**C**O), 173.1 (**C**O), 66.1 (**C**H_2OH), 44.6 (**C**H_2N), 34.5 (**C**H_2CO), 22.2 (**C**H_2), 20.0 (**C**H_2); HRMS (ESI): calcd. for $C_7H_{11}NNaO_3$, 180.0631. Found: $[MNa]^+$, 180.0634 (−1.6 ppm error).

9a-Hydroxyhexahydrooxazolo[3,2-a]azepin-3(2H)-one and 1-(2-hydroxyacetyl)azepan-2-one and 1,4-oxazecane-3,10-dione (10e and 8e and 12e)

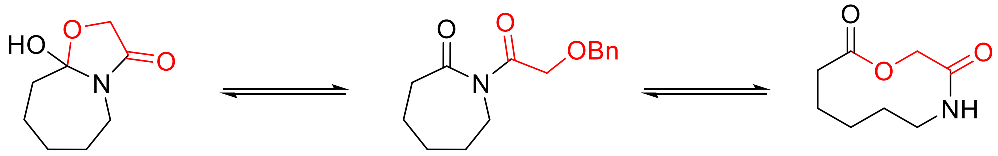

1-(2-(Benzyloxy)acetyl)azepan-2-one (203 mg, 0.776 mmol) was dissolved in THF (7.7 mL) and placed under an argon atmosphere. Palladium on carbon (77 mg, Pd 10% on carbon) and water (1.40 mL, 77.6 mmol) was then added and the reaction vessel was backfilled with hydrogen (via balloon) several times, then stirred at RT under a slight positive pressure of hydrogen (balloon) for 2 h. The reaction was purged with argon, filtered through Celite, washed with methanol where the solvent was removed *in vacuo*. The crude material was re-dissolved in chloroform (7.7 mL) and triethylamine (162 μL, 1.16 mmol) added, and stirred at RT for 16 h, then reduced *in vacuo*. Purification by flash column chromatography (SiO_2, 2:1 hexane:ethyl acetate → ethyl acetate) afforded an inseparable mixture of the *title compounds* (as a 69:13:1 mixture, **10e**:**8e**:**12e**) as a colourless oil (128 mg, 96%); R_f 0.42 (ethyl acetate); ν_{max}/cm^{-1} (thin film) 3331, 2932, 2857, 1688; δ_H (400 MHz, $CDCl_3$) data for the 9a-hydroxyhexahydrooxazolo[3,2-a]azepin-3(2H)-one: 5.91 (1H, s, O**H**), 4.29 (1H, d, J = 13.7 Hz, C**H**H′O), 4.11 (1H, d, J = 13.7 Hz, CH**H′**O), 3.78–3.69 (1H, m, C**H**H′COH), 2.94–2.83 (1H, m, CH**H′**COH), 2.28–2.18 (1H, m, C**H**H′N), 1.90–1.80 (1H, m, CH**H′**N), [1.80–1.55 (3H, m) 1.54–1.39 (1H, m), 1.37–1.23 (1H, m), 1.22–1.09 (1H, m), 3 × C**HH′**]. Diagnostic ^{1}H NMR resonances for 1-(2-hydroxyacetyl)azepan-2-one: 4.52 (2H, d, J = 4.6 Hz, C**H₂**OH), 3.93–3.86 (2H, m, C**H₂**N), 2.79 (1H, br s, O**H**), 2.69–2.59 (2H, m, C**H₂**CO). Diagnostic ^{1}H NMR resonances for 1,4-oxazecane-3,10-dione: 6.16 (1H, br s, N**H**), 3.41–3.32 (2H, m, C**H₂**NH); δ_C

(100 MHz, $CDCl_3$) data for the 9a-hydroxyhexahydrooxazolo[3,2-a]azepin-3(2H)-one only: 170.5 (**C**O), 113.9 (**C**OH), 66.5 (**C**H_2O), [40.2, 39.9 (**C**H_2COH and **C**H_2N)], 29.6 (**C**H_2), 27.9 (**C**H_2), 22.7 (**C**H_2). ^{13}C NMR resonances for 1-(2-hydroxyacetyl)azepan-2-one: 177.7 (**C**O), 176.7 (**C**O), 65.8 (**C**H_2OH), 43.8 (**C**H_2N), 39.3 (**C**H_2CO), 29.1 (**C**H_2), 28.3 (**C**H_2), 23.6 (**C**H_2). Diagnostic ^{13}C NMR resonances for 1,4-oxazecane-3,10-dione: 178.0 (**C**O), 172.5 (**C**O), 68.0 (**C**H_2O), 43.4 (**C**H_2NH), 35.0 (**C**H_2CO), 27.2 (**C**H_2), 22.5 (**C**H_2); HRMS (ESI): calcd. for $C_8H_{13}NNaO_3$, 194.0788. Found: $[MNa]^+$, 194.0788 (−0.9 ppm error).

1-Oxa-4-azacycloundecane-3,11-dione (12f)

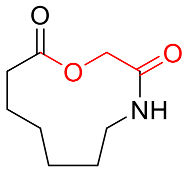

1-(2-(Benzyloxy)acetyl)azocan-2-one (207 mg, 0.751 mmol) was dissolved in ethyl acetate (7.5 mL) and placed under an argon atmosphere. Palladium on carbon (75 mg, Pd 10% on carbon) was then added and the reaction vessel was backfilled with hydrogen (via balloon) several times, then stirred at RT under a slight positive pressure of hydrogen (balloon) for 1 h. The reaction was then purged with argon, filtered through Celite, washed with methanol where the solvent was removed *in vacuo*. The crude material was then re-dissolved in chloroform (7.5 mL) and triethylamine (157 µL, 1.13 mmol) was added, and then stirred at RT for 16 h, and reduced *in vacuo*. Purification by flash column chromatography (SiO_2, 2:1 hexane:ethyl acetate → ethyl acetate → 9:1 ethyl acetate:methanol) afforded the *title compound* (as a 9:1 mixture of rotamers) as a white solid (123 mg, 89%); mp. 98–99 °C; R_f 0.35 (ethyl acetate); ν_{max}/cm^{-1} (thin film) 3287, 2928, 2889, 2868, 2842, 1731, 1659; δ_H (400 MHz, $CDCl_3$) 6.06 (1H, s, N**H**, minor rotamer), 5.94 (1H, s, N**H**, major rotamer), 4.58 (2H, s, C**H₂**O, minor), 4.53 (2H, s, C**H₂**O, major), 3.26–3.12 (4H, m, C**H₂**NH, both rotamers), 2.43–2.29 (4H, m, C**H₂**COO, both), 1.77–1.64 (4H, m, C**H₂**, both), 1.60–1.50 (4H, m, C**H₂**, both), 1.46–1.37 (4H, m, C**H₂**, both), 1.36–1.25 (4H, m, C**H₂**, both); δ_C (100 MHz, $CDCl_3$) data for the major rotamer only: 173.5 (**C**O), 168.3 (**C**O), 64.2 (**C**H_2O), 39.4 (**C**H_2NH), 34.7 (**C**H_2COO), 25.8 (**C**H_2), 25.2 (**C**H_2), 25.1 (**C**H_2), 23.1 (**C**H_2). Diagnostic ^{13}C NMR resonances for the minor rotamer: 172.7 (**C**O), 169.4 (**C**O), 63.9 (**C**H_2O), 40.4 (**C**H_2NH), 28.9 (**C**H_2); HRMS (ESI): calcd. for $C_9H_{16}NO_3$, 186.1125. Found: $[MH]^+$, 186.1129 (−0.4 ppm error).

1-Oxa-4-azacyclododecane-3,12-dione (12g)

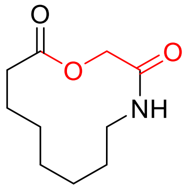

1-(2-(Benzyloxy)acetyl)azonan-2-one (209 mg, 0.729 mmol) was dissolved in THF (7.2 mL) and placed under an argon atmosphere. Palladium on carbon (72 mg, Pd 10% on carbon) and water (1.3 mL, 72.9 mmol) was then added and the reaction vessel was backfilled with hydrogen (via balloon) several times, then stirred at RT under a slight positive pressure of hydrogen (balloon) for 2 h. The reaction was then purged with argon, filtered through Celite, washed with methanol where the solvent was removed *in vacuo*. The crude material was then re-dissolved in chloroform (7.2 mL) and triethylamine (151 μL, 1.09 mmol) added, and stirred at RT for 16 h, then reduced *in vacuo*. Purification by flash column chromatography (SiO_2, 2:1 hexane:ethyl acetate → ethyl acetate → 9:1 ethyl acetate:methanol) afforded the *title compound* as a white solid (139 mg, 97%); mp. 78–79 °C; R_f 0.43 (ethyl acetate); ν_{max}/cm^{-1} (thin film) 3274, 2935, 1733, 1660, 1551; δ_H (400 MHz, $CDCl_3$) 6.26 (1H, s, N**H**), 4.60 (2H, s, C**H₂**O), 3.34–3.23 (2H, m, C**H₂**NH), 2.49–2.38 (2H, m, C**H₂**COO), 1.82–1.70 (2H, m, C**H₂**), 1.55–1.29 (8H, m, 4 × C**H₂**); δ_C (100 MHz, $CDCl_3$) 172.7 (**C**O), 167.2 (**C**O), 62.8 (**C**H_2O), 39.6 (**C**H_2NH), 33.9 (**C**H_2CO), 27.2 (**C**H_2), 25.9 (**C**H_2), 25.3 (**C**H_2), 24.7 (**C**H_2), 24.3 (**C**H_2); HRMS (ESI): calcd. for $C_{10}H_{17}NNaO_3$, 222.1101. Found: $[MNa]^+$, 222.1102 (−1.2 ppm error).

1-Oxa-4-azacyclotridecane-3,13-dione (12h)

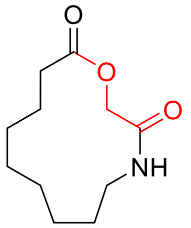

1-(2-(Benzyloxy)acetyl)azecan-2-one (207 mg, 0.681 mmol) was dissolved in THF (6.8 mL) and placed under an argon atmosphere. Palladium on carbon (68 mg, Pd 10% on carbon) and water (1.2 mL, 68.1 mmol) was then added and the reaction vessel was backfilled with hydrogen (via balloon) several times, then stirred at RT under a slight positive pressure of hydrogen (balloon) for 2 h. The reaction was then purged with argon, filtered through Celite, washed with methanol where the solvent was removed *in vacuo*. The crude material was then re-dissolved in chloroform (6.8 mL) and triethylamine (142 μL, 1.02 mmol) added, and stirred

at RT for 16 h, then reduced *in vacuo*. Purification by flash column chromatography (SiO_2, 2:1 hexane:ethyl acetate → ethyl acetate → 9:1 ethyl acetate:methanol) afforded the *title compound* (as a 5:1 mixture of rotamers) as a white solid (135 mg, 93%); mp. 82.0–83.0 °C; R_f 0.48 (ethyl acetate); ν_{max}/cm^{-1} (thin film) 3384, 3282, 2919, 2859, 1720, 1662, 1541; δ_H (400 MHz, $CDCl_3$) 6.01 (1H, br s, N**H**, major rotamer), 4.63 (2H, s, C**H₂**O, major), 4.14 (2H, s, C**H₂**O, minor rotamer), 3.73 (1H, br s, N**H**, minor), 3.53–3.46 (2H, m, C**H₂**NH, minor), 3.33–3.24 (2H, m, C**H₂**NH, major), 3.22–3.16 (2H, m, C**H₂**CO, minor), 2.50–2.39 (2H, m, C**H₂**CO, major), 1.85–1.68 (4H, m, C**H₂**, both rotamers), 1.62–1.28 (20H, m, 5 × C**H₂**, both); δ_C (100 MHz, $CDCl_3$) 172.4 (**C**O), 167.6 (**C**O), 62.8 (**C**H₂O), 38.9 (**C**H₂NH), 34.0 (**C**H₂CO), 26.3 (**C**H₂), 26.0 (**C**H₂), 25.7 (**C**H₂), 25.5 (**C**H₂), 24.0 (**C**H₂), 23.3 (**C**H₂). Diagnostic ^{13}C NMR resonances for minor rotamer: 173.0 (**C**O), 60.5 (**C**H₂O), 48.3 (**C**H₂NH), 25.4 (**C**H₂), 25.2 (**C**H₂), 24.2 (**C**H₂), 23.6 (**C**H₂), 23.0 (**C**H₂); HRMS (ESI): calcd. for $C_{11}H_{19}NNaO_3$, 236.1257. Found: $[MNa]^+$, 236.1259 (−0.6 ppm error).

1-Oxa-4-azacyclotetradecane-3,14-dione (12i)

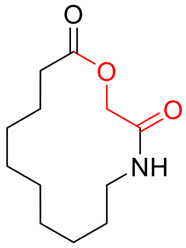

A mixture of azacycloundecan-2-one (133 mg, 0.786 mmol), DMAP (10 mg, 0.0786 mmol) and pyridine (380 µL, 4.72 mmol) in DCM (5.5 mL) under an argon atmosphere was stirred at RT for 5 mins. Next, a solution of acid chloride (1.18 mmol, 1.50 equiv. prepared using the general procedure) in DCM (3 mL) was added and the resulting mixture was heated, at reflux, at 50 °C for 16 h. The solvent was concentrated *in vacuo*, loaded onto a short silica plug and eluted with 2:1 hexane:ethyl acetate, to remove the majority of excess carboxylic acid and pyridine residues, and concentrated *in vacuo*. This material was re-dissolved in THF (7.8 mL) and placed under an argon atmosphere. Palladium on carbon (78 mg, Pd 10% on carbon) and water (1.42 mL, 78.6 mmol) was then added and the reaction vessel was backfilled with hydrogen (via balloon) several times, then stirred at RT under a slight positive pressure of hydrogen (balloon) for 1 h. The reaction was then purged with argon, filtered through Celite, washed with methanol where the solvent was removed *in vacuo*. The crude material was then re-dissolved in chloroform (7.8 mL) and triethylamine (165 µL, 1.18 mmol) added, and stirred at RT for 16 h, then reduced *in vacuo*. Purification by flash column chromatography (SiO_2, 2:1

hexane:ethyl acetate → ethyl acetate) afforded the *title compound* as a white solid (154 mg, 86%); mp. 97.0–98.0 °C; R_f 0.52 (ethyl acetate); ν_{max}/cm^{-1} (thin film) 3397, 2921, 2861, 1722, 1678, 1663, 1534; δ_H (400 MHz, $CDCl_3$) 6.13 (1H, br s, N**H**), 4.58 (2H, s, C**H$_2$**O), 3.40–3.29 (2H, m, C**H$_2$**NH), 2.50–2.38 (2H, m, C**H$_2$**COO), 1.77–1.64 (2H, m, C**H$_2$**), 1.58–1.48 (2H, m, C**H$_2$**), 1.44–1.21 (10H, m, 5 × C**H$_2$**); δ_C (100 MHz, $CDCl_3$) 172.4 (**C**O), 167.2 (**C**O), 63.1 (**C**H$_2$O), 38.2 (**C**H$_2$NH), 33.4 (**C**H$_2$CO), 26.6 (**C**H$_2$), 26.2 (**C**H$_2$), 25.8 (**C**H$_2$), 25.7 (**C**H$_2$), 25.2 (**C**H$_2$), 24.2 (**C**H$_2$), 23.9 (**C**H$_2$); HRMS (ESI): calcd. for $C_{12}H_{21}NNaO_3$, 250.1414. Found: $[MNa]^+$, 250.1416 (−1.3 ppm error).

1-Oxa-4-azacyclopentadecane-3,15-dione (12j)

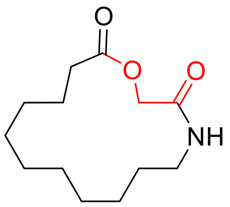

A mixture of azacyclododecan-2-one (144 mg, 0.786 mmol), DMAP (10 mg, 0.0786 mmol) and pyridine (380 μL, 4.72 mmol) in DCM (5.5 mL) under an argon atmosphere was stirred at RT for 5 mins. Next, a solution of acid chloride (1.18 mmol, 1.50 equiv. prepared using the general procedure) in DCM (3 mL) was added and the resulting mixture was heated, at reflux, at 50 °C for 16 h. The solvent was concentrated *in vacuo*, loaded onto a short silica plug and eluted with 2:1 hexane:ethyl acetate, to remove the majority of excess carboxylic acid and pyridine residues, and concentrated *in vacuo*. This material was re-dissolved in THF (7.8 mL) and placed under an argon atmosphere. Palladium on carbon (78 mg, Pd 10% on carbon) and water (1.42 mL, 78.6 mmol) was then added and the reaction vessel was backfilled with hydrogen (via balloon) several times, then stirred at RT under a slight positive pressure of hydrogen (balloon) for 1 h. The reaction was then purged with argon, filtered through Celite, washed with methanol where the solvent was removed *in vacuo*. The crude material was then re-dissolved in chloroform (7.8 mL) and triethylamine (165 μL, 1.18 mmol) added, and stirred at RT for 16 h, then reduced *in vacuo*. Purification by flash column chromatography (SiO_2, 2:1 hexane:ethyl acetate → ethyl acetate) afforded the *title compound* (as a 3:1 mixture of rotamers) as a white solid (155 mg, 83%); mp. 82.0–83.0 °C; R_f 0.54 (ethyl acetate); ν_{max}/cm^{-1} (thin film) 3398, 2927, 2857, 1725, 1677, 1530; δ_H (400 MHz, $CDCl_3$) 6.16 (2H, br s, N**H**, both rotamers), 4.58 (2H, s, C**H$_2$**O, minor rotamer), 4.55 (2H, s, C**H$_2$**O, major rotamer), 3.40–3.27 (4H, m, C**H$_2$**NH, both), 2.48–2.37 (4H, m, C**H$_2$**COO, both), 1.79–1.65 (4H, m, C**H$_2$**, both), 1.59–1.48 (4H, m, C**H$_2$**, both), 1.42–1.19 (24H, m, 6 × C**H$_2$**, both); δ_C (100 MHz, $CDCl_3$) data

for the major rotamer only: 172.2 (**C**O), 167.3 (**C**O), 63.0 (**C**H_2O), 38.5 (**C**H_2NH), 33.3 (**C**H_2CO), 29.9 (**C**H_2), 27.3 (**C**H_2), 26.7 (**C**H_2), 26.5 (**C**H_2), 26.4 (**C**H_2), 26.3 (**C**H_2), 24.8 (**C**H_2), 24.3 (**C**H_2). Diagnostic ^{13}C NMR resonances for the minor rotamer: 172.4 (**C**O), 167.2 (**C**O), 63.1 (**C**H_2O), 38.2 (**C**H_2NH), 33.4 (**C**H_2CO), 26.6 (**C**H_2), 26.2 (**C**H_2), 25.8 (**C**H_2), 25.7 (**C**H_2), 25.2 (**C**H_2), 24.2 (**C**H_2), 23.9 (**C**H_2); HRMS (ESI): calcd. for $C_{13}H_{23}NNaO_3$, 264.1570. Found: $[MNa]^+$, 264.1574 (−1.1 ppm error).

1-Oxa-5-azacycloheptadecane-4,17-dione (13a)

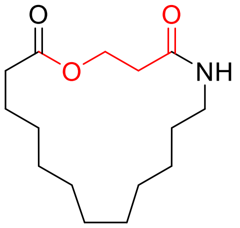

1-(3-(Benzyloxy)propanoyl)azacyclotridecan-2-one (170 mg, 0.473 mmol) was dissolved in ethyl acetate (4.7 mL) and placed under an argon atmosphere. Palladium on carbon (47 mg, Pd 10% on carbon) was then added and the reaction vessel was backfilled with hydrogen (via balloon) several times, then stirred at RT under a slight positive pressure of hydrogen (balloon) for 2 h. The reaction was then purged with argon, filtered through Celite, washed with methanol where the solvent was removed *in vacuo*. The crude material was then re-dissolved in chloroform (4.7 mL) and triethylamine (98 µL, 0.709 mmol) added, and stirred at RT for 16 h, then reduced *in vacuo*. Purification by flash column chromatography (SiO_2, 2:1 hexane:ethyl acetate → ethyl acetate) afforded the *title compound* as a colourless oil (121 mg, 95%). Data consistent with those previously reported in the literature.[1]

1-(3-Hydroxypropanoyl)azetidin-2-one (9b)

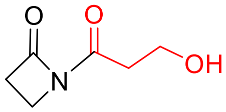

1-(3-(Benzyloxy)propanoyl)azetidin-2-one (153 mg, 0.657 mmol) was dissolved in ethyl acetate (6.6 mL) and placed under an argon atmosphere. Palladium on carbon (66 mg, Pd 10% on carbon) was then added and the reaction vessel was backfilled with hydrogen (via balloon) several times, then stirred at RT under a slight positive pressure of hydrogen (balloon) for 2 h. The reaction was then purged with argon, filtered through Celite, washed with methanol where the solvent was removed *in vacuo*. The crude material was then re-dissolved in chloroform (6.6 mL) and triethylamine (137 µL, 0.986 mmol) added, and stirred at RT for 16 h, then reduced

in vacuo. Purification by flash column chromatography (SiO_2, 2:1 hexane:ethyl acetate → ethyl acetate) afforded the *title compound* as a colourless oil (86 mg, 92%); R_f 0.28 (ethyl acetate); ν_{max}/cm^{-1} (thin film) 3407, 2913, 1777, 1682; δ_H (400 MHz, $CDCl_3$) 3.90 (2H, t, *J* = 5.3 Hz, C**H**$_2$OH), 3.59 (2H, t, *J* = 5.3 Hz, C**H**$_2$N), 3.05 (2H, t, *J* = 5.3 Hz, C**H**$_2$CH$_2$N), 2.92 (2H, t, *J* = 5.3 Hz, C**H**$_2$CH$_2$OH), 2.61 (1H, br s, O**H**); δ_C (100 MHz, $CDCl_3$) 170.4 (**C**O), 165.3 (**C**O), 57.8 (**C**H$_2$OH), 39.1 (**C**H$_2$N), 36.7 (**C**H$_2$CO), 36.2 (**C**H$_2$CO); HRMS (ESI): calcd. for $C_6H_9NNaO_3$, 166.0475. Found: $[MNa]^+$, 166.0476 (−0.6 ppm error).

1-(3-Hydroxypropanoyl)pyrrolidin-2-one (9e)

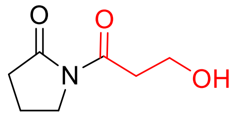

1-(3-(Benzyloxy)propanoyl)pyrrolidin-2-one (95 mg, 0.383 mmol) was dissolved in ethyl acetate (3.8 mL) and placed under an argon atmosphere. Palladium on carbon (38 mg, Pd 10% on carbon) was then added and the reaction vessel was backfilled with hydrogen (via balloon) several times, then stirred at RT under a slight positive pressure of hydrogen (balloon) for 1 h. The reaction was then purged with argon, filtered through Celite, washed with methanol where the solvent was removed *in vacuo*. The crude material was then re-dissolved in chloroform (3.8 mL) and triethylamine (80 µL, 0.575 mmol) added, and stirred at RT for 16 h, then reduced *in vacuo*. Purification by flash column chromatography (SiO_2, 2:1 hexane:ethyl acetate → ethyl acetate) afforded the *title compound* as a colourless oil (58 mg, 96%); R_f 0.30 (ethyl acetate); ν_{max}/cm^{-1} (thin film) 3406, 2896, 1732, 1682; δ_H (400 MHz, $CDCl_3$) 3.87 (2H, t, *J* = 5.3 Hz, C**H**$_2$OH), 3.78 (2H, t, *J* = 7.3 Hz, C**H**$_2$N), 3.10 (2H, t, *J* = 5.3 Hz, C**H**$_2$CH$_2$OH), 2.74 (1H, br s, O**H**), 2.56 (2H, t, *J* = 7.3 Hz, C**H**$_2$CON), 2.07–1.96 (2H, m, C**H**$_2$CH$_2$N); δ_C (100 MHz, $CDCl_3$) 175.8 (**C**O), 173.8 (**C**O), 58.0 (**C**H$_2$OH), 45.3 (**C**H$_2$N), 39.6 (**C**H$_2$CH$_2$OH), 33.6 (**C**H$_2$CON), 17.3 (**C**H$_2$); HRMS (ESI): calcd. for $C_7H_{11}NNaO_3$, 180.0631. Found: $[MNa]^+$, 180.0632 (−1.5 ppm error).

1,5-Oxazecane-4,10-dione (13d)

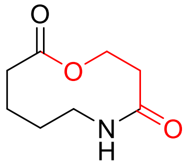

1-(3-(Benzyloxy)propanoyl)piperidin-2-one (80 mg, 0.318 mmol) was dissolved in ethyl acetate (3.2 mL) and placed under an argon atmosphere. Palladium on carbon (32 mg, Pd 10% on carbon) was then added and the reaction vessel was backfilled with hydrogen (via balloon)

several times, then stirred at RT under a slight positive pressure of hydrogen (balloon) for 1 h. The reaction was then purged with argon, filtered through Celite, washed with methanol where the solvent was removed *in vacuo*. The crude material was then re-dissolved in chloroform (3.2 mL) and triethylamine (66 uL, 0.468 mmol) added, and stirred at RT for 16 h, then reduced *in vacuo*. Purification by flash column chromatography (SiO_2, 2:1 hexane:ethyl acetate → ethyl acetate → 9:1 ethyl acetate:methanol) afforded the *title compound* (as a 1:3 mixture of rotamers) as a colourless oil (46 mg, 85%); R_f 0.11 (ethyl acetate); ν_{max}/cm^{-1} (thin film) 3288, 3088, 2932, 1727, 1647, 1552; δ_H (400 MHz, $CDCl_3$) 5.95 (1H, s, N**H**, minor rotamer), 5.57 (1H, s, N**H**, major rotamer), 4.54–4.40 (4H, m, C**H**$_2$O, both rotamers), 3.41–3.18 (4H, m, C**H**$_2$NH, both), 2.60–2.45 (4H, m, C**H**$_2$CONH, both), 2.38–2.25 (4H, m, C**H**$_2$CO$_2$, both), 1.88–1.77 (4H, m, C**H**$_2$, both), 1.74–1.61 (4H, m, C**H**$_2$, both); δ_C (100 MHz, $CDCl_3$) data for the major rotamer only: 174.9 (**C**O), 170.1 (**C**O), 61.0 (**C**H$_2$O), 40.1 (**C**H$_2$NH), 37.1 (**C**H$_2$CO), 36.5 (**C**H$_2$CO), 28.1 (**C**H$_2$), 24.5 (**C**H$_2$). Diagnostic ^{13}C NMR resonances for the minor rotamer: 174.3 (**C**O), 173.4 (**C**O), 41.0 (**C**H$_2$NH), 35.0 (**C**H$_2$CO), 31.2 (**C**H$_2$CO), 30.0 (**C**H$_2$), 20.4 (**C**H$_2$); HRMS (ESI): calcd. for $C_8H_{14}NO_3$, 172.0968. Found: $[MH]^+$, 172.0970 (0.6 ppm error).

1-Oxa-5-azacycloundecane-4,11-dione (13e)

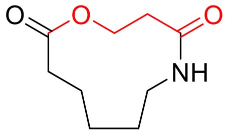

1-(3-(Benzyloxy)propanoyl)azepan-2-one (115 mg, 0.422 mmol) was dissolved in ethyl acetate (4.2 mL) and placed under an argon atmosphere. Palladium on carbon (42 mg, Pd 10% on carbon) was then added and the reaction vessel was backfilled with hydrogen (via balloon) several times, then stirred at RT under a slight positive pressure of hydrogen (balloon) for 1 h. The reaction was then purged with argon, filtered through Celite, washed with methanol where the solvent was removed *in vacuo* to afford the title compound as a white solid (74 mg, 94%); m.p: 103–105 °C; R_f 0.31 (ethyl acetate); ν_{max}/cm^{-1} (thin film) 3288, 2932, 1724, 1637, 1559; δ_H (400 MHz, $CDCl_3$) 6.16 (1H, s, N**H**), 4.51–4.35 (2H, m, C**H**$_2$O), 3.40–3.23 (2H, m, C**H**$_2$), 2.54–2.38 (2H, m, C**H**$_2$CONH), 2.34–2.20 (2H, m, C**H**$_2$CO$_2$), 1.69–1.55 (2H, m, C**H**$_2$), 1.52–1.39 (2H, m, C**H**$_2$), 1.36–1.24 (2H, m, C**H**$_2$); δ_C (100 MHz, $CDCl_3$) 173.1 (**C**O), 171.1 (**C**O), 60.9 (**C**H$_2$O), 39.1 (**C**H$_2$N), 37.1 (**C**H$_2$CONH), 34.5 (**C**H$_2$CO$_2$), 27.2 (**C**H$_2$), 25.8 (**C**H$_2$), 23.9 (**C**H$_2$); HRMS (ESI): calcd. for $C_9H_{16}NO_3$, 186.1125. Found: $[MH]^+$, 186.1126 (3.7 ppm error).

1-Oxa-5-azacyclododecane-4,12-dione (13f)

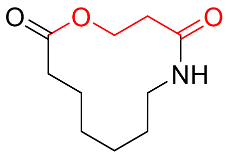

1-(3-(Benzyloxy)propanoyl)azocan-2-one (105 mg, 0.363 mmol) was dissolved in ethyl acetate (3.6 mL) and placed under an argon atmosphere. Palladium on carbon (36 mg, Pd 10% on carbon) was then added and the reaction vessel was backfilled with hydrogen (via balloon) several times, then stirred at RT under a slight positive pressure of hydrogen (balloon) for 1 h. The reaction was then purged with argon, filtered through Celite, washed with methanol where the solvent was removed *in vacuo*. The crude material was then re-dissolved in chloroform (3.6 mL) and triethylamine (76 uL, 0.545 mmol) was added, and then stirred at RT for 16 h, then reduced *in vacuo*. Purification by flash column chromatography (SiO_2, 2:1 hexane:ethyl acetate → ethyl acetate → 9:1 ethyl acetate:methanol) afforded the *title compound* as a white solid (68 mg, 94%); mp. 128–129 °C; R_f 0.25 (ethyl acetate); ν_{max}/cm^{-1} (thin film) 3301, 2922, 2858, 1723, 1639, 1561; δ_H (400 MHz, $CDCl_3$) 6.13 (1H, s, N**H**), 4.54–4.30 (2H, m, C**H₂**O), 3.45–3.21 (2H, m, C**H₂**NH), 2.59–2.43 (2H, m, C**H₂**CO), 2.42–2.21 (2H, m, C**H₂**CO), 1.78–1.11 (8H, m, 4 × C**H₂**); δ_C (100 MHz, $CDCl_3$) 171.7 (**C**O), 168.9 (**C**O), 59.4 (**C**H_2O), 36.7 (**C**H_2NH), 35.4 (**C**H_2CO), 32.0 (**C**H_2CO), 23.9 (**C**H_2), 23.8 (**C**H_2), 23.0 (**C**H_2), 22.4 (**C**H_2); HRMS (ESI): calcd. for $C_{10}H_{17}NNaO_3$, 222.1101. Found: $[MNa]^+$, 222.1103 (−1.5 ppm error).

1-Oxa-5-azacyclotridecane-4,13-dione (13g)

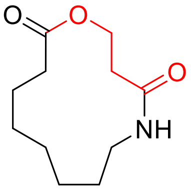

1-(3-(Benzyloxy)propanoyl)azonan-2-one (220 mg, 0.725 mmol) was dissolved in ethyl acetate (7.2 mL) and placed under an argon atmosphere. Palladium on carbon (72 mg, Pd 10% on carbon) was then added and the reaction vessel was backfilled with hydrogen (via balloon) several times, then stirred at RT under a slight positive pressure of hydrogen (balloon) for 1 h. The reaction was then purged with argon, filtered through Celite, washed with methanol where the solvent was removed *in vacuo*. The crude material was dissolved in chloroform (7.2 mL) and trimethylamine (152 µL, 1.09 mmol) was added and stirred at RT for 16 h, after which the solvent was then reduced *in vacuo*. Purification by flash column chromatography (SiO_2, 2:1 hexane:ethyl acetate → ethyl acetate → 9:1 ethyl acetate:methanol) to afford the title

compound as a white solid (142 mg, 92%); mp. 129–130 °C; R_f 0.31 (ethyl acetate); ν_{max}/cm^{-1} (thin film) 3299, 2928, 2847, 1724, 1641, 1554; δ_H (400 MHz, $CDCl_3$) 5.85 (1H, s, N**H**), 4.36 (2H, t, *J* = 5.3 Hz, C**H$_2$**O), 3.37–3.21 (2H, m, C**H$_2$**NH), 2.50 (2H, t, *J* = 5.3 Hz, C**H$_2$**CH$_2$O), 2.42–2.30 (2H, m, C**H$_2$**COO), 1.73–1.60 (2H, m, C**H$_2$**), 1.59–1.50 (2H, m, C**H$_2$**), 1.47–1.25 (6H, m, 3 × C**H$_2$**); δ_C (100 MHz, $CDCl_3$) 173.5 (**C**O), 170.3 (**C**O), 61.7 (**C**H$_2$O), 40.3 (**C**H-$_2$NH), 36.6 (**C**H$_2$CO), 34.1 (**C**H$_2$CO), 27.2 (**C**H$_2$), 26.8 (**C**H$_2$), 26.4 (**C**H$_2$), 25.5 (**C**H$_2$), 24.2 (**C**H$_2$); HRMS (ESI): calcd. for $C_{11}H_{19}NNaO_3$, 236.1257. Found: $[MNa]^+$, 236.1257 (−0.6 ppm error).

1-Oxa-5-azacyclotetradecane-4,14-dione (13h)

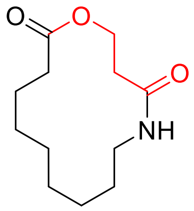

1-(3-(Benzyloxy)propanoyl)azecan-2-one (212 mg, 0.630 mmol) was dissolved in ethyl acetate (6.8 mL) and placed under an argon atmosphere. Palladium on carbon (68 mg, Pd 10% on carbon) was then added and the reaction vessel was backfilled with hydrogen (via balloon) several times, then stirred at RT under a slight positive pressure of hydrogen (balloon) for 1 h. The reaction was then purged with argon, filtered through Celite, washed with methanol where the solvent was removed *in vacuo*. The crude material was then re-dissolved in chloroform (6.3 mL) and triethylamine (131 uL, 0.945 mmol) was added, and then stirred at RT for 16 h, then reduced *in vacuo*. Purification by flash column chromatography (SiO_2, 2:1 hexane:ethyl acetate → ethyl acetate → 9:1 ethyl acetate:methanol) afforded the *title compound* as a white solid (137 mg, 96%); m.p. 121–122 °C; R_f 0.40 (ethyl acetate); ν_{max}/cm^{-1} (thin film) 3284, 3096, 2929, 2861, 1722, 1646, 1556; δ_H (400 MHz, $CDCl_3$) 5.87 (1H, br s, N**H**), 4.38 (2H, t, *J* = 5.2 Hz, C**H$_2$**O), 3.35–3.21 (2H, m, C**H$_2$**NH), 2.54–2.43 (2H, m, C**H$_2$**CONH), 2.39–2.28 (2H, m, C**H$_2$**COO), 1.64–1.20 (12H, m, 6 × C**H$_2$**); δ_C (100 MHz, $CDCl_3$) 173.6 (**C**O), 170.2 (**C**O), 61.4 (**C**H$_2$O), 38.9 (**C**H$_2$NH), 36.4 (**C**H$_2$CO), 34.7 (**C**H$_2$CO), 28.1 (**C**H$_2$), 25.9 (**C**H$_2$), 25.7 (**C**H$_2$), 25.6 (**C**H$_2$), 23.9 (**C**H$_2$), 23.0 (**C**H$_2$); HRMS (ESI): calcd. for $C_{12}H_{21}NNaO_3$, 250.1414. Found: $[MNa]^+$, 250.1415 (−0.3 ppm error).

1-Oxa-5-azacyclopentadecane-4,15-dione (13i)

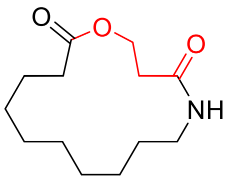

1-(3-(Benzyloxy)propanoyl)azacycloundecan-2-one (193 mg, 0.582 mmol) was dissolved in ethyl acetate (5.8 mL) and placed under an argon atmosphere. Palladium on carbon (58 mg, Pd 10% on carbon) was then added and the reaction vessel was backfilled with hydrogen (via balloon) several times, then stirred at RT under a slight positive pressure of hydrogen (balloon) for 1 h. The reaction was then purged with argon, filtered through Celite, washed with methanol where the solvent was removed *in vacuo*. The crude material was then re-dissolved in chloroform (5.5 mL) and triethylamine (115 uL, 0.825 mmol) was added, and then stirred at RT for 16 h, then reduced *in vacuo*. Purification by flash column chromatography (SiO_2, 2:1 hexane:ethyl acetate → ethyl acetate → 9:1 ethyl acetate:methanol) afforded the *title compound* (as a 10:1 mixture of rotamers) as a white solid (130 mg, 92%); m.p. 126–128 °C; R_f 0.42 (ethyl acetate); ν_{max}/cm^{-1} (thin film) 3311, 2924, 2855, 1725, 1646, 1549; δ_H (400 MHz, $CDCl_3$) 5.90 (1H, s, N**H**, minor rotamer), 5.72 (1H, s, N**H**, major rotamer), 4.46–4.27 (4H, m, C**H₂**O, both rotamers), 3.42–3.20 (4H, m, C**H₂**NH, both), 2.57–2.40 (4H, m, C**H₂**CONH, both), 2.39–2.22 (4H, m, C**H₂**COO, both), 1.69–1.15 (28H, m, 7 × C**H₂**, both); δ_C (100 MHz, $CDCl_3$) data for the major rotamer only: 174.2 (**C**O), 170.1 (**C**O), 61.1 (**C**H_2O), 38.5 (**C**H_2NH), 36.1 (**C**H_2CO), 33.7 (**C**H_2CO), 28.3 (**C**H_2), 26.9 (**C**H_2), 26.7 (**C**H_2), 25.8 (**C**H_2), 25.7 (**C**H_2), 25.1 (**C**H_2), 23.8 (**C**H_2). Diagnostic ^{13}C NMR resonances for minor rotamer: 173.7 (**C**O), 170.3 (**C**O), 61.4 (**C**H_2O), 38.9 (**C**H_2NH), 36.4 (**C**H_2CO), 34.7 (**C**H_2CO); HRMS (ESI): calcd. for $C_{13}H_{23}NNaO_3$, 264.1570. Found: $[MNa]^+$, 264.1578 (−2.7 ppm error).

1-Oxa-5-azacyclohexadecane-4,16-dione (13j)

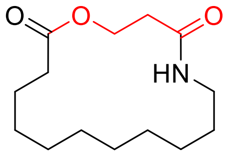

1-(3-(Benzyloxy)propanoyl)azacyclododecan-2-one (180 mg, 0.521 mmol) was dissolved in ethyl acetate (5.2 mL) and placed under an argon atmosphere. Palladium on carbon (52 mg, Pd 10% on carbon) was then added and the reaction vessel was backfilled with hydrogen (via balloon) several times, then stirred at RT under a slight positive pressure of hydrogen (balloon) for 1 h. The reaction was then purged with argon, filtered through Celite, washed with methanol

where the solvent was removed *in vacuo*. The crude material was then re-dissolved in chloroform (5.2 mL) and triethylamine (110 µL, 0.782 mmol) was added, and then stirred at RT for 16 h, then reduced *in vacuo*. Purification by flash column chromatography (SiO_2, 2:1 hexane:ethyl acetate → ethyl acetate → 9:1 ethyl acetate:methanol) afforded the *title compound* (as a 7:2 mixture of rotamers) as a white solid (119 mg, 91%); m.p. 127–129 °C; R_f 0.45 (ethyl acetate); ν_{max}/cm^{-1} (thin film) 3272, 2930, 2860, 1722, 1643, 1559; δ_H (400 MHz, $CDCl_3$) 5.89 (1H, s, N**H**, major rotamer), 5.80 (1H, s, N**H**, minor rotamer), 4.41–4.29 (4H, m, C**H₂**O, both rotamers), 3.36–3.24 (4H, m, C**H₂**NH, both), 2.48 (2H, t, *J* = 5.3 Hz, C**H₂**CONH, minor), 2.43 (2H, t, *J* = 5.3 Hz, C**H₂**CONH, major), 2.34–2.28 (2H, m, C**H₂**COO, minor), 2.24 (2H, t, *J* = 7.7 Hz, C**H₂**COO, major), 1.67–1.56 (4H, m, C**H₂**, both), 1.53–1.42 (4H, m, C**H₂**, both), 1.39–1.15 (24H, m, 6 × C**H₂**, both); δ_C (100 MHz, $CDCl_3$) data for the major rotamer only: 173.7 (**C**O), 170.1 (**C**O), 60.6 (**C**H₂O), 39.2 (**C**H₂NH), 36.2 (**C**H₂CO), 33.6 (**C**H₂CO), 29.1 (**C**H₂), 26.8 (**C**H₂), 26.52 (**C**H₂), 26.46 (**C**H₂), 25.5 (**C**H₂), 25.1 (**C**H₂), 24.8 (**C**H₂), 23.7 (**C**H₂). Diagnostic ^{13}C NMR resonances for minor rotamer: 174.1 (**C**O), 61.0 (**C**H₂O), 38.5 (**C**H₂NH), 36.0 (**C**H₂CO), 33.7 (**C**H₂CO); HRMS (ESI): calcd. for $C_{14}H_{25}NNaO_3$, 278.1727. Found: $[MNa]^+$, 278.1732 (−0.9 ppm error).

1,8-Dioxa-4-azacycloundecane-5,9-dione (16a)

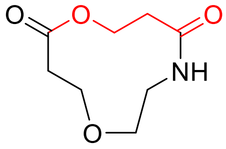

A mixture of 1,4-oxazepan-5-one (422 mg, 3.66 mmol), DMAP (45 mg, 0.370 mmol) and pyridine (1.77 mL, 21.9 mmol) in DCM (26 mL) under an argon atmosphere was stirred at RT for 5 mins. Next, a solution of acid chloride (5.49 mmol, 1.5 equiv. prepared using the general procedure) in DCM (14 mL) was added and the resulting mixture was heated, under reflux, at 50 °C for 16 h. An additional solution of acid chloride (5.49 mmol, 1.5 equiv. prepared using the general procedure) in DCM (14 mL) was added and the reaction heated, under reflux, at 50 °C for another 12 h to achieve reaction completion. The solvent was concentrated *in vacuo*, loaded onto a short silica plug and eluted with 7:3 hexane:ethyl acetate → 1:1 hexane:ethyl acetate to remove the excess carboxylic acid and pyridine, and concentrate *in vacuo*. This material was re-dissolved in ethyl acetate (37 mL) and placed under an argon atmosphere. Palladium on carbon (366 mg, Pd 10% on carbon) was then added and the reaction vessel was backfilled with hydrogen (*via* balloon) several times, then stirred at RT under a slight positive pressure of hydrogen (balloon) for 16 h. The reaction was then purged with argon, filtered

through Celite, washed with methanol and the solvent was removed *in vacuo*. The crude material was re-dissolved in chloroform (37 mL) and triethylamine (0.760 mL, 5.49 mmol) added, and stirred for 16 h. After removal of solvent under reduced pressure, the mixture was purified by flash column chromatography (SiO_2, ethyl acetate → 9:1 ethyl acetate:methanol) to afford the *title compound* as a white solid (514 mg, 75%); m.p. 80−82 °C; R_f 0.29 (9:1 ethyl acetate:methanol); ν_{max} (thin film)/cm^{-1} 3299, 2930, 1734, 1652, 1555; δ_H (400 MHz, $CDCl_3$) 6.09 (1H, br s, NH), 4.45 (2H, t, *J* = 6.1 Hz, COO-CH_2), 3.76 (2H, t, *J* = 5.3 Hz, C**H**$_2$-CH_2-COO), 3.53 (2H, t, *J* = 4.9 Hz, C**H**$_2$-CH_2-NH), 3.27 (2H, q, *J* = 5.3 Hz, C**H**$_2$-NH), 2.48 (4H, m, CH_2-COO and C**H**$_2$-CO-NH); δ_C (100 MHz, $CDCl_3$) 171.9 (COO), 171.1 (CO-NH), 70.2 (**C**H_2-CH_2-NH), 68.4 (**C**H_2-CH_2-COO), 61.4 (COO-**C**H_2), 40.2 (CH_2-NH), 37.3 (**C**H_2-CO-NH), 36.9 (**C**H_2-COO); HRMS (ESI$^+$): calcd. for $C_8H_{13}NNaO_4$, 210.0737. Found: [MNa^+], 210.0738 (−0.6 ppm error).

3,4,6,7,8,9-Hexahydrobenzo[*i*][1]oxa[5]azacycloundecine-1,5-dione (16b)

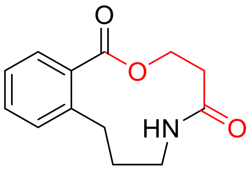

A mixture of 2,3,4,5-tetrahydro-1H-benzo[*c*]azepin-1-one (127 mg, 0.786 mmol), DMAP (10 mg, 0.0786 mmol) and pyridine (380 µL, 4.72 mmol) in DCM (5.5 mL) under an argon atmosphere was stirred at RT for 5 mins. Next, a solution of acid chloride (1.18 mmol, 1.50 equiv. prepared using the general procedure) in DCM (3 mL) was added and the resulting mixture was heated, at reflux, at 50 °C for 16 h. The solvent was concentrated *in vacuo*, loaded onto a short silica plug and eluted with 2:1 hexane:ethyl acetate, to remove the majority of excess carboxylic acid and pyridine residues, and concentrated *in vacuo*. This material was re-dissolved in ethyl acetate (7.8 mL) and placed under an argon atmosphere. Palladium on carbon (78 mg, Pd 10% on carbon) was then added, and the reaction vessel was backfilled with hydrogen (via balloon) several times, then stirred at RT under a slight positive pressure of hydrogen (balloon) for 16 h. The reaction was purged with argon, filtered through Celite, washed with methanol where the solvent was removed *in vacuo*. The crude material was then re-dissolved in chloroform (7.8 mL) and triethylamine (165 µL, 1.18 mmol) added, and stirred at RT for 16 h, then reduced *in vacuo*. Purification by flash column chromatography (SiO_2, 2:1 hexane:ethyl acetate → ethyl acetate) afforded the *title compound* as a colourless oil (98 mg, 53%). 2-(3-Hydroxypropanoyl)-2,3,4,5-tetrahydro-1*H*-benzo[*c*]azepin-1-one (48 mg, 26%)

was also isolated, and re-subjected to the reaction conditions where it was re-dissolved in chloroform (2.1 mL) and triethylamine (45 µL, 0.309 mmol) was added and stirred at RT for 16 h. Purification of this second reaction mixture by flash column chromatography (SiO_2, 2:1 hexane:ethyl acetate → ethyl acetate) afforded the *title compound*, which was combined with the initial purified compound, as a colourless oil (130 mg, 71%); R_f 0.32 (ethyl acetate); ν_{max}/cm^{-1} (thin film) 3302, 3071, 2949, 1703, 1651, 1547; δ_H (400 MHz, $CDCl_3$) 7.76 (1H, d, *J* = 6.9 Hz, C**H**), 7.40–7.31 (1H, m, C**H**), 7.23–7.17 (1H, m, C**H**), 7.13 (1H, d, *J* = 7.6 Hz, C**H**), 6.16 (1H, br s, N**H**), 4.57 (2H, t, *J* = 6.1 Hz, C**H₂**O), 3.41–3.19 (2H, m, C**H₂**NH), 2.86 (2H, t, *J* = 7.6 Hz, C**H₂**C), 2.58 (2H, t, *J* = 6.1 Hz, C**H₂**CON), 1.80–1.63 (2H, m, C**H₂**CH₂NH); δ_C (100 MHz, $CDCl_3$) 172.6 (**C**O), 168.8 (**C**O), 142.9 (**C**), 132.1 (**C**H), 131.3 (**C**H), 131.1 (**C**H), 130.0 (**C**), 126.3 (**C**H), 63.2 (**C**H₂O), 40.1 (**C**H₂NH), 37.0 (**C**H₂C), 32.1 (**C**H₂CON), 30.4 (**C**H₂CH₂NH); HRMS (ESI): calcd. for $C_{13}H_{15}NNaO_3$, 256.0944. Found: $[MNa]^+$, 256.0945 (−0.2 ppm error).

4,5,6,7,8,9-Hexahydro-2H-benzo[b][1]oxa[5]azacyclododecine-2,10(3*H*)-dione (16c)

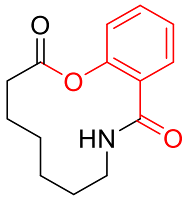

A mixture of azacyclooctan-2-one (100 mg, 0.786 mmol), DMAP (10 mg, 0.0786 mmol) and pyridine (380 µL, 4.72 mmol) in DCM (5.5 mL) under an argon atmosphere was stirred at RT for 5 mins. Next, a solution of acid chloride (1.18 mmol, 1.50 equiv. prepared using the general procedure) in DCM (3 mL) was added and the resulting mixture was heated, at reflux, at 50 °C for 16 h. An additional solution of acid chloride (1.18 mmol, 1.50 equiv. prepared using the general procedure) in DCM (3 mL) was added and heated, to reflux, at 50 °C for a further 16 h in order to achieve reaction completion. The solvent was then concentrated *in vacuo*, loaded onto a short silica plug and eluted with 2:1 hexane:ethyl acetate, to remove the majority of excess carboxylic acid and pyridine residues, and concentrated *in vacuo*. This material was re-dissolved in ethyl acetate (7.8 mL) and placed under an argon atmosphere. Palladium on carbon (78 mg, Pd 10% on carbon) was then added and the reaction vessel was backfilled with hydrogen (via balloon) several times, then stirred at RT under a slight positive pressure of hydrogen (balloon) for 1 h. The reaction was then purged with argon, filtered through Celite, washed with methanol where the solvent was removed *in vacuo*. The crude material was then re-dissolved in chloroform (7.8 mL) and triethylamine (165 µL, 1.18 mmol) added, and stirred

at RT for 16 h, then reduced *in vacuo*. Purification by flash column chromatography (SiO_2, 2:1 hexane:ethyl acetate → ethyl acetate) afforded the *title compound* as a white solid (174 mg, 90%); mp. 138.0–140.0 °C; R_f 0.62 (ethyl acetate); ν_{max}/cm^{-1} (thin film) 3296, 2939, 2900, 2849, 1742, 1632, 1542; δ_H (400 MHz, $CDCl_3$) 7.74 (1H, d, *J* = 7.7 Hz, C**H**), 7.44–7.35 (1H, m, C**H**), 7.30–7.20 (1H, m, C**H**), 7.01 (1H, d, *J* = 8.1 Hz, C**H**), 6.46 (1H, br s, N**H**), 3.50–3.34 (2H, m, C**H₂**NH), 2.67–2.53 (2H, m, C**H₂**COO), 1.90–1.75 (2H, m, C**H₂**), 1.71–1.61 (2H, m, C**H₂**), 1.59–1.43 (4H, m, 2 × C**H₂**); δ_C (100 MHz, $CDCl_3$) 171.9 (**C**O), 165.4 (**C**O), 147.2 (**C**), 131.5 (**C**H), 130.5 (**C**H), 129.1 (**C**), 126.5 (**C**H), 123.3 (**C**H), 40.1 (**C**H_2NH), 34.6 (**C**H_2CO), 26.3 (**C**H_2), 25.8 (**C**H_2), 24.7 (**C**H_2), 22.5 (**C**H_2); HRMS (ESI): calcd. for $C_{14}H_{17}NNaO_3$, 270.1101. Found: $[MNa]^+$, 270.1107 (−2.1 ppm error).

2-Methyl-1-oxa-4-azacyclohexadecane-3,16-dione (16d)

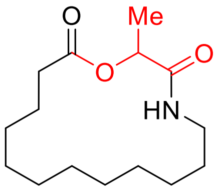

A mixture of laurolactam (155 mg, 0.786 mmol), DMAP (10 mg, 0.0786 mmol) and pyridine (380 µL, 4.72 mmol) in DCM (5.5 mL) under an argon atmosphere was stirred at RT for 5 mins. Next, a solution of acid chloride (1.18 mmol, 1.50 equiv. prepared using the general procedure) in DCM (3 mL) was added and the resulting mixture was heated, at reflux, at 50 °C for 16 h. The solvent was concentrated *in vacuo*, loaded onto a short silica plug and eluted with 2:1 hexane:ethyl acetate, to remove the majority of excess carboxylic acid and pyridine residues, and concentrated *in vacuo*. This material was re-dissolved in THF (7.8 mL) and placed under an argon atmosphere. Palladium on carbon (78 mg, Pd 10% on carbon) and water (1.42 mL, 78.6 mmol) was then added and the reaction vessel was backfilled with hydrogen (via balloon) several times, then stirred at RT under a slight positive pressure of hydrogen (balloon) for 2 h. The reaction was then purged with argon, filtered through Celite, washed with methanol where the solvent was removed *in vacuo*. The crude material was then re-dissolved in chloroform (7.8 mL) and triethylamine (165 µL, 1.18 mmol) added, and stirred at RT for 16 h, then reduced *in vacuo*. Purification by flash column chromatography (SiO_2, 2:1 hexane:ethyl acetate → ethyl acetate) afforded the *title compound* as a colourless oil (124 mg, 59%). 1-(2-Hydroxypropanoyl)azacyclotridecan-2-one (82 mg, 38%) was also isolated, and re-subjected to the reaction conditions where it was re-dissolved in chloroform (3.0 mL) and triethylamine (65 µL, 0.452 mmol) was added and stirred at RT for 16 h. Purification of this

second reaction mixture by flash column chromatography (SiO_2, 2:1 hexane:ethyl acetate → ethyl acetate) afforded the *title compound*, which was combined with the initial purified compound, as a colourless oil (180 mg, 85%); R_f 0.67 (ethyl acetate); ν_{max}/cm^{-1} (thin film) 3314, 2928, 2857, 1738, 1659, 1536; δ_H (400 MHz, $CDCl_3$) 6.24 (1H, br s, N**H**), 5.17 (1H, q, J = 6.9 Hz, C**H**CH_3), 3.47–3.37 (1H, m, C**H**H′NH), 3.20 (1H, m, CH**H′**NH), 2.39 (2H, m, C**H₂**COO), 1.73–1.14 (21H, m, 9 × C**H₂** and C**H₃**); δ_C (100 MHz, $CDCl_3$) 172.2 (**C**O), 170.4 (**C**O), 70.8 (**C**HO), 38.6 (**C**H_2NH), 34.8 (**C**H_2COO), 28.5 (**C**H_2), 27.1 (**C**H_2), 27.0 (**C**H_2), 26.40 (**C**H_2), 26.36 (**C**H_2), 25.7 (**C**H_2), 24.9 (**C**H_2), 24.7 (**C**H_2), 24.6 (**C**H_2), 18.1 (**C**H_3); HRMS (ESI): calcd. for $C_{15}H_{28}NO_3$, 270.2064. Found: $[MH]^+$, 270.2065 (−0.1 ppm error).

2-Phenyl-1-oxa-4-azacyclohexadecane-3,16-dione (16e)

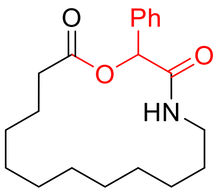

A mixture of laurolactam (155 mg, 0.786 mmol), DMAP (10 mg, 0.0786 mmol) and pyridine (380 µL, 4.72 mmol) in DCM (5.5 mL) under an argon atmosphere was stirred at RT for 5 mins. Next, a solution of acid chloride (1.18 mmol, 1.50 equiv. prepared using the general procedure) in DCM (3 mL) was added and the resulting mixture was heated, at reflux, at 50 °C for 16 h. The solvent was concentrated *in vacuo*, loaded onto a short silica plug and eluted with 2:1 hexane:ethyl acetate, to remove the majority of excess carboxylic acid and pyridine residues, and concentrated *in vacuo*. This material was re-dissolved in THF (7.8 mL) and placed under an argon atmosphere. Palladium on carbon (78 mg, Pd 10% on carbon) and water (1.42 mL, 78.6 mmol) was then added and the reaction vessel was backfilled with hydrogen (via balloon) several times, then stirred at RT under a slight positive pressure of hydrogen (balloon) for 2 h. The reaction was then purged with argon, filtered through Celite, washed with methanol where the solvent was removed *in vacuo*. The crude material was then re-dissolved in chloroform (7.8 mL) and triethylamine (165 µL, 1.18 mmol) added, and stirred at RT for 16 h, then reduced *in vacuo*. Purification by flash column chromatography (SiO_2, 2:1 hexane:ethyl acetate → ethyl acetate) afforded the *title compound* as a white solid (176 mg, 68%); mp. 126–128 °C; R_f 0.73 (ethyl acetate); ν_{max}/cm^{-1} (thin film) 3300, 2927, 2856, 1736, 1654, 1537; δ_H (400 MHz, $CDCl_3$) 7.53–7.16 (5H, m, Ph), 6.41 (1H, br s, N**H**), 6.13 (1H, s, C**H**Ph), 3.61–3.50 (1H, m, C**H**H′NH), 3.21–3.10 (1H, m, CH**H′**NH), 2.49–2.34 (2H, m, C**HH′**COO), 1.83–1.21 (18H, m, 9 × C**H₂**); δ_C (100 MHz, $CDCl_3$) 171.7 (**C**O), 168.4 (**C**O),

136.0 (**C**), 128.9 (**C**H), 128.7 (**C**H), 127.3 (**C**H), 75.6 (**C**HPh), 38.7 (**C**H_2NH), 34.8 (**C**H_2CO), 28.8 (**C**H_2), 27.2 (**C**H_2), 27.1 (**C**H_2), 26.6 (**C**H_2), 26.5 (**C**H_2), 25.7 (**C**H_2), 25.0 (**C**H_2), 24.8 (**C**H_2), 24.6 (**C**H_2); HRMS (ESI): calcd. for $C_{20}H_{29}NNaO_3$, 354.2040. Found: $[MNa]^+$, 354.2041 (−0.5 ppm error).

3-(Ethylamino)-3-oxopropyl propionate (16f)

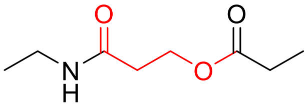

A mixture of *N*-ethylpropionamide (80 mg, 0.786 mmol), DMAP (10 mg, 0.0786 mmol) and pyridine (380 µL, 4.72 mmol) in DCM (5.5 mL) under an argon atmosphere was stirred at RT for 5 mins. Next, a solution of acid chloride (1.18 mmol, 1.50 equiv. prepared using the general procedure) in DCM (3 mL) was added and the resulting mixture was heated, at reflux, at 50 °C for 16 h. The solvent was concentrated *in vacuo*, loaded onto a short silica plug and eluted with 2:1 hexane:ethyl acetate, to remove the majority of excess carboxylic acid and pyridine residues, and concentrated *in vacuo*. This material was re-dissolved in ethyl acetate (7.8 mL) and placed under an argon atmosphere. Palladium on carbon (78 mg, Pd 10% on carbon) was then added and the reaction vessel was backfilled with hydrogen (via balloon) several times, then stirred at RT under a slight positive pressure of hydrogen (balloon) for 1 h. The reaction was then purged with argon, filtered through Celite, washed with methanol where the solvent was removed *in vacuo*. The crude material was then re-dissolved in chloroform (7.8 mL) and triethylamine (165 µL, 1.18 mmol) added, and stirred at RT for 16 h, then reduced *in vacuo*. Purification by flash column chromatography (SiO_2, 2:1 hexane:ethyl acetate → ethyl acetate) afforded the *title compound* as a colourless oil (102 mg, 75%); R_f 0.36 (ethyl acetate); ν_{max}/cm^{-1} (thin film) 3299, 3094, 2978, 2941, 1736, 1644, 1551; δ_H (400 MHz, $CDCl_3$) 6.12 (1H, br s, N**H**), 4.36–4.25 (2H, m, C**H₂**O), 3.29–3.16 (2H, m, C**H₂**NH), 2.43 (2H, t, *J* = 6.3 Hz, C**H₂**CONH), 2.25 (2H, q, *J* = 7.6 Hz, C**H₂**COO), 1.15–0.97 (6H, m, 2 × C**H₃**); δ_C (100 MHz, $CDCl_3$) 174.5 (**C**O), 169.9 (**C**O), 60.6 (**C**H_2O), 35.9 (**C**H_2NH), 34.5 (**C**H_2CON), 27.5 (**C**H_2COO), 14.8 (**C**H_3), 9.1 (**C**H_3); HRMS (ESI): calcd. for $C_8H_{15}NNaO_3$, 1986.0944. Found: $[MNa]^+$, 196.0948 (−1.5 ppm error).

1,7,11-Trioxa-4-azacyclotetradecane-3,10,14-trione (17a)

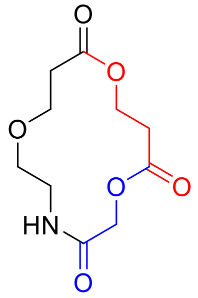

A mixture of 1,8-dioxa-4-azacycloundecane-5,9-dione (126 mg, 0.670 mmol), DMAP (8 mg, 0.0670 mmol) and pyridine (0.320 mL, 4.00 mmol) in DCM (4.7 mL) under an argon atmosphere was stirred at RT for 30 mins. A solution of acid chloride (1.01 mmol, 1.5 equiv. prepared using the general procedure) in DCM (2.6 mL) was added and the resulting mixture was heated, at reflux, at 50 °C for 16 h. An additional solution of acid chloride (1.01 mmol, 1.5 equiv. prepared using the general procedure) in DCM (2.6 mL) was added and the reaction heated, at reflux, at 50 °C for another 12 h to achieve reaction completion. The solvent was concentrated *in vacuo*, loaded onto a short silica plug and eluted with 7:3 hexane:ethyl acetate → 1:1 hexane:ethyl acetate to remove the excess carboxylic acid and pyridine, and concentrated *in vacuo*. This material was re-dissolved in THF (6.6 mL) and placed under an argon atmosphere. Palladium on carbon (66 mg, Pd 10% on carbon) and water (1.2 mL, 67 mmol) was added and the reaction vessel was backfilled with hydrogen (*via* balloon) several times. The reaction was stirred at RT under a slight positive pressure of hydrogen (balloon) for 16 h. The reaction was then purged with argon, filtered through Celite, washed with methanol and the solvent was removed *in vacuo*. The crude material was re-dissolved in chloroform (6.7 mL) and triethylamine (0.140 mL, 1.01 mmol) added, and stirred for 12 h. After removal of solvent *in vacuo*, the mixture was purified by flash column chromatography (SiO_2, ethyl acetate → 9:1 ethyl acetate:methanol) to afford the *title compound* as a white crystalline solid (125 mg, 76%); m.p. 85–88 °C; R_f 0.71 (4:1 ethyl acetate:methanol); ν_{max} (thin film)/cm^{-1} 3399, 2874, 1717, 1670, 1542; δ_H (400 MHz, $CDCl_3$) 6.54 (1H, br s, NH), 4.68 (2H, s, C**H**$_2$-CO-NH), 4.50 (2H, t, J = 5.3 Hz, COO-C**H**$_2$-CH$_2$-COO), 3.74 (2H, t, J = 5.5 Hz, OC**H**$_2$-CH$_2$-COO), 3.53 (2H, t, J = 4.6 Hz, OC**H**$_2$-CH$_2$-NH), 3.47 (2H, q, J = 4.6 Hz, C**H**$_2$-NH), 2.83 (2H, t, J = 5.3 Hz, COO-CH$_2$-C**H**$_2$-COO), 2.61 (2H, t, J = 5.5 Hz, OCH$_2$-C**H**$_2$-COO); δ_C (100 MHz, $CDCl_3$) 171.7 (OCH$_2$-CH$_2$-**C**OO), 169.3 (**C**OO-CH$_2$-CO-NH), 166.8 (CO-NH), 68.7 (O**C**H$_2$-CH$_2$-NH), 66.3 (O**C**H$_2$-CH$_2$-COO), 62.9 (COO-**C**H$_2$-CO-NH), 60.7 (COO-**C**H$_2$-CH$_2$-COO), 38.3 (CH$_2$-NH), 35.5 (OCH$_2$-**C**H$_2$-COO), 34.3 (COO-CH$_2$-**C**H$_2$-COO); HRMS (ESI$^+$): calcd. for $C_{10}H_{15}NNaO_6$, 268.0792. Found: [MNa$^+$], 268.0790 (0.4 ppm error).

7,8,10,11,12,13-Hexahydro-3*H*-benzo[*m*][1,5]dioxa[9]azacyclopentadecine-1,5,9(4*H*)-trione (17b)

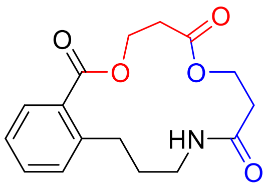

A mixture of 3,4,6,7,8,9-hexahydrobenzo[*i*][1]oxa[5]azacycloundecine-1,5-dione (60 mg, 0.269 mmol), DMAP (4 mg, 0.0269 mmol) and pyridine (130 μL, 1.61 mmol) in DCM (2.0 mL) under an argon atmosphere was stirred at RT for 5 mins. Next, a solution of acid chloride (0.403 mmol, 1.50 equiv. prepared using the general procedure) in DCM (1 mL) was added and the resulting mixture was heated, at reflux, at 50 °C for 16 h. The solvent was then concentrated *in vacuo*, loaded onto a short silica plug and eluted with 2:1 hexane:ethyl acetate, to remove the majority of excess carboxylic acid and pyridine residues, and concentrated *in vacuo*. This material was re-dissolved in ethyl acetate (2.7 mL) and placed under an argon atmosphere. Palladium on carbon (27 mg, Pd 10% on carbon) was then added and the reaction vessel was backfilled with hydrogen (via balloon) several times, then stirred at RT under a slight positive pressure of hydrogen (balloon) for 16 h. The reaction was then purged with argon, filtered through Celite, washed with methanol and the solvent was removed *in vacuo*. The crude material was then re-dissolved in chloroform (2.7 mL) and triethylamine (56 μL, 0.403 mmol) added, and stirred at RT for 16 h, then reduced *in vacuo*. Purification by flash column chromatography (SiO_2, 2:1 hexane:ethyl acetate → ethyl acetate → 9:1 ethyl acetate:methanol) afforded the *title compound* as a colourless oil (74 mg, 90%); R_f 0.50 (9:1 ethyl acetate:methanol); ν_{max}/cm^{-1} (thin film) 3316, 1707, 1640, 1557; δ_H (400 MHz, $CDCl_3$) 7.81 (1H, d, J = 7.6 Hz, C**H**), 7.50–7.38 (1H, m, C**H**), 7.29–7.17 (2H, m, 2 × C**H**), 6.09 (1H, br s, N**H**), 4.62 (2H, t, J = 4.6 Hz, C**H**$_2$O), 4.45 (2H, t, J = 4.6 Hz, C**H**$_2$O), 3.39–3.29 (2H, m, C**H**$_2$NH), 2.96 (2H, t, J = 7.6 Hz, C**H**$_2$C), 2.74 (2H, t, J = 4.6 Hz, C**H**$_2$CH$_2$O), 2.58 (2H, t, J = 4.6 Hz, C**H**$_2$CH$_2$O). 1.88–1.72 (2H, m, C**H**$_2$); δ_C (100 MHz, $CDCl_3$) 171.6 (**C**O), 170.2 (**C**O), 168.9 (**C**O), 142.7 (**C**), 132.5 (**C**H), 131.0 (**C**H), 130.8 (**C**H), 129.6 (**C**), 126.3 (**C**H), 61.7 (**C**H$_2$O), 61.4 (**C**H$_2$O), 39.0 (**C**H$_2$NH), 36.7 (**C**H$_2$CO), 35.2 (**C**H$_2$CO), 31.5 (**C**H$_2$), 31.3 (**C**H$_2$); HRMS (ESI): calcd. for $C_{16}H_{19}NNaO_5$, 328.1155. Found: $[MNa]^+$, 328.1158 (−0.7 ppm error).

1,8,12-Trioxa-4-azacyclopentadecane-5,9,13-trione (17c)

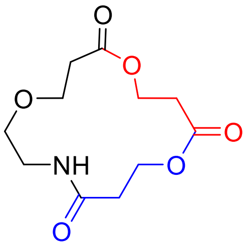

A mixture of 1,8-dioxa-4-azacycloundecane-5,9-dione (49 mg, 0.260 mmol), DMAP (3 mg, 0.0260 mmol) and pyridine (80 μL, 1.60 mmol) in DCM (1.9 mL) under and argon atmosphere was stirred at RT for 30 mins. A solution of acid chloride (0.40 mmol, 1.5 equiv. prepared using the general procedure) in DCM (1.1 mL) was added and the resulting mixture was heated, at reflux, at 50 °C for 16 h. An additional solution of acid chloride (0.730 mmol, 1.5 equiv. prepared using the general procedure) in DCM (1.9 mL) was added and the reaction was heated, at reflux, at 50 °C for another 12 h to achieve reaction completion. The solvent was concentrated *in vacuo,* loaded onto a short silica plug and eluted with 7:3 hexane:ethyl acetate → 1:1 hexane:ethyl acetate to remove the excess carboxylic acid and pyridine. The acylated product was concentrated *in vacuo*, re-dissolved in ethyl acetate (2.7 mL) and placed under an argon atmosphere. Palladium on carbon (26 mg, Pd 10% on carbon) was then added and the reaction vessel was backfilled with hydrogen (*via* balloon) several times. The reaction was stirred at RT under a slight positive pressure of hydrogen (balloon) for 5 h. The reaction was then purged with argon, filtered through Celite, washed with methanol and the solvent was removed *in vacuo*. The crude material was re-dissolved in chloroform (2.7 mL) and triethylamine (70 μL, 0.500 mmol) and stirred for 12 h. After removal of solvent *in vacuo*, the mixture was purified by flash column chromatography (SiO_2, ethyl acetate → 9:1 ethyl acetate/methanol) to afford the *title compound* as a colourless oil (50 mg, 73%); R_f 0.54 (4:1 ethyl acetate:methanol); ν_{max} (thin film)/cm^{-1} 3279, 3087, 2868, 1725, 1642, 1552; δ_H (400 MHz, $CDCl_3$) 6.27 (br s, 1H, NH), 4.43 (2H, t, *J* = 5.3 Hz, OCH_2-CH_2-COO-C**H**$_2$), 4.38 (2H, t, *J* = 5.3 Hz, COO-C**H**$_2$-CH_2-CO-NH), 3.71 (2H, t, *J* = 5.3 Hz, OC**H**$_2$-CH_2-COO), 3.51 (2H, t, *J* = 5.3 Hz, OC**H**$_2$-CH_2-NH), 3.46–3.41 (2H, m, C**H**$_2$-NH), 2.68 (2H, t, *J* = 5.3 Hz, COO-CH_2-C**H**$_2$-COO), 2.59 (2H, t, *J* = 5.3 Hz, COO-CH_2-C**H**$_2$-CO-NH), 2.54 (2H, t, *J* = 5.3 Hz, OCH_2-C**H**$_2$-COO); δ_C (100 MHz, $CDCl_3$) 172.1 (OCH_2-CH_2-**C**OO), 171.2 (**C**OO-CH_2-CH_2-CO-NH), 170.5 (CO-NH), 69.2 (O**C**H_2-CH_2-NH), 66.4 (OC**H**$_2$-CH_2-COO), 61.8 (COO-**C**H_2-CH_2-CO-NH), 60.6 (OCH_2-CH_2-COO-**C**H_2), 39.4 (CH_2-NH), 36.3 (COO-CH_2-**C**H_2-CO-NH), 35.4 (OCH_2-**C**H_2-COO), 34.6 (COO-CH_2-**C**H_2-COO); HRMS (ESI$^+$): calcd. for $C_{11}H_{17}NNaO_6$, 282.0948. Found: [MNa^+], 282.0942 (2.3 ppm error).

(*S*)-Dodecahydropyrrolo[1,2-*d*][1]oxa[4,7]diazacyclopentadecine-1,10,13(12*H*)-trione (17d)

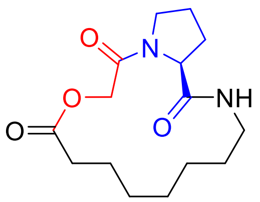

A mixture of 1-oxa-4-azacyclododecane-3,12-dione (80 mg, 0.402 mmol), DMAP (5 mg, 0.0402 mmol) and pyridine (195 µL, 2.41 mmol) in DCM (3 mL) under an argon atmosphere was stirred at RT for 5 mins. Next, a solution of acid chloride (0.602 mmol, 1.50 equiv. prepared using the general procedure) in DCM (1.5 mL) was added and the resulting mixture was heated, at reflux, at 50 °C for 16 h. The solvent was then concentrated *in vacuo*, loaded onto a short silica plug and eluted with 2:1 hexane:ethyl acetate, to remove the majority of excess carboxylic acid and pyridine residues, and concentrated *in vacuo*. This material was re-dissolved in DCM (4.0 mL) and placed under an argon atmosphere. DBU (615 µL, 4.02 mmol) was then added and stirred at RT for 16 h, then reduced *in vacuo*. Purification by flash column chromatography (SiO_2, 2:1 hexane:ethyl acetate → ethyl acetate → 9:1 ethyl acetate:methanol) afforded the *title compound* as a colourless oil (217 mg, 74%); $[\alpha]_D^{25}$ −95.1 (c = 1.0, chloroform); R_f 0.64 (9:1 ethyl acetate:methanol); ν_{max}/cm^{-1} (thin film) 3316, 2856, 1725, 1660, 1544; δ_H (400 MHz, $CDCl_3$) 6.96 (1H, br s, N**H**), 4.88 (1H, d, *J* = 13.3 Hz, C**H**H′O), 4.55 (1H, d, *J* = 6.9 Hz, NC**H**), 4.24 (1H, d, *J* = 13.3 Hz, CH**H′**O), 3.85–3.73 (1H, m, C**H**H′N), 3.46–3.34 (1H, m, CH**H′**N), 3.32–3.20 (1H, m, C**H**H′NH), 3.19–3.04 (1H, m, CH**H′**NH), 2.54–1.10 (16H, m, 8 × C**H**$_2$); δ_C (100 MHz, $CDCl_3$) 174.8 (**C**O), 170.2 (**C**O), 167.4 (**C**O), 61.9 (**C**H$_2$O), 60.4 (**C**HN), 46.8 (**C**H$_2$N), 39.1 (**C**H$_2$NH), 33.7 (**C**H$_2$COO), 28.4 (**C**H$_2$), 28.1 (**C**H$_2$), 27.3 (**C**H$_2$), 26.8 (**C**H$_2$), 25.6 (**C**H$_2$), 24.8 (**C**H$_2$), 23.3 (**C**H$_2$); HRMS (ESI): calcd. for $C_{15}H_{24}N_2NaO_4$, 319.1628. Found: $[MNa]^+$, 319.1633 (−1.5 ppm error).

1,4-Dioxa-8-azacyclopentadecane-3,7,15-trione (17e)

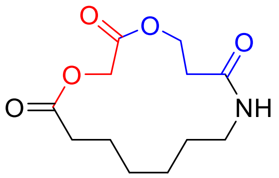

A mixture of 1-oxa-4-azacycloundecane-3,11-dione (50 mg, 0.270 mmol), DMAP (6 mg, 0.0270 mmol) and pyridine (130 µL, 1.62 mmol) in DCM (2 mL) under an argon atmosphere was stirred at RT for 5 mins. Next, a solution of acid chloride (0.405 mmol, 1.50 equiv.

prepared using the general procedure) in DCM (1 mL) was added and the resulting mixture was heated, at reflux, at 50 °C for 16 h. The solvent was concentrated *in vacuo*, loaded onto a short silica plug and eluted with 2:1 hexane:ethyl acetate, to remove the majority of excess carboxylic acid and pyridine residues, and concentrated *in vacuo*. This material was re-dissolved in ethyl acetate (2.7 mL) and placed under an argon atmosphere. Palladium on carbon (27 mg, Pd 10% on carbon) was then added and the reaction vessel was backfilled with hydrogen (via balloon) several times, then stirred at RT under a slight positive pressure of hydrogen (balloon) for 4 h. The reaction was then purged with argon, filtered through Celite, washed with methanol where the solvent was removed *in vacuo*. The crude material was then re-dissolved in chloroform (2.7 mL) and triethylamine (56 µL, 0.405 mmol) added, and stirred at RT for 16 h, then reduced *in vacuo*. Purification by flash column chromatography (SiO_2, 2:1 hexane:ethyl acetate → ethyl acetate) afforded the *title compound* as a white solid (61 mg, 88%); mp. 114–116 °C; R_f 0.26 (ethyl acetate); ν_{max}/cm^{-1} (thin film) 3290, 3087, 2926, 2858, 1732, 1639, 1566; δ_H (400 MHz, $CDCl_3$) 5.77 (1H, br s, N**H**), 4.60 (2H, s, OC**H₂**CO), 4.46 (2H, t, *J* = 5.2 Hz, OC**H**$_2$CH$_2$CO), 3.37–3.26 (2H, m, C**H**$_2$NH), 2.44 (2H, t, *J* = 5.2 Hz, OCH$_2$C**H**$_2$CO), 2.40 (2H, t, *J* = 6.1 Hz, C**H**$_2$CO), 1.73–1.63 (2H, m, C**H**$_2$), 1.60–1.51 (2H, m, C**H**$_2$), 1.44–1.27 (4H, m, 2 × C**H**$_2$); δ_C (100 MHz, $CDCl_3$) 173.2 (**C**O), 169.8 (**C**O), 168.1 (**C**O), 62.2 (**C**H$_2$O), 61.1 (**C**H$_2$O), 39.3 (**C**H$_2$NH), 36.1 (**C**H$_2$CO), 32.6 (**C**O), 28.0 (**C**H$_2$), 27.9 (**C**H$_2$), 25.2 (**C**H$_2$), 23.4 (**C**H$_2$); HRMS (ESI): calcd. for $C_{12}H_{19}NNaO_5$, 280.1155. Found: [MNa]$^+$, 280.1154 (0.4 ppm error).

5-Benzyl-1-oxa-5,13-diazacyclohexadecane-2,6,14-trione (17f)

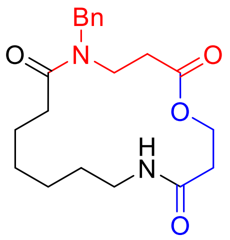

A mixture of 5-benzyl-1,5-diazacyclododecane-2,6-dione (340 mg, 1.179 mmol), DMAP (14 mg, 0.118 mmol) and pyridine (570 µL, 7.07 mmol) in DCM (8 mL) under an argon atmosphere was stirred at RT for 5 mins. Next, a solution of acid chloride (1.77 mmol, 1.50 equiv. prepared using the general procedure) in DCM (4.5 mL) was added and the resulting mixture was heated, at reflux, at 50 °C for 16 h. The solvent was then concentrated *in vacuo*, loaded onto a short silica plug and eluted with 2:1 hexane:ethyl acetate, to remove the majority of excess carboxylic acid and pyridine residues, and concentrated *in vacuo*. This material was re-

dissolved in ethyl acetate (11.8 mL) and placed under an argon atmosphere. Palladium on carbon (118 mg, Pd 10% on carbon) was then added and the reaction vessel was backfilled with hydrogen (via balloon) several times, then stirred at RT under a slight positive pressure of hydrogen (balloon) for 3 h. The reaction was then purged with argon, filtered through Celite, washed with methanol and the solvent was removed *in vacuo*. The crude material was then re-dissolved in chloroform (11.8 mL) and triethylamine (245 µL, 1.77 mmol) added, and stirred at RT for 16 h, then reduced *in vacuo*. Purification by flash column chromatography (SiO_2, 2:1 hexane:ethyl acetate → ethyl acetate → 9:1 ethyl acetate:methanol) afforded the *title compound* (as a mixture of 3 rotamers, comprising one major rotamer and two minor rotamers which could not be accurately measured due to overlap of resonances in ^1H NMR) as a colourless oil (260 mg, 61%); R_f 0.23 (9:1 ethyl acetate:methanol); ν_{max}/cm^{-1} (thin film) 3311, 2932, 2859, 1732, 1631, 1552; δ_H (400 MHz, $CDCl_3$) 7.35–7.01 (15H, m, Ph, all rotamers), 6.42–6.28 (2H, m, N**H**, two rotamers), 6.15 (1H, br s, N**H**, single rotamer), 4.58 (2H, s, C**H₂**Ph, single rotamer), 4.51 (2H, s, C**H₂**Ph, single rotamer), 4.41–4.24 (8H, m, C**H₂**Ph, single rotamer and C**H₂**O, all rotamers), 3.60–3.51 (2H, m, C**H₂**NBn, single rotamer), 3.48–3.40 (2H, m, C**H₂**NBn, single rotamer), 3.35–3.18 (8H, m, C**H₂**NBn, single rotamer and C**H₂**NH, all rotamers), 2.65–2.19 (18H, m, C**H₂**CONBn and C**H₂**COO and C**H₂**CONH, all rotamers), 1.80–1.13 (24H, m, 4 × C**H₂**, all rotamers); δ_C (100 MHz, $CDCl_3$) data for the major rotamer only: 173.7 (**C**O), 170.1 (**C**O), 169.9 (**C**O), 137.4 (**C**), 128.8 (**C**H), 127.9 (**C**H), 126.4 (**C**H), 61.3 (**C**H₂O), 48.8 (**C**H₂NPh), 43.8 (**C**H₂NBn), 38.9 (**C**H₂NH), 35.1 (**C**H₂CO), 33.6 (**C**H₂CO), 31.6 (**C**H₂CO), 29.4 (**C**H₂), 27.82 (**C**H₂), 25.2 (**C**H₂), 25.1 (**C**H₂). Diagnostic ^{13}C NMR resonances for minor rotamers: 174.2 (**C**O), 173.4 (**C**O), 173.0 (**C**O), 170.5 (**C**O), 170.3 (**C**O), 137.2 (**C**), 129.0 (**C**H), 127.6 (**C**H), 61.5 (**C**H₂O), 61.0 (**C**H₂O), 44.0 (**C**H₂NBn), 38.3 (**C**H₂NH), 37.1 (**C**H₂NH), 36.4 (**C**H₂CO), 35.8 (**C**H₂CO), 33.7 (**C**H₂CO), 32.0 (**C**H₂CO), 29.0 (**C**H₂), 27.78 (**C**H₂), 25.6 (**C**H₂), 25.5 (**C**H₂), 25.4 (**C**H₂), 24.6 (**C**H₂), 24.1 (**C**H₂), 24.0 (**C**H₂); HRMS (ESI): calcd. for $C_{20}H_{28}N_2NaO_4$, 383.1941. Found: $[MNa]^+$, 383.1949 (−1.9 ppm error).

1,5-Dioxa-9-azacyclohexadecane-4,8,16-trione (17g)

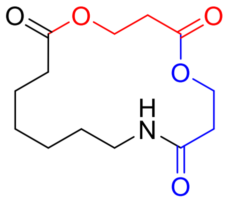

A mixture of 1-oxa-5-azacyclododecane-4,12-dione (60 mg, 0.301 mmol), DMAP (4 mg, 0.0301 mmol) and pyridine (145 µL, 1.81 mmol) in DCM (2.5 mL) under an argon atmosphere

was stirred at RT for 5 mins. Next, a solution of acid chloride (0.452 mmol, 1.50 equiv. prepared using the general procedure) in DCM (1 mL) was added and the resulting mixture was heated, at reflux, at 50 °C for 16 h. An additional solution of acid chloride (0.452 mmol, 1.50 equiv. prepared using the general procedure) in DCM (1 mL) was added and heated, to reflux, at 50 °C for a further 16 h in order to achieve reaction completion. The solvent was then concentrated *in vacuo*, loaded onto a short silica plug and eluted with 2:1 hexane:ethyl acetate, to remove the majority of excess carboxylic acid and pyridine residues, and concentrated *in vacuo*. This material was re-dissolved in ethyl acetate (3.0 mL) and placed under an argon atmosphere. Palladium on carbon (30 mg, Pd 10% on carbon) was then added and the reaction vessel was backfilled with hydrogen (via balloon) several times, then stirred at RT under a slight positive pressure of hydrogen (balloon) for 16 h. The reaction was then purged with argon, filtered through Celite, washed with methanol and the solvent was removed *in vacuo*. The crude material was then re-dissolved in chloroform (3.0 mL) and triethylamine (63 µL, 0.452 mmol) added, and stirred at RT for 16 h, then reduced *in vacuo*. Purification by flash column chromatography (SiO_2, 2:1 hexane:ethyl acetate → ethyl acetate) afforded the *title compound* as a yellow oil (69 mg, 84%); R_f 0.31 (9:1 ethyl acetate:methanol); ν_{max}/cm^{-1} (thin film) 3311, 2932, 1735, 1649, 1552; δ_H (400 MHz, $CDCl_3$) 5.80 (1H, s, N**H**), 4.47–4.32 (4H, m, 2 × C**H**$_2$O), 3.38–3.23 (2H, m, C**H**$_2$NH), 2.65–2.49 (4H, m, OCH$_2$C**H**$_2$CO), 2.38–2.26 (2H, m, C**H**$_2$CO), 1.73–1.63 (2H, m, C**H**$_2$), 1.55–1.43 (2H, m, C**H**$_2$), 1.39–1.18 (4H, m, 2 × C**H**$_2$); δ_C (100 MHz, $CDCl_3$) 173.7 (**C**O), 171.3 (**C**O), 170.0 (**C**O), 61.7 (O**C**H$_2$), 59.6 (O**C**H$_2$), 39.0 (**C**H$_2$NH), 36.5 (**C**H$_2$CO), 34.6 (**C**H$_2$CO), 32.8 (**C**H$_2$CO), 29.3 (**C**H$_2$), 27.6 (**C**H$_2$), 25.1 (**C**H$_2$), 24.2 (**C**H$_2$); HRMS (ESI): calcd. for $C_{13}H_{21}NNaO_5$, 294.1312. Found: $[MNa]^+$, 294.1309 (1.1 ppm error).

14-Benzyl-1-oxa-4,14-diazacycloheptadecane-3,13,17-trione (17h)

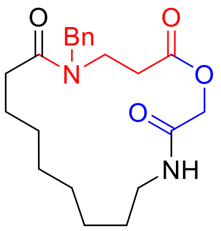

A mixture of 5-benzyl-1,5-diazacyclotetradecane-2,6-dione (65 mg, 0.205 mmol), DMAP (3 mg, 0.0205 mmol) and pyridine (100 µL, 1.23 mmol) in DCM (1.5 mL) under an argon atmosphere was stirred at RT for 5 mins. Next, a solution of acid chloride (0.308 mmol, 1.50 equiv. prepared using the general procedure) in DCM (1 mL) was added and the resulting

mixture was heated, at reflux, at 50 °C for 16 h. An additional solution of acid chloride (0.308 mmol, 1.50 equiv. prepared using the general procedure) in DCM (1 mL) was added and heated, to reflux, at 50 °C for a further 16 h in order to achieve reaction completion. The solvent was then concentrated *in vacuo*, loaded onto a short silica plug and eluted with 2:1 hexane:ethyl acetate, to remove the majority of excess carboxylic acid and pyridine residues, and concentrated *in vacuo*. This material was re-dissolved in THF (2.1 mL) and placed under an argon atmosphere. Palladium on carbon (21 mg, Pd 10% on carbon) and water (370 µL, 20.5 mmol) was then added and the reaction vessel was backfilled with hydrogen (via balloon) several times, then stirred at RT under a slight positive pressure of hydrogen (balloon) for 3 h. The reaction was then purged with argon, filtered through Celite, washed with methanol and the solvent was removed *in vacuo*. The crude material was then re-dissolved in chloroform (2.1 mL) and triethylamine (45 µL, 0.308 mmol) added, and stirred at RT for 16 h, then reduced *in vacuo*. Purification by flash column chromatography (SiO_2, 2:1 hexane:ethyl acetate → ethyl acetate → 9:1 ethyl acetate:methanol) afforded the *title compound* as a colourless oil (75 mg, 98%); R_f 0.43 (ethyl acetate); ν_{max}/cm^{-1} (thin film) 3331, 2930, 2857, 1744, 1634, 1546; δ_H (400 MHz, $CDCl_3$) 7.56 (1H, br s, N**H**), 7.40–7.07 (5H, m, Ph), 4.61 (2H, s, C**H₂**O), 4.59 (2H, s, C**H₂**Ph), 3.72–3.54 (2H, m, C**H₂**NBn), 3.41–3.27 (2H, m, C**H₂**NH), 2.65–2.51 (2H, m, C**H-₂**COO), 2.45–2.28 (2H, m, C**H₂**CONBn), 1.66–1.17 (12H, m, 6 × C**H₂**); δ_C (100 MHz, $CDCl_3$) 174.3 (**C**O), 171.3 (**C**O), 167.7 (**C**O), 136.4 (**C**), 129.1 (**C**H), 127.9 (**C**H), 126.4 (**C**H), 63.3 (**C**H₂O), 52.4 (**C**H₂Ph), 43.3 (**C**H₂NBn), 38.1 (**C**H₂NH), 34.5 (**C**H₂COO), 32.2 (**C**H₂CONBn), 27.9 (**C**H₂), 26.7 (**C**H₂), 26.0 (**C**H₂), 25.5 (**C**H₂), 23.2 (2 × **C**H₂); HRMS (ESI): calcd. for $C_{21}H_{30}N_2NaO_4$, 397.2098. Found: $[MNa]^+$, 397.2108 (−2.6 ppm error).

1,15-Dioxa-4-azacyclooctadecane-3,14,18-trione (17i)

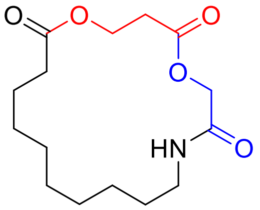

A mixture of 1-oxa-5-azacyclopentadecane-4,15-dione (50 mg, 0.207 mmol), DMAP (3 mg, 0.0207 mmol) and pyridine (100 µL, 1.24 mmol) in DCM (1.5 mL) under an argon atmosphere was stirred at RT for 5 mins. Next, a solution of acid chloride (0.311 mmol, 1.50 equiv. prepared using the general procedure) in DCM (1 mL) was added and the resulting mixture was heated, at reflux, at 50 °C for 16 h. An additional solution of acid chloride (0.311 mmol,

1.50 equiv. prepared using the general procedure) in DCM (1 mL) was added and heated, to reflux, at 50 °C for a further 16 h in order to achieve reaction completion. The solvent was then concentrated *in vacuo*, loaded onto a short silica plug and eluted with 2:1 hexane:ethyl acetate, to remove the majority of excess carboxylic acid and pyridine residues, and concentrated *in vacuo*. This material was re-dissolved in THF (2.1 mL) and placed under an argon atmosphere. Palladium on carbon (21 mg, Pd 10% on carbon) and water (380 µL, 20.7 mmol) was then added and the reaction vessel was backfilled with hydrogen (via balloon) several times, then stirred at RT under a slight positive pressure of hydrogen (balloon) for 16 h. The reaction was then purged with argon, filtered through Celite, washed with methanol and the solvent was removed *in vacuo*. The crude material was then re-dissolved in chloroform (2.1 mL) and triethylamine (45 µL, 0.311 mmol) added, and stirred at RT for 16 h, then reduced *in vacuo*. Purification by flash column chromatography (SiO_2, 2:1 hexane:ethyl acetate → ethyl acetate) afforded the *title compound* as a colourless oil (53 mg, 86%); R_f 0.56 (9:1 ethyl acetate:methanol); ν_{max}/cm^{-1} (thin film) 3313, 2929, 2857, 1733, 1662, 1545; δ_H (400 MHz, $CDCl_3$) 6.28 (1H, br s, N**H**), 4.60 (2H, s, OC**H$_2$**CO), 4.41 (2H, t, *J* = 5.3 Hz, OC**H$_2$**CH_2CO), 3.40–3.29 (2H, m, C**H$_2$**NH), 2.70 (2H, t, *J* = 5.3 Hz, OCH_2C**H$_2$**CO), 2.30 (2H, t, *J* = 6.9 Hz, C**H$_2$**CO), 1.71–1.13 (14H, m, 7 × C**H$_2$**); δ_C (100 MHz, $CDCl_3$) 173.9 (**C**O), 169.7 (**C**O), 166.9 (**C**O), 63.5 (O**C**H_2CO), 59.6 (O**C**H_2CH_2), 39.0 (**C**H_2NH), 34.6 (**C**H_2CO), 33.4 (**C**H_2CO), 28.2 (**C**H_2), 27.1 (2 × **C**H_2), 26.8 (**C**H_2), 26.4 (**C**H_2), 25.4 (**C**H_2), 23.9 (**C**H_2); HRMS (ESI): calcd. for $C_{15}H_{25}NNaO_5$, 322.1625. Found: $[MNa]^+$, 322.1626 (−0.2 ppm error).

1,17-Dioxa-4-azacycloicosane-3,16,20-trione (17j)

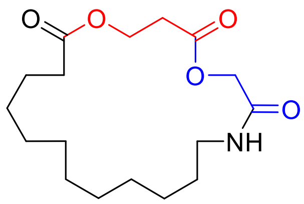

A mixture of 1-oxa-5-azacycloheptadecane-4,17-dione (240 mg, 0.891 mmol), DMAP (11 mg, 0.0891 mmol) and pyridine (430 µL, 5.35 mmol) in DCM (6 mL) under an argon atmosphere was stirred at RT for 5 mins. Next, a solution of acid chloride (1.34 mmol, 1.50 equiv. prepared using the general procedure) in DCM (3.5 mL) was added and the resulting mixture was heated, at reflux, at 50 °C for 16 h. An additional solution of acid chloride (1.34 mmol, 1.50 equiv. prepared using the general procedure) in DCM (3.5 mL) was added and heated, to reflux, at 50 °C for a further 24 h in order to achieve reaction completion. The solvent was then concentrated *in vacuo*, loaded onto a short silica plug and eluted with 2:1 hexane:ethyl acetate, to remove

the majority of excess carboxylic acid and pyridine residues, and concentrated *in vacuo*. This material was re-dissolved in THF (8.9 mL) and placed under an argon atmosphere. Palladium on carbon (89 mg, Pd 10% on carbon) and water (1.60 mL, 89.1 mmol) was then added and the reaction vessel was backfilled with hydrogen (via balloon) several times, then stirred at RT under a slight positive pressure of hydrogen (balloon) for 4 h. The reaction was then purged with argon, filtered through Celite, washed with methanol where the solvent was removed *in vacuo*. The crude material was then re-dissolved in chloroform (8.9 mL) and triethylamine (185 μL, 1.34 mmol) added, and stirred at RT for 16 h, then reduced *in vacuo*. Purification by flash column chromatography (SiO_2, 2:1 hexane:ethyl acetate → ethyl acetate) afforded the *title compound* as a colourless oil (217 mg, 74%); R_f 0.64 (9:1 ethyl acetate:methanol); ν_{max}/cm^{-1} (thin film) 3314, 2927, 2856, 1735, 1662, 1545; δ_H (400 MHz, $CDCl_3$) 6.41 (1H, br s, N**H**), 4.51 (2H, s, OC**H**$_2$CO), 4.35 (2H, t, *J* = 6.0 Hz, OC**H**$_2$CH$_2$), 3.34–3.19 (2H, m, C**H**$_2$NH), 2.66 (2H, t, *J* = 6.0 Hz, CH$_2$C**H**$_2$COO), 2.24 (2H, t, *J* = 6.9 Hz, C**H**$_2$COOCH$_2$CH$_2$), 1.65–1.06 (18H, m, 9 × C**H**$_2$); δ_C (100 MHz, $CDCl_3$) 173.8 (**C**O), 169.3 (**C**O), 166.7 (**C**O), 63.3 (O**C**H$_2$CO), 59.3 (O**C**H$_2$CH$_2$), 38.9 (**C**H$_2$NH), 34.3 (**C**H$_2$CO), 33.8 (**C**H$_2$CO), 28.5 (**C**H$_2$), 27.8 (**C**H$_2$), 27.6 (**C**H$_2$), 27.5 (2 × **C**H$_2$), 27.4 (**C**H$_2$), 26.9 (**C**H$_2$), 25.5 (**C**H$_2$), 24.4 (**C**H$_2$); HRMS (ESI): calcd. for $C_{17}H_{29}NNaO_5$, 250.1938. Found: $[MNa]^+$, 350.1943 (−1.6 ppm error).

(*S*)-Hexadecahydro-16*H*-pyrrolo[1,2-*e*][1]oxa[5,8]diazacycloicosine-1,14,18(17*H*)-trione (17k)

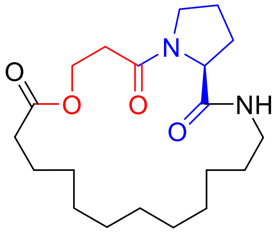

A mixture of 1-oxa-5-azacycloheptadecane-4,17-dione (110 mg, 0.408 mmol), DMAP (5 mg, 0.0408 mmol) and pyridine (0.200 mL, 2.45 mmol) in DCM (3 mL) under an argon atmosphere was stirred at RT for 30 mins. Next, a solution of acid chloride (0.613 mmol, 1.50 eqv prepared using the general procedure) in DCM (2 mL) was added and the resulting mixture was heated, at reflux, at 50 °C for 16 h. An additional solution of acid chloride (0.613 mmol, 1.50 equiv. prepared using the general procedure) in DCM (2 mL) was added and heated, to reflux, at 50 °C for a further 24 h in order to achieve reaction completion. The solvent was then concentrated *in vacuo*, loaded onto a short silica plug and eluted with 2:1 hexane:ethyl acetate, to remove the majority of excess carboxylic acid and pyridine residues, and concentrated *in vacuo*. The

crude material was then re-dissolved in DCM (4.1 mL) and DBU (0.620 mL, 4.083 mmol) was added, followed by stirring at RT for 16 h, before the solvent was removed *in vacuo*. Purification by flash column chromatography (2:1 hexane:ethyl acetate→ethyl acetate→9:1 ethyl acetate:methanol) afforded the *title compound* (as a 4.4:1 mixture of rotamers) as a colourless oil (119 mg, 81%); $[\alpha]_D^{25}$ −43.6 (c = 1.0, $CHCl_3$); R_f 0.20 (ethyl acetate); ν_{max}/cm^{-1} (neat) 3309, 2917, 1739, 1621, 1551; δ_H (400 MHz, $CDCl_3$) 7.18 (1H, br s, N**H**, major rotamer), 6.16 (1H, br s, N**H**, minor rotamer), 4.59 (1H, d, *J* = 8.4 Hz, C**H**, major), 4.42 (1H, d, *J* = 5.3 Hz, C**H**, minor), 4.40–4.27 (4H, m, C**H₂**NCO, both rotamers), 3.63–3.48 (2H, m, C**H**H′N, both), 3.44–3.25 (4H, m, CH**H′**NCO and C**H**H′NH, both), 3.11–2.96 (2H, m, CH**H′**NH), 2.71–2.53 (4H, m, C**H₂**CON, both), 2.52–2.41 (2H, m, C**H**H′CHN, both), 2.33–2.16 (4H, m, C**H₂**COO), 2.15–2.02 (2H, m, CH**H′**CHN, both), 2.03–1.92 (2H, m, C**H**H′CH_2N, both), 1.87–1.72 (2H, m, CH**H′**CH_2N, both), 1.70–1.54 (4H, m, C**H₂**, both), 1.53–1.40 (4H, m, C**H₂**, both), 1.37–1.16 (28H, m, 7 × C**H₂**, both); δ_C (100 MHz, $CDCl_3$) data for the major rotamer only: 174.1 (**C**O), 170.8 (**C**O), 170.4 (**C**O), [59.8, 59.7 (**C**HN and **C**H_2O)], 47.6 (**C**H_2NCO), 39.5 (**C**H_2NH), 33.9 (**C**H_2CO), 33.8 (**C**H_2CO), 28.7 (**C**H_2), 28.1 (**C**H_2), 27.38 (**C**H_2), 27.36 (**C**H_2), 27.3 (**C**H_2), 27.1 (**C**H_2), 26.9 (**C**H_2), 26.7 (**C**H_2), 26.0 (**C**H_2), 25.1 (**C**H_2), 24.2 (**C**H_2). Diagnostic ^{13}C NMR resonances for the minor rotamer: 173.9 (**C**O), 171.4 (**C**O), 169.9 (**C**O), [61.8, 60.2 (**C**HN and **C**H_2O)], 46.9 (**C**H_2NCO), 39.6 (**C**H_2NH), 33.5 (**C**H_2CO), 32.0 (**C**H_2CO), 29.3 (**C**H_2), 28.2 (**C**H_2), 26.5 (**C**H_2), 26.1 (**C**H_2), 22.7 (**C**H_2); HRMS (ESI): calcd. for $C_{20}H_{34}N_2NaO_4$, 389.2411. Found: $[MNa]^+$, 389.2413 (−0.5 ppm error).

5-Benzyl-1-oxa-5,9-diazacyclohenicosane-4,8,21-trione (17l)

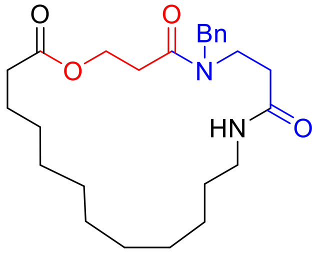

A mixture of 1-oxa-5-azacycloheptadecane-4,17-dione (45 mg, 0.167 mmol), DMAP (2 mg, 0.0167 mmol) and pyridine (81 μL, 1.00 mmol) in DCM (1.5 mL) under an argon atmosphere was stirred at RT for 5 mins. Next, a solution of acid chloride (0.251 mmol, 1.50 equiv. prepared using the general procedure) in DCM (1 mL) was added and the resulting mixture was heated, at reflux, at 50 °C for 16 h. The solvent was then concentrated *in vacuo*, loaded onto a short silica plug and eluted with ethyl acetate, to remove the majority of excess

carboxylic acid and pyridine residues. The crude material was then re-dissolved in DCM (1.67 mL) and DBU (0.250 mL, 1.67 mmol) was added, followed by stirring at RT for 16 h, before the solvent was removed *in vacuo*. Purification by flash column chromatography (2:1 hexane:ethyl acetate→ethyl acetate→9:1 ethyl acetate:methanol) afforded the *title compound* (as a mixture of rotamers) as a colourless oil (58 mg, 84%); R_f 0.13 (ethyl acetate); ν_{max}/cm^{-1} (thin film) 3300, 2927, 2856, 1642, 1554; δ_H (400 MHz, $CDCl_3$) 7.40–7.04 (5H, m, Ph), 6.97 (1H, br s, N**H**), 4.62 (2H, s, C**H**$_2$Ph), 3.76–3.15 (6H, m, 2 × C**H**$_2$N and C**H**$_2$O), 2.62–2.14 (6H, m, 3 × C**H**$_2$CO), 1.94–1.17 (18H, m, 9 × C**H**$_2$); δ_C (100 MHz, $CDCl_3$) 175.2 (**C**O), 172.9 (**C**O), 171.3 (**C**O), 136.6 (**C**), 129.1 (**C**H), 127.2 (**C**H), 126.3 (**C**H), 66.4 (**C**H$_2$O), 54.9 (**C**H$_2$Ph), 52.2 (**C**H$_2$NBn), 42.7 (**C**H$_2$NH), 39.9 (**C**H$_2$CO), 35.6 (**C**H$_2$CO), 32.9 (**C**H$_2$CO), 31.4 (**C**H$_2$), 28.5 (**C**H$_2$), 27.6 (**C**H$_2$), 27.2 (**C**H$_2$), 26.6 (**C**H$_2$), 25.6 (**C**H$_2$), 25.1 (**C**H$_2$), 24.8 (**C**H$_2$), 24.5 (**C**H$_2$); HRMS (ESI): calcd. for $C_{25}H_{39}N_2O_4$, 431.2904. Found: $[MH]^+$, 431.2912 (0.1 ppm error).

1,7,11,15-Tetraoxa-4-azacyclooctadecane-3,10,14,18-tetraone (18a)

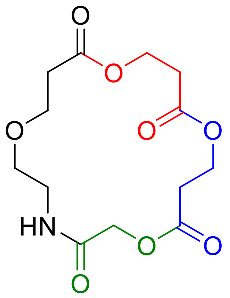

A mixture of 1,8,12-trioxa-4-azacyclopentadecane-5,9,13-trione (35 mg, 0.133 mmol), DMAP (2 mg, 0.0133 mmol) and pyridine (0.064 mL, 0.79 mmol) in DCM (1.0 mL) under an argon atmosphere was stirred at RT for 30 mins. A solution of acid chloride (0.199 mmol, 1.5 equiv. prepared using the general procedure) in DCM (1.0 mL) was then added and the resulting mixture was heated, at reflux, at 50 °C for 16 h. A further three portions of acid chloride (0.131 mmol, 1.5 equiv. prepared using the general procedure) in DCM (0.5 mL) were added at 48 h, 72 h and 96 h. The solvent was concentrated *in vacuo*, loaded onto a short silica plug and eluted with 3:2 hexane:ethyl acetate →1:4 hexane:ethyl acetate to remove the excess carboxylic acid and pyridine. 12 mg of starting material was recovered. The acylated product was concentrated *in vacuo*, re-dissolved in THF (1.5 mL) and placed under an argon atmosphere. Palladium on carbon (13 mg, Pd 10% on carbon) and water (0.24 mL, 13.3 mmol) was added and the reaction vessel was backfilled with hydrogen (*via* balloon) several times. The reaction was stirred at RT under a slight positive pressure of hydrogen (balloon) overnight. The reaction was then purged with argon, filtered through Celite, washed with methanol and the solvent was removed *in*

vacuo. The crude material was re-dissolved in chloroform (1.5 mL) and triethylamine (29 μL, 0.21 mmol) and stirred at RT for 12 h, then concentrate *in vacuo*. Purification by flash column chromatography (SiO_2, ethyl acetate → 9:1 ethyl acetate:methanol) afforded the *title compound* as a colourless oil (15 mg, 36% or 54% brsm); R_f 0.55 (4:1 ethyl acetate:methanol); ν_{max} (thin film)/cm^{-1} 3382, 2926, 1736, 1677, 1542; δ_H (400 MHz, $CDCl_3$) 6.93 (1H, br s, NH), 4.66 (2H, s, C**H**$_2$-CO-NH), 4.49 (2H, t, *J* = 5.3 Hz, COO-C**H**$_2$-CH$_2$-COO), 4.41 (2H, t, *J* = 5.5 Hz, OCH$_2$-CH$_2$-COO-C**H**$_2$), 3.72 (2H, t, *J* = 5.3 Hz, OC**H**$_2$-CH$_2$-COO), 3.56−3.54 (2H, m, OC**H**$_2$-CH$_2$-NH), 3.50−3.46 (2H, m, CH$_2$-NH), 2.73 (2H, t, *J* = 5.3 Hz, C**H**$_2$-COO-CH$_2$-CO-NH), 2.60 (2H, t, *J* = 5.3 Hz, C**H**$_2$-COO-CH$_2$-CH$_2$-COO), 2.53 (2H, t, *J* = 5.3 Hz, OCH$_2$-C**H**$_2$-COO); δ_C (100 MHz, $CDCl_3$) 172.0 (OCH$_2$-CH$_2$-**C**OO), 171.0 (**C**OO-CH$_2$-CH$_2$-COO), 169.6 (**C**OO-CH$_2$-CO-NH), 167.2 (CO-NH), 69.2 (O**C**H$_2$-CH$_2$-NH), 66.4 (O**C**H$_2$-CH$_2$-COO), 63.5 (**C**H$_2$-CO-NH), 60.2 (OCH$_2$-CH$_2$-COO-**C**H$_2$), 60.0 (COO-**C**H$_2$-CH$_2$), 39.3 (CH$_2$-NH), 35.1 (OCH$_2$-C**H**$_2$-COO), 35.0 (C**H**$_2$-COO-CH$_2$-CH$_2$-COO), 34.6 (**C**H$_2$-COO-CH$_2$-CO-NH); HRMS (ESI$^+$): calcd. for $C_{13}H_{19}NNaO_8$, 340.1003. Found: [MNa$^+$], 340.1007 (−1.2 ppm error).

1,12,16-Trioxa-4-azacyclononadecane-3,11,15,19-tetraone (18b)

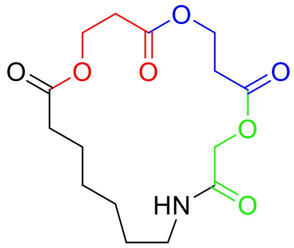

A mixture of 1,5-dioxa-9-azacyclohexadecane-4,8,16-trione (50 mg, 0.184 mmol), DMAP (2 mg, 0.0184 mmol) and pyridine (90 μL, 1.10 mmol) in DCM (2 mL) under an argon atmosphere was stirred at RT for 5 mins. Next, a solution of acid chloride (0.276 mmol, 1.50 equiv. prepared using the general procedure) in DCM (1 mL) was added and the resulting mixture was heated, at reflux, at 50 °C for 16 h. An additional solution of acid chloride (0.276 mmol, 1.50 equiv. prepared using the general procedure) in DCM (1 mL) was added and heated, to reflux, at 50 °C for a further 16 h in order to achieve reaction completion. The solvent was then concentrated *in vacuo*, loaded onto a short silica plug and eluted with ethyl acetate, to remove the majority of excess carboxylic acid and pyridine residues, and concentrated *in vacuo*. This material was re-dissolved in THF (1.8 mL) and placed under an argon atmosphere. Palladium on carbon (18 mg, Pd 10% on carbon) and water (330 μL, 18.4 mmol) was then added and the reaction vessel was backfilled with hydrogen (via balloon) several times, then stirred at RT under a slight positive pressure of hydrogen (balloon) for 4 h. The reaction was

then purged with argon, filtered through Celite, washed with methanol and the solvent was removed *in vacuo*. The crude material was then re-dissolved in chloroform (1.8 mL) and triethylamine (40 µL, 0.276 mmol) added, and stirred at RT for 16 h, then reduced *in vacuo*. Purification by flash column chromatography (SiO_2, 2:1 hexane:ethyl acetate→ethyl acetate) afforded the *title compound* as a colourless oil (51 mg, 84%); R_f 0.60 (9:1 ethyl acetate:methanol); ν_{max}/cm^{-1} (thin film) 3385, 2935, 2860, 1732, 1667, 1543; δ_H (400 MHz, $CDCl_3$) 6.61 (1H, br s, N**H**), 4.59 (2H, s,OC**H₂**CO), 4.48 (2H, t, *J* = 5.3 Hz, C**H₂**O), 4.32 (2H, t, *J* = 5.3 Hz, C**H₂**O), 3.36–3.27 (2H, m, C**H₂**NH), 2.72 (2H, t, *J* = 5.3 Hz, C**H₂**CH₂O), 2.61 (2H, t, *J* = 5.3 Hz, C**H₂**CH₂O), 2.29 (2H, t, *J* = 6.1 Hz, C**H₂**COO), 1.66–1.57 (2H, m, C**H₂**), 1.56–1.48 (2H, m, C**H₂**), 1.36–1.25 (4H, m, 2 × C**H₂**); δ_C (100 MHz, $CDCl_3$) 173.5 (**C**O), 171.3 (**C**O), 169.4 (**C**O), 167.0 (**C**O), 63.5 (**C**H₂O), 60.3 (**C**H₂O), 59.6 (**C**H₂O), 38.2 (**C**H₂NH), 34.9 (**C**H₂CO), 34.2 (**C**H₂CO), 33.8 (**C**H₂CO), 28.3 (**C**H₂), 27.3 (**C**H₂), 25.2 (**C**H₂), 24.5 (**C**H₂); HRMS (ESI): calcd. for $C_{15}H_{23}NNaO_7$, 352.1367. Found: $[MNa]^+$, 352.1363 (0.7 ppm error).

12-Benzyl-1,16-dioxa-4,12-diazacyclononadecane-3,11,15,19-tetraone (18c)

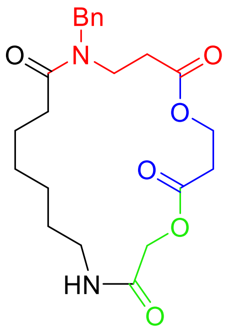

A mixture of 5-benzyl-1-oxa-5,13-diazacyclohexadecane-2,6,14-trione (90 mg, 0.250 mmol), DMAP (3 mg, 0.0250 mmol) and pyridine (121 µL, 1.50 mmol) in DCM (2.0 mL) under an argon atmosphere was stirred at RT for 5 mins. Next, a solution of acid chloride (0.375 mmol, 1.50 equiv. prepared using the general procedure) in DCM (1 mL) was added and the resulting mixture was heated, at reflux, at 50 °C for 16 h. The solvent was then concentrated *in vacuo*, loaded onto a short silica plug and eluted with 2:1 hexane:ethyl acetate, to remove the majority of excess carboxylic acid and pyridine residues, and concentrated *in vacuo*. This material was re-dissolved in THF (2.5 mL) and placed under an argon atmosphere. Palladium on carbon (25 mg, Pd 10% on carbon) and water (450 µL, 25 mmol) was then added and the reaction vessel was backfilled with hydrogen (via balloon) several times, then stirred at RT under a slight positive pressure of hydrogen (balloon) for 3 h. The reaction was then purged with argon, filtered through Celite, washed with methanol and the solvent was removed *in vacuo*. The crude

material was then re-dissolved in chloroform (2.5 mL) and triethylamine (53 μL, 0.375 mmol) added, and stirred at RT for 16 h, then reduced *in vacuo*. Purification by flash column chromatography (SiO_2, 2:1 hexane:ethyl acetate → ethyl acetate → 9:1 ethyl acetate:methanol) afforded the *title compound* (as a 1:1:1.4:2.1 {A:B:C:D} mixture of rotamers) as a yellow oil (71 mg, 68%); R_f 0.18 (ethyl acetate); ν_{max}/cm^{-1} (thin film) 3312, 2931, 2858, 1735, 1636, 1548; δ_H (400 MHz, $CDCl_3$) 7.50–7.08 (20H, m, Ph, all rotamers), 7.05 (1H, br s, N**H**, rotamer A), 6.37 (1H, br s, N**H**, rotamer B), 6.09 (1H, br s, N**H**, rotamer C), 5.73 (1H, br s, N**H**, rotamer D), 4.67–4.54 (8H, m, NC**H₂**Ph, all rotamers), 4.49–4.33 (8H, m, CH_2C**H₂**O, all rotamers), 3.73–3.23 (24H, m, C**H₂**NBn and C**H₂**NH and C**H₂**O, all rotamers), 2.80–2.27 (24H, m, 3 × C**H₂**CO, all rotamers), 1.82–1.20 (32H, m, 4 × C**H₂**, all rotamers); δ_C (100 MHz, $CDCl_3$) 174.22 (**C**O), 174.16 (**C**O), 173.6 (**C**O), 173.4 (**C**O), 173.0 (**C**O), 172.0 (**C**O), 171.0 (**C**O), 170.6 (**C**O), 170.2 (**C**O), 169.7 (**C**O), 169.4 (**C**O), 167.3 (**C**O), 166.9 (**C**O), 137.5 (**C**), 137.4 (**C**), 137.2 (**C**), 136.7 (**C**), 129.1 (**C**H), 129.0 (**C**H), 128.8 (**C**H), 128.0 (**C**H), 127.9 (**C**H), 127.7–127.6 (3 × **C**H), 126.5 (**C**H), 126.4 (**C**H), 63.5 (**C**H₂), 63.2 (**C**H₂), 61.6 (**C**H₂), 61.3 (**C**H₂), 60.2 (**C**H₂), 59.9 (**C**H₂), 53.7 (**C**H₂O), 51.6 (**C**H₂O), 48.9 (**C**H₂), 48.3 (**C**H₂O), 44.1 (**C**H₂NBn), 43.8 (**C**H₂NBn), 42.9 (**C**H₂NBn), 42.2 (**C**H₂NBn), 39.1 (**C**H₂NH), 39.0 (**C**H₂NH), 38.9 (**C**H₂NH), 37.5 (**C**H₂CO), 36.6 (**C**H₂CO), 36.0 (**C**H₂CO), 35.2 (**C**H₂CO), 34.5 (**C**H₂CO), 34.0 (**C**H₂CO), 33.8 (**C**H₂CO), 33.3 (**C**H₂CO), 32.5 (**C**H₂CO), 32.14 (**C**H₂CO), 32.09 (**C**H₂CO), 31.7 (**C**H₂CO), 29.5 (**C**H₂), 29.0 (**C**H₂), 28.7 (**C**H₂), 27.92 (**C**H₂), 27.89 (**C**H₂), 27.85 (**C**H₂), 27.81 (**C**H₂), 26.4 (**C**H₂), 25.7 (**C**H₂), 25.5 (**C**H₂), 25.2 (**C**H₂), 25.0 (**C**H₂), 24.62 (**C**H₂), 24.56 (**C**H₂), 24.4 (**C**H₂), 24.0 (**C**H₂); HRMS (ESI): calcd. for $C_{22}H_{30}N_2NaO_6$, 441.1996. Found: $[MNa]^+$, 441.1994 (1.0 ppm error).

1,5,9-Trioxa-13-azacycloicosane-4,8,12,20-tetraone (18d)

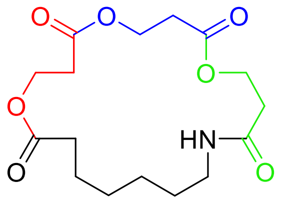

A mixture of 1,5-dioxa-9-azacyclohexadecane-4,8,16-trione (50 mg, 0.184 mmol), DMAP (2 mg, 0.0184 mmol) and pyridine (90 μL, 1.10 mmol) in DCM (2 mL) under an argon atmosphere was stirred at RT for 5 mins. Next, a solution of acid chloride (0.276 mmol, 1.50 equiv. prepared using the general procedure) in DCM (1 mL) was added and the resulting mixture was heated, at reflux, at 50 °C for 16 h. An additional solution of acid chloride (0.276 mmol, 1.50 equiv. prepared using the general procedure) in DCM (1 mL) was added and heated, to

reflux, at 50 °C for a further 16 h in order to achieve reaction completion. The solvent was then concentrated *in vacuo*, loaded onto a short silica plug and eluted with ethyl acetate, to remove the majority of excess carboxylic acid and pyridine residues, and concentrated *in vacuo*. This material was re-dissolved in ethyl acetate (1.8 mL) and placed under an argon atmosphere. Palladium on carbon (18 mg, Pd 10% on carbon) was then added and the reaction vessel was backfilled with hydrogen (via balloon) several times, then stirred at RT under a slight positive pressure of hydrogen (balloon) for 3 h. The reaction was then purged with argon, filtered through Celite, washed with methanol and the solvent was removed *in vacuo*. The crude material was then re-dissolved in chloroform (1.8 mL) and triethylamine (40 µL, 0.276 mmol) added, and stirred at RT for 16 h, then reduced *in vacuo*. Purification by flash column chromatography (SiO_2, ethyl acetate→9:1 ethyl acetate:methanol) afforded the *title compound* as a white solid (49 mg, 79%); mp. 121–123 °C; R_f 0.50 (9:1 ethyl acetate:methanol); ν_{max}/cm^{-1} (thin film) 3301, 2932, 2858, 1732, 1646, 1551; δ_H (400 MHz, $CDCl_3$) 5.86 (1H, br s, N**H**), 4.49–4.27 (6H, m, 3 × C**H₂**O), 3.36–3.23 (2H, m, C**H₂**NH), 2.68–2.58 (4H, m, 2 × C**H₂**COO), 2.44 (2H, t, *J* = 5.3 Hz, C**H₂**CONH), 2.28 (2H, t, *J* = 6.9 Hz, C**H₂**COO), 1.61–1.53 (2H, m, C**H₂**), 1.52–1.43 (2H, m, C**H₂**), 1.37–1.26 (4H, m, 2 × C**H₂**); δ_C (100 MHz, $CDCl_3$) 173.3 (**C**O), 170.8 (**C**O), 169.93 (**C**O), 169.91 (**C**O), 61.4 (**C**H_2O), 60.0 (**C**H_2O), 59.9 (**C**H_2O), 39.3 (**C**H_2NH), 36.4 (**C**H_2CO), 34.7 (**C**H_2CO), 34.2 (**C**H_2CO), 33.9 (**C**H_2CO), 29.2 (**C**H_2), 28.7 (**C**H_2), 26.4 (**C**H_2), 25.2 (**C**H_2); HRMS (ESI): calcd. for $C_{16}H_{25}NNaO_7$, 366.1523. Found: $[MNa]^+$, 366.1521 (1.0 ppm error).

Procedure for one single the synthesis of 18d from 4f with one chromatographic purification

A mixture of azocan-2-one **4f** (100 mg, 0.786 mmol), DMAP (10 mg, 0.0786 mmol) and pyridine (380 µL, 4.72 mmol) in DCM (5.5 mL) under an argon atmosphere was stirred at RT for 5 mins. Next, a solution of acid chloride (2.36 mmol, 3.0 equiv. prepared using the general procedure) in DCM (3 mL) was added and the resulting mixture was heated, at reflux, at 50 °C for 16 h. The mixture was then diluted with diethyl ether (30 mL) and washed with 10% aq. HCl (15 mL). The aqueous layer was then extracted with diethyl ether (3 × 20 mL) and washed with sat. aq. $NaHCO_3$ (10 mL). The combined organic extracts dried over $MgSO_4$ and concentrated *in vacuo*. The crude material was then re-dissolved in ethyl acetate (7.8 mL) and placed under an argon atmosphere. Palladium on carbon (78 mg, Pd 10% on carbon) was then added and the reaction vessel was backfilled with hydrogen (via balloon) several times, then

stirred at RT under a slight positive pressure of hydrogen (balloon) for 4 h. The reaction was then purged with argon, filtered through Celite, washed with ethyl acetate and the solvent was removed *in vacuo*. The crude material was then re-dissolved in chloroform (7.8 mL) and triethylamine (164 µL, 1.18 mmol) added, stirred at RT for 16 h, and the solvent was removed *in vacuo*. The residue was dissolved in DCM (30 mL), washed with sat. aq. $NaHCO_3$ (10 mL) and brine (10 mL), dried over $MgSO_4$ and the solvent removed *in vacuo*. The crude material was added to DMAP (10 mg, 0.0786 mmol) and pyridine (380 µL, 4.72 mmol) in DCM (5.5 mL) under an argon atmosphere was stirred at RT for 5 mins. Next, a solution of acid chloride (2.36 mmol, 3.0 equiv. prepared using the general procedure) in DCM (3 mL) was added and the resulting mixture was heated, at reflux, at 50 °C for 16 h. The mixture was then diluted with diethyl ether (30 mL) and washed with 10% aq. HCl (15 mL). The aqueous layer was then extracted with diethyl ether (3 × 20 mL) and washed with sat. aq. $NaHCO_3$ (10 mL). The combined organic extracts dried over $MgSO_4$ and concentrated *in vacuo*. The crude material was then re-dissolved in ethyl acetate (7.8 mL) and placed under an argon atmosphere. Palladium on carbon (78 mg, Pd 10% on carbon) was then added and the reaction vessel was backfilled with hydrogen (via balloon) several times, then stirred at RT under a slight positive pressure of hydrogen (balloon) for 16 h. The reaction was then purged with argon, filtered through Celite, washed with ethyl acetate and the solvent was removed *in vacuo*. The crude material was then re-dissolved in chloroform (7.8 mL) and triethylamine (164 µL, 1.18 mmol) added, stirred at RT for 16 h, and the solvent was removed *in vacuo*. The residue was dissolved in DCM (30 mL), washed with sat. aq. $NaHCO_3$ (10 mL) and brine (10 mL), dried over $MgSO_4$ and the solvent removed *in vacuo*. The crude material was added to DMAP (10 mg, 0.0786 mmol) and pyridine (380 µL, 4.72 mmol) in DCM (5.5 mL) under an argon atmosphere was stirred at RT for 5 mins. Next, a solution of acid chloride (2.36 mmol, 3.0 equiv. prepared using the general procedure) in DCM (3 mL) was added and the resulting mixture was heated, at reflux, at 50 °C for 16 h. An additional solution of acid chloride (2.36 mmol, 3.0 equiv. prepared using the general procedure) in DCM (3 mL) was added and heated, to reflux, at 50 °C for a further 16 h in order to achieve reaction completion. The mixture was then diluted with DCM (30 mL) and washed with 10% aq. HCl (15 mL). The aqueous layer was then extracted with DCM (3 × 30 mL) and washed with sat. aq. $NaHCO_3$ (10 mL). The combined organic extracts dried over $MgSO_4$ and concentrated *in vacuo*. The crude material was then re-dissolved in ethyl acetate (7.8 mL) and placed under an argon atmosphere. Palladium on carbon (78 mg, Pd 10% on carbon) was then added and the reaction vessel was backfilled with hydrogen (via balloon) several times, then stirred at RT under a slight positive pressure of

hydrogen (balloon) for 16 h. The reaction was then purged with argon, filtered through Celite, washed with ethyl acetate and the solvent was removed *in vacuo*. The crude material was then re-dissolved in chloroform (7.8 mL) and triethylamine (164 µL, 1.18 mmol) added, stirred at RT for 16 h, and concentrated *in vacuo*. Purification by flash column chromatography (SiO_2, 2:1 hexane:ethyl acetate → ethyl acetate→9:1 ethyl acetate:methanol) afforded the *title compound* **18d** as a white solid (130 mg, 48%); R_f 0.50 (9:1 ethyl acetate:methanol); δ_H (400 MHz, $CDCl_3$) 5.87 (1H, br s, N**H**), 4.49–4.27 (6H, m, 3 × C**H**$_2$O), 3.36–3.23 (2H, m, C**H**$_2$NH), 2.68–2.58 (4H, m, 2 × C**H**$_2$COO), 2.44 (2H, t, *J* = 5.3 Hz, C**H**$_2$CONH), 2.28 (2H, t, *J* = 6.9 Hz, C**H**$_2$COO), 1.76–1.18 (8H, m, 4 × C**H**$_2$). Data consistent with those reported above.

17,21-Dibenzyl-1-oxa-4,17,21-triazacyclotetracosane-3,16,20,24-tetraone (18e)

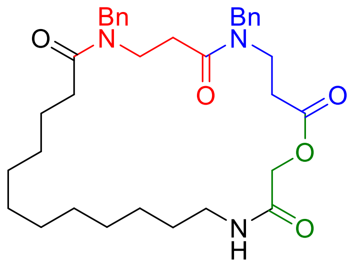

A mixture of 5,9-dibenzyl-1,5,9-triazacyclohenicosane-2,6,10-trione (50 mg, 0.0962 mmol), DMAP (1 mg, 9.62×10^{-3} mmol) and pyridine (46 µL, 0.577 mmol) in DCM (1 mL) under an argon atmosphere was stirred at RT for 5 mins. Next, a solution of acid chloride (0.144 mmol, 1.50 equiv. prepared using the general procedure) in DCM (1 mL) was added and the resulting mixture was heated, at reflux, at 50 °C for 16 h. An additional solution of acid chloride (0.144 mmol, 1.50 equiv. prepared using the general procedure) in DCM (1 mL) was added and heated, to reflux, at 50 °C for a further 16 h in order to achieve reaction completion. The solvent was then concentrated *in vacuo*, loaded onto a short silica plug and eluted with ethyl acetate, to remove the majority of excess carboxylic acid and pyridine residues, and concentrated *in vacuo*. This material was re-dissolved in THF (1.0 mL) and placed under an argon atmosphere. Palladium on carbon (18 mg, Pd 10% on carbon) and water (173 µL) was then added and the reaction vessel was backfilled with hydrogen (via balloon) several times, then stirred at RT under a slight positive pressure of hydrogen (balloon) for 5 h. The reaction was then purged with argon, filtered through Celite, washed with ethyl acetate and the solvent was removed *in vacuo*. The crude material was then re-dissolved in chloroform (1.0 mL) and triethylamine (20 µL, 0.144 mmol) added, and stirred at RT for 16 h, then reduced *in vacuo*. Purification by flash column chromatography (SiO_2, 2:1 hexane:ethyl acetate→ethyl

acetate→9:1 ethyl acetate:methanol) afforded the *title compound* (as a mixture of 2 rotamers) as a yellow oil (38 mg, 68%). The number of rotamers was determined by number of carbonyl resonances in the ^{13}C NMR spectrum; R_f 0.18 (ethyl acetate); ν_{max}/cm^{-1} (thin film) 3313, 2927, 2855, 1731, 1637, 1452; δ_H (400 MHz, $CDCl_3$) 7.47–6.93 (22H, m, 2 × Ph and N**H**, both rotamers), 4.76–4.05 (12H, m, C**H₂**O and 2 × NC**H₂**Ph, both), 3.73–3.48 (8H, m, 2 × C**H₂**NBn, both), 3.36–3.10 (4H, m, C**H₂**NH, both), 2.79–2.13 (12H, m, 3 × C**H₂**CO, both), 1.87–0.72 (36H, m, 9 × C**H₂**, both); δ_C (100 MHz, $CDCl_3$) 174.7 (**C**O), 174.5 (**C**O), 172.4 (**C**O), 172.1 (**C**O), 171.9 (**C**O), 171.4 (**C**O), 171.2 (**C**O), 170.4 (**C**O), 137.7 (**C**), 137.6 (**C**), 136.8 (**C**), 136.69 (**C**), 136.65 (**C**), 136.6 (**C**), 136.1 (**C**), 129.3 (**C**H), 129.1 (**C**H), 129.0 (**C**H), 128.7 (**C**H), 128.3 (**C**H), 128.23 (**C**H), 128.19 (**C**H), 128.0 (**C**H), 127.9 (**C**H), 127.7 (**C**H), 127.5 (**C**H), 126.6 (**C**H), 126.43 (**C**H), 126.37 (**C**H), 126.0 (**C**H), 73.5 (**C**H_2O), 66.9 (**C**H_2O), 52.6 (**C**H_2Ph), 52.5 (**C**H_2Ph), 52.0 (**C**H_2Ph), 51.3 (**C**H_2Ph), 49.4 (N**C**$H_2$$CH_2$), 48.6 (N**C**$H_2$$CH_2$), 45.5 (N**C**$H_2$$CH_2$), 45.3 (N**C**$H_2$$CH_2$), 44.0 (**C**$H_2$NH), 42.6 (**C**$H_2$NH), 39.7 (**C**$H_2$CO), 39.6 (**C**$H_2$CO), 39.4 (**C**$H_2$CO), 36.7 (**C**$H_2$CO), 35.5 (**C**$H_2$CO), 35.3 (**C**$H_2$CO), 33.3 (**C**$H_2$), 32.9 (**C**$H_2$), 32.3 (**C**$H_2$), 31.7 (**C**$H_2$), 31.2 (**C**$H_2$), 28.9 (**C**$H_2$), 28.7 (**C**$H_2$), 28.6 (**C**$H_2$), 28.3 (**C**$H_2$), 28.1 (**C**$H_2$), 27.8 (**C**$H_2$), 27.7 (**C**$H_2$), 27.64 (**C**$H_2$), 27.57 (**C**$H_2$), 27.4 (**C**$H_2$), 27.3 (**C**$H_2$), 27.1 (**C**$H_2$), 26.9 (**C**$H_2$), 26.4 (**C**$H_2$), 26.1 (**C**$H_2$), 26.0 (**C**$H_2$), 25.4 (**C**$H_2$), 24.9 (**C**$H_2$), 24.6 (**C**$H_2$), 24.5 (**C**$H_2$); HRMS (ESI): calcd. for $C_{34}H_{47}N_3NaO_5$, 600.3408. Found: $[MNa]^+$, 600.3419 (0.9 ppm error).

Compound 6b

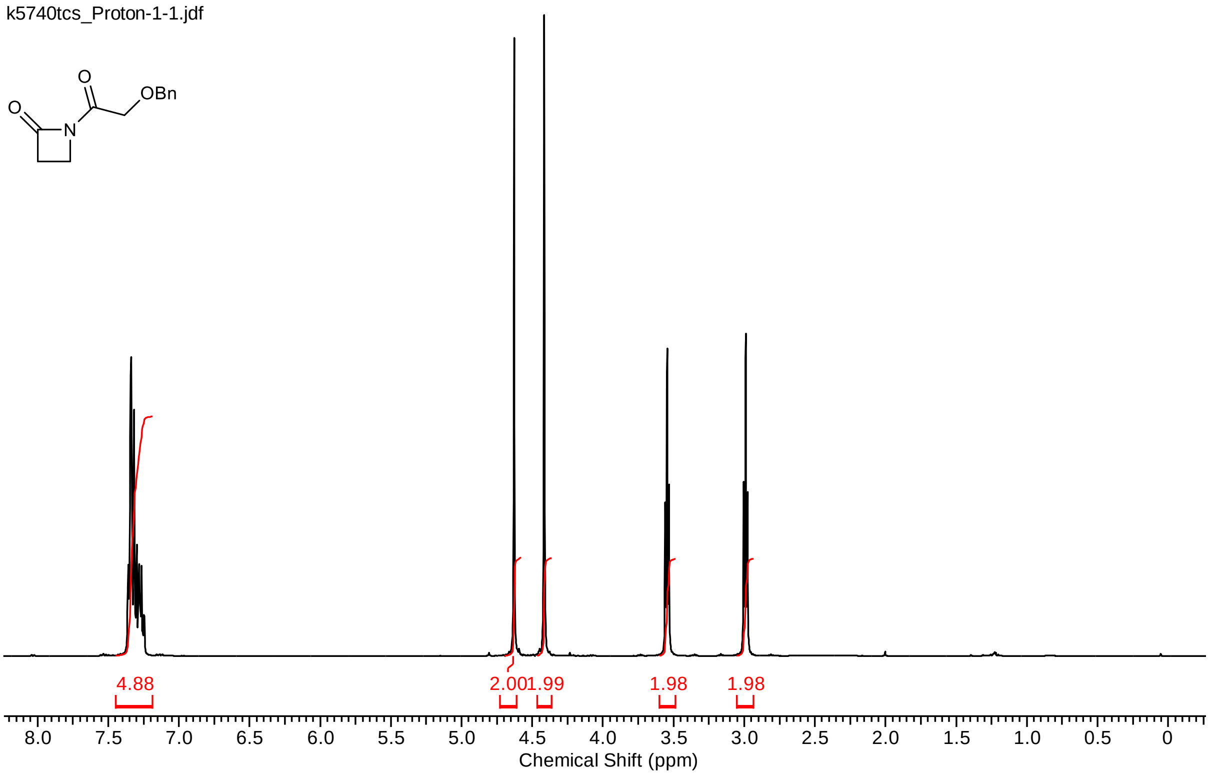

k5740tcs_Proton-1-1.jdf
O
OBn
O
N
4.88
2.00
1.99
1.98
1.98
8.0
7.5
7.0
6.5
6.0
5.5
5.0
4.5
4.0
3.5
3.0
2.5
2.0
1.5
1.0
0.5
0
Chemical Shift (ppm)

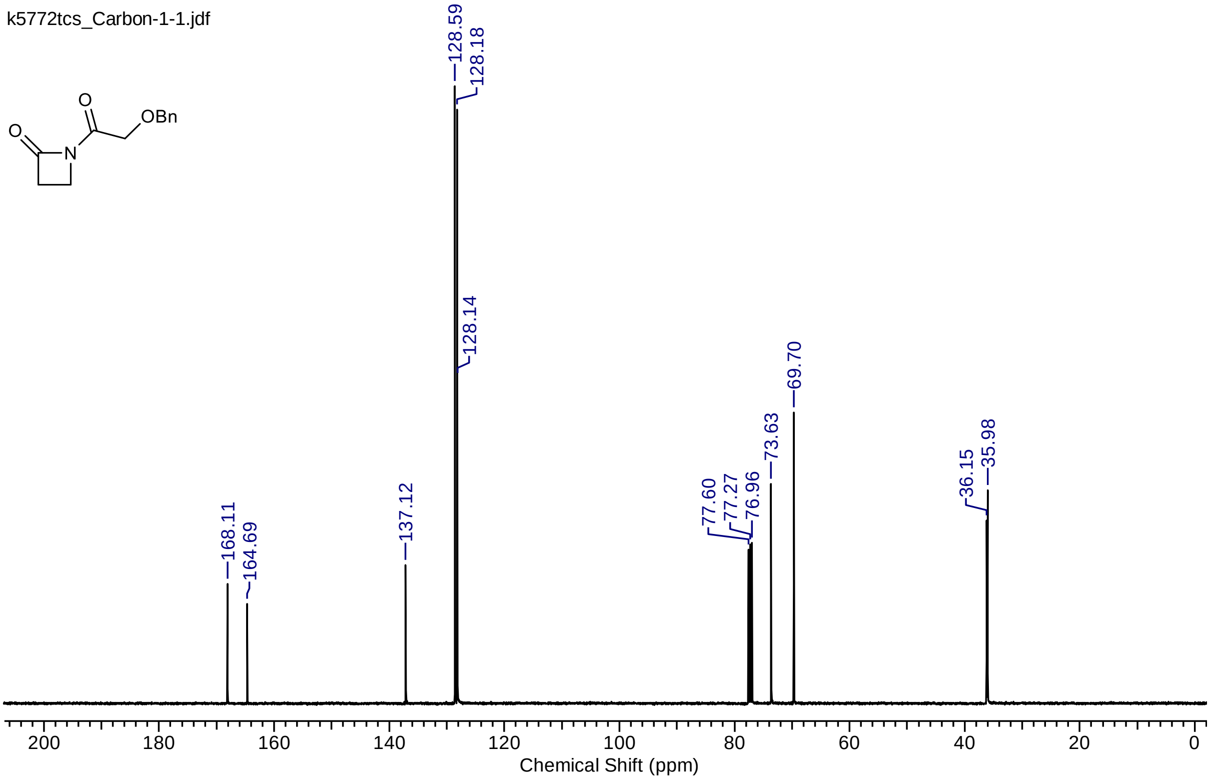

k5772tcs_Carbon-1-1.jdf
O
OBn
O
N
128.59
128.18
128.14
69.70
73.63
77.60
77.27
76.96
36.15
35.98
137.12
168.11
164.69
200
180
160
140
120
100
80
60
40
20
0
Chemical Shift (ppm)

Compound 6c[5]

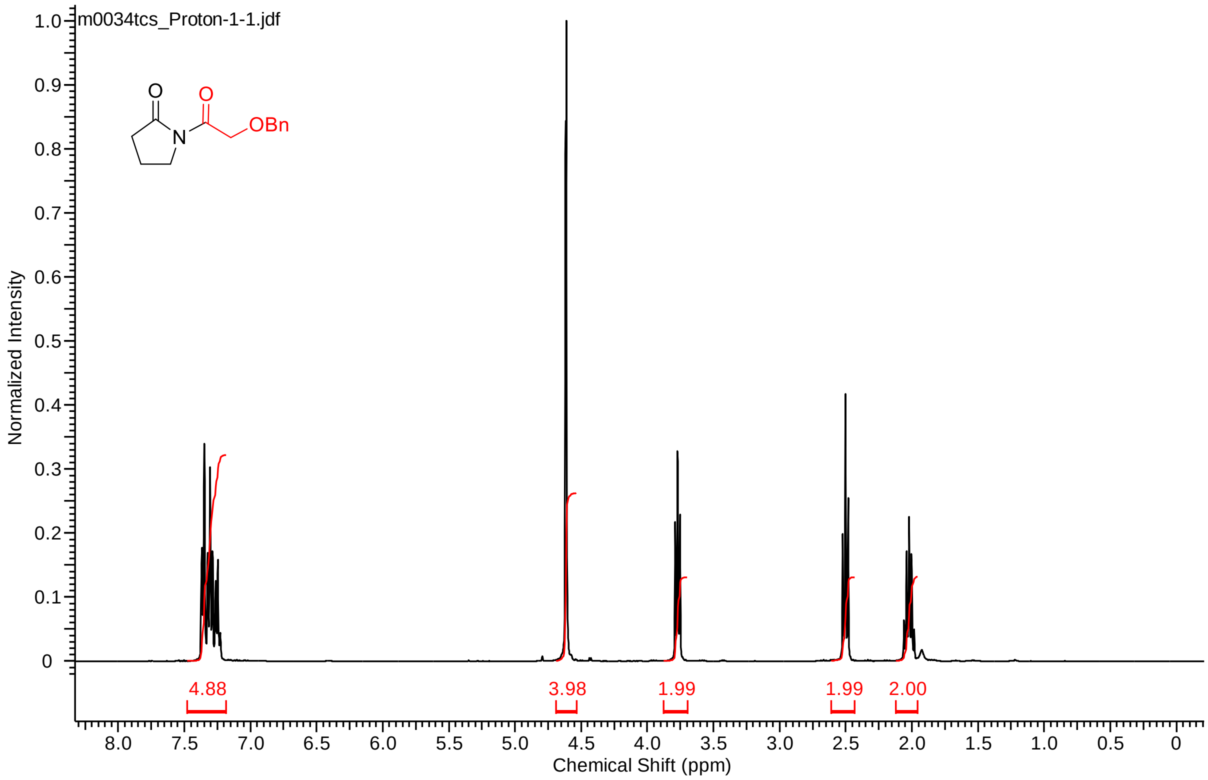

Compound 6d

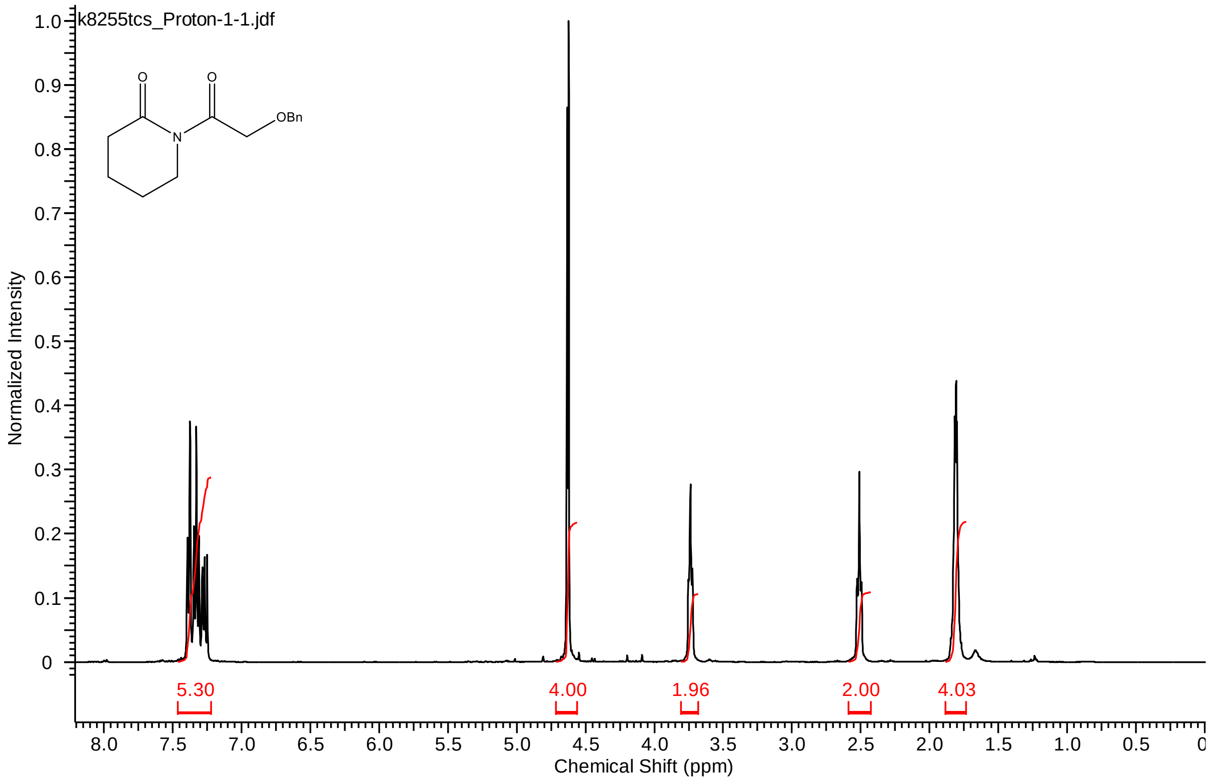
k8255tcs_Proton-1-1.jdf
O
O
N
OBn
Normalized Intensity
1.0
0.9
0.8
0.7
0.6
0.5
0.4
0.3
0.2
0.1
0
5.30
4.00
1.96
2.00
4.03
8.0 7.5 7.0 6.5 6.0 5.5 5.0 4.5 4.0 3.5 3.0 2.5 2.0 1.5 1.0 0.5 0
Chemical Shift (ppm)

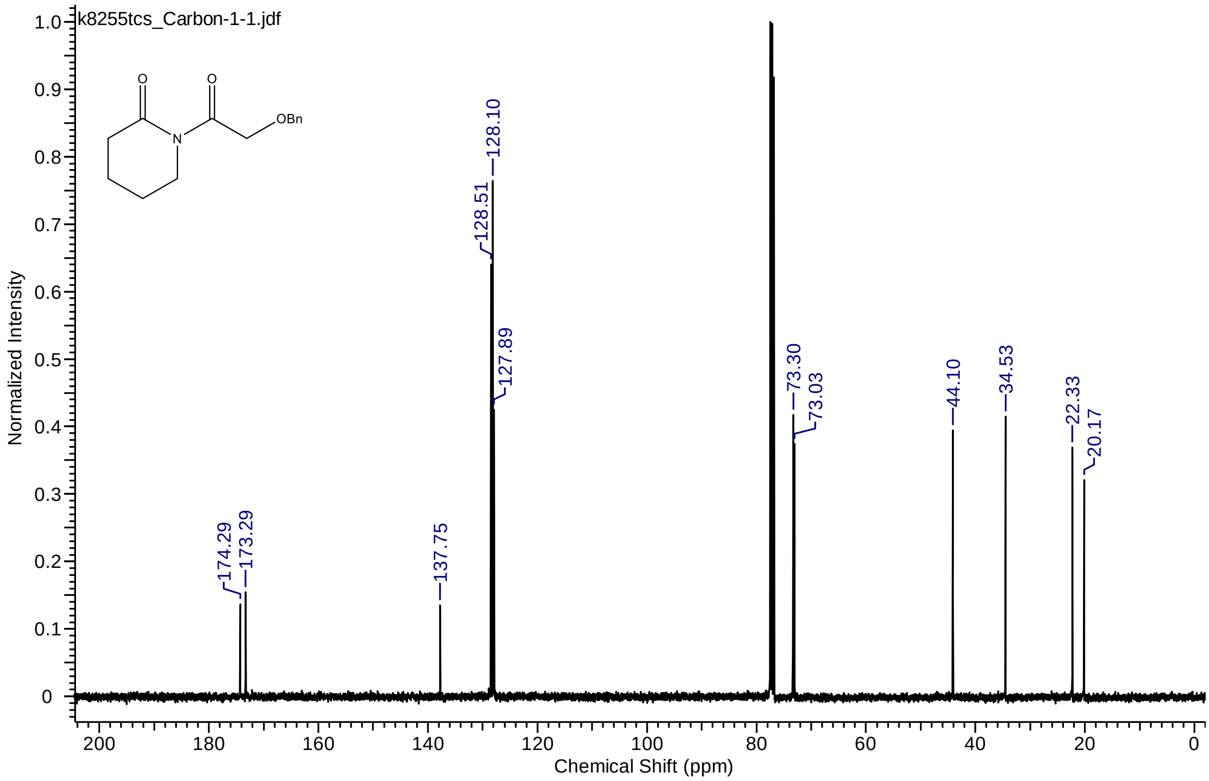
k8255tcs_Carbon-1-1.jdf
O
O
N
OBn
Normalized Intensity
1.0
0.9
0.8
0.7
0.6
0.5
0.4
0.3
0.2
0.1
0
174.29
173.29
137.75
128.51
128.10
127.89
73.30
73.03
44.10
34.53
22.33
20.17
200 180 160 140 120 100 80 60 40 20 0
Chemical Shift (ppm)

Compound 6e

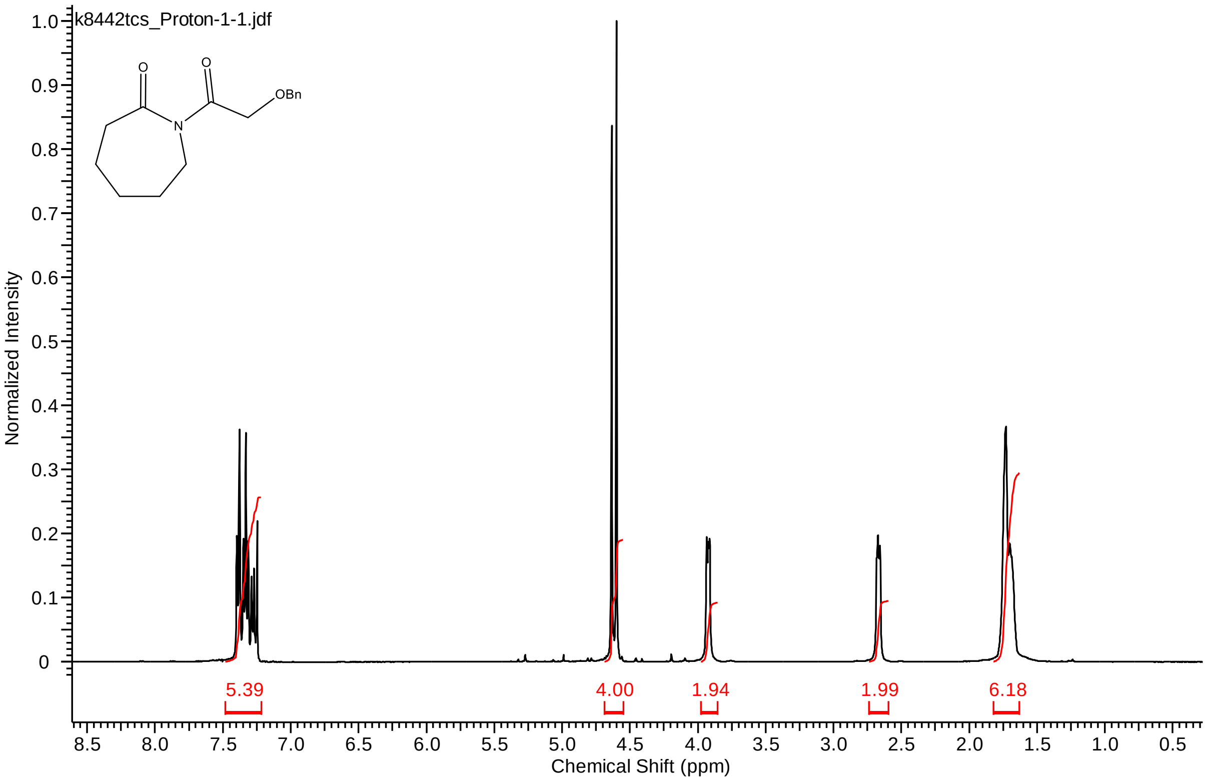

k8442tcs_Proton-1-1.jdf
O
O
N
OBn
Normalized Intensity
1.0
0.9
0.8
0.7
0.6
0.5
0.4
0.3
0.2
0.1
0
5.39
4.00
1.94
1.99
6.18
8.5 8.0 7.5 7.0 6.5 6.0 5.5 5.0 4.5 4.0 3.5 3.0 2.5 2.0 1.5 1.0 0.5
Chemical Shift (ppm)

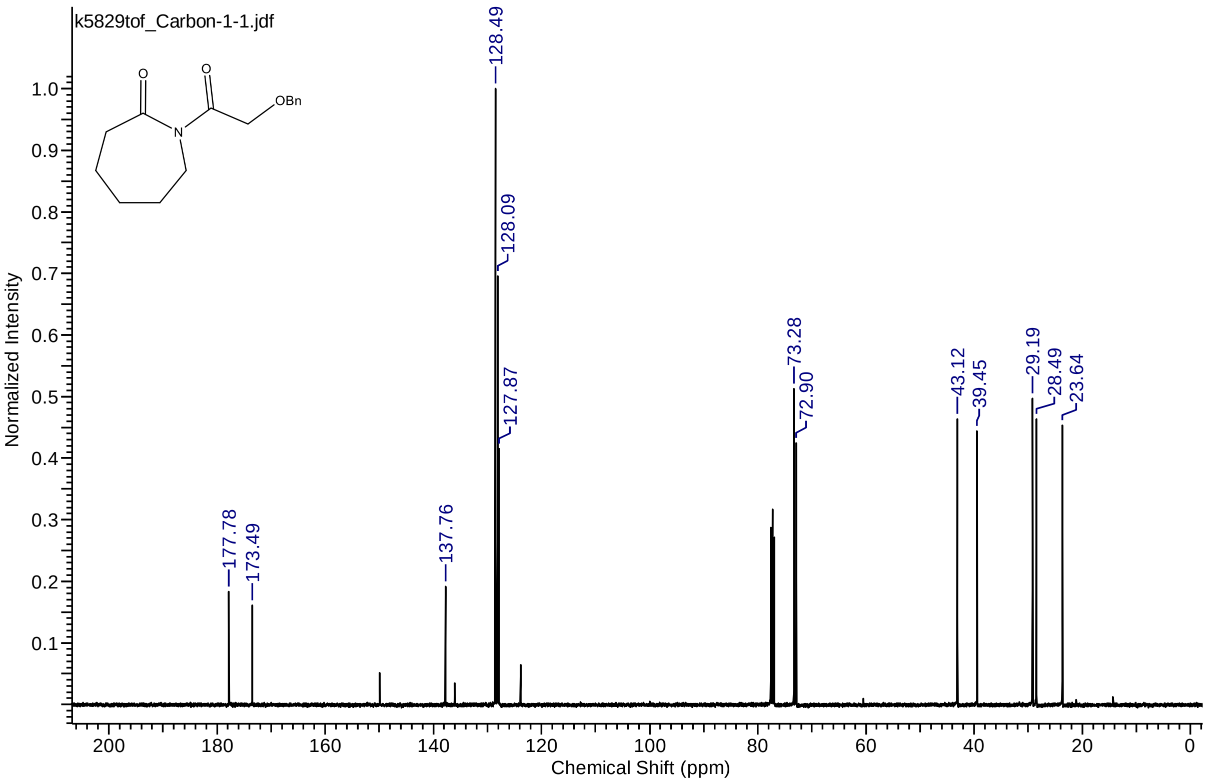

k5829tof_Carbon-1-1.jdf
O
O
N
OBn
Normalized Intensity
1.0
0.9
0.8
0.7
0.6
0.5
0.4
0.3
0.2
0.1
177.78
173.49
137.76
128.49
128.09
127.87
73.28
72.90
43.12
39.45
29.19
28.49
23.64
200 180 160 140 120 100 80 60 40 20 0
Chemical Shift (ppm)

Compound 6f

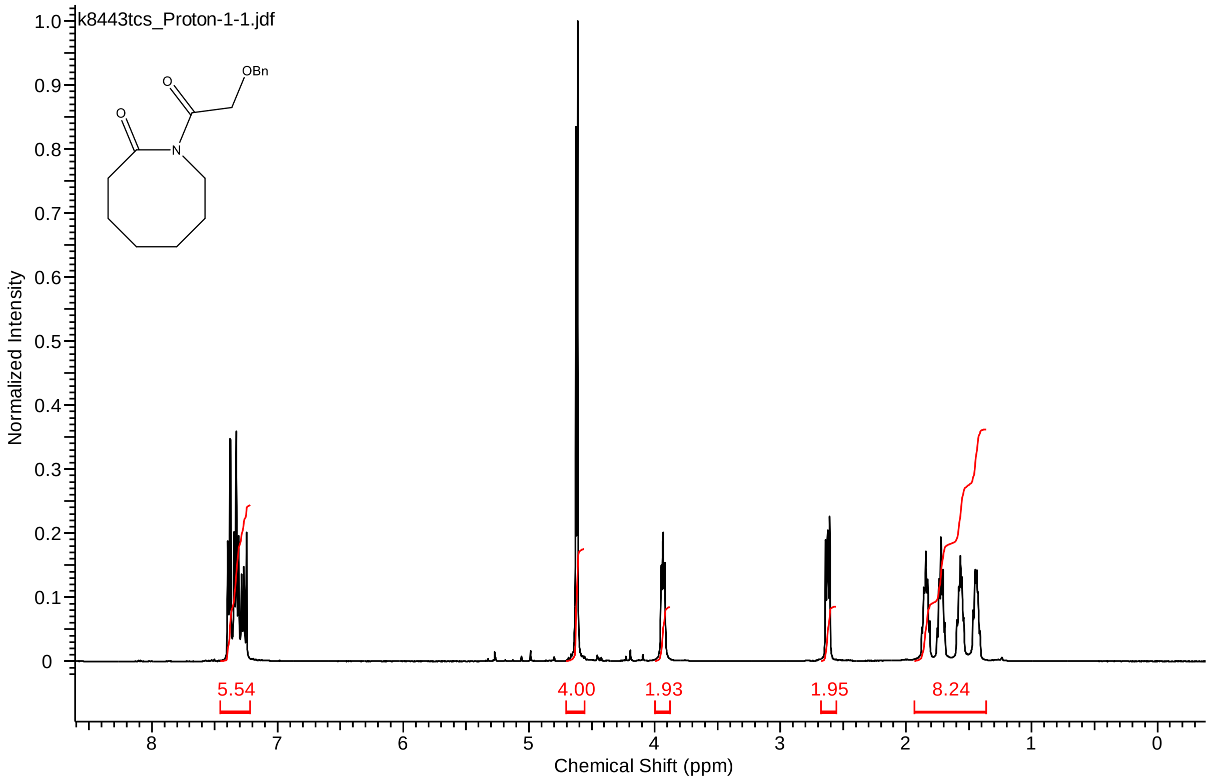

k8443tcs_Proton-1-1.jdf
OBn
O
O
N
Normalized Intensity
5.54
4.00
1.93
1.95
8.24
Chemical Shift (ppm)

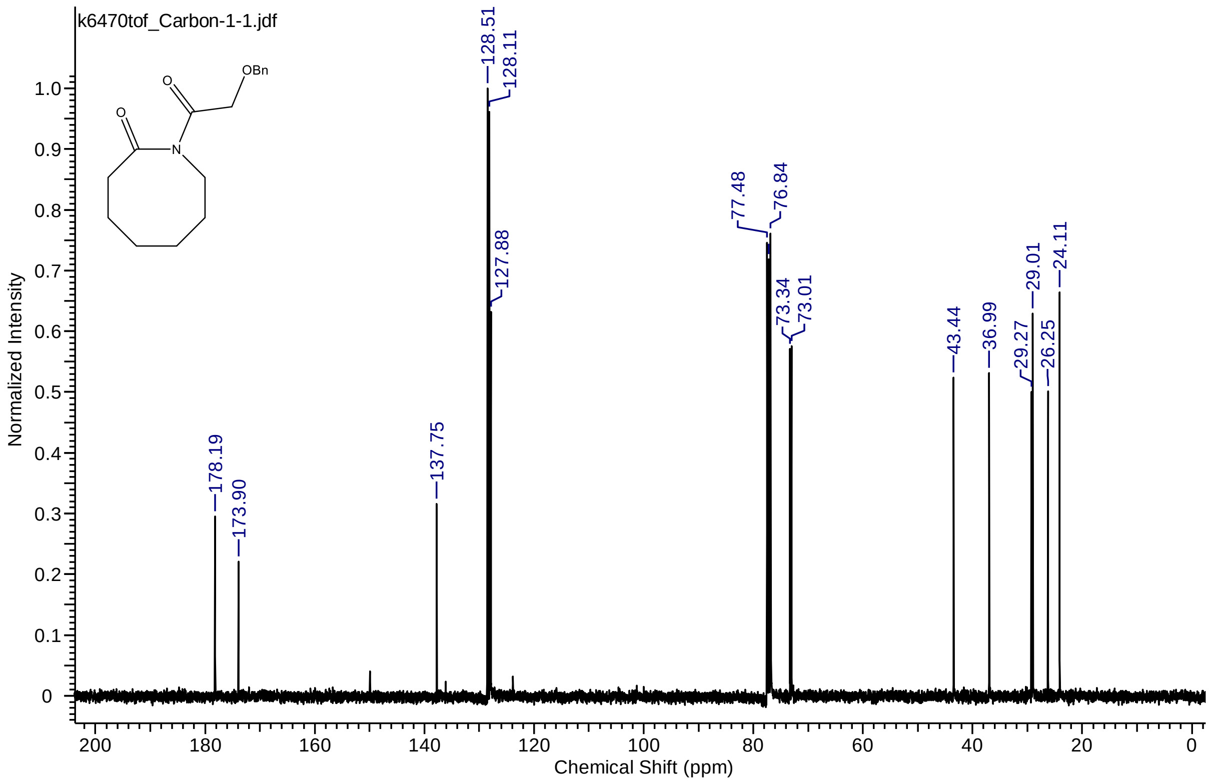

k6470tof_Carbon-1-1.jdf
OBn
O
O
N
Normalized Intensity
178.19
173.90
137.75
128.51
128.11
127.88
77.48
76.84
73.34
73.01
43.44
36.99
29.27
29.01
26.25
24.11
Chemical Shift (ppm)

Compound 6g

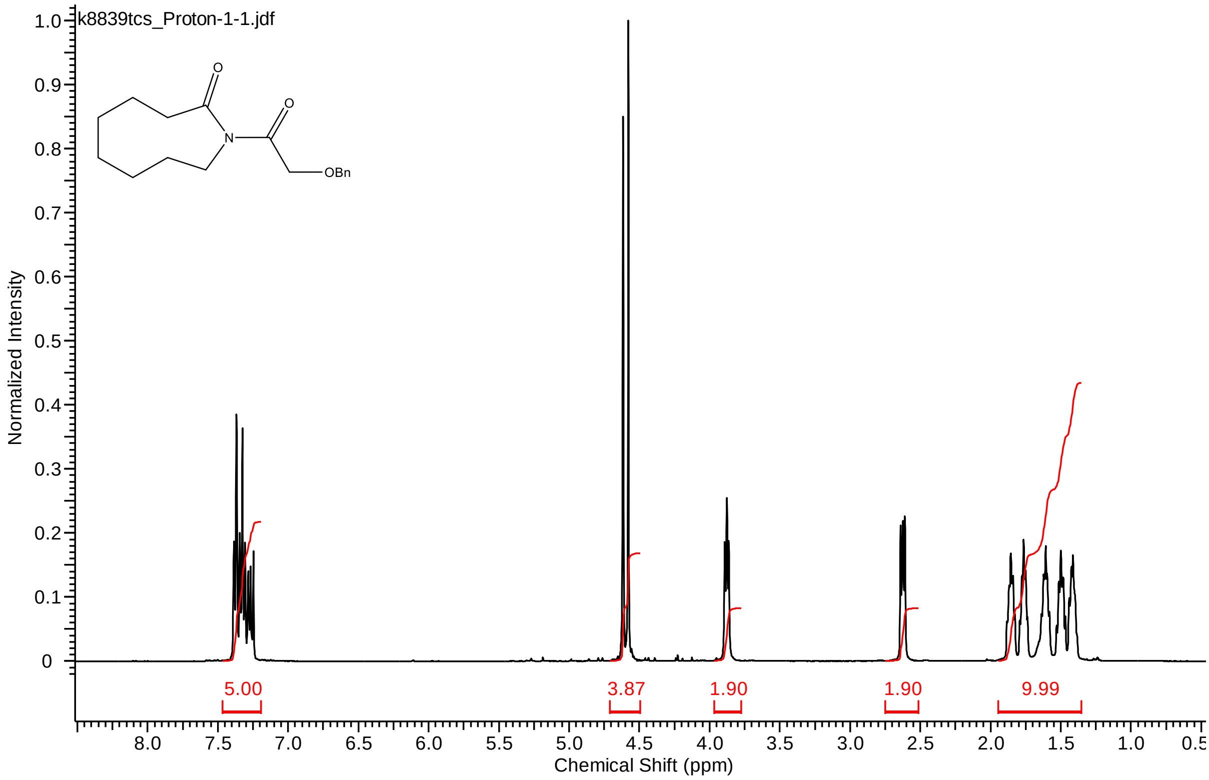
k8839tcs_Proton-1-1.jdf
O
O
N
OBn
Normalized Intensity
5.00
3.87
1.90
1.90
9.99
Chemical Shift (ppm)

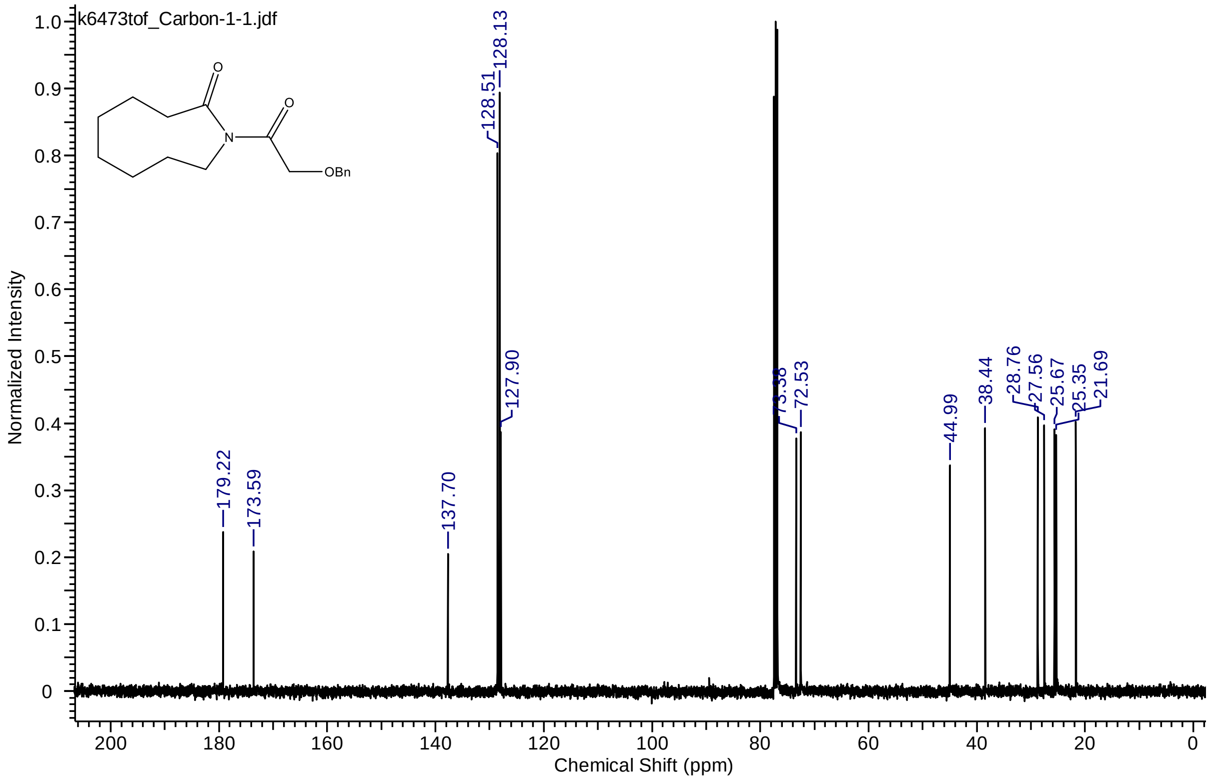
k6473tof_Carbon-1-1.jdf
O
O
N
OBn
Normalized Intensity
179.22
173.59
137.70
128.51
128.13
127.90
73.58
72.53
44.99
38.44
28.76
27.56
25.67
25.35
21.69
Chemical Shift (ppm)

Compound 6h

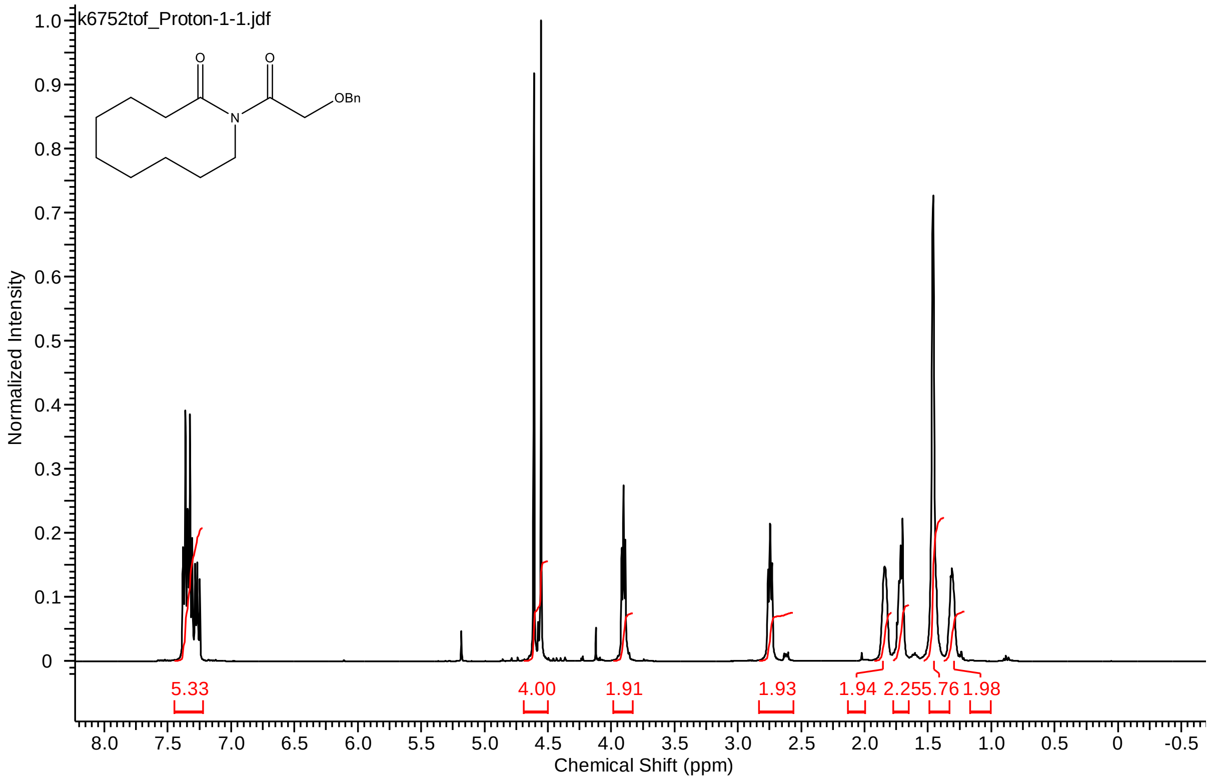
k6752tof_Proton-1-1.jdf
O
O
N
OBn
Normalized Intensity
5.33
4.00
1.91
1.93
1.94
2.25
5.76
1.98
Chemical Shift (ppm)

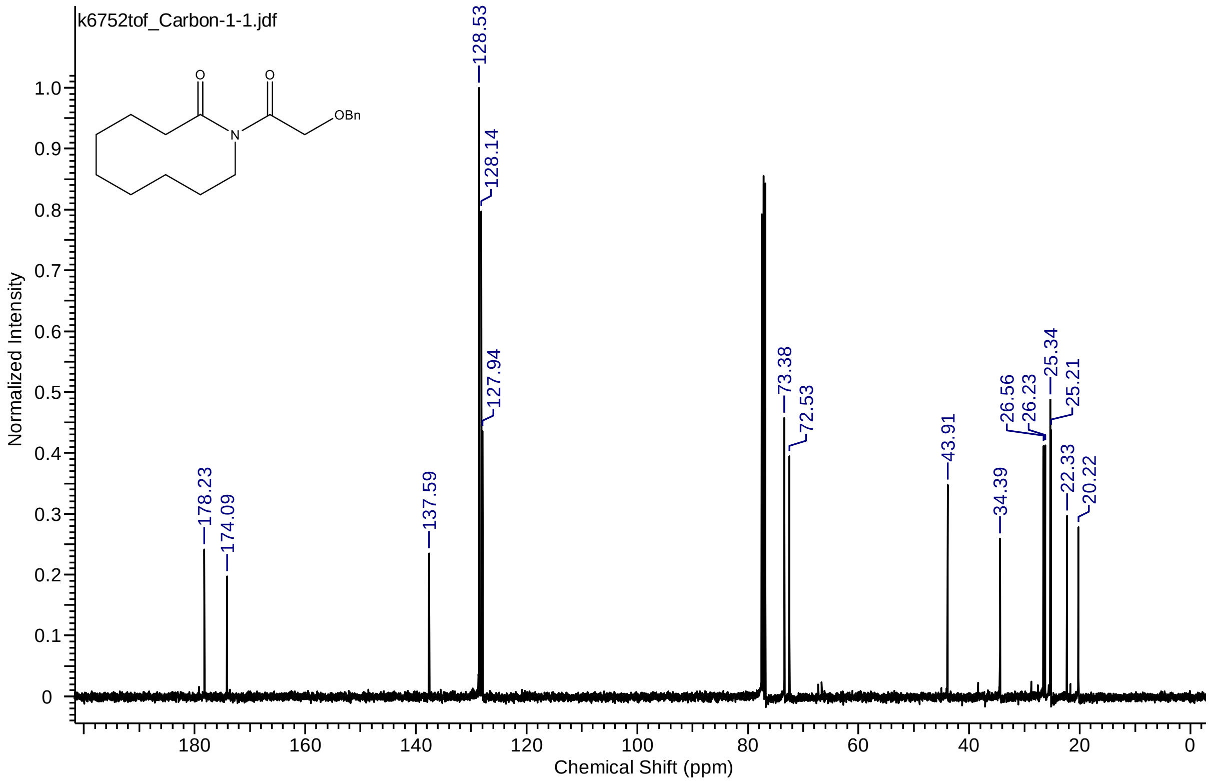
k6752tof_Carbon-1-1.jdf
O
O
N
OBn
Normalized Intensity
178.23
174.09
137.59
128.53
128.14
127.94
73.38
72.53
43.91
34.39
26.56
26.23
25.34
25.21
22.33
20.22
Chemical Shift (ppm)

Compound 7a

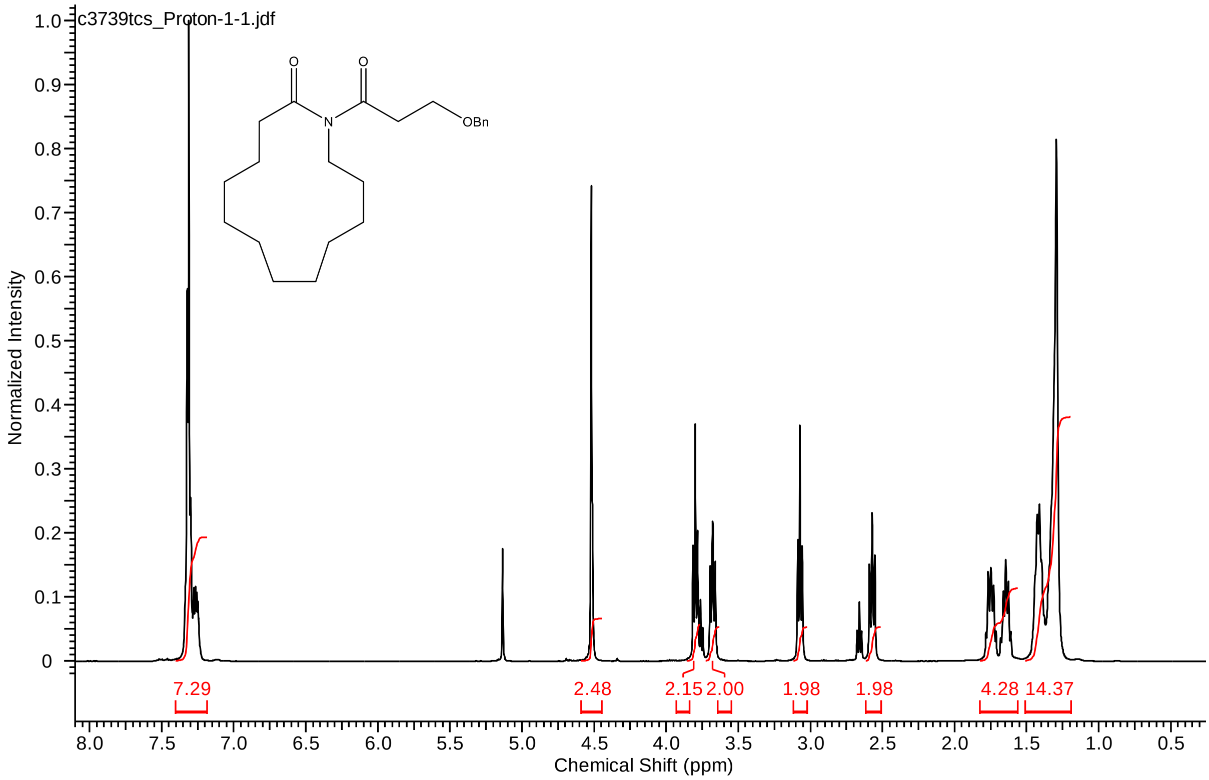
c3739tcs_Proton-1-1.jdf
O
O
N
OBn
Normalized Intensity
7.29
2.48
2.15
2.00
1.98
1.98
4.28
14.37
Chemical Shift (ppm)

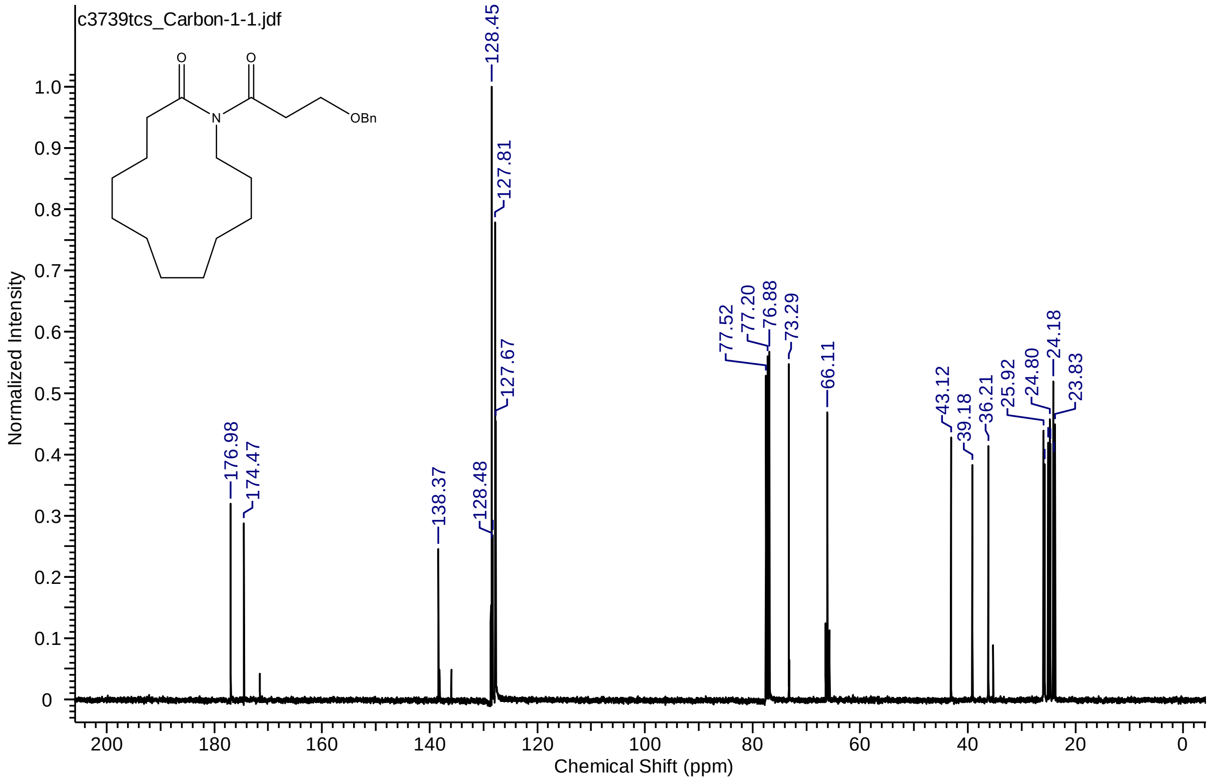
c3739tcs_Carbon-1-1.jdf
O
O
N
OBn
Normalized Intensity
176.98
174.47
138.37
128.48
128.45
127.81
127.67
77.52
77.20
76.88
73.29
66.11
43.12
39.18
36.21
25.92
24.80
24.18
23.83
Chemical Shift (ppm)

Compound 7b

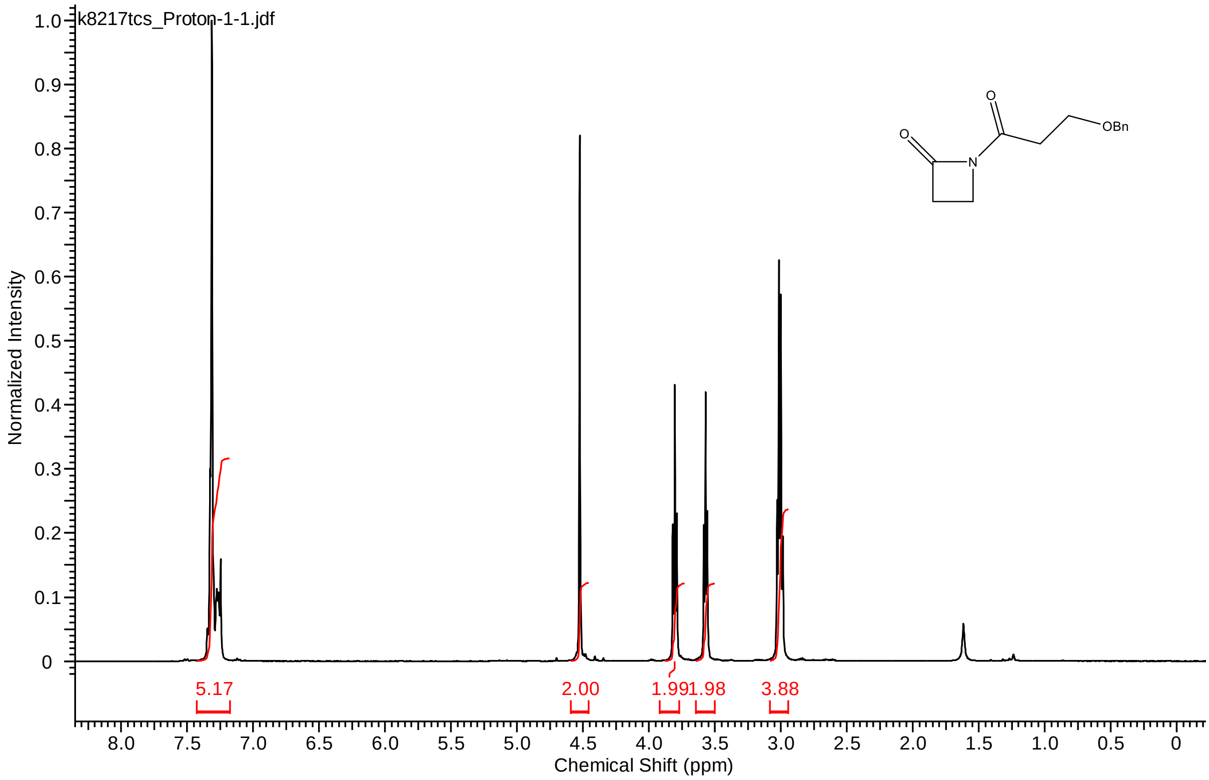
k8217tcs_Proton-1-1.jdf
Normalized Intensity
Chemical Shift (ppm)
5.17
2.00
1.99
1.98
3.88
O
O
N
OBn

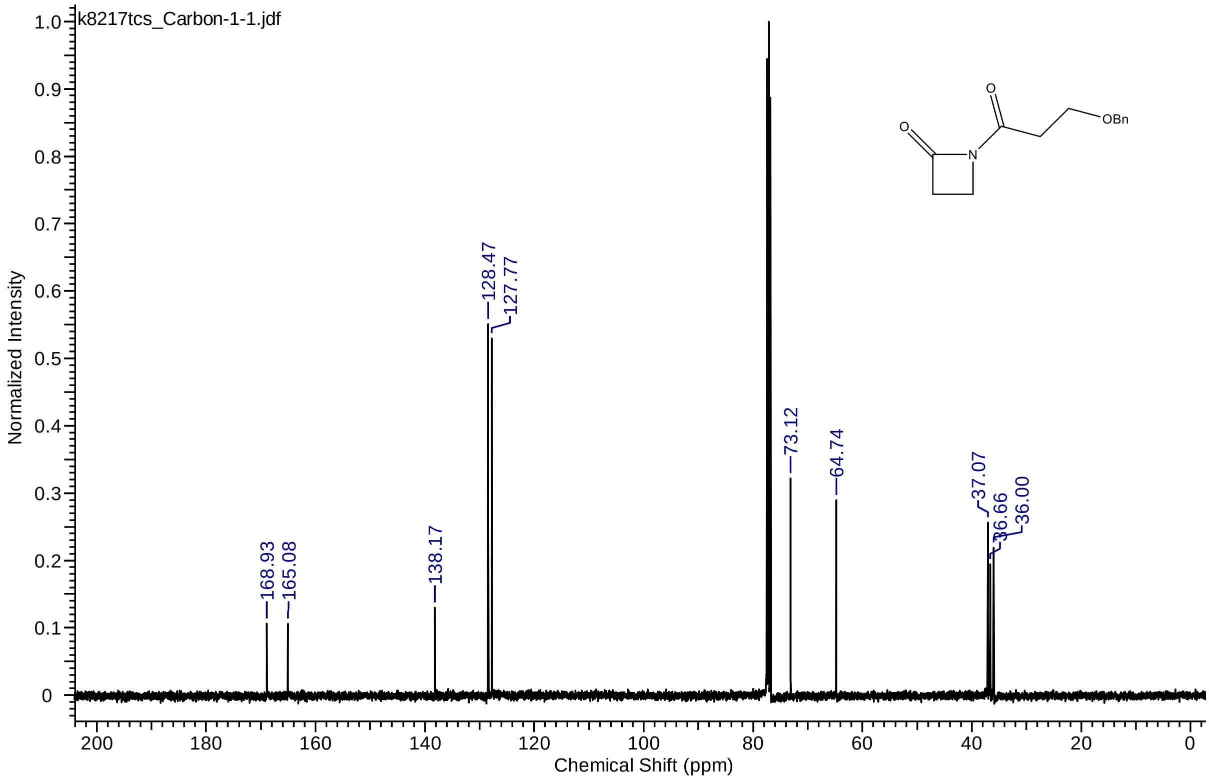
k8217tcs_Carbon-1-1.jdf
Normalized Intensity
Chemical Shift (ppm)
168.93
165.08
138.17
128.47
127.77
73.12
64.74
37.07
36.66
36.00
O
O
N
OBn

Compound 7c

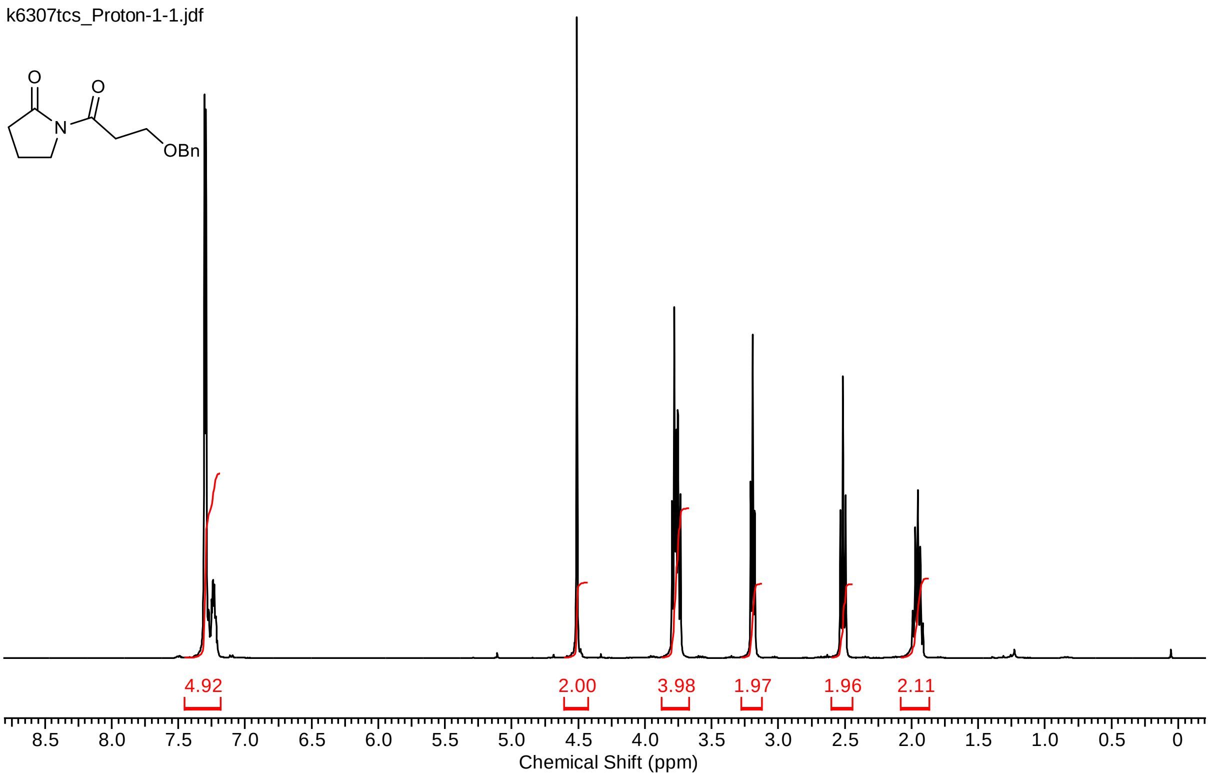
k6307tcs_Proton-1-1.jdf
O
O
N
OBn
4.92
2.00
3.98
1.97
1.96
2.11
8.5 8.0 7.5 7.0 6.5 6.0 5.5 5.0 4.5 4.0 3.5 3.0 2.5 2.0 1.5 1.0 0.5 0
Chemical Shift (ppm)

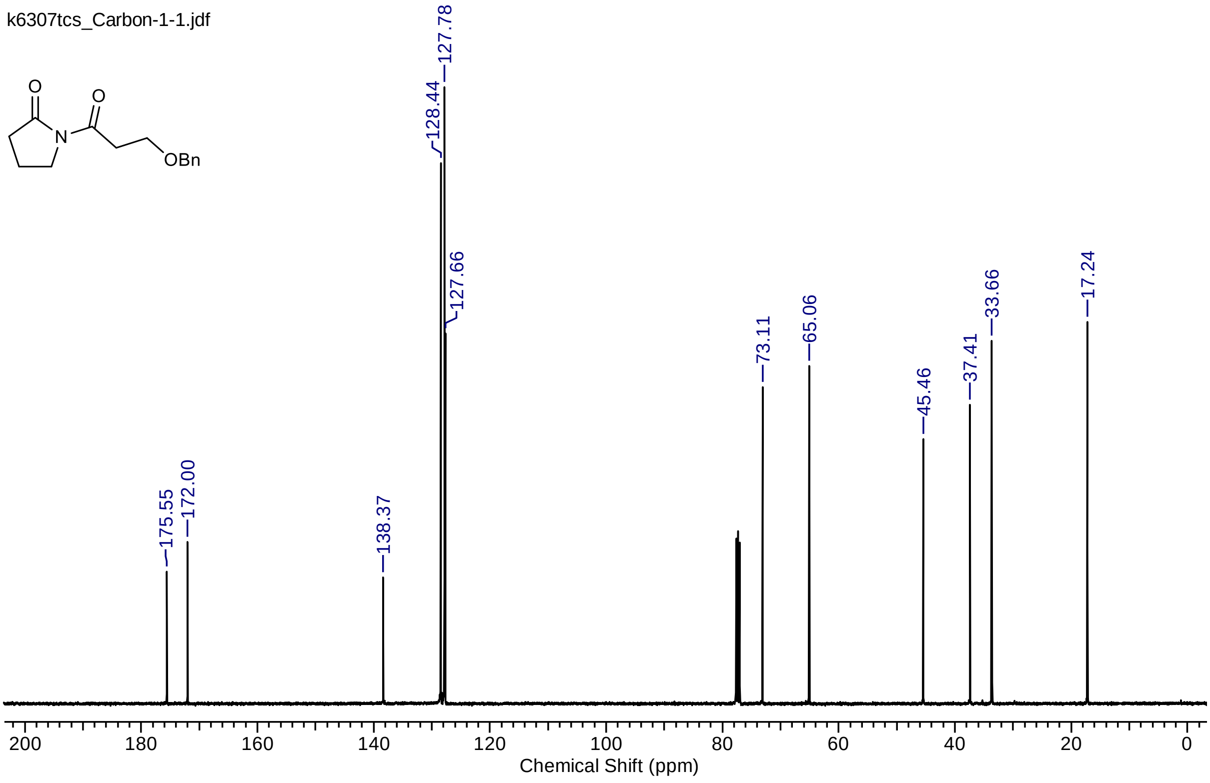
k6307tcs_Carbon-1-1.jdf
O
O
N
OBn
175.55
172.00
138.37
128.44
127.78
127.66
73.11
65.06
45.46
37.41
33.66
17.24
200 180 160 140 120 100 80 60 40 20 0
Chemical Shift (ppm)

Compound 7d

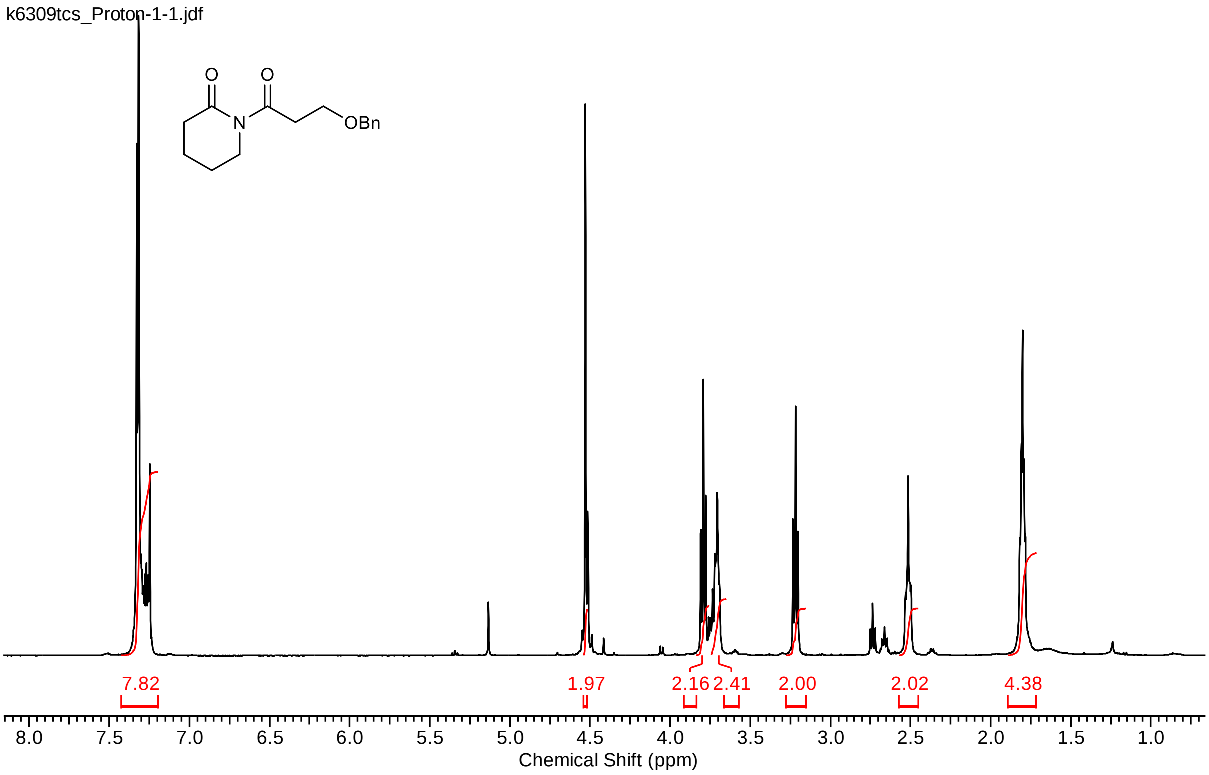
k6309tcs_Proton-1-1.jdf
O
O
N
OBn
7.82
1.97
2.16
2.41
2.00
2.02
4.38
8.0 7.5 7.0 6.5 6.0 5.5 5.0 4.5 4.0 3.5 3.0 2.5 2.0 1.5 1.0
Chemical Shift (ppm)

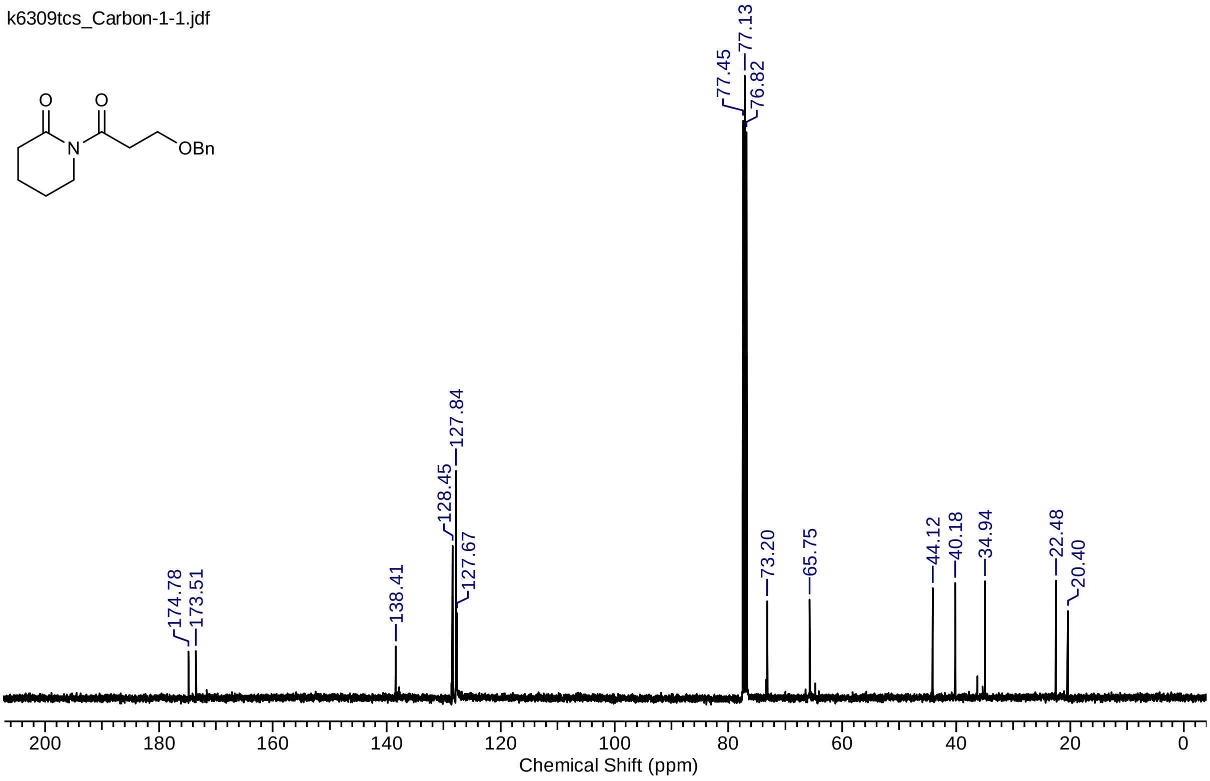
k6309tcs_Carbon-1-1.jdf
O
O
N
OBn
77.45
77.13
76.82
127.84
128.45
127.67
174.78
173.51
138.41
73.20
65.75
44.12
40.18
34.94
22.48
20.40
200 180 160 140 120 100 80 60 40 20 0
Chemical Shift (ppm)

Compound 7e

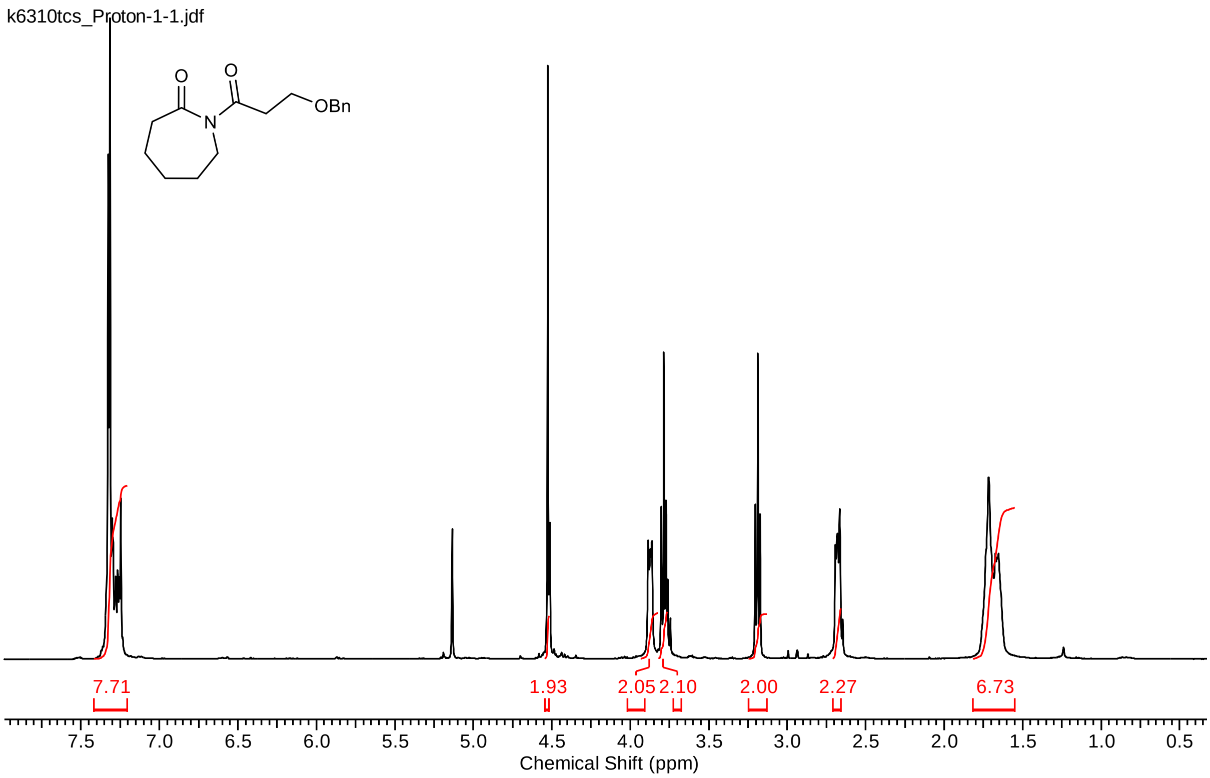
k6310tcs_Proton-1-1.jdf
O
O
N
OBn
7.71
1.93
2.05
2.10
2.00
2.27
6.73
7.5
7.0
6.5
6.0
5.5
5.0
4.5
4.0
3.5
3.0
2.5
2.0
1.5
1.0
0.5
Chemical Shift (ppm)

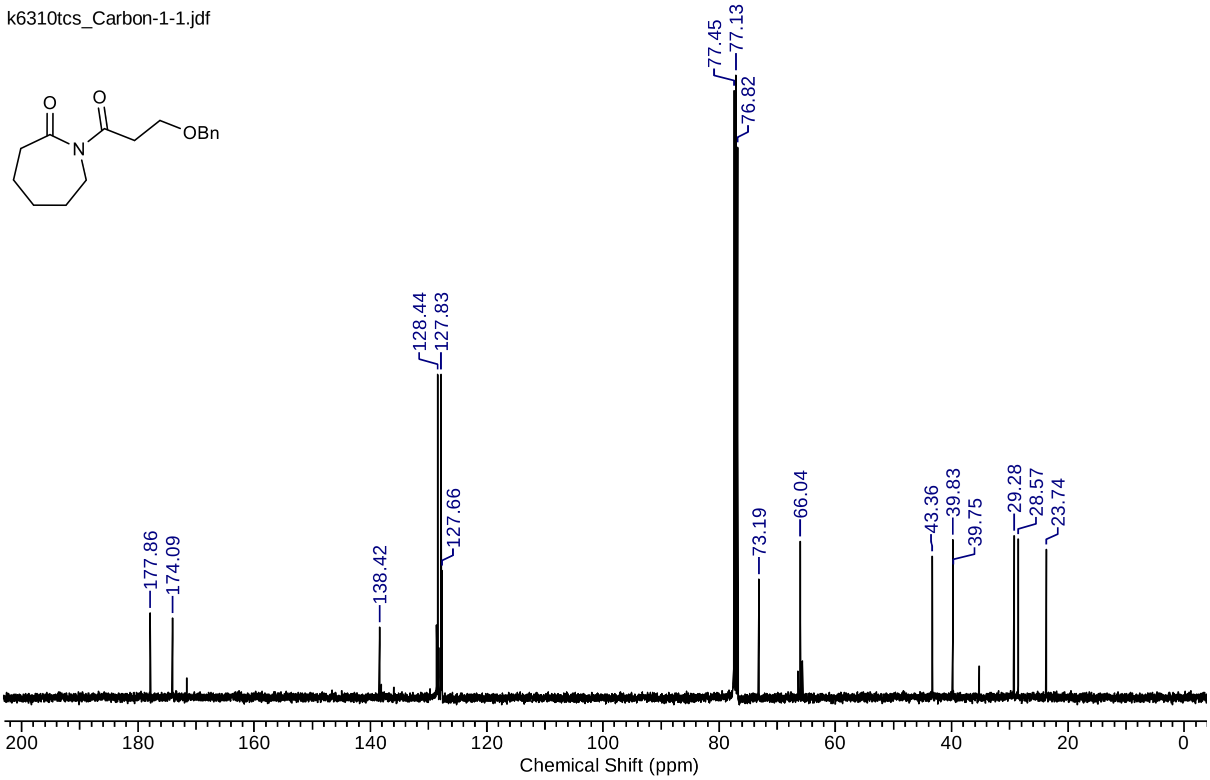
k6310tcs_Carbon-1-1.jdf
O
O
N
OBn
77.45
77.13
76.82
128.44
127.83
127.66
177.86
174.09
138.42
73.19
66.04
43.36
39.83
39.75
29.28
28.57
23.74
200
180
160
140
120
100
80
60
40
20
0
Chemical Shift (ppm)

Compound 7f

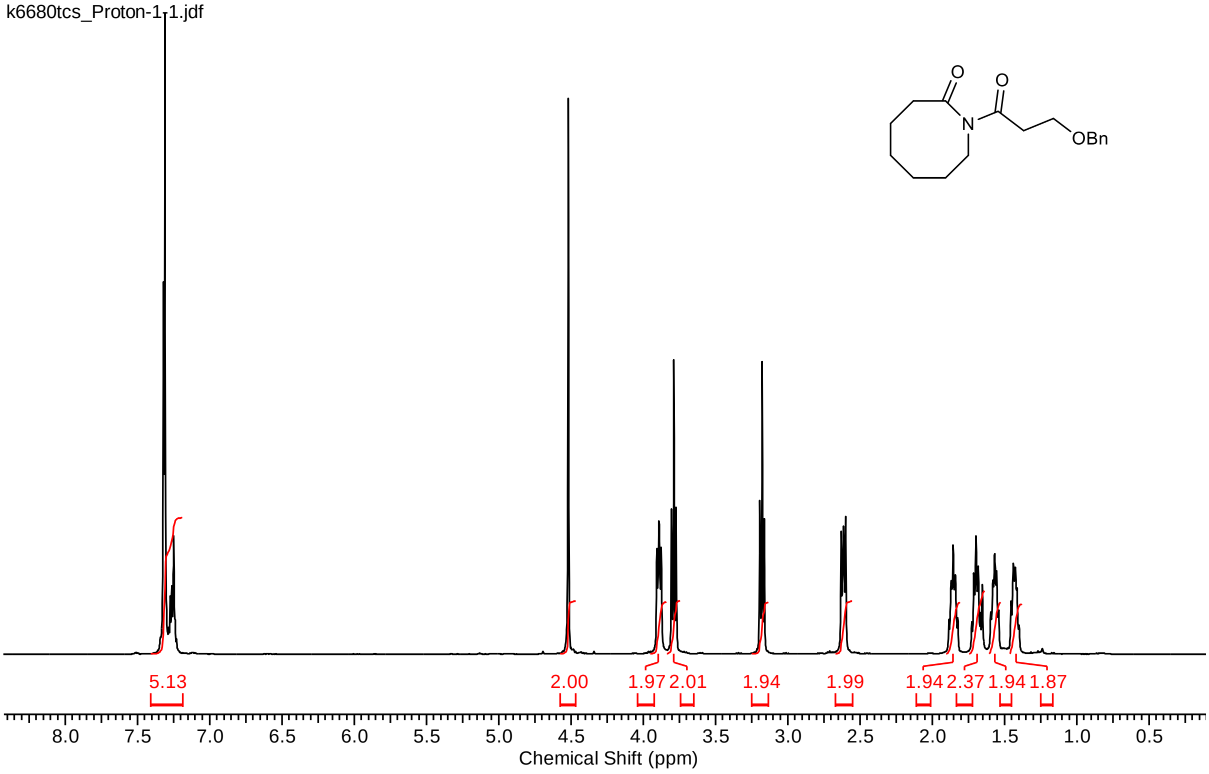
k6680tcs_Proton-1-1.jdf
O
O
N
OBn
5.13
2.00
1.97
2.01
1.94
1.99
1.94
2.37
1.94
1.87
8.0
7.5
7.0
6.5
6.0
5.5
5.0
4.5
4.0
3.5
3.0
2.5
2.0
1.5
1.0
0.5
Chemical Shift (ppm)

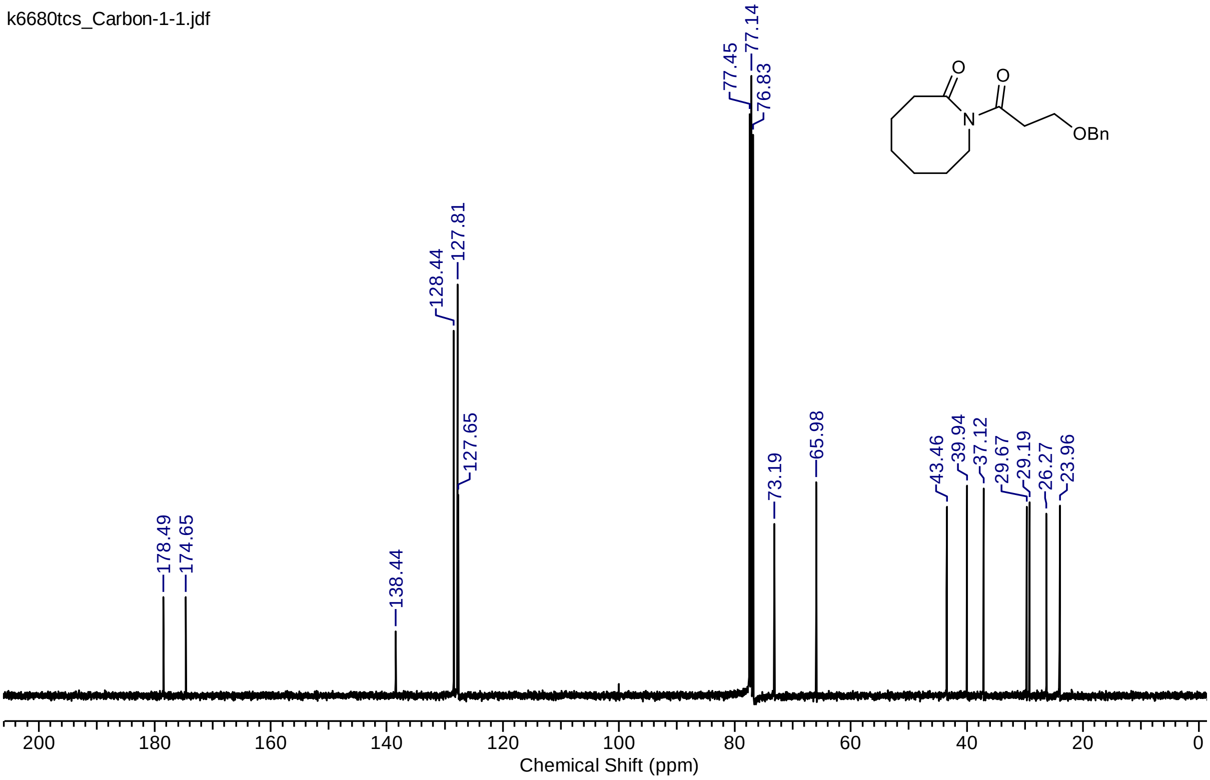
k6680tcs_Carbon-1-1.jdf
77.45
77.14
76.83
O
O
N
OBn
128.44
127.81
127.65
178.49
174.65
138.44
73.19
65.98
43.46
39.94
37.12
29.67
29.19
26.27
23.96
200
180
160
140
120
100
80
60
40
20
0
Chemical Shift (ppm)

Compound 7g

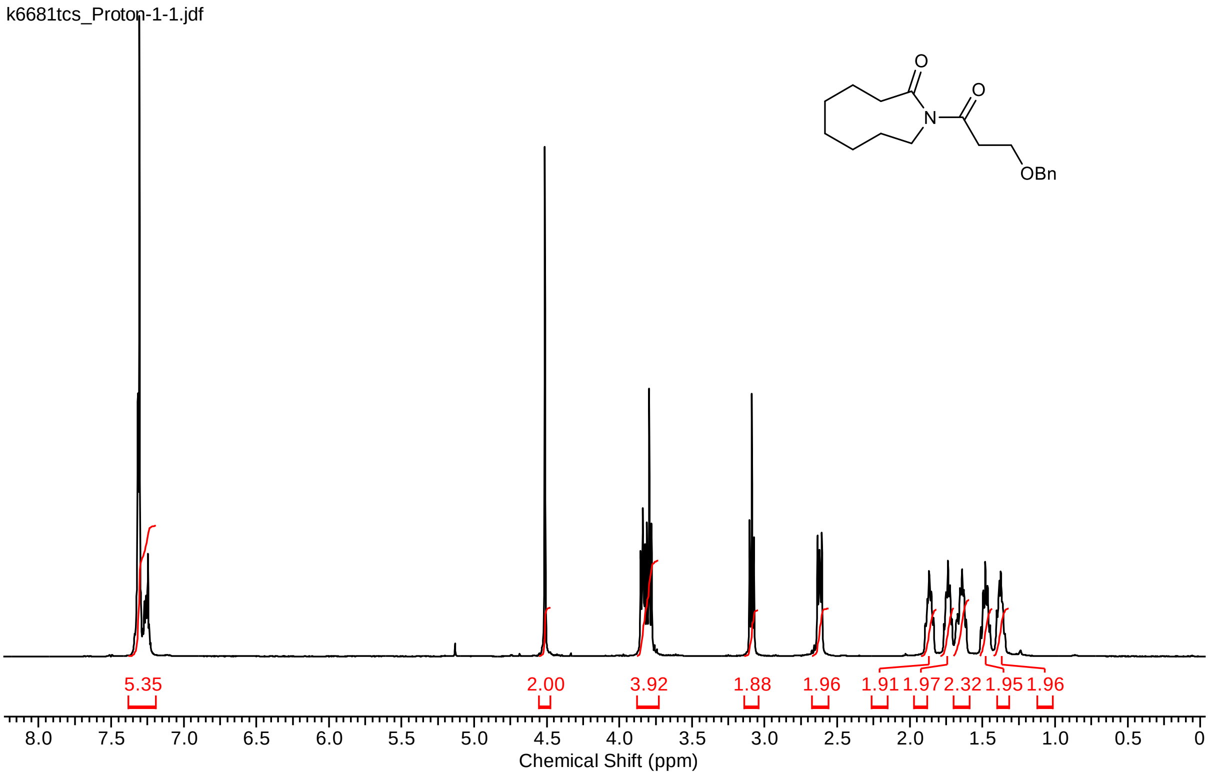
k6681tcs_Proton-1-1.jdf
O
O
N
OBn
5.35
2.00
3.92
1.88
1.96
1.91
1.97
2.32
1.95
1.96
8.0
7.5
7.0
6.5
6.0
5.5
5.0
4.5
4.0
3.5
3.0
2.5
2.0
1.5
1.0
0.5
0
Chemical Shift (ppm)

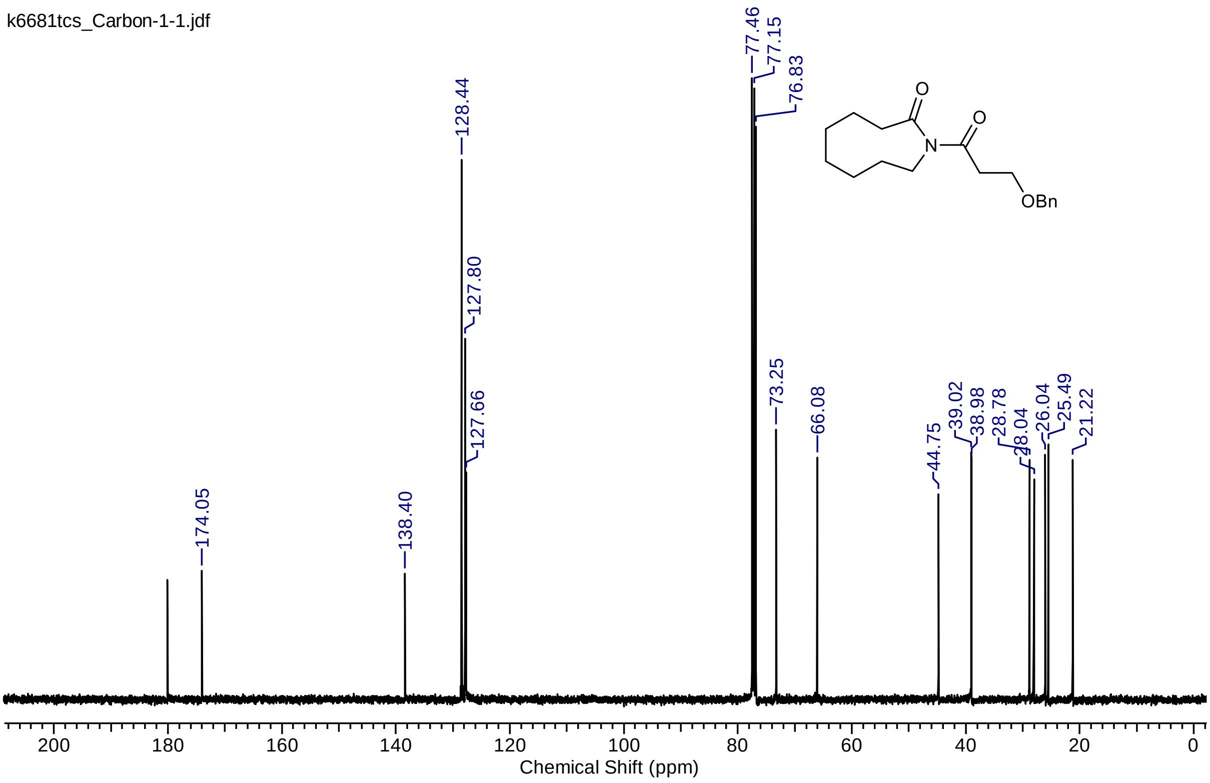
k6681tcs_Carbon-1-1.jdf
O
O
N
OBn
77.46
77.15
76.83
128.44
127.80
127.66
174.05
138.40
73.25
66.08
44.75
39.02
38.98
28.78
28.04
26.04
25.49
21.22
200
180
160
140
120
100
80
60
40
20
0
Chemical Shift (ppm)

Compound 7h

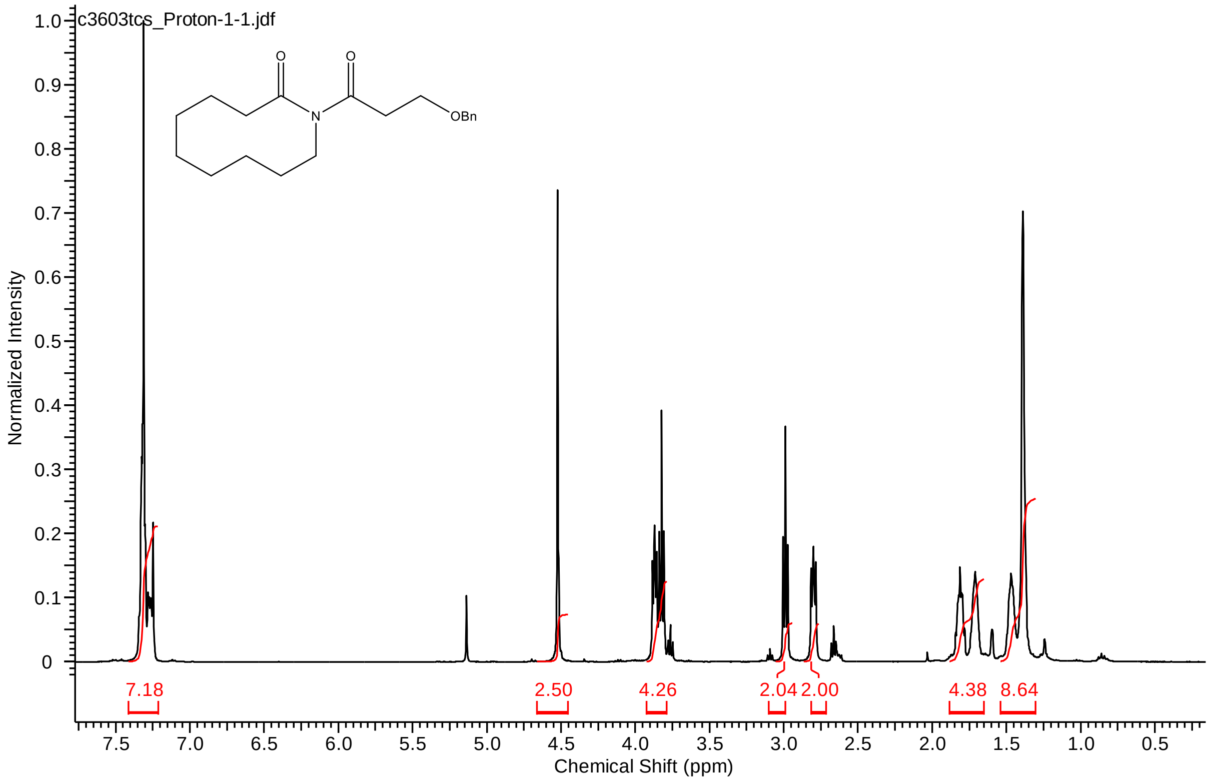
c3603tcs_Proton-1-1.jdf
O
O
N
OBn
Normalized Intensity
7.18
2.50
4.26
2.04
2.00
4.38
8.64
Chemical Shift (ppm)

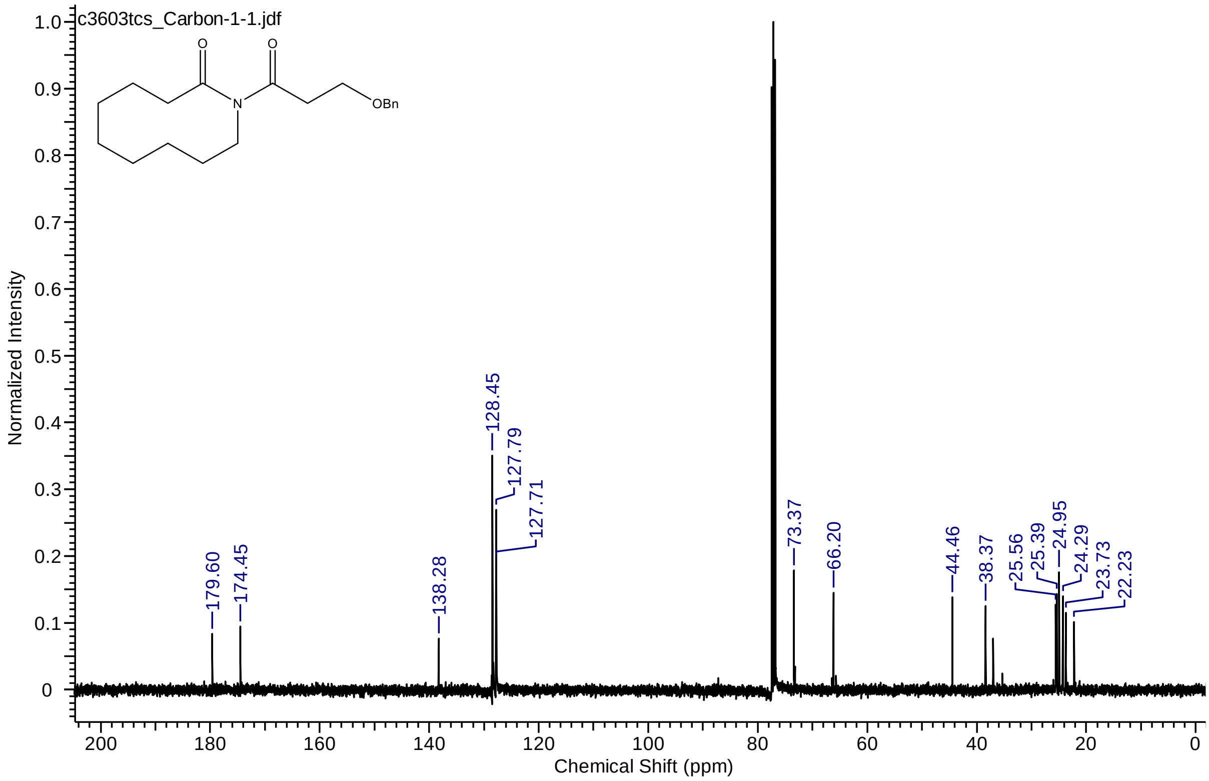
c3603tcs_Carbon-1-1.jdf
O
O
N
OBn
Normalized Intensity
179.60
174.45
138.28
128.45
127.79
127.71
73.37
66.20
44.46
38.37
25.56
25.39
24.95
24.29
23.73
22.23
Chemical Shift (ppm)

Compound 7i

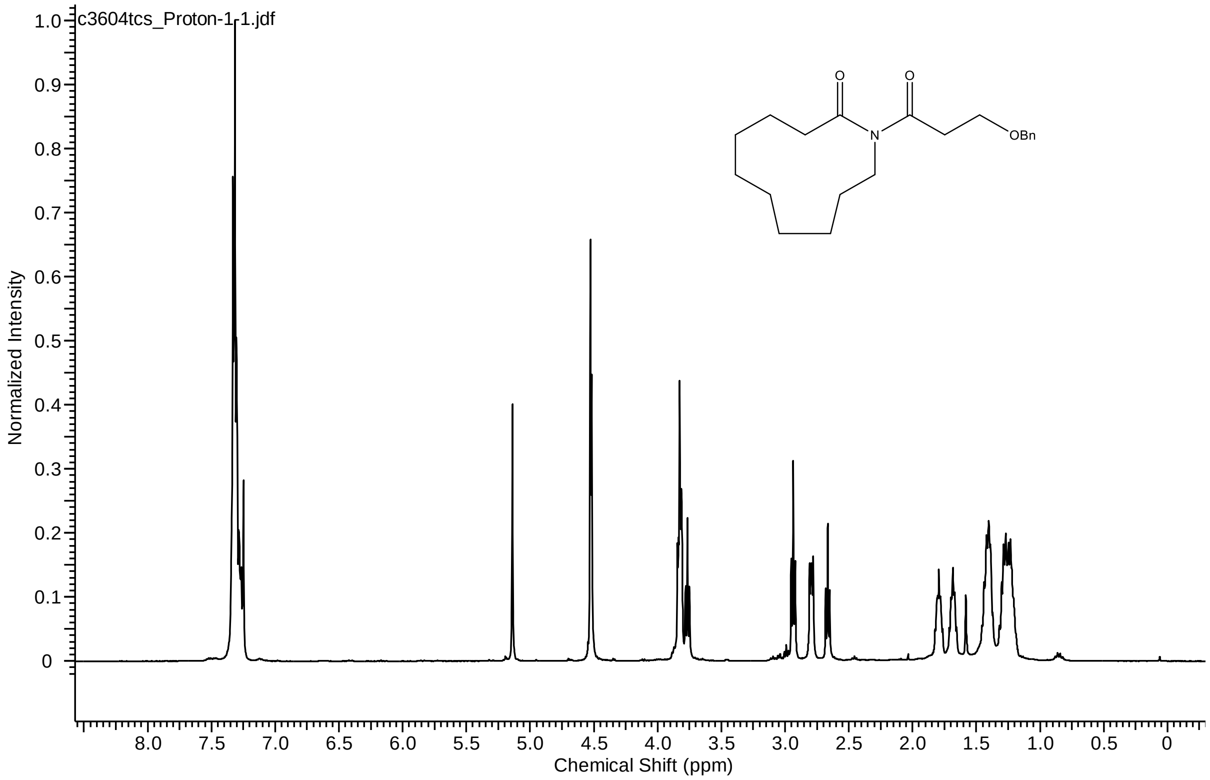

c3604tcs_Proton-1-1.jdf
Normalized Intensity
Chemical Shift (ppm)
O
O
N
OBn

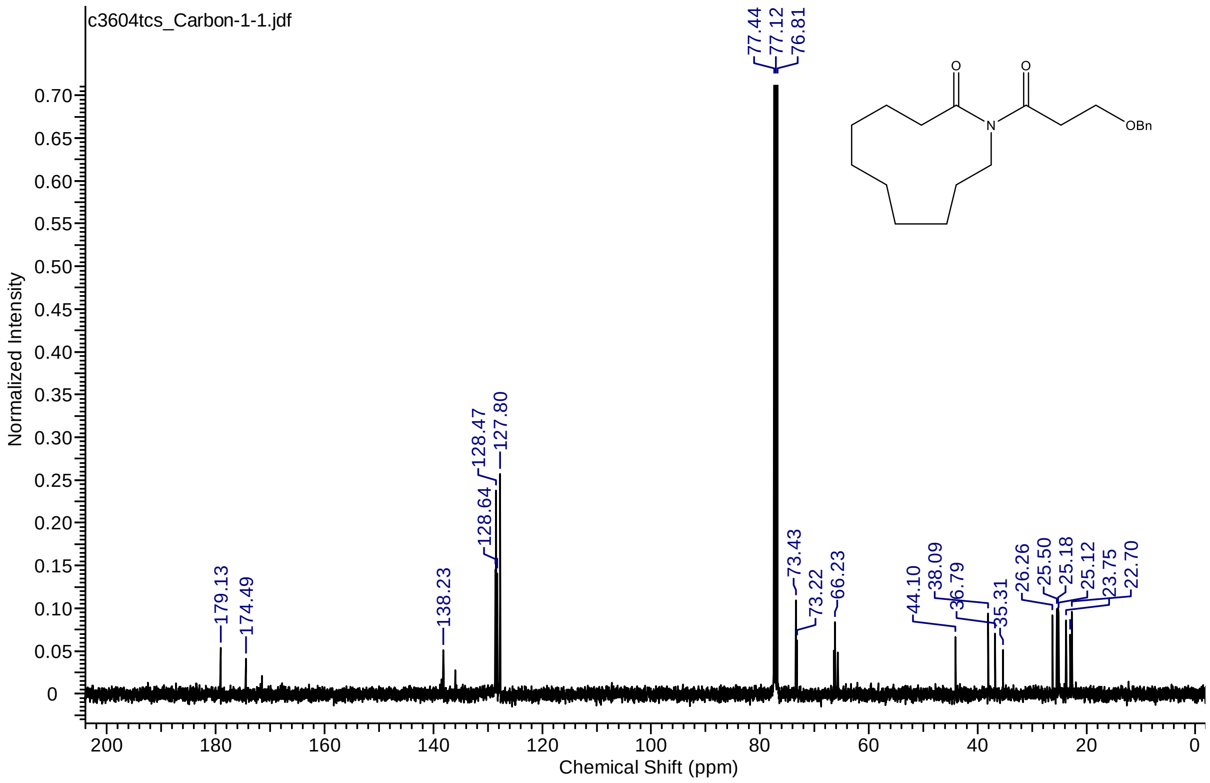

c3604tcs_Carbon-1-1.jdf
Normalized Intensity
Chemical Shift (ppm)
77.44
77.12
76.81
179.13
174.49
138.23
128.64
128.47
127.80
73.43
73.22
66.23
44.10
38.09
36.79
35.31
26.26
25.50
25.18
25.12
23.75
22.70
O
O
N
OBn

Compound 7j

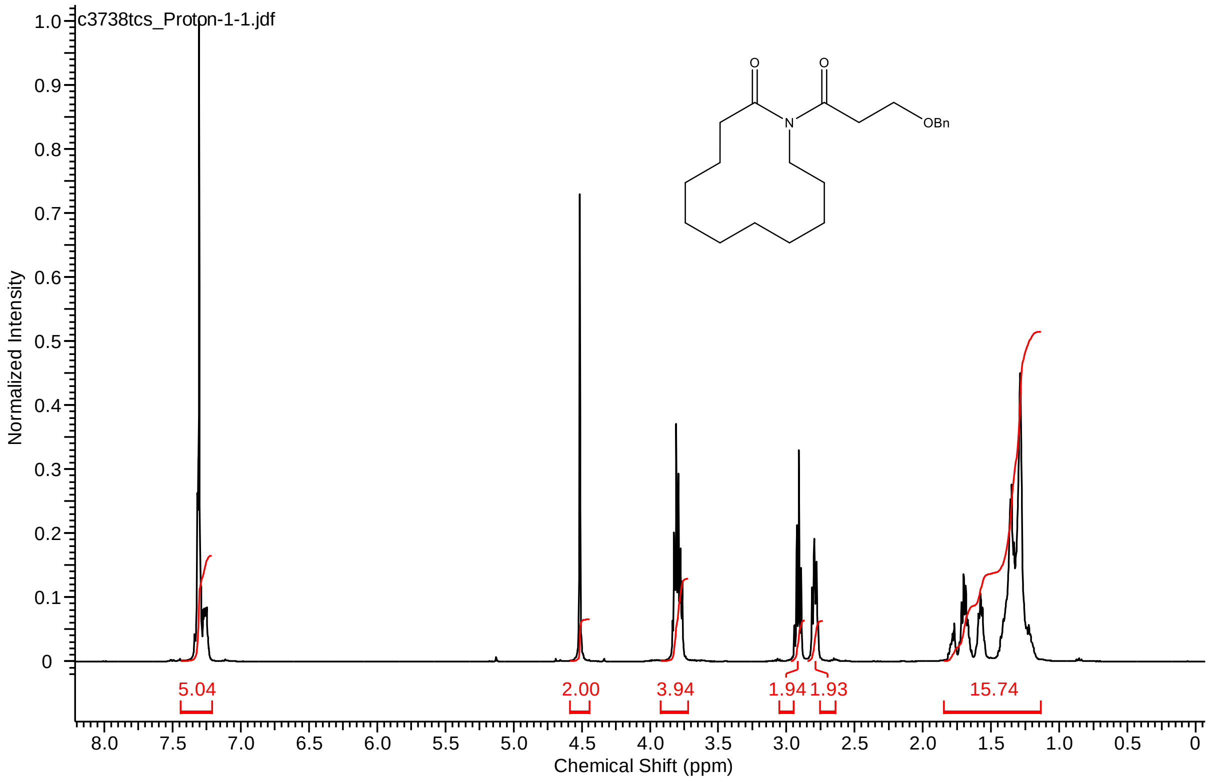
c3738tcs_Proton-1-1.jdf
Normalized Intensity
O
O
N
OBn
5.04
2.00
3.94
1.94
1.93
15.74
Chemical Shift (ppm)

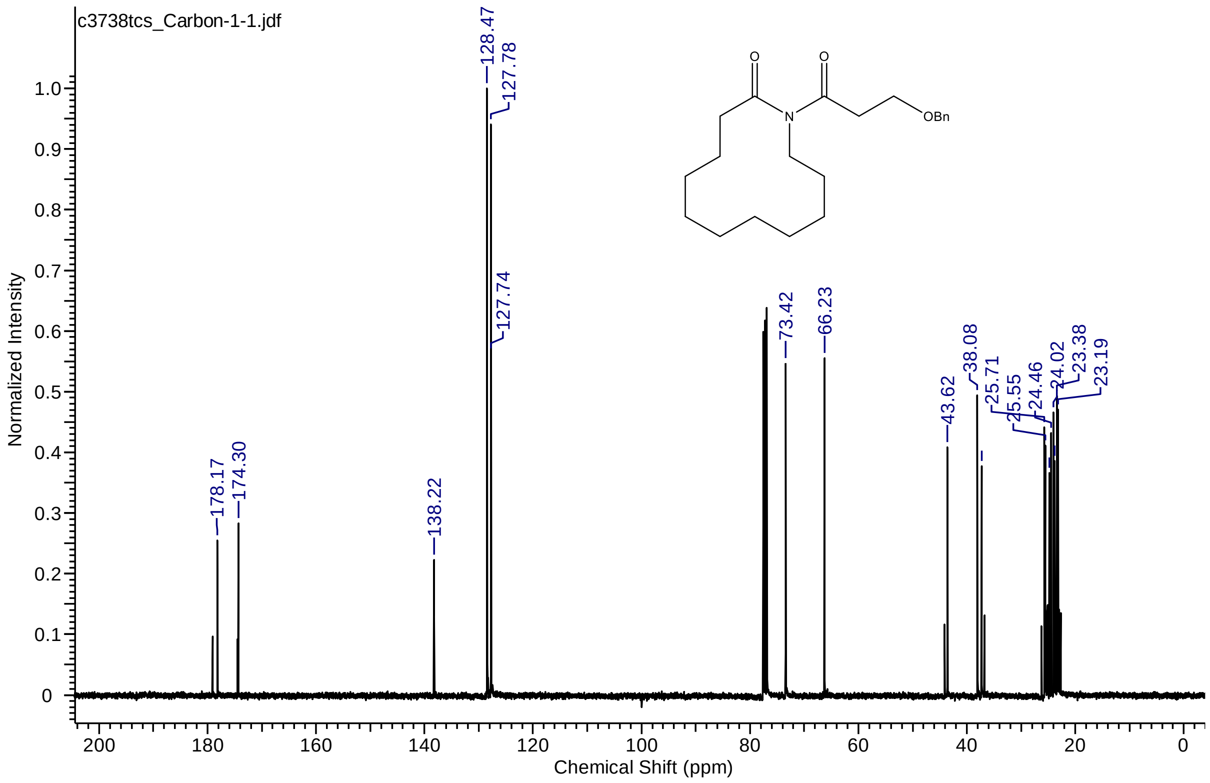
c3738tcs_Carbon-1-1.jdf
Normalized Intensity
O
O
N
OBn
128.47
127.78
127.74
178.17
174.30
138.22
73.42
66.23
43.62
38.08
25.71
25.55
24.46
24.02
23.38
23.19
Chemical Shift (ppm)

Compound 15d

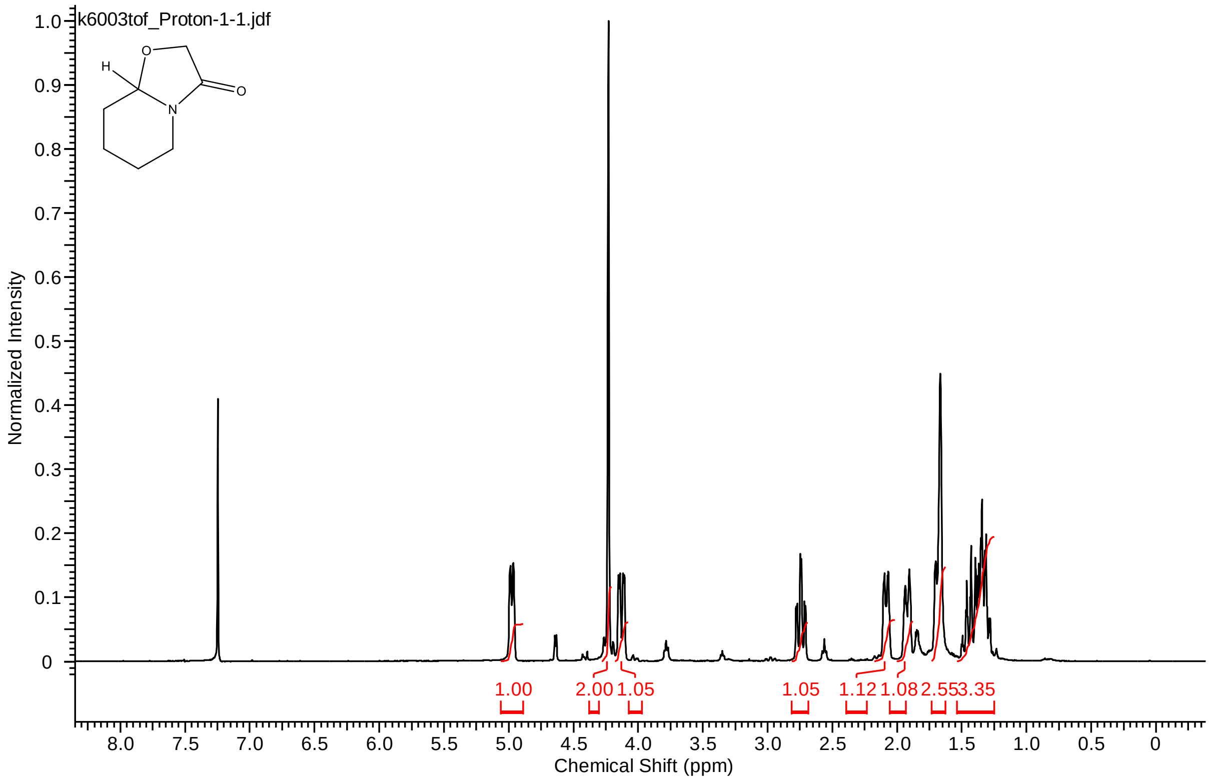

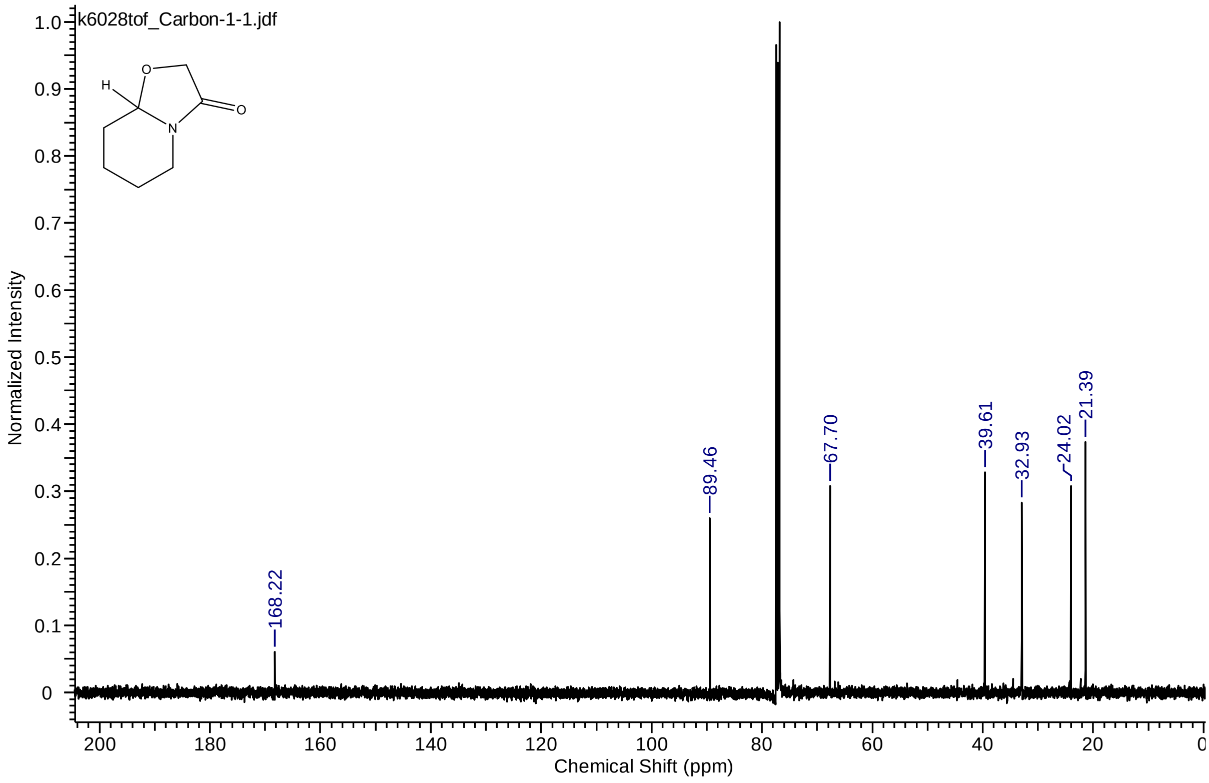

Compound 15e

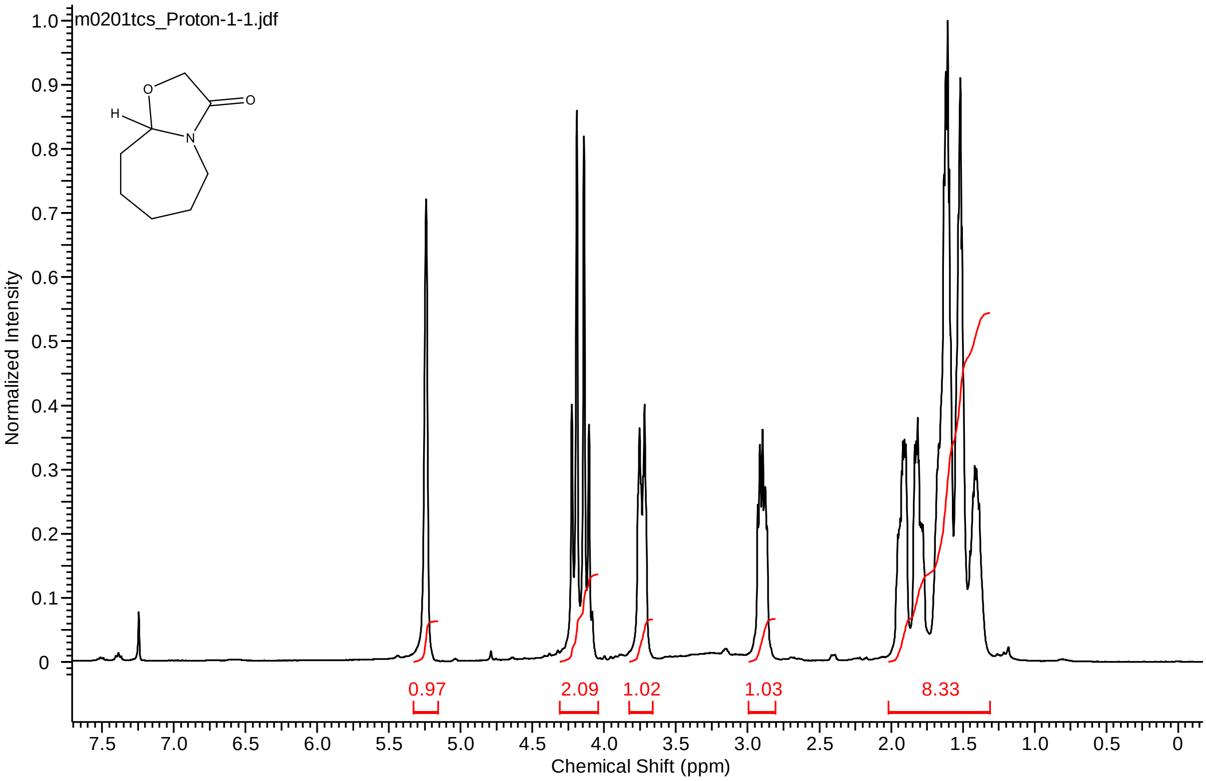

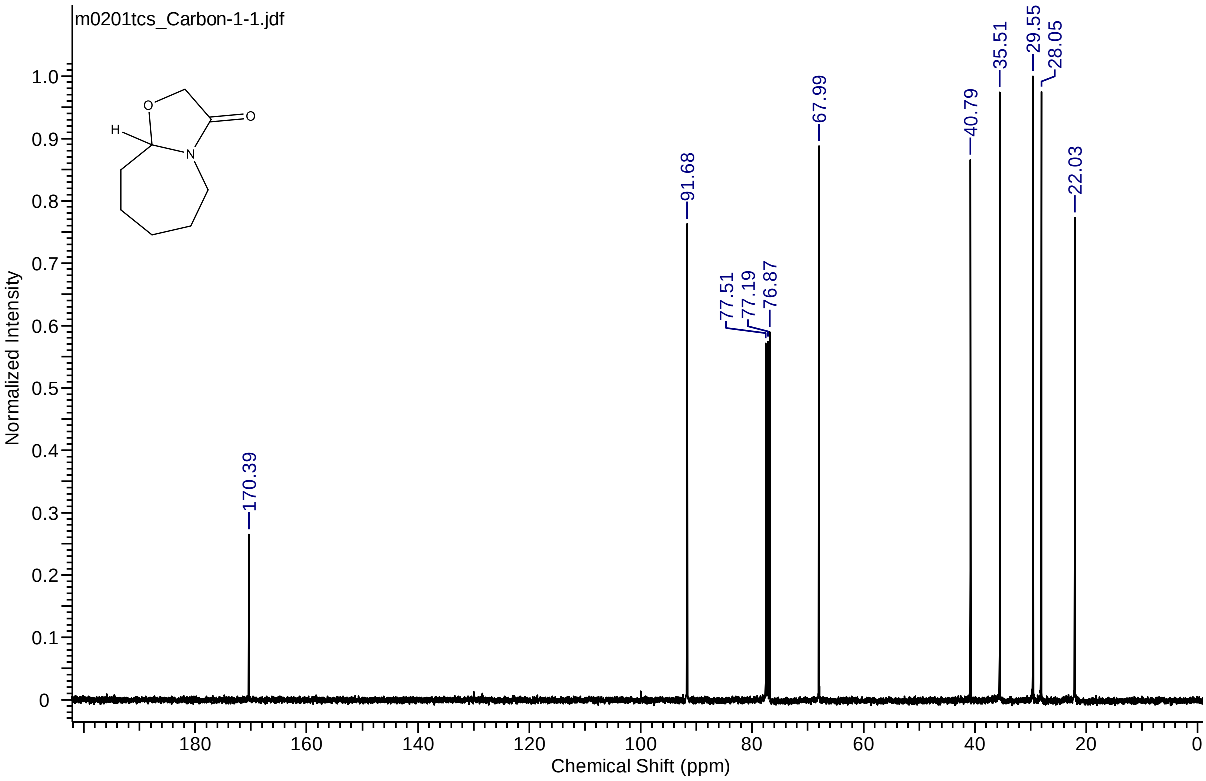

Compound 15f

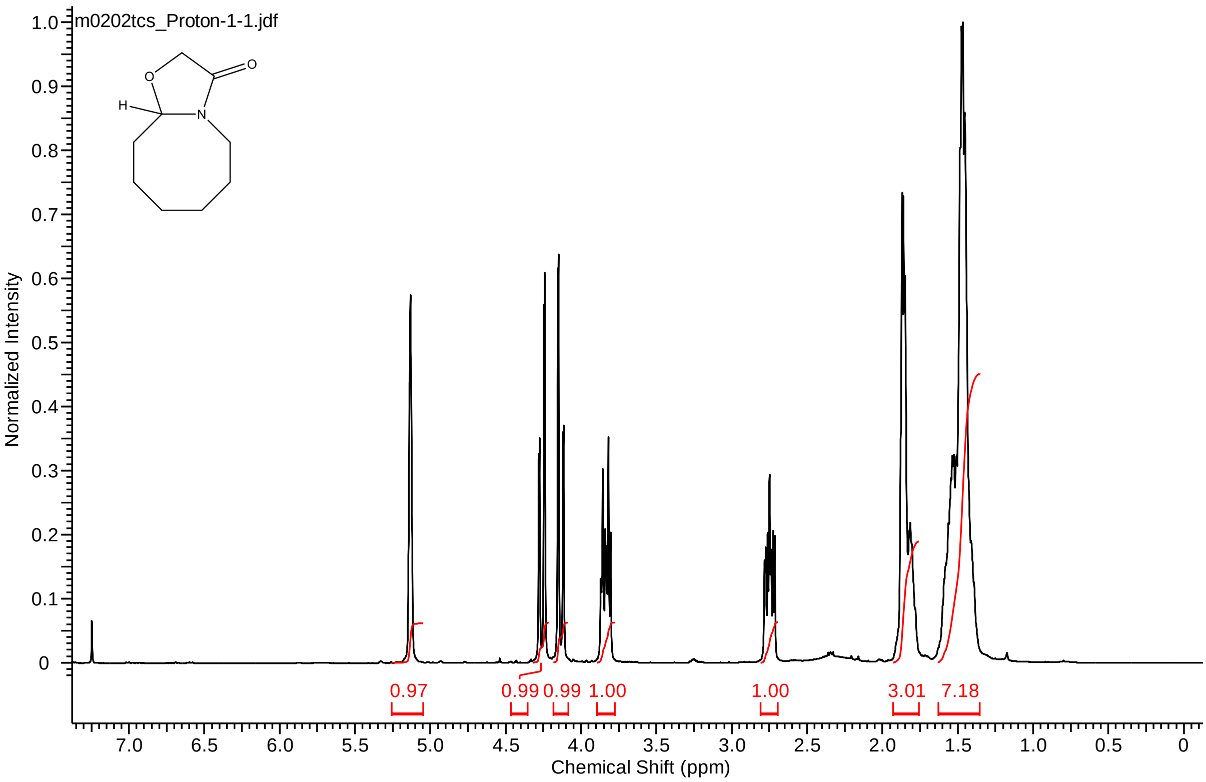

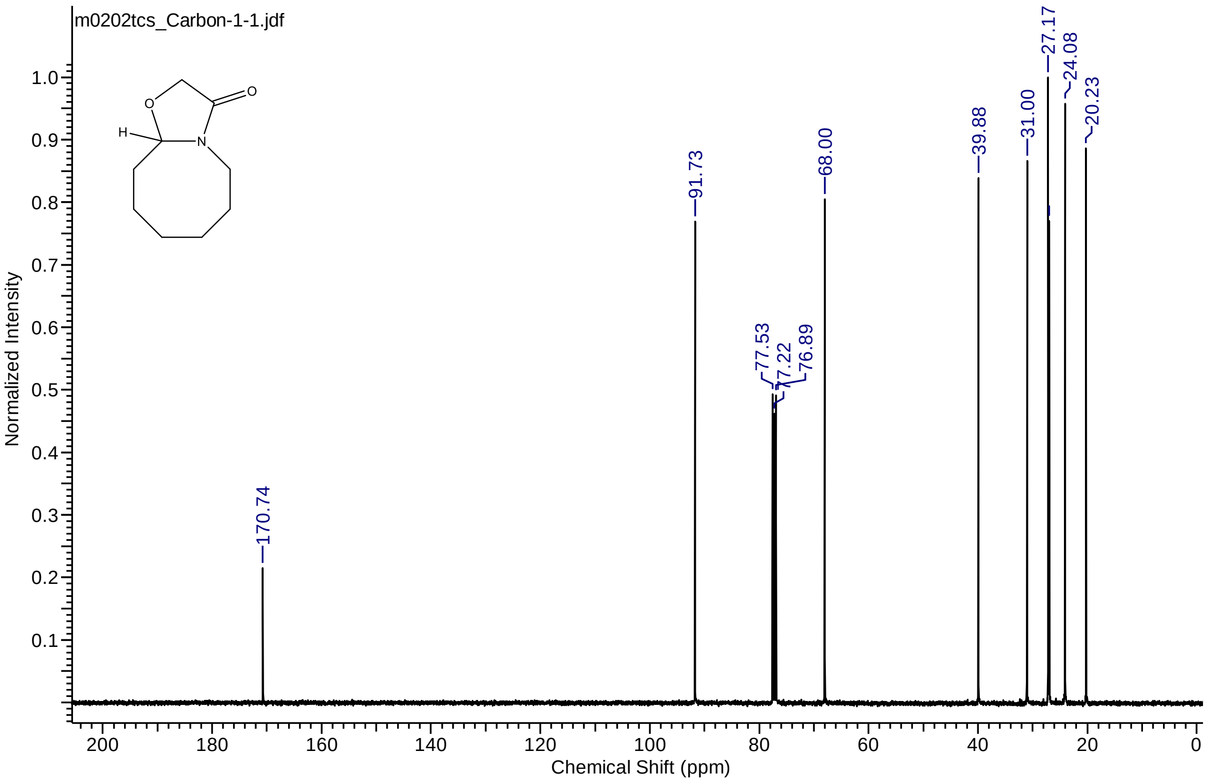

Compound 15g

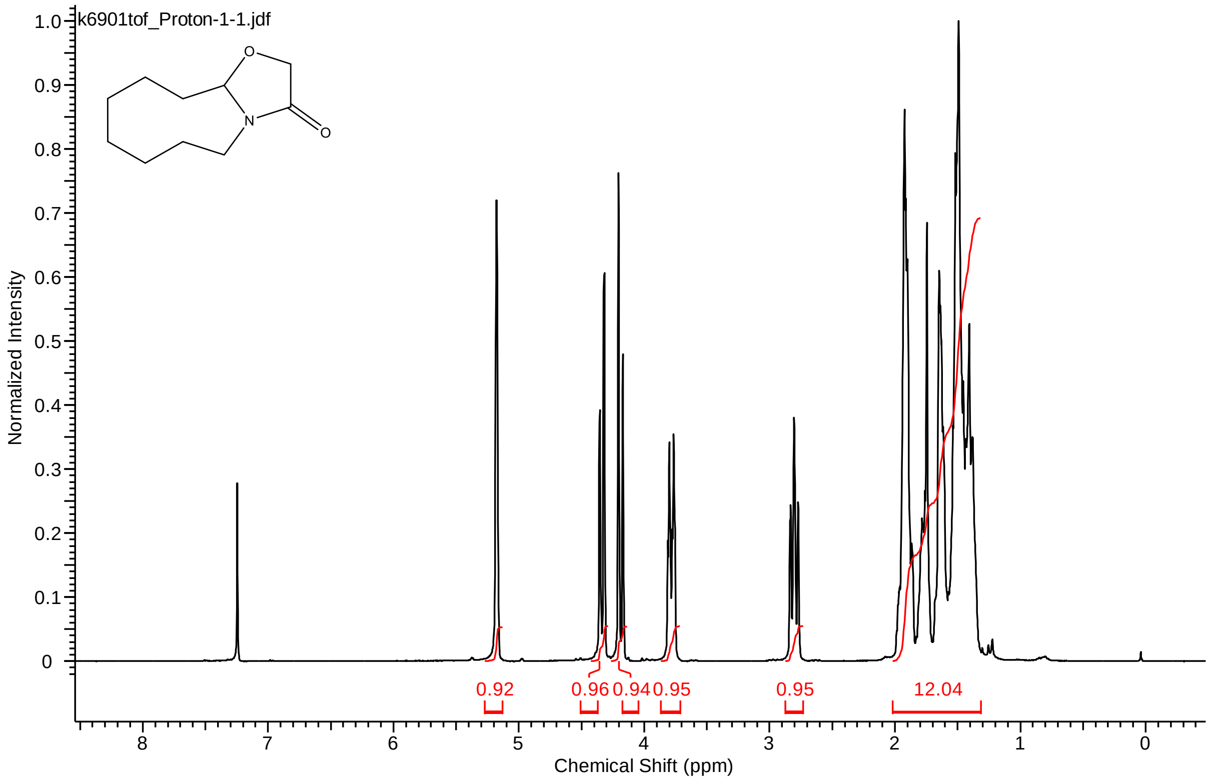

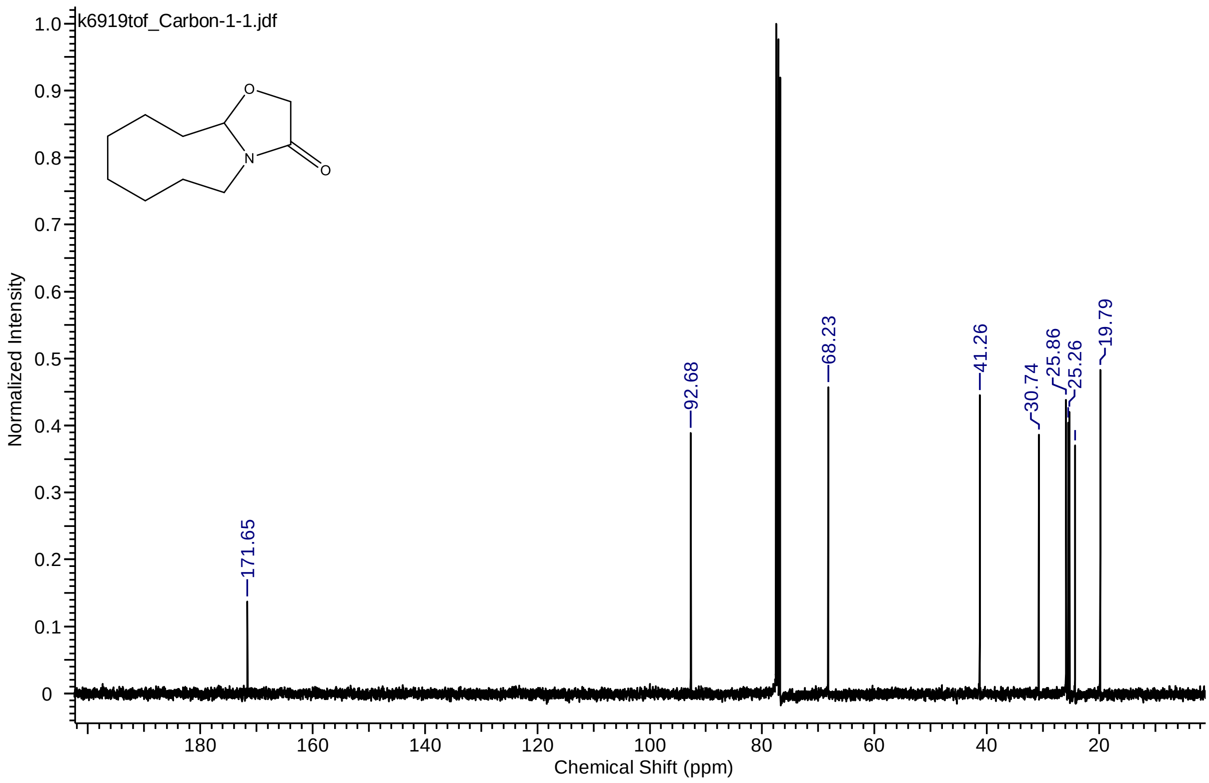

Compound 15h

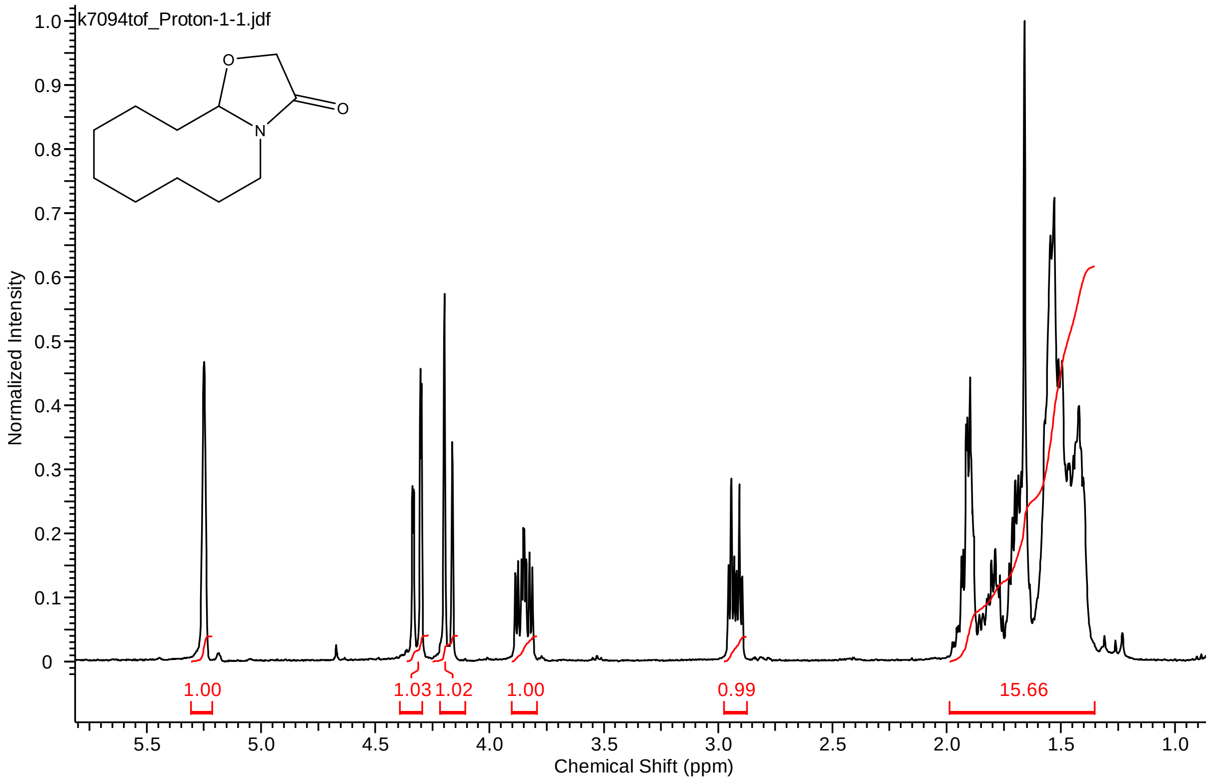

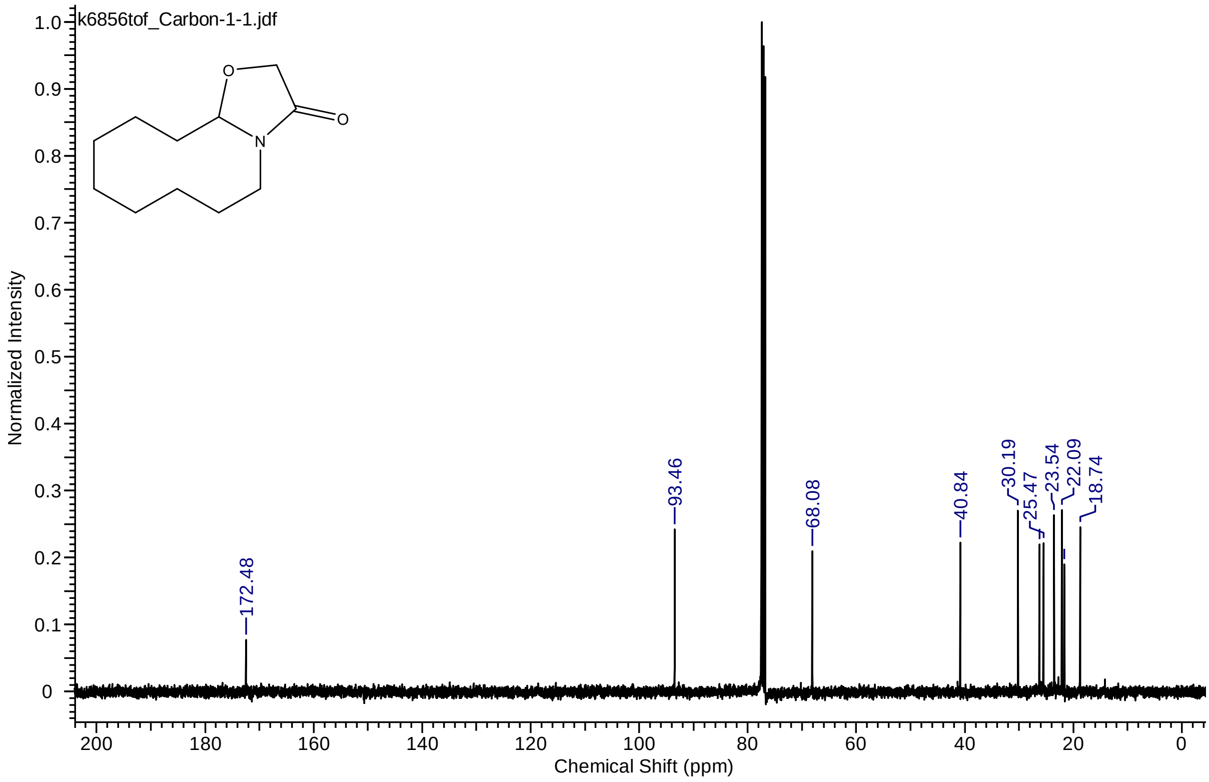

Compound 14f

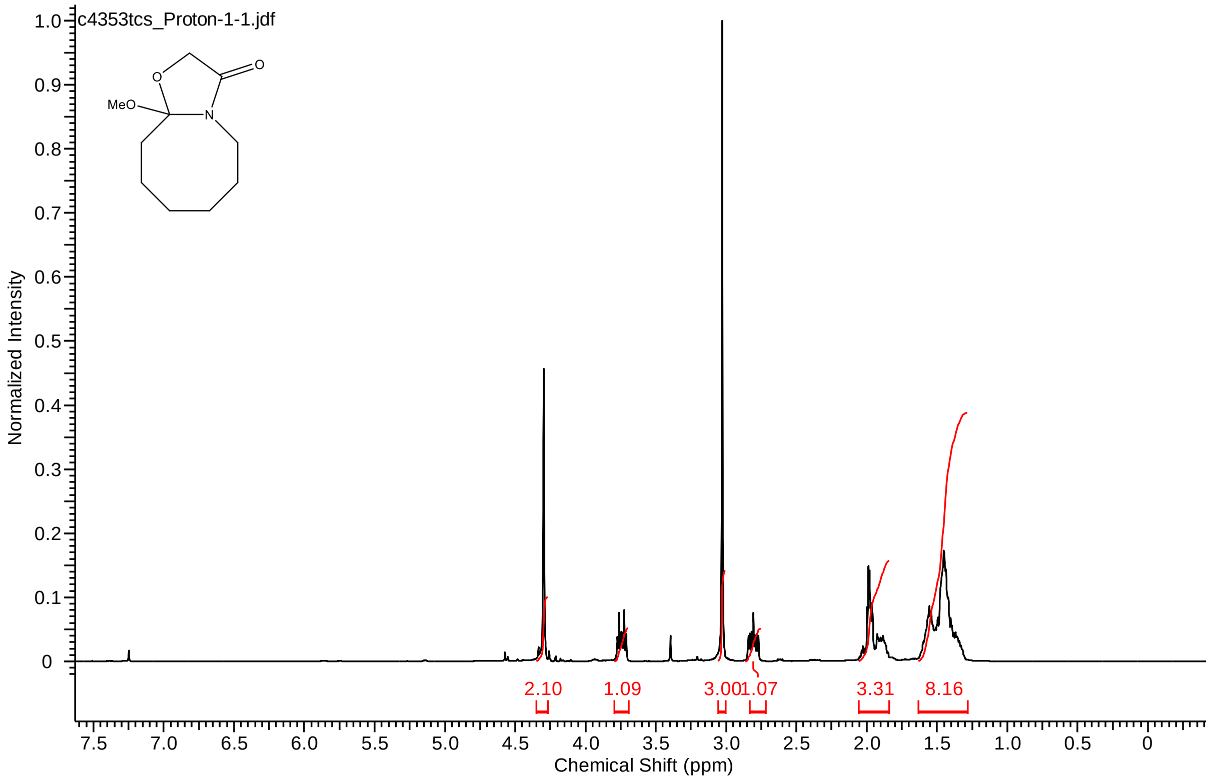

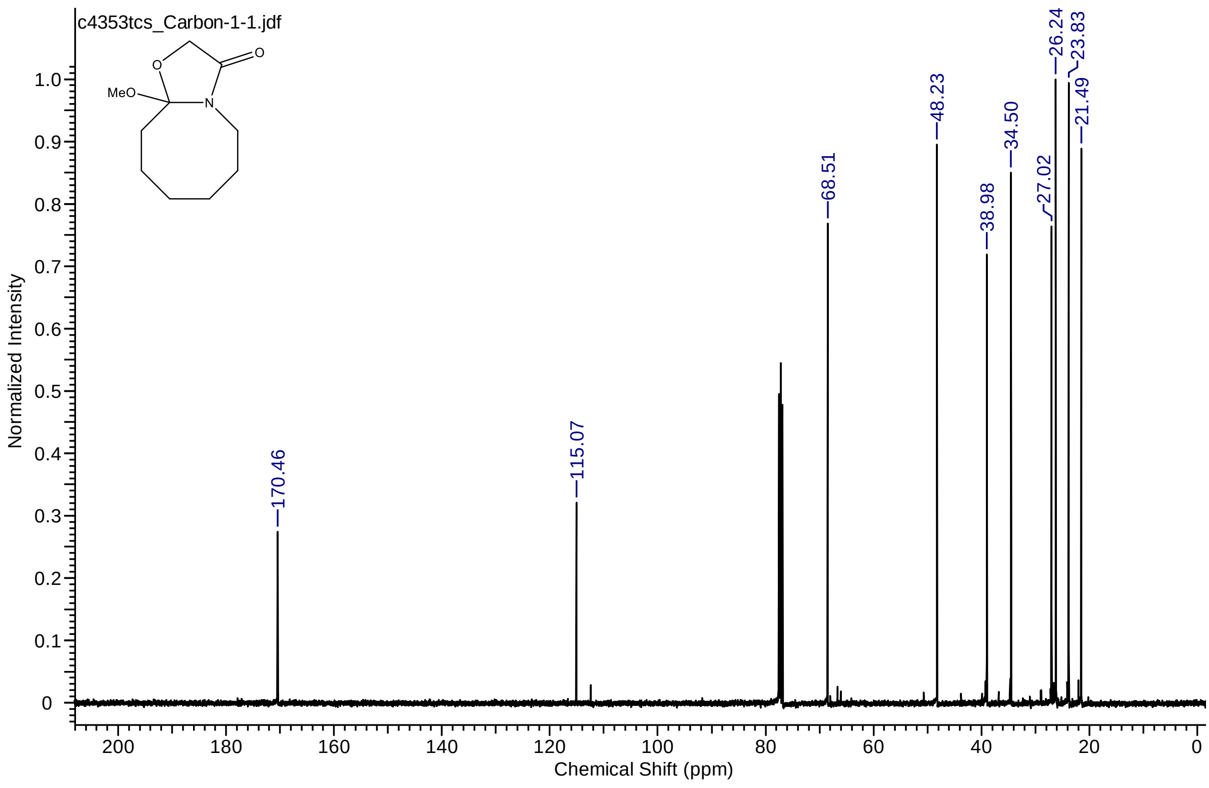

Compound 12a

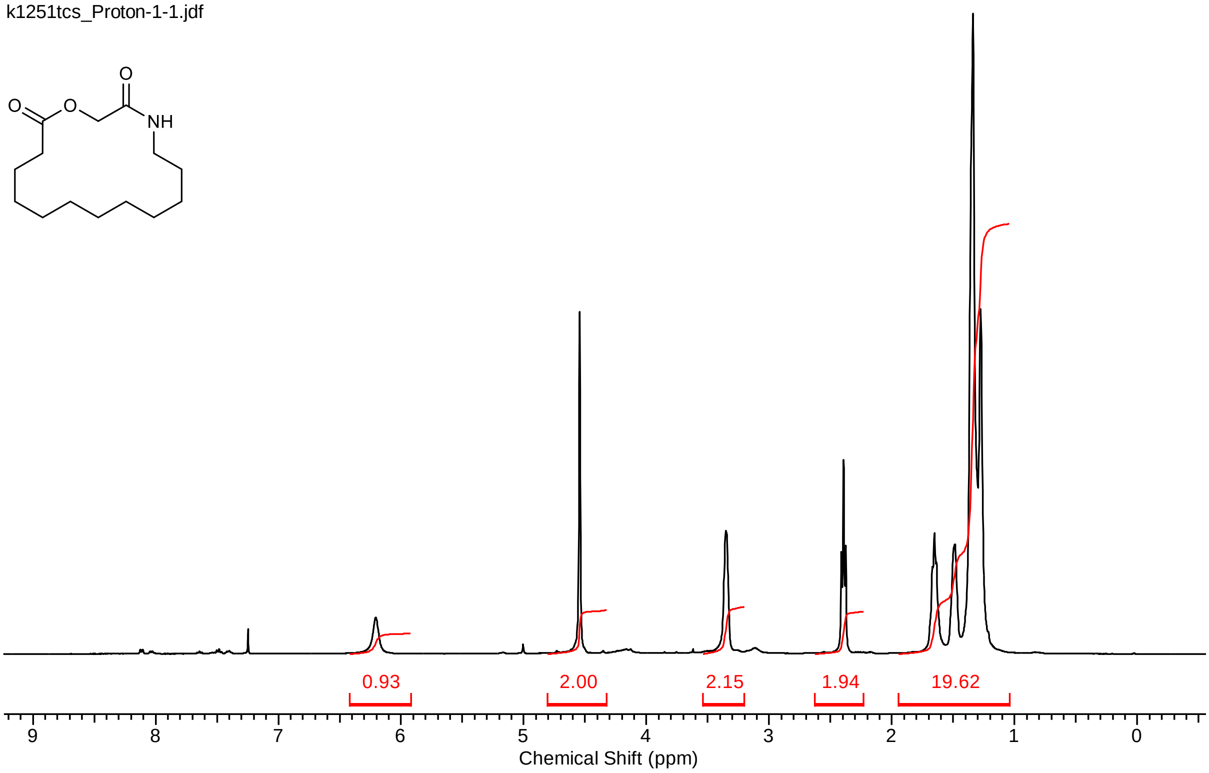

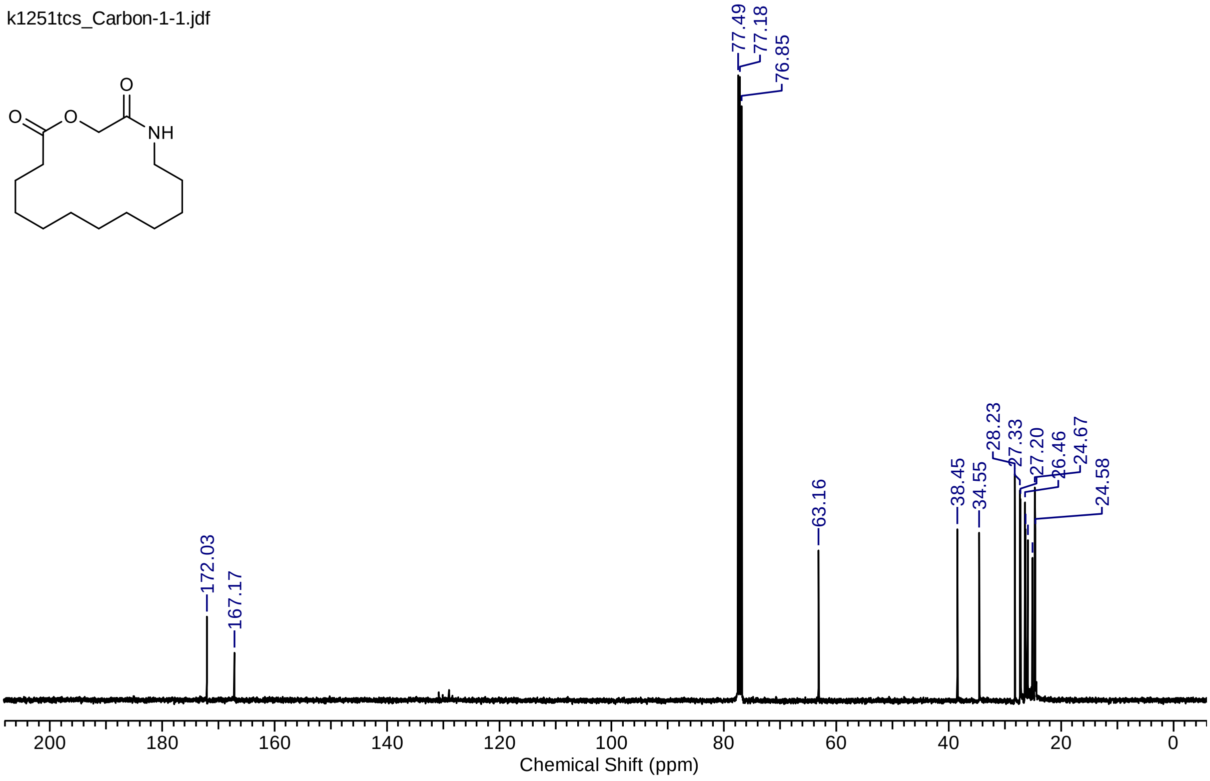

Compound 8b

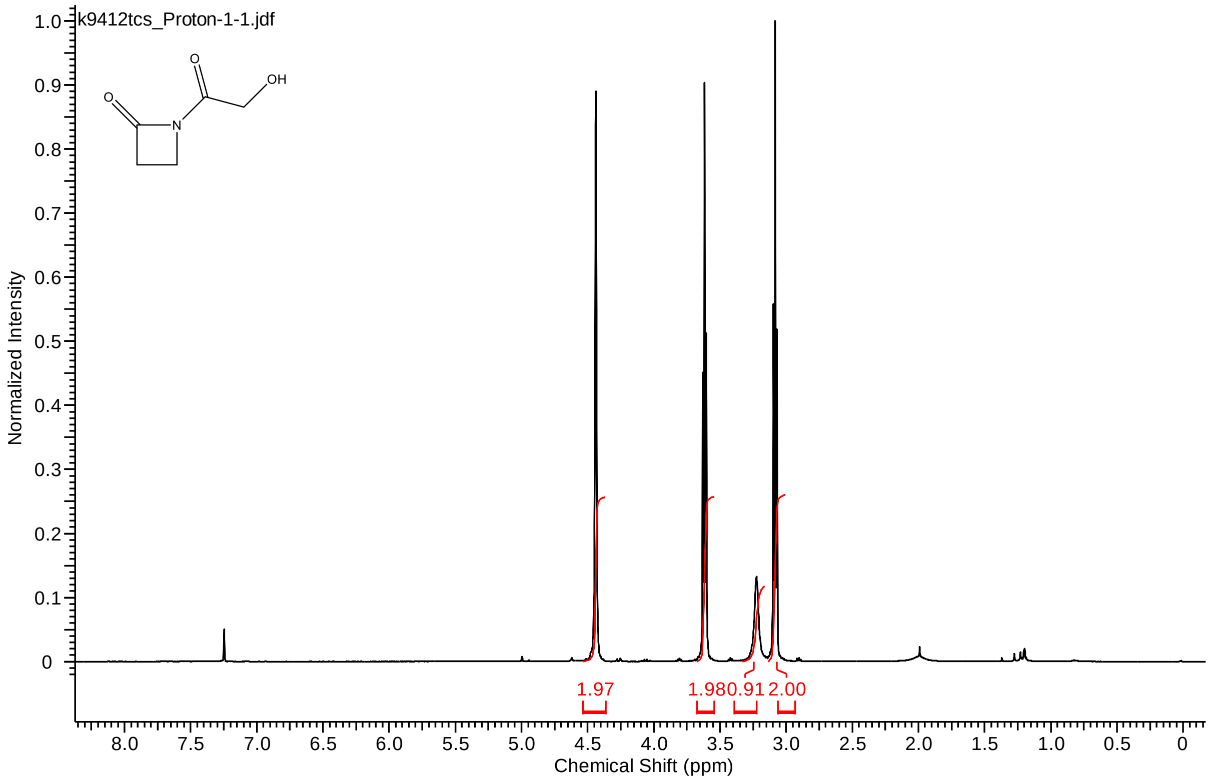

k9412tcs_Proton-1-1.jdf
O
O
N
OH
1.97
1.98
0.91
2.00
8.0
7.5
7.0
6.5
6.0
5.5
5.0
4.5
4.0
3.5
3.0
2.5
2.0
1.5
1.0
0.5
0
Chemical Shift (ppm)
Normalized Intensity
0
0.1
0.2
0.3
0.4
0.5
0.6
0.7
0.8
0.9
1.0

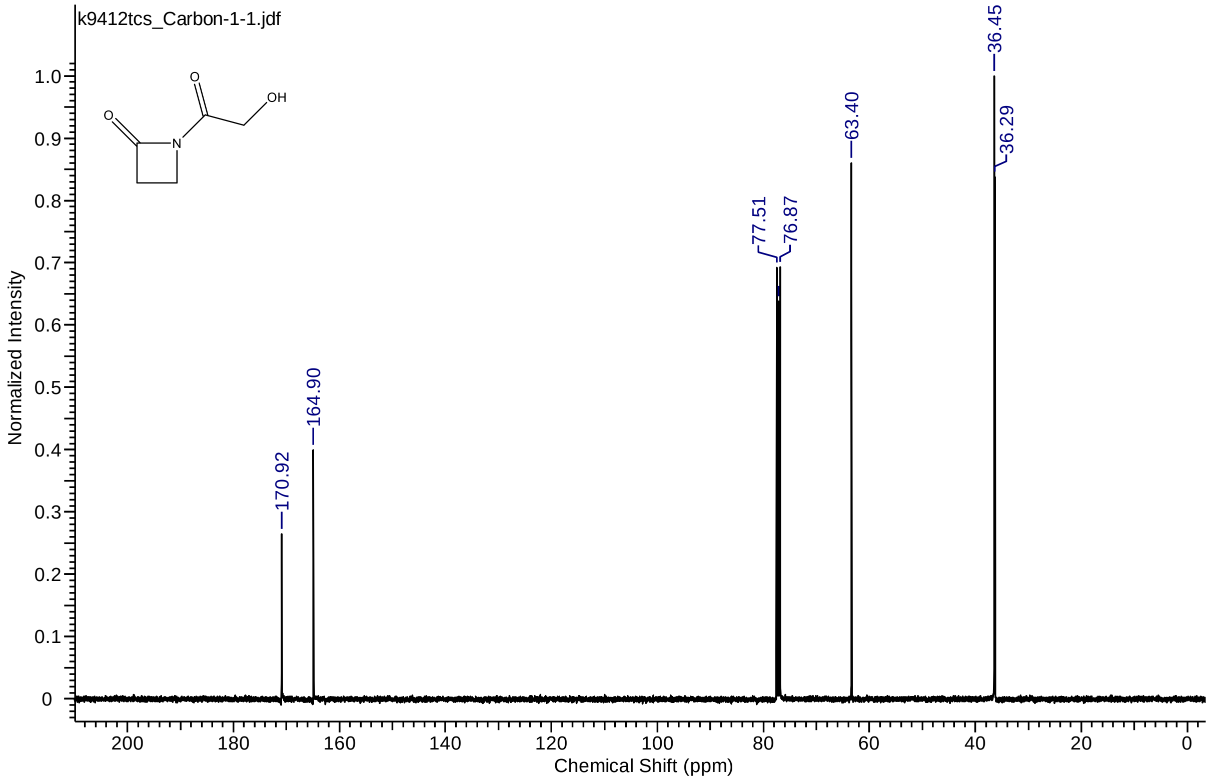

k9412tcs_Carbon-1-1.jdf
O
O
N
OH
170.92
164.90
77.51
76.87
63.40
36.45
36.29
200
180
160
140
120
100
80
60
40
20
0
Chemical Shift (ppm)
Normalized Intensity
0
0.1
0.2
0.3
0.4
0.5
0.6
0.7
0.8
0.9
1.0

Compound 8c

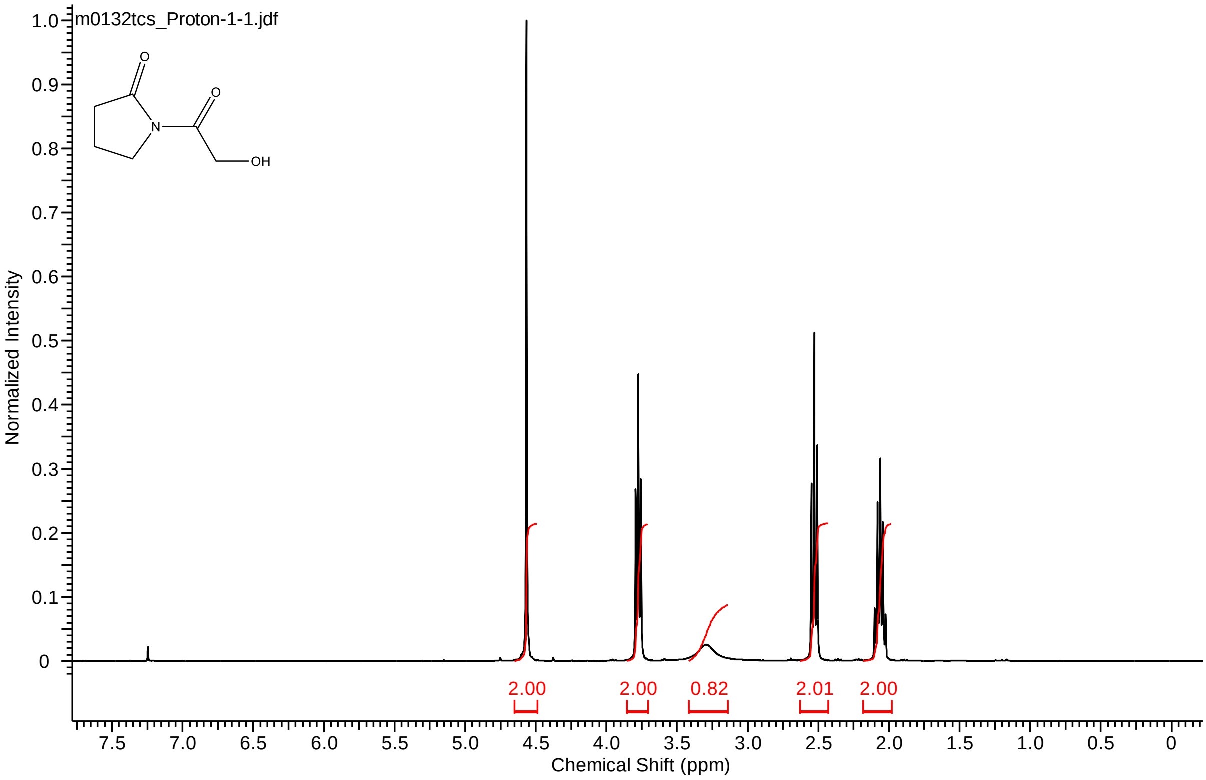
m0132tcs_Proton-1-1.jdf
Normalized Intensity
Chemical Shift (ppm)
2.00
2.00
0.82
2.01
2.00
O
O
N
OH

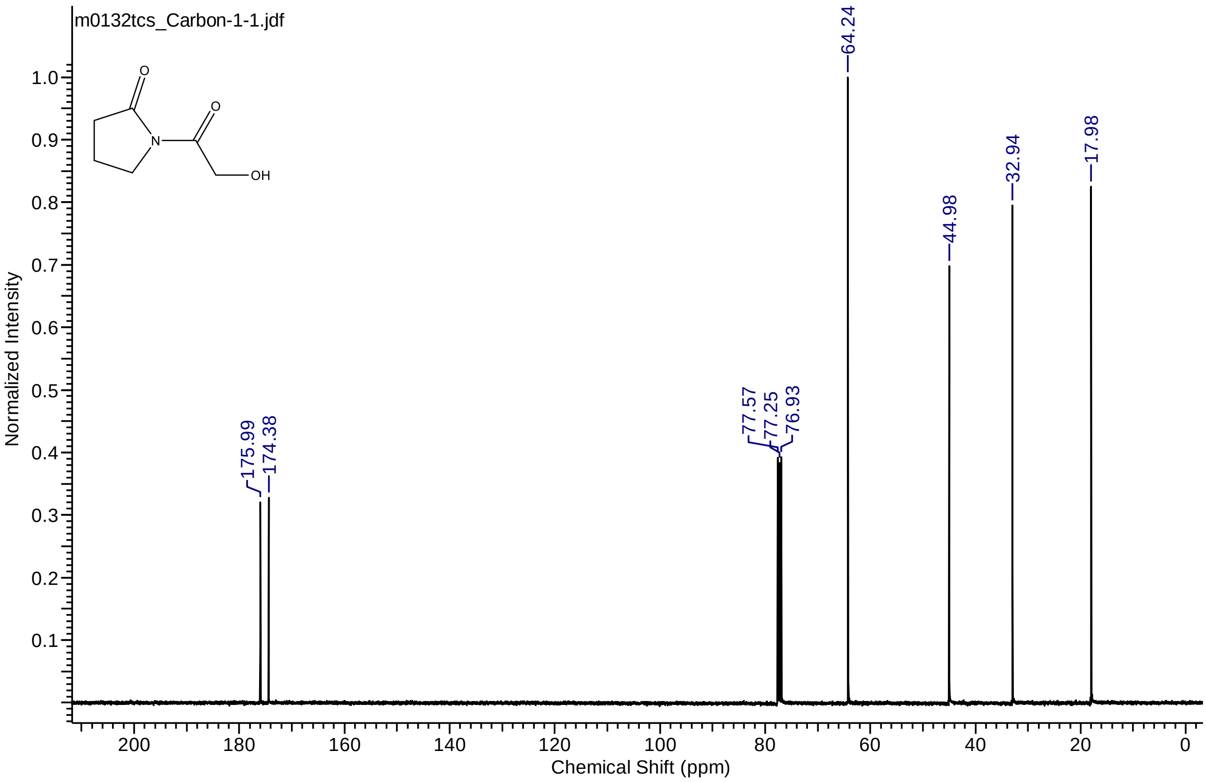
m0132tcs_Carbon-1-1.jdf
Normalized Intensity
Chemical Shift (ppm)
175.99
174.38
77.57
77.25
76.93
64.24
44.98
32.94
17.98
O
O
N
OH

Compound 10d

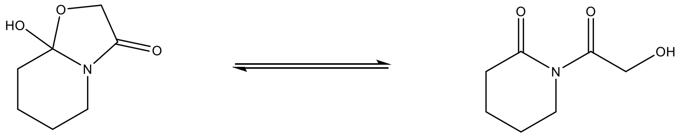

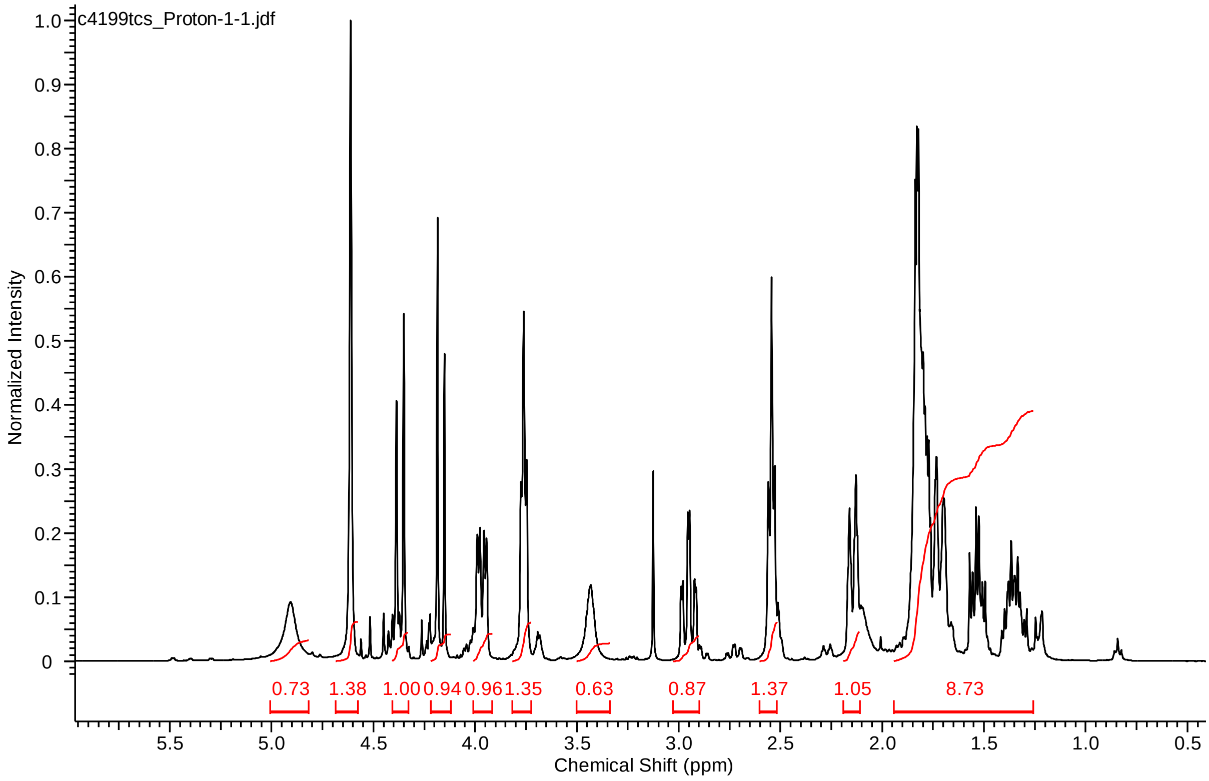

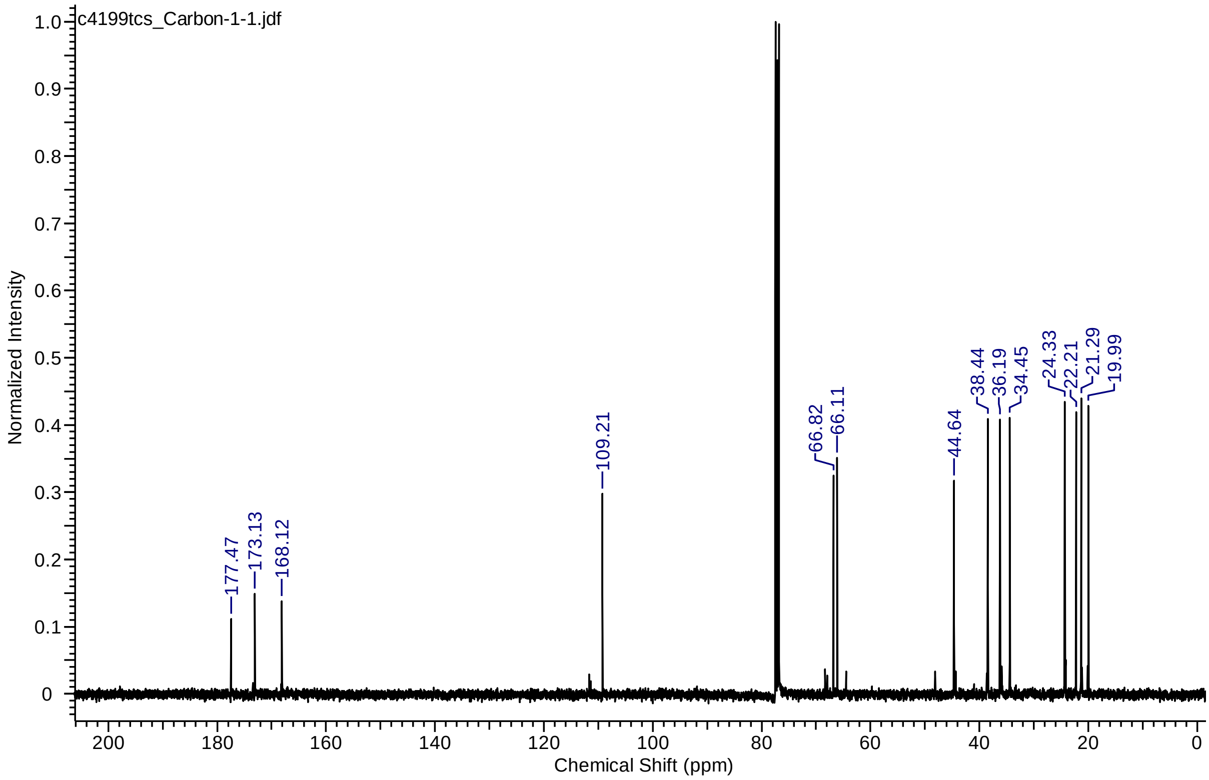

Compound 10e

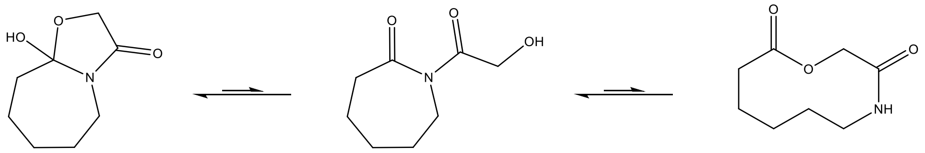

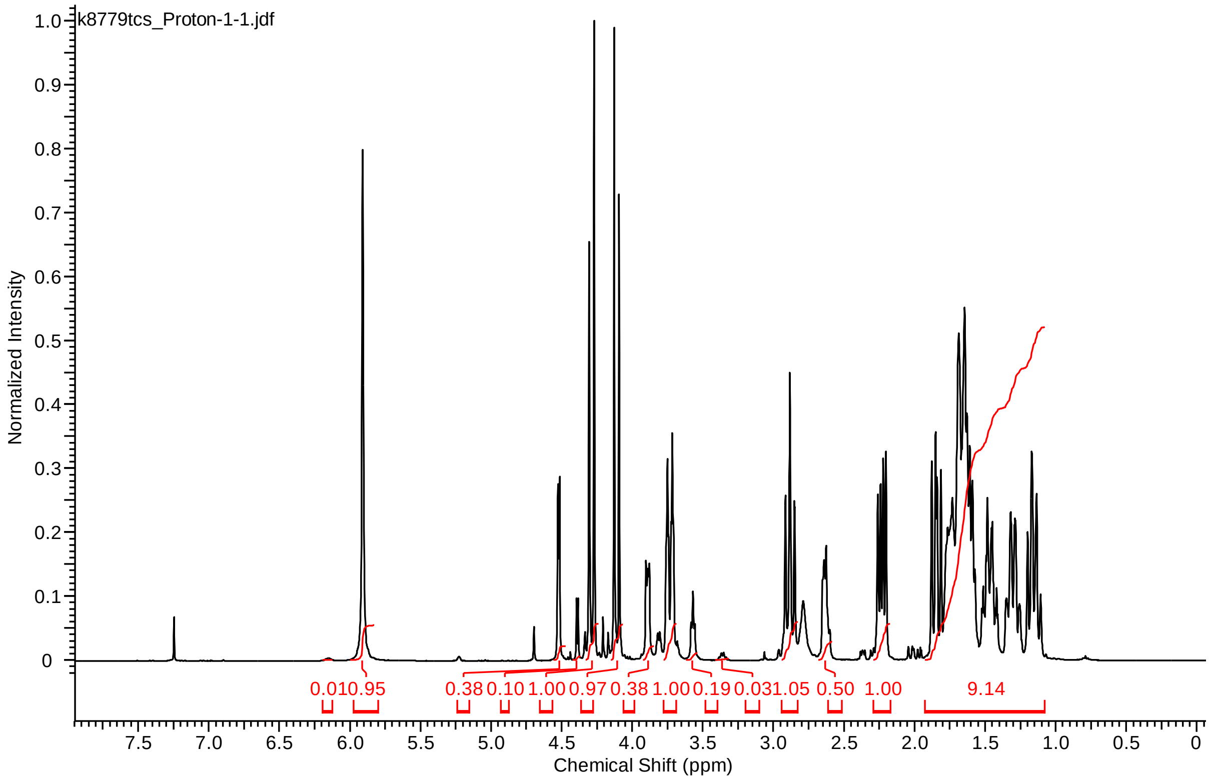

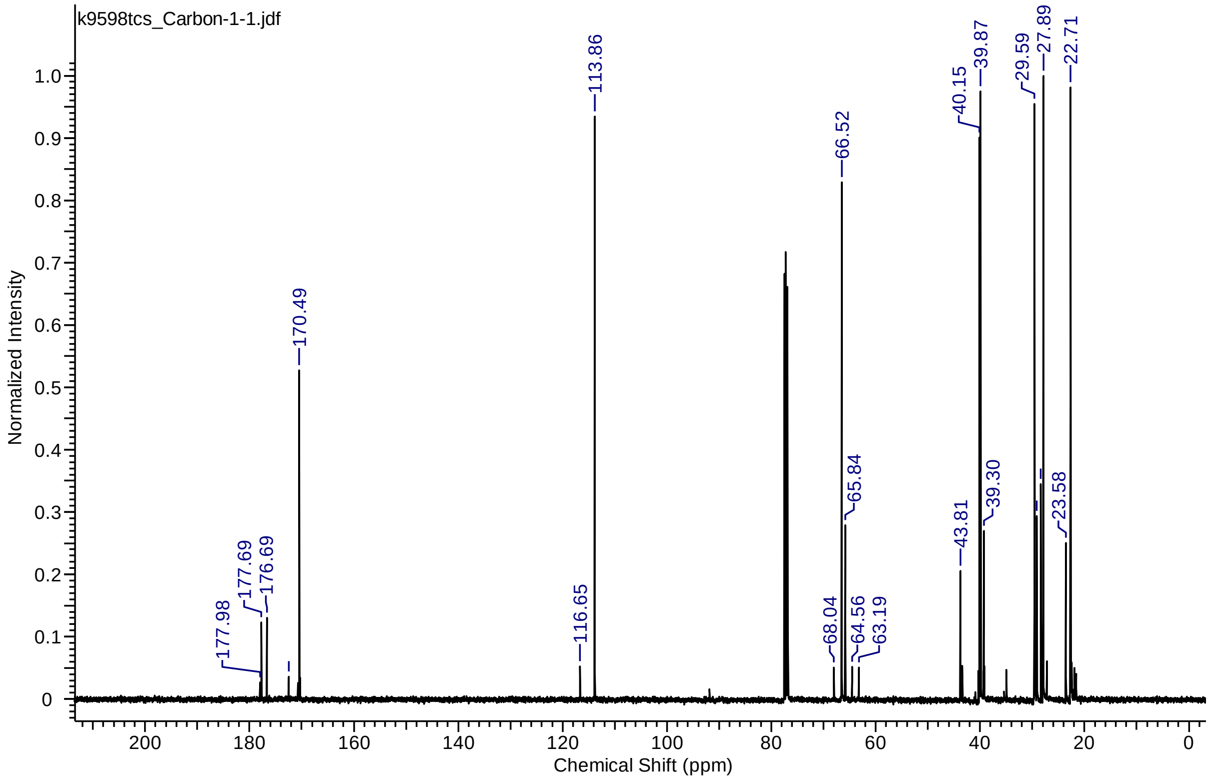

Compound 12f

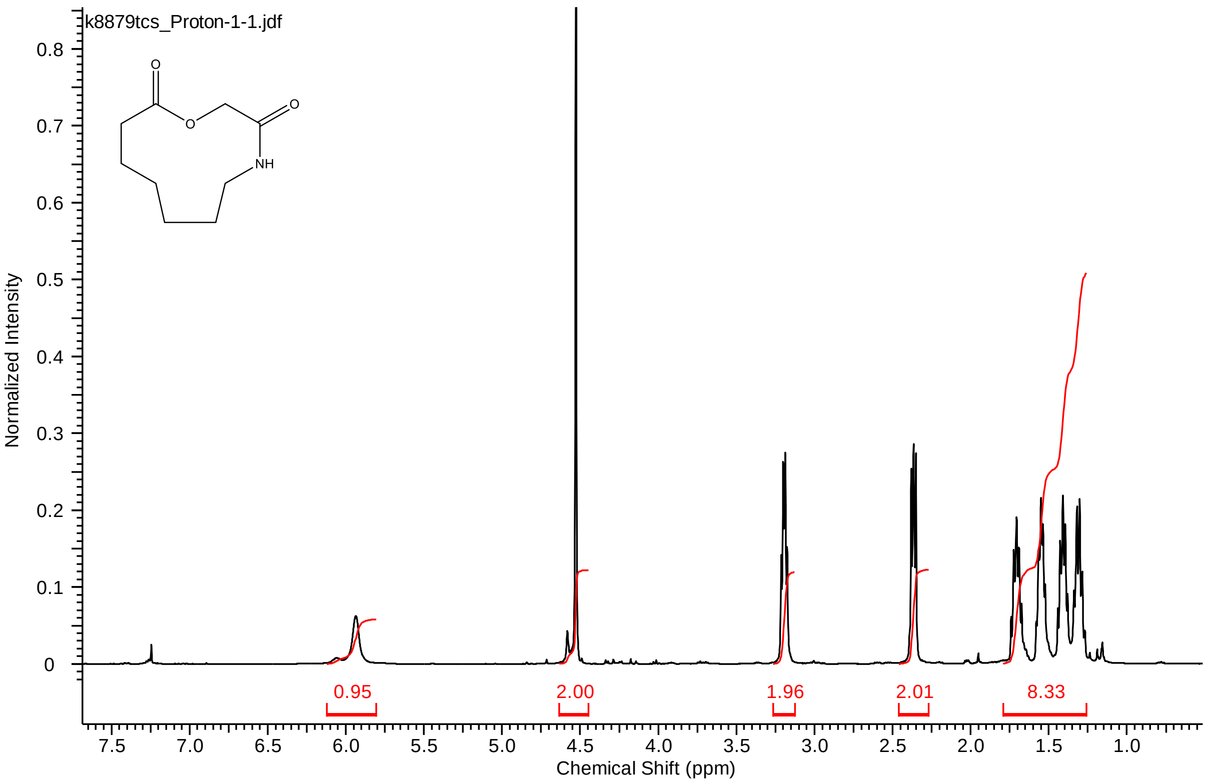

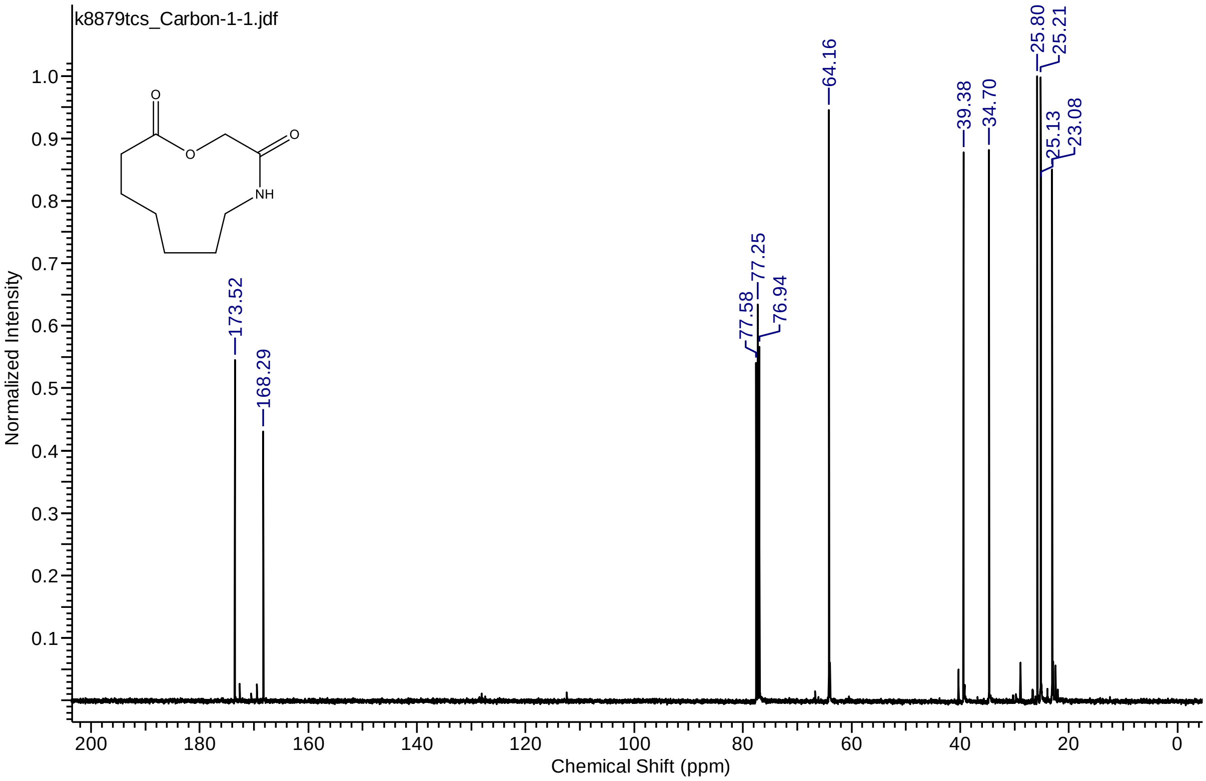

Compound 12g

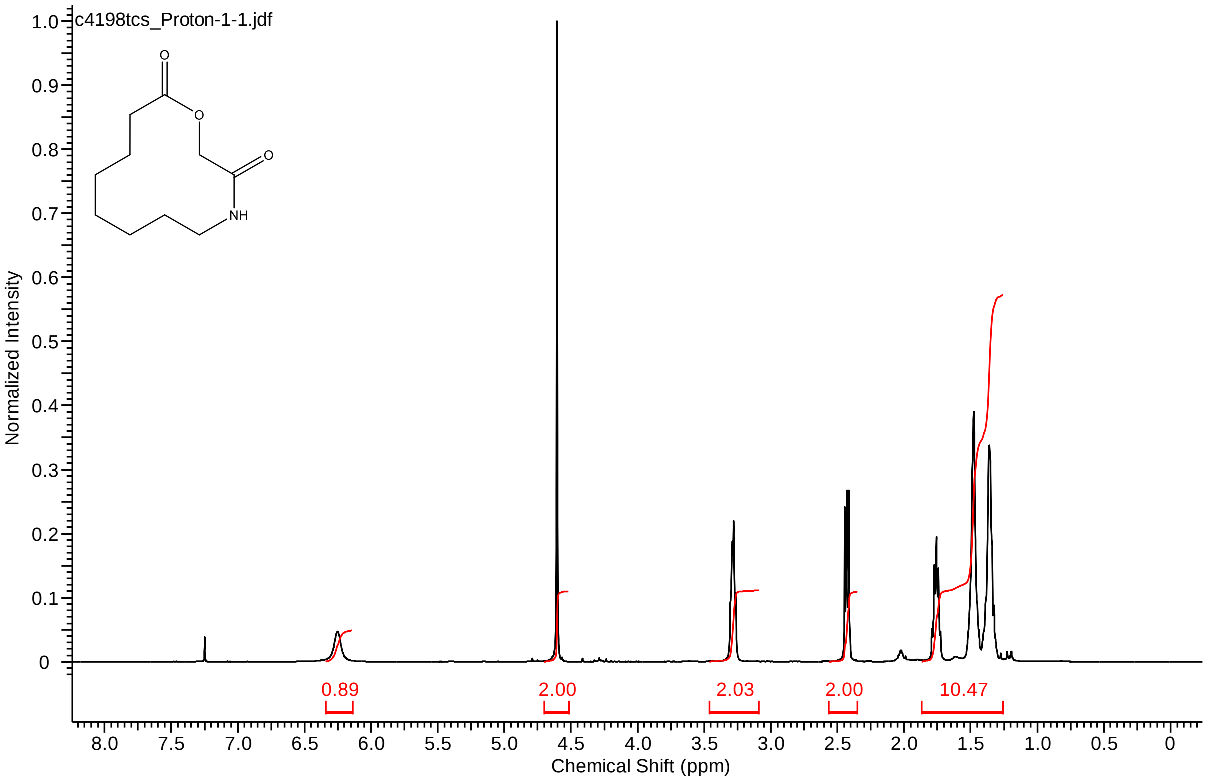

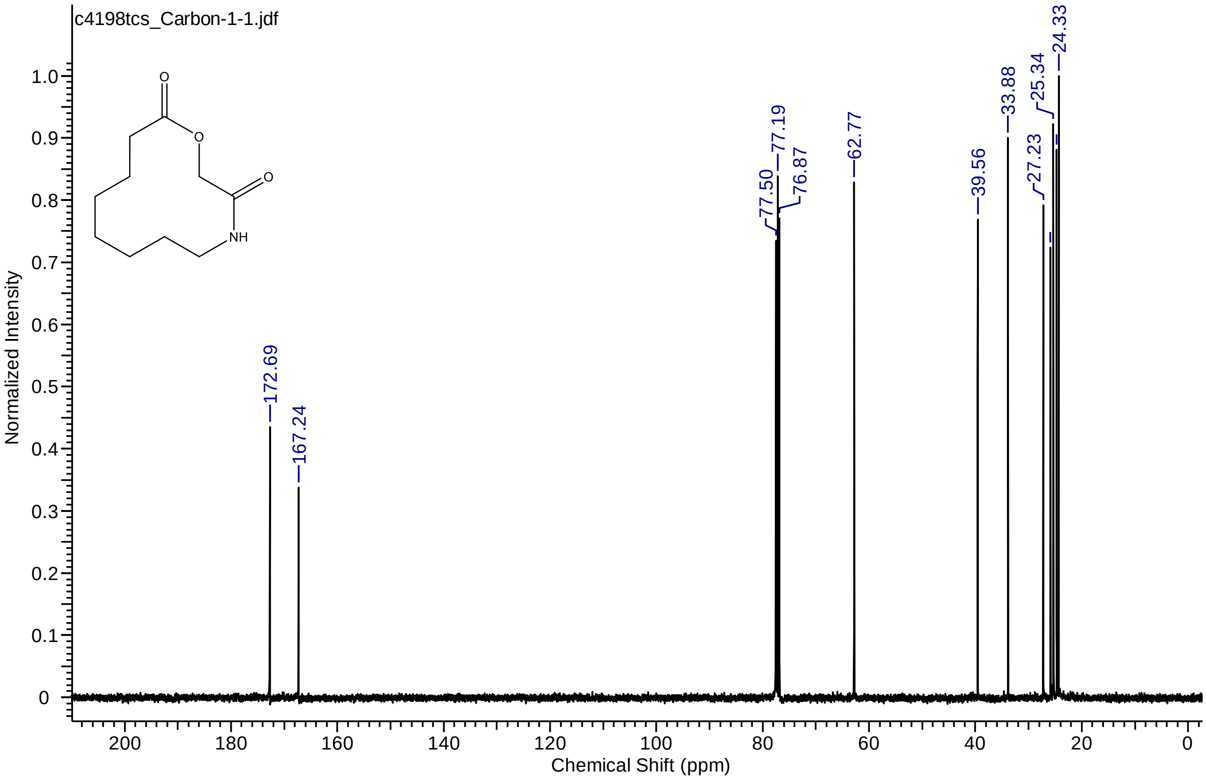

Compound 12h

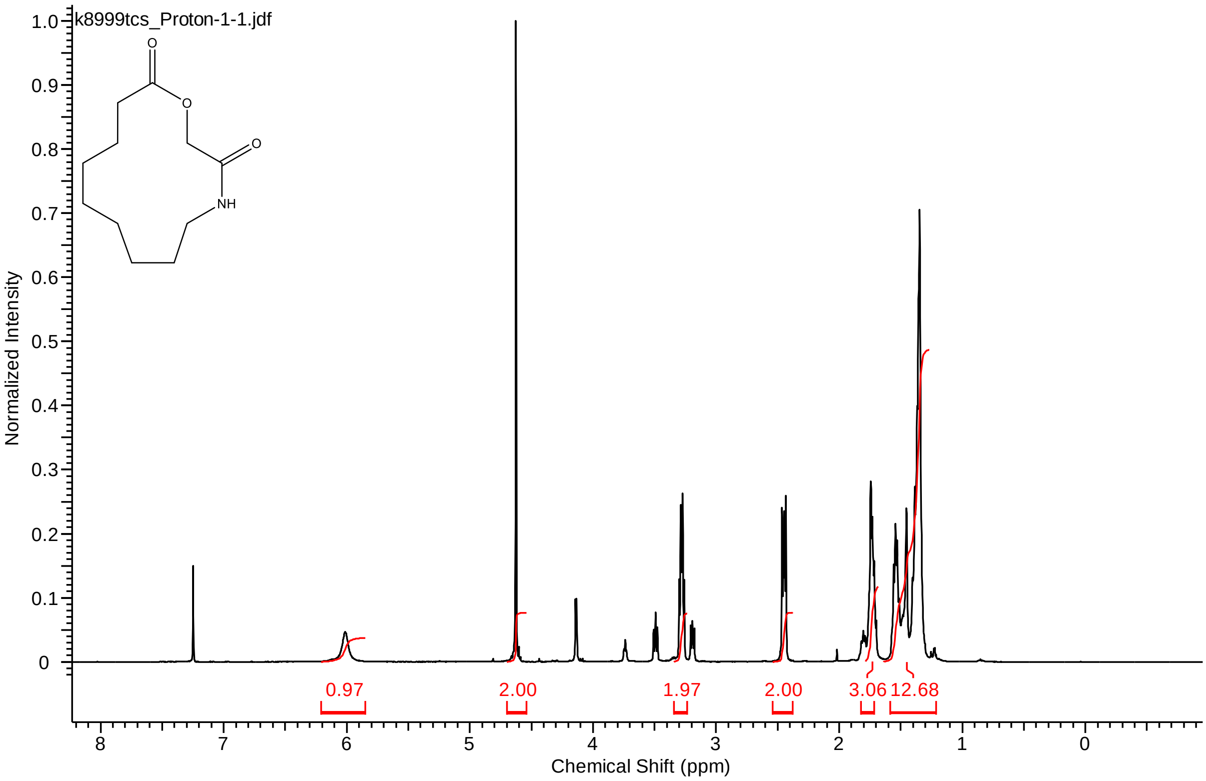

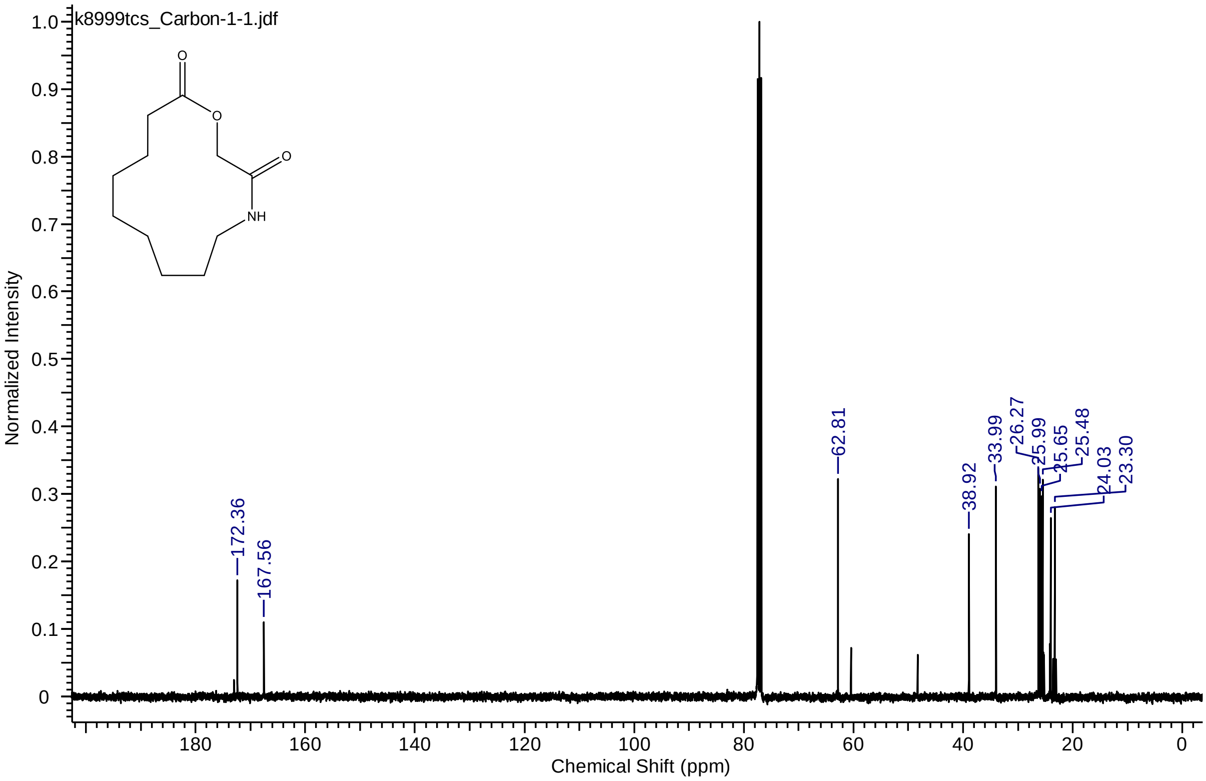

Compound 12i

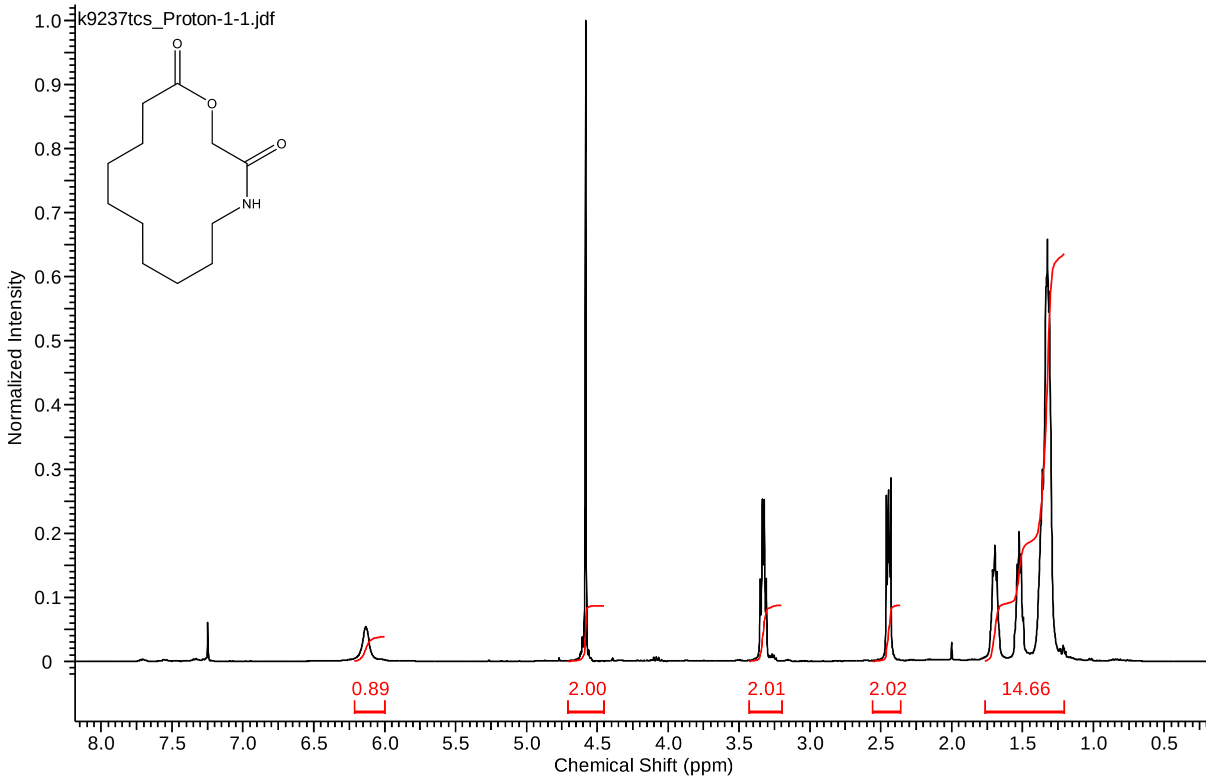

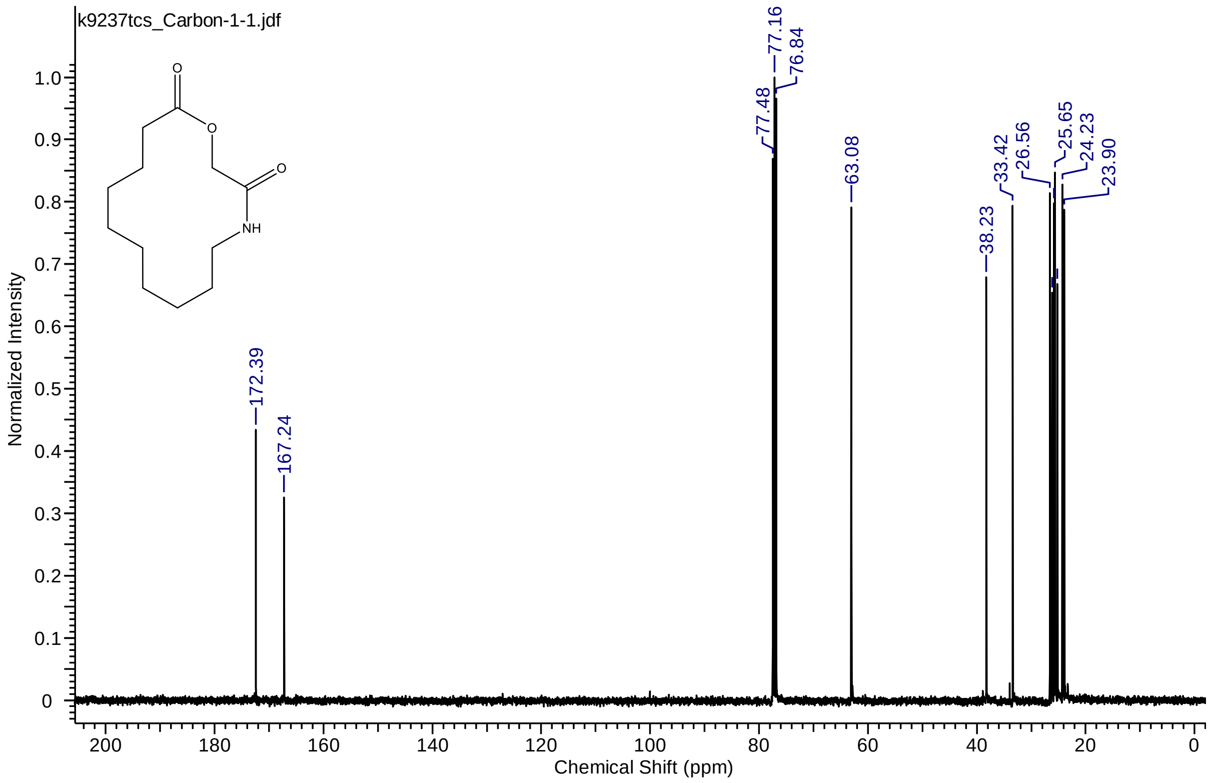

Compound 12j

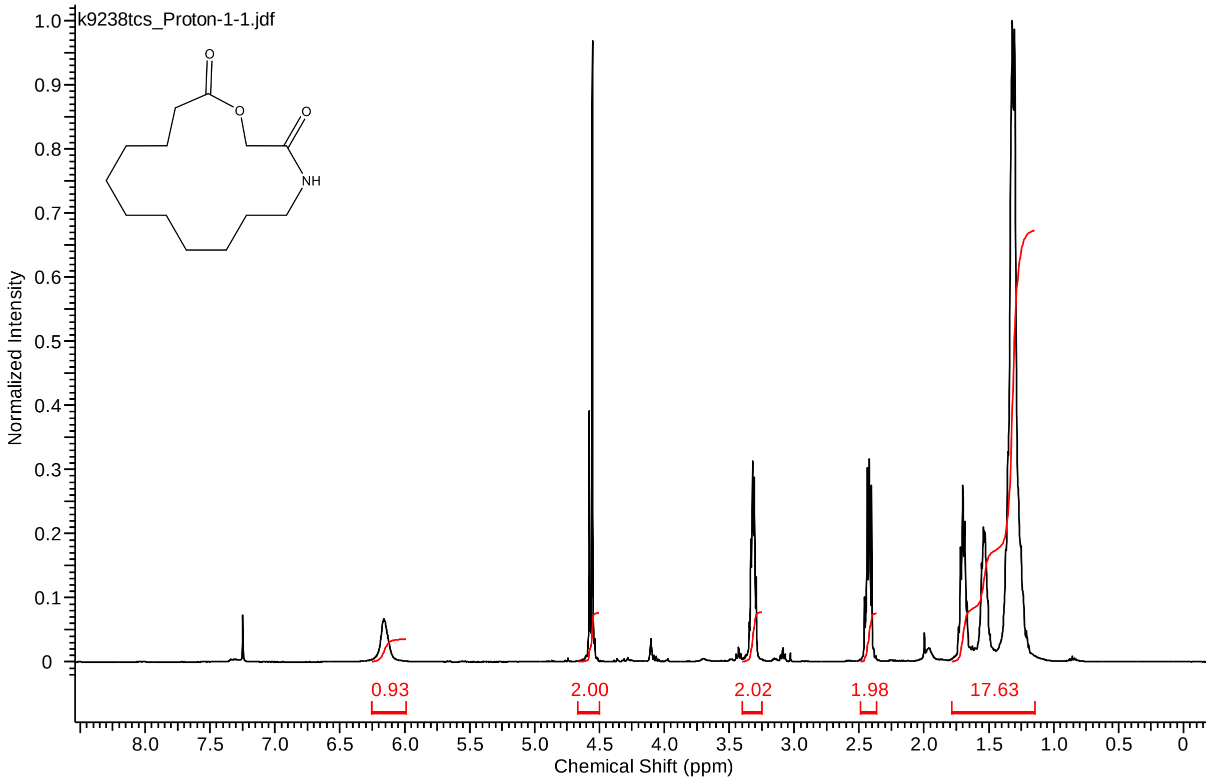

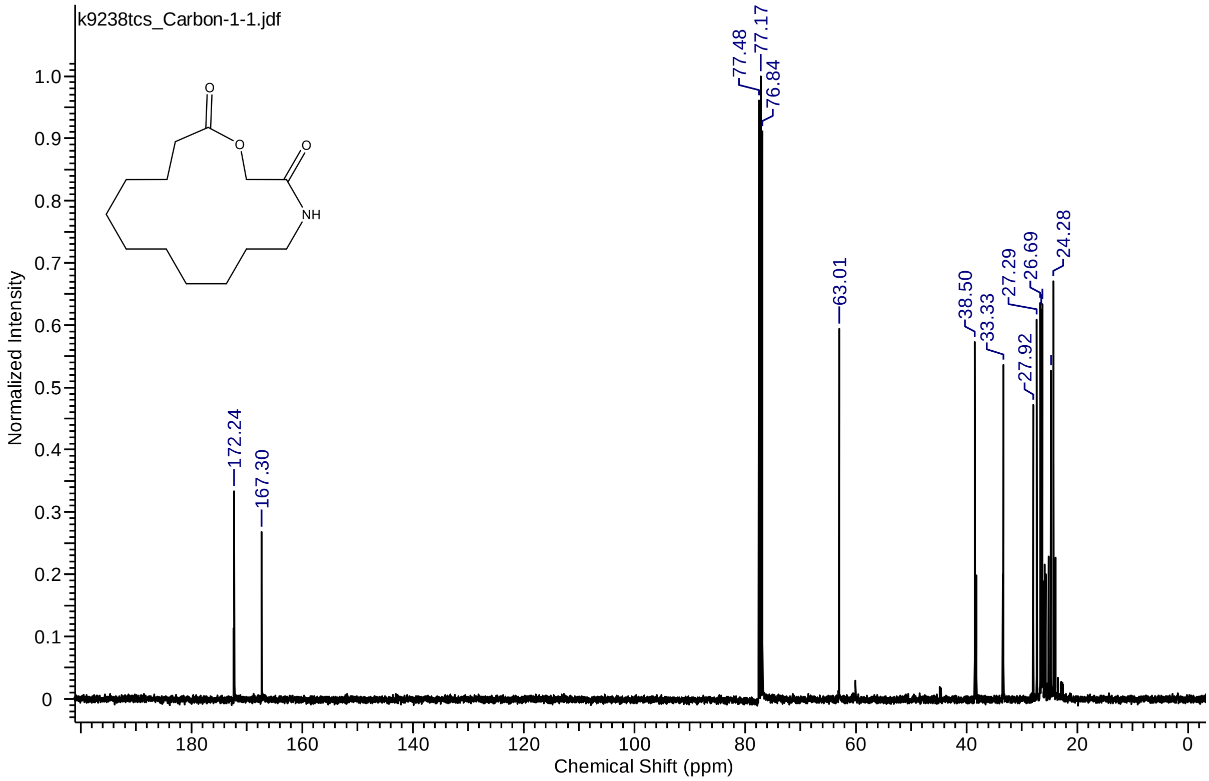

Compound 13a

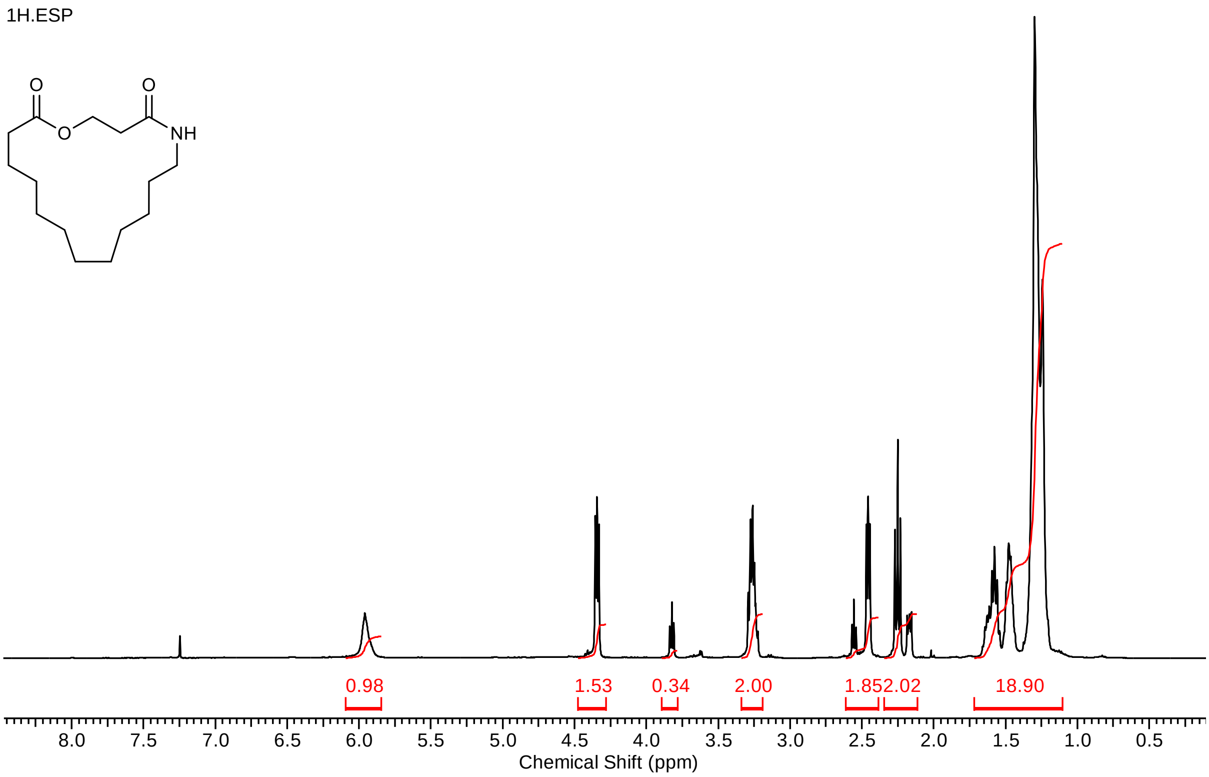

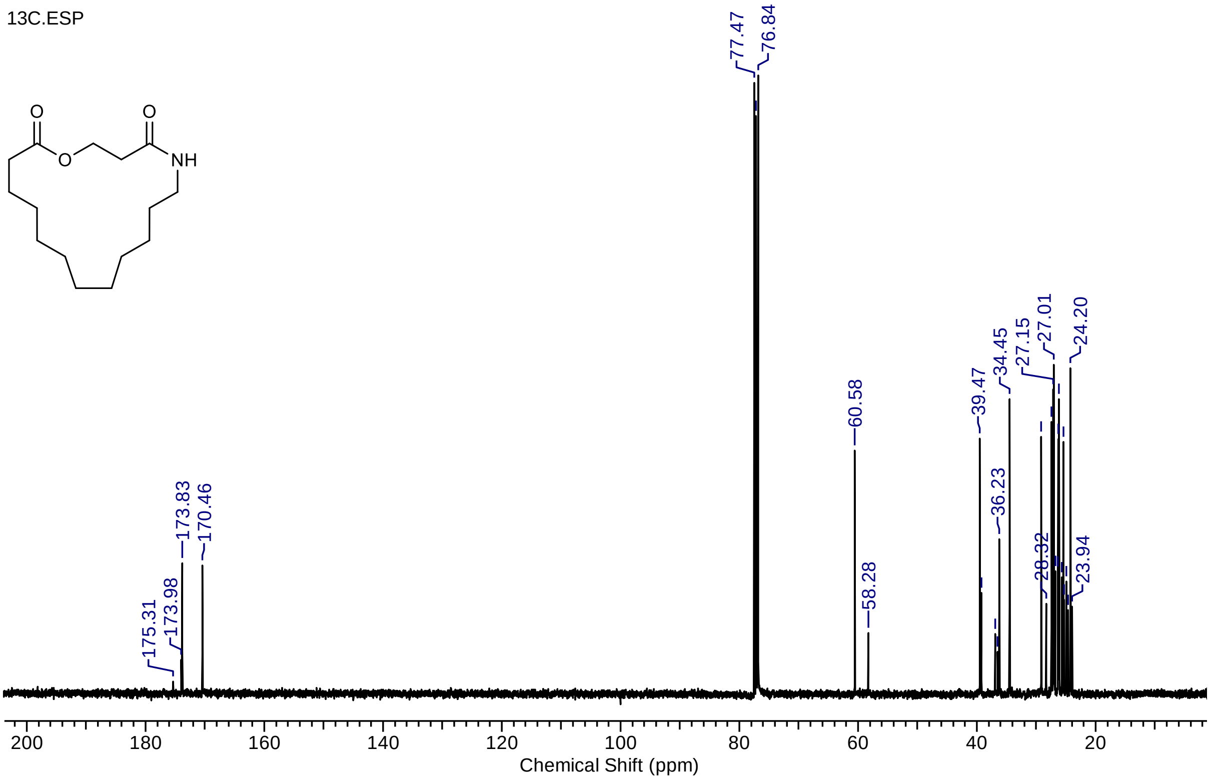

Compound 9b

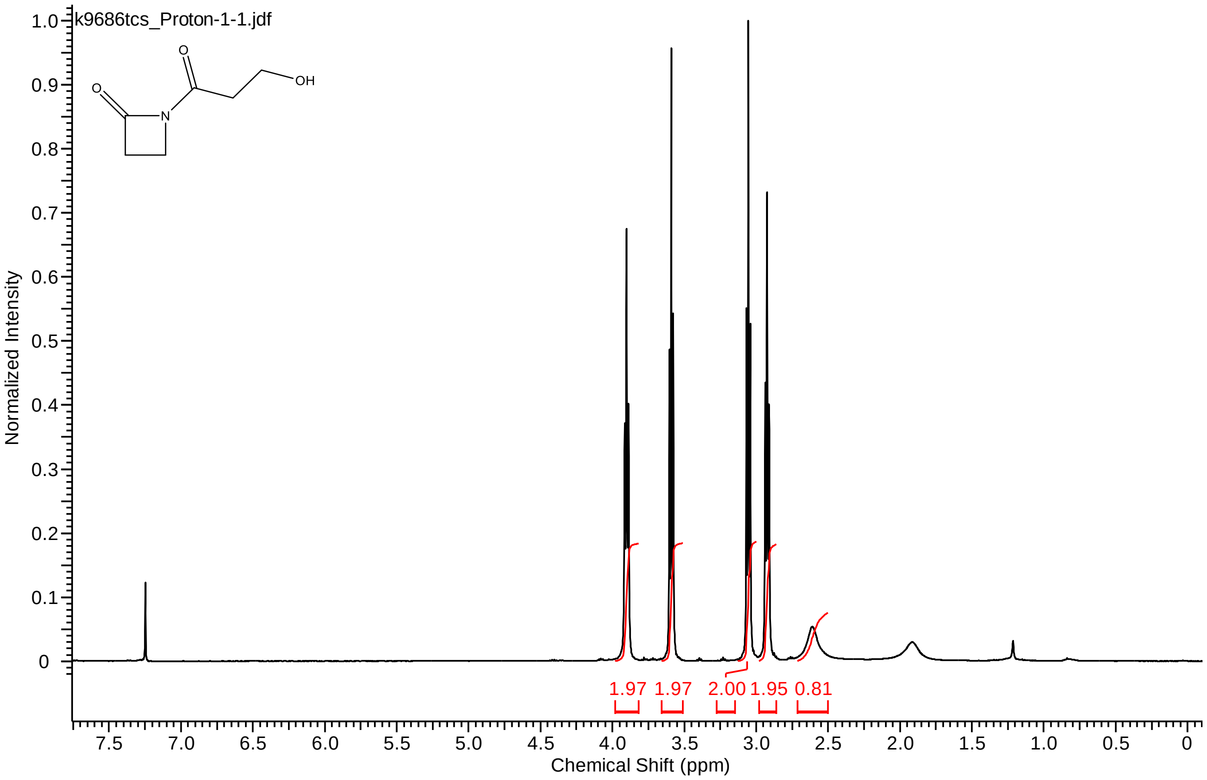

k9686tcs_Proton-1-1.jdf
O
O
N
OH
Normalized Intensity
1.97 1.97 2.00 1.95 0.81
Chemical Shift (ppm)

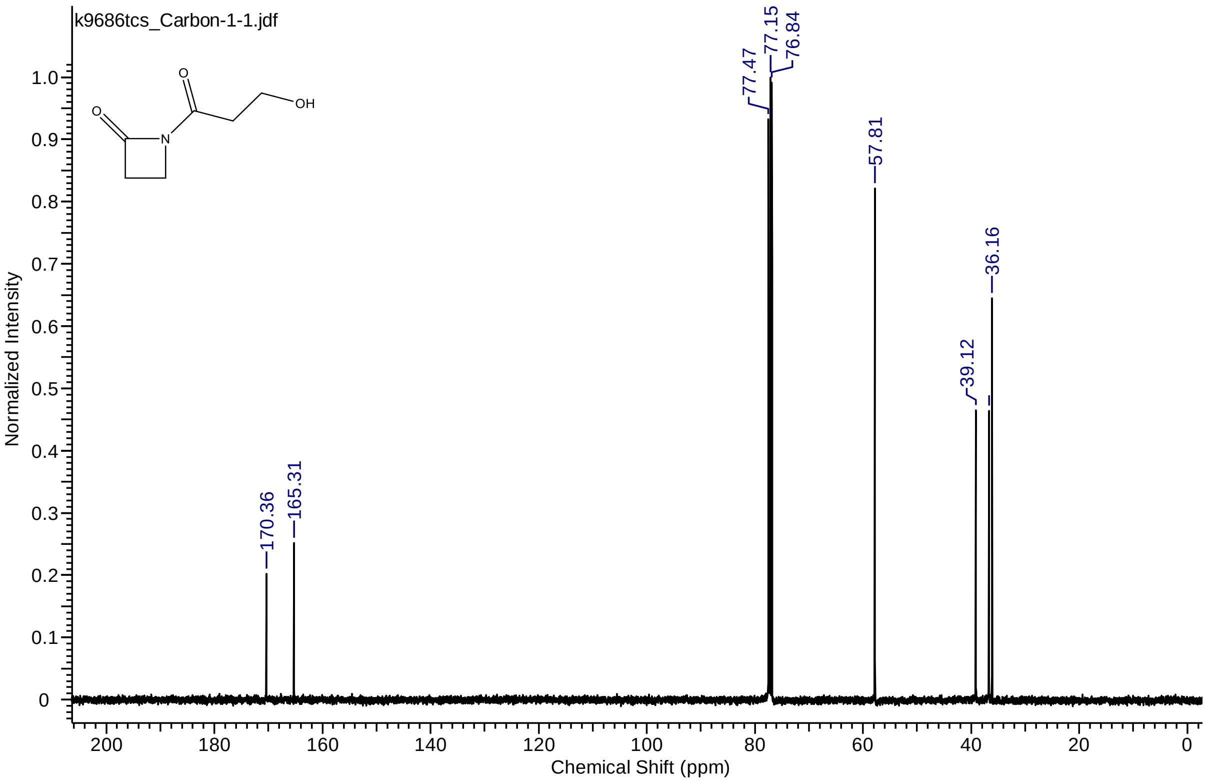

k9686tcs_Carbon-1-1.jdf
O
O
N
OH
Normalized Intensity
77.47
77.15
76.84
57.81
36.16
39.12
170.36
165.31
Chemical Shift (ppm)

Compound 9c

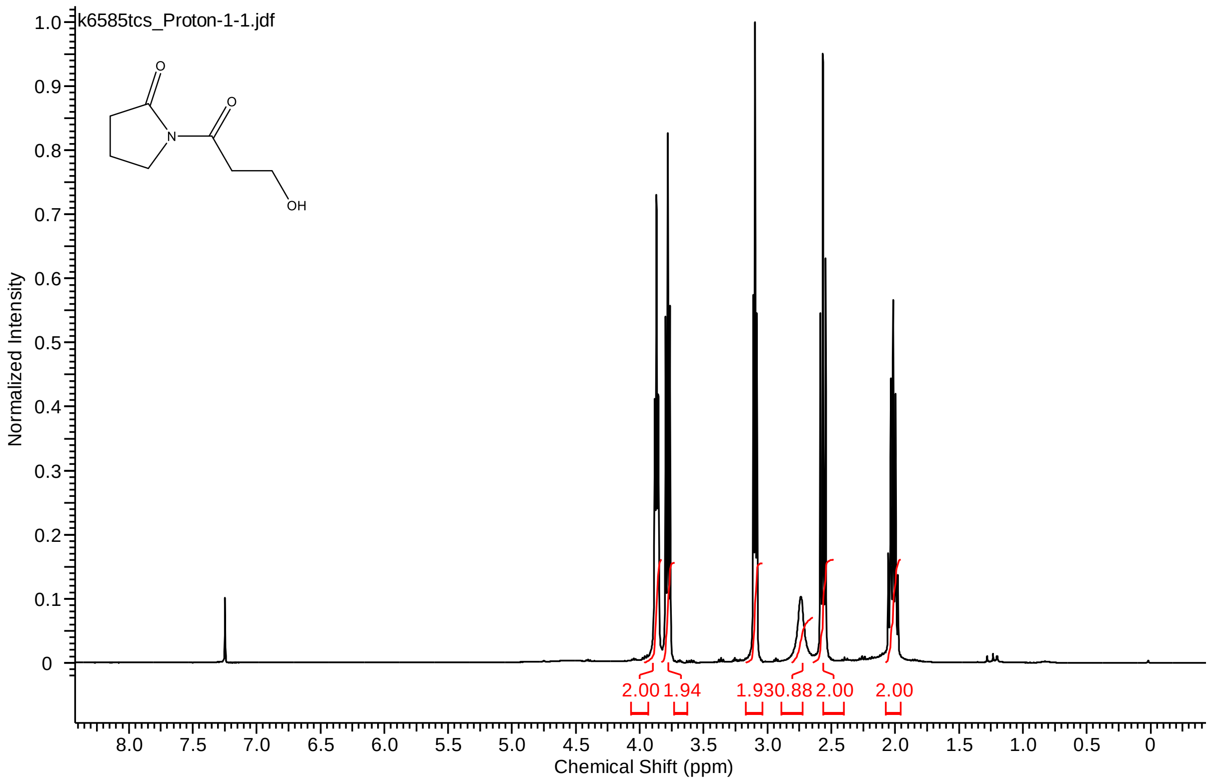

k6585tcs_Proton-1-1.jdf
Normalized Intensity
Chemical Shift (ppm)
2.00 1.94 1.93 0.88 2.00 2.00
O
N
O
OH

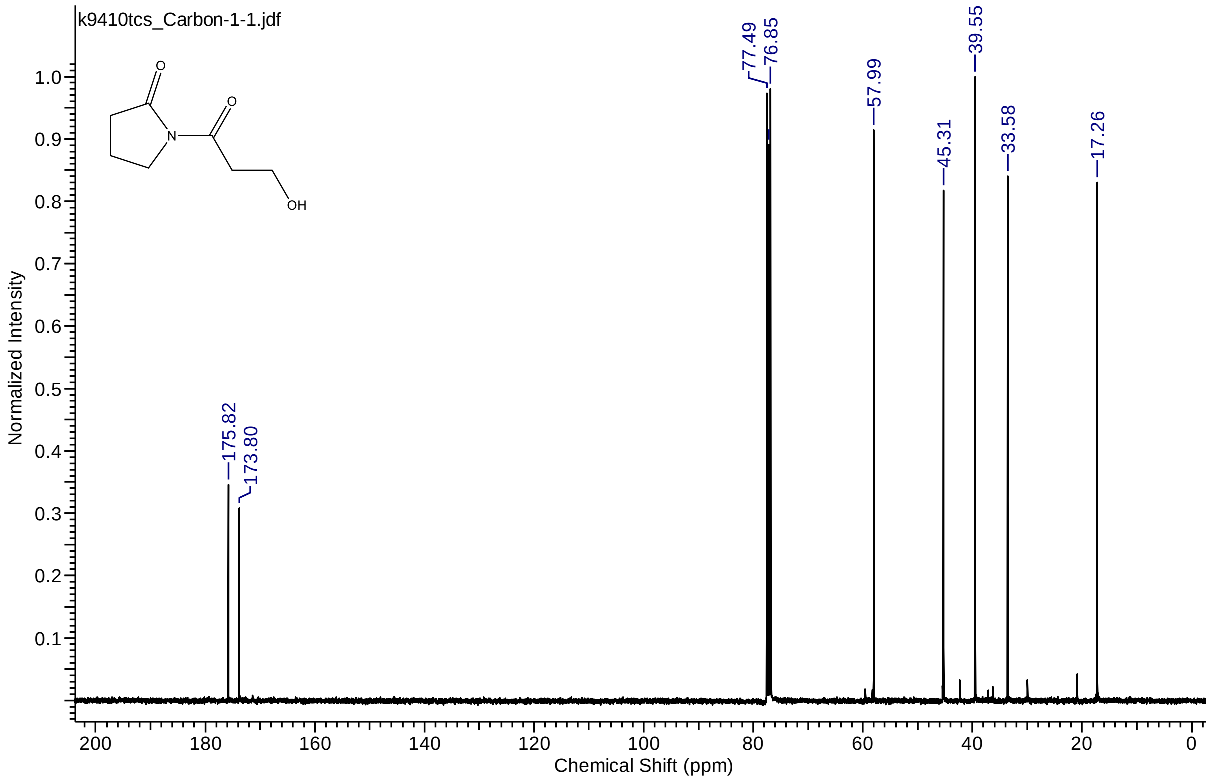

k9410tcs_Carbon-1-1.jdf
Normalized Intensity
Chemical Shift (ppm)
175.82
173.80
77.49
76.85
57.99
45.31
39.55
33.58
17.26
O
N
O
OH

Compound 13d

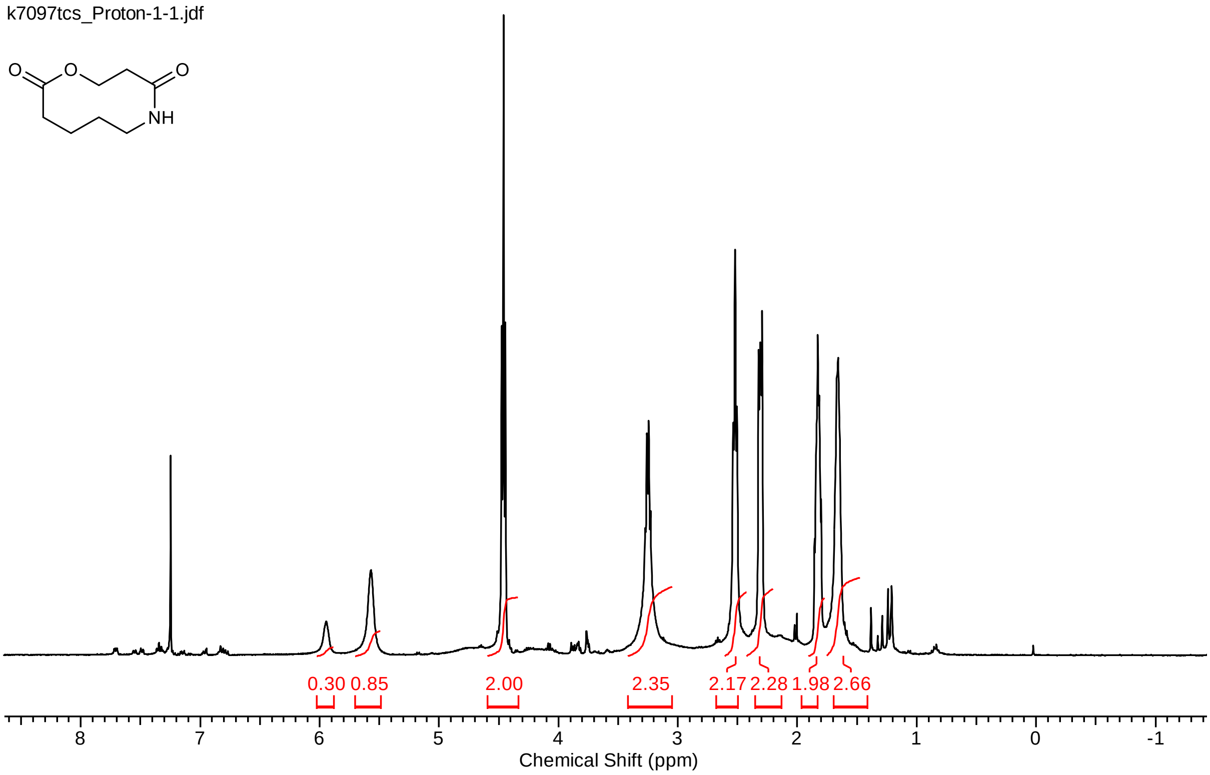

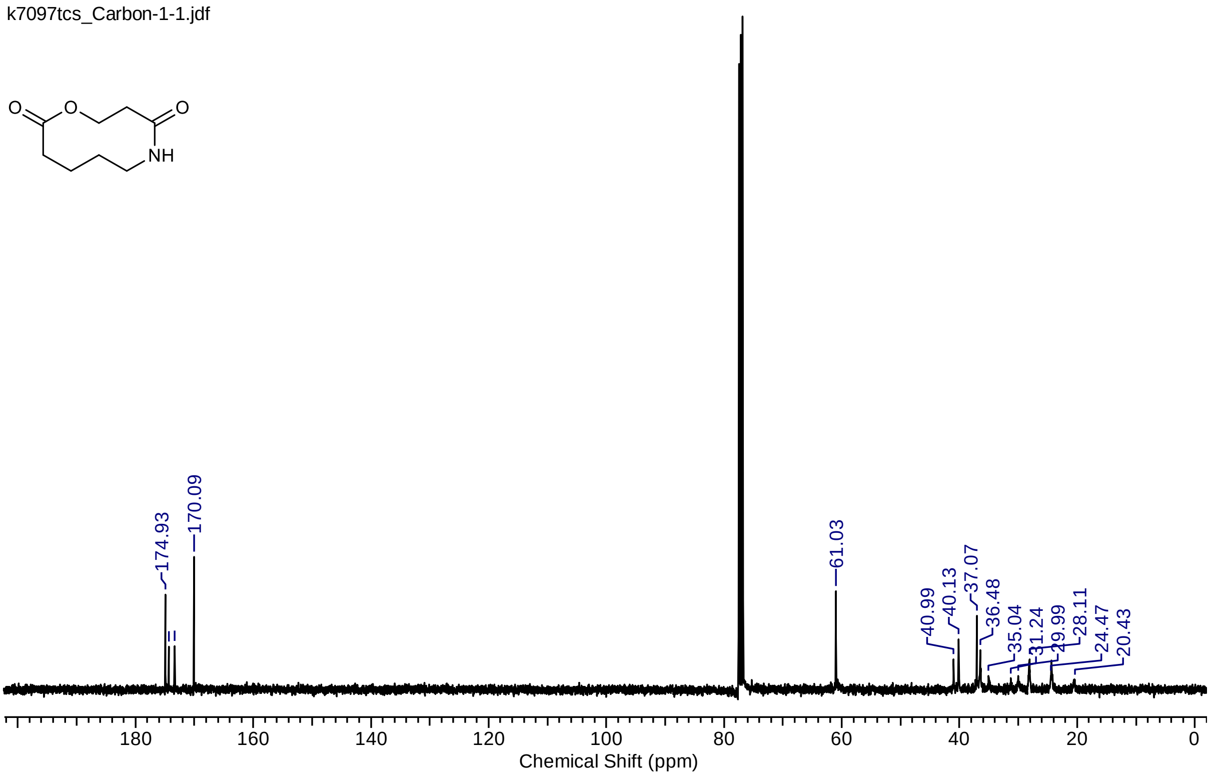

Compound 13e

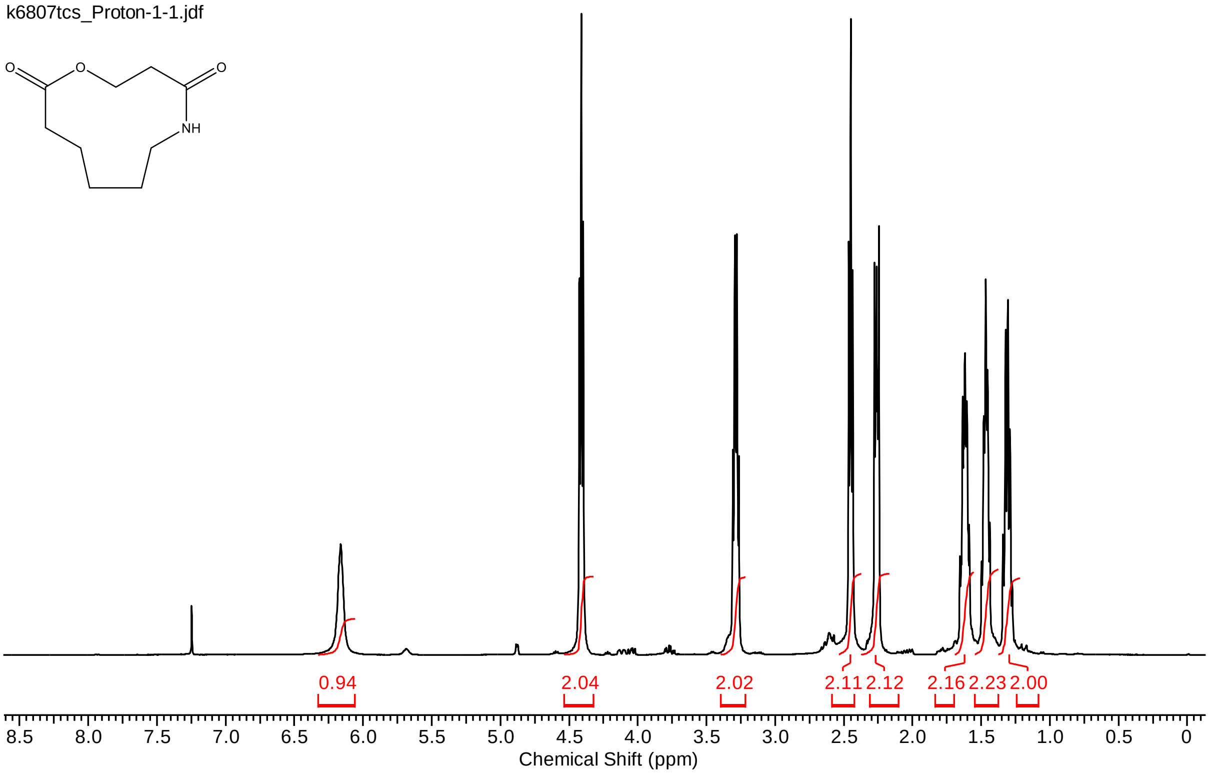

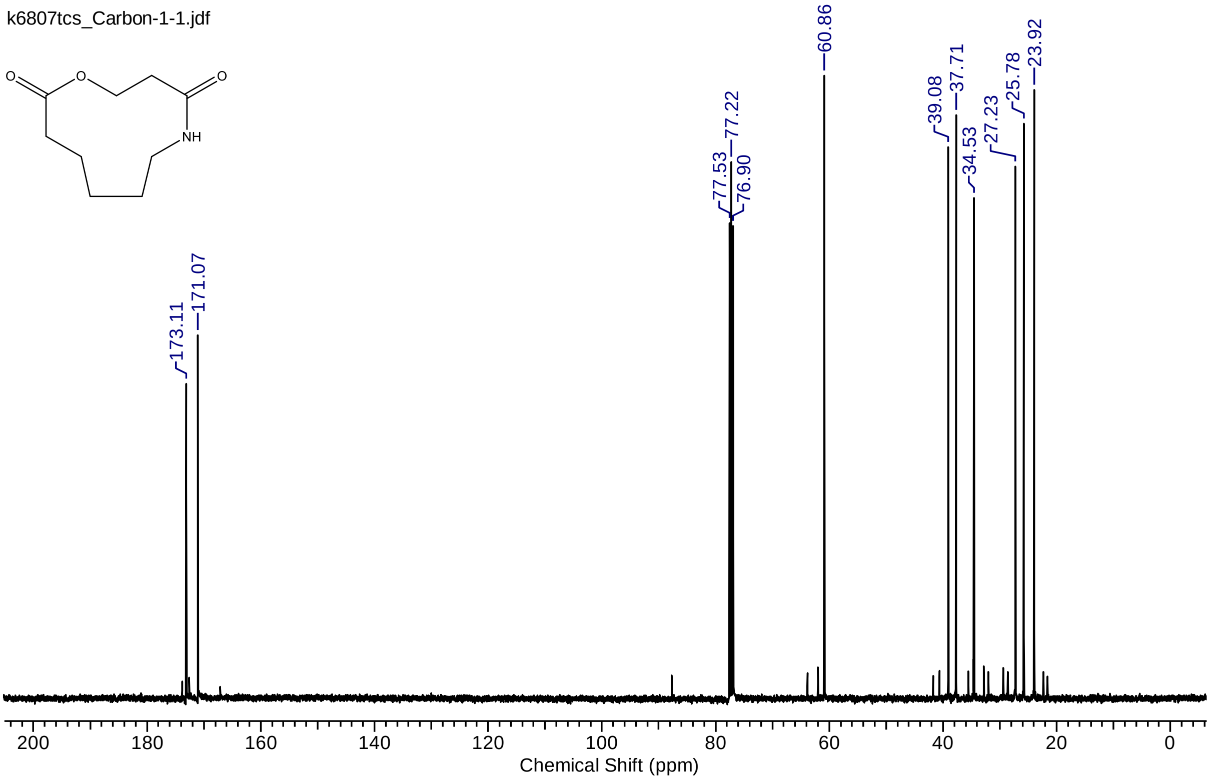

Compound 13f

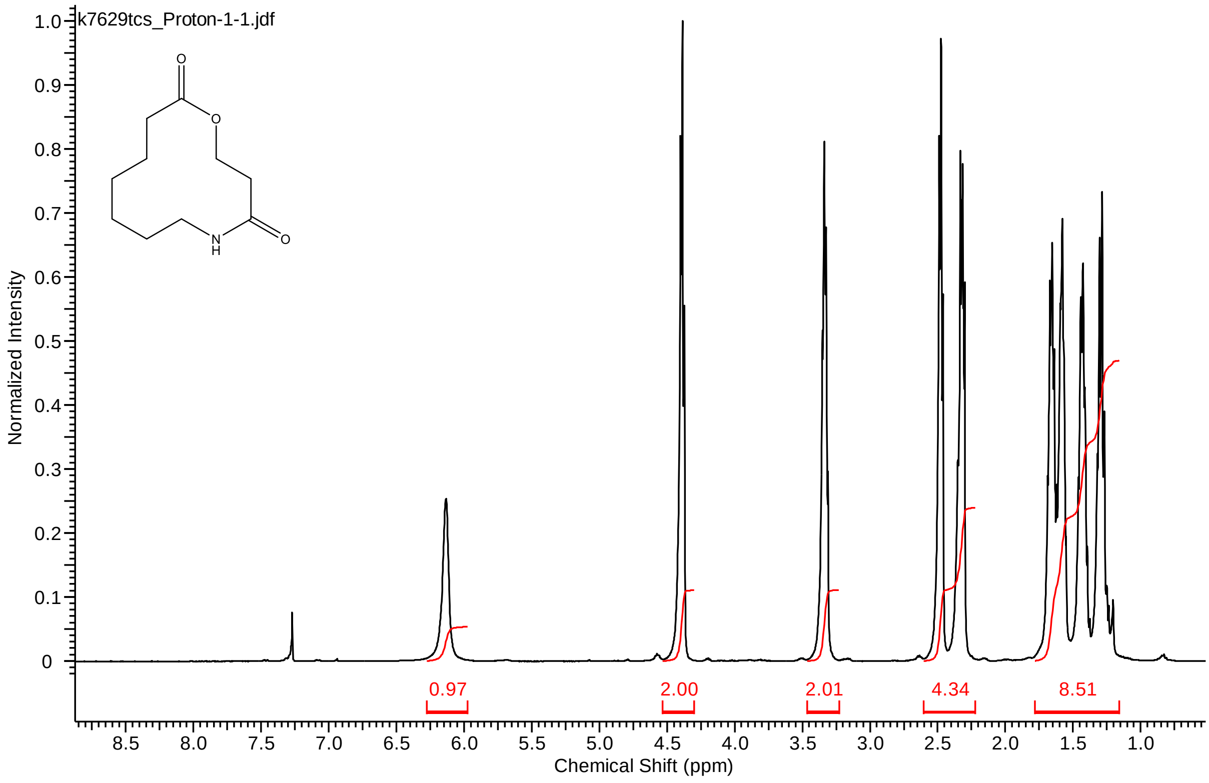

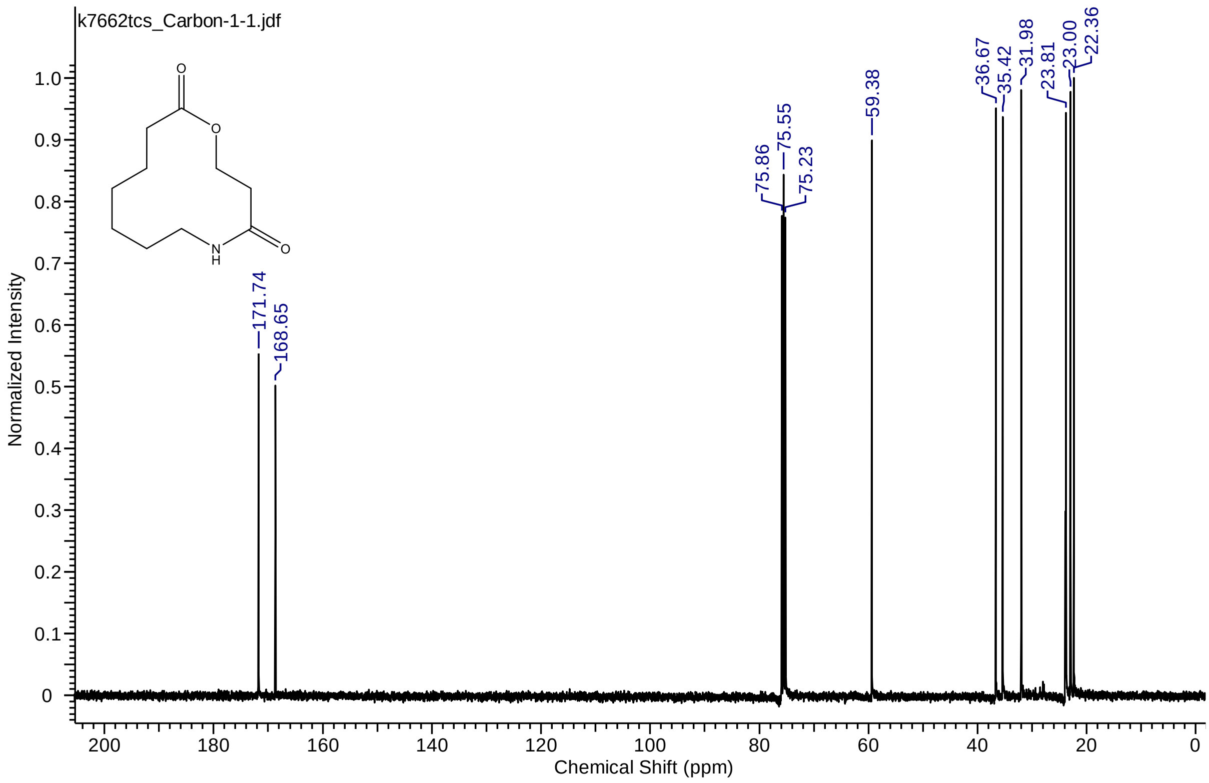

Compound 13g

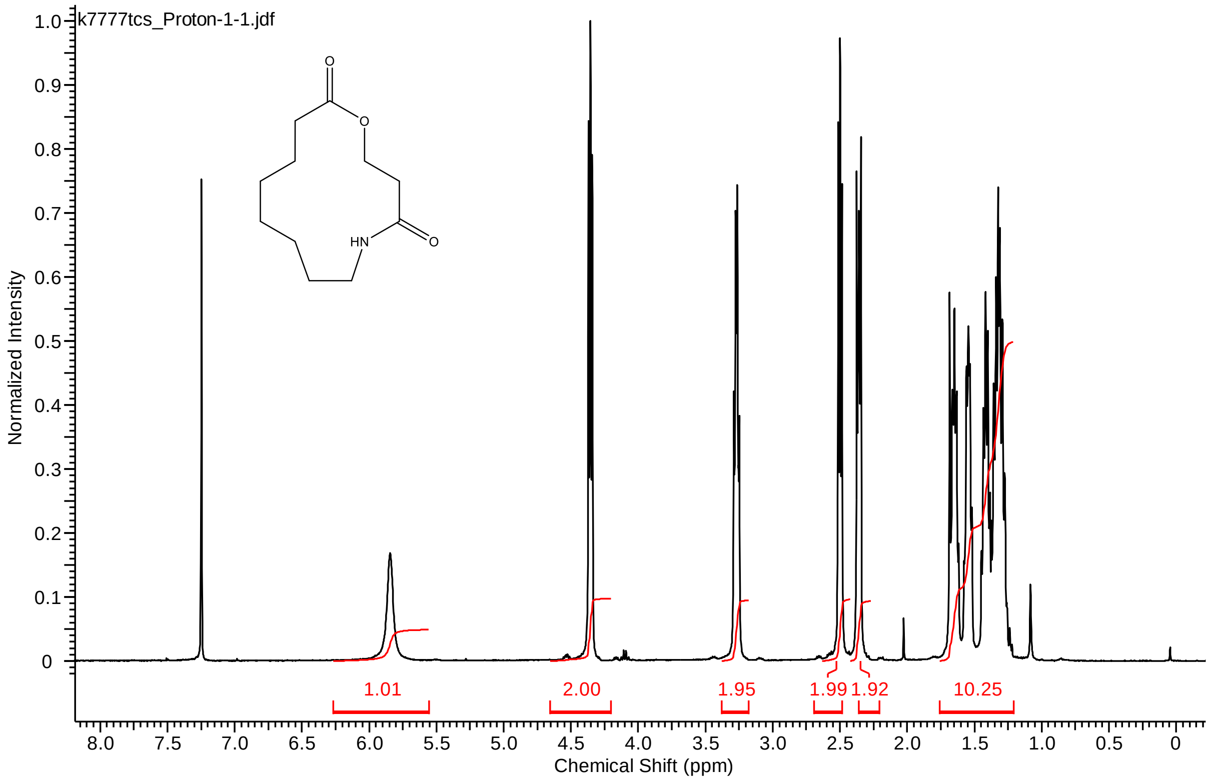

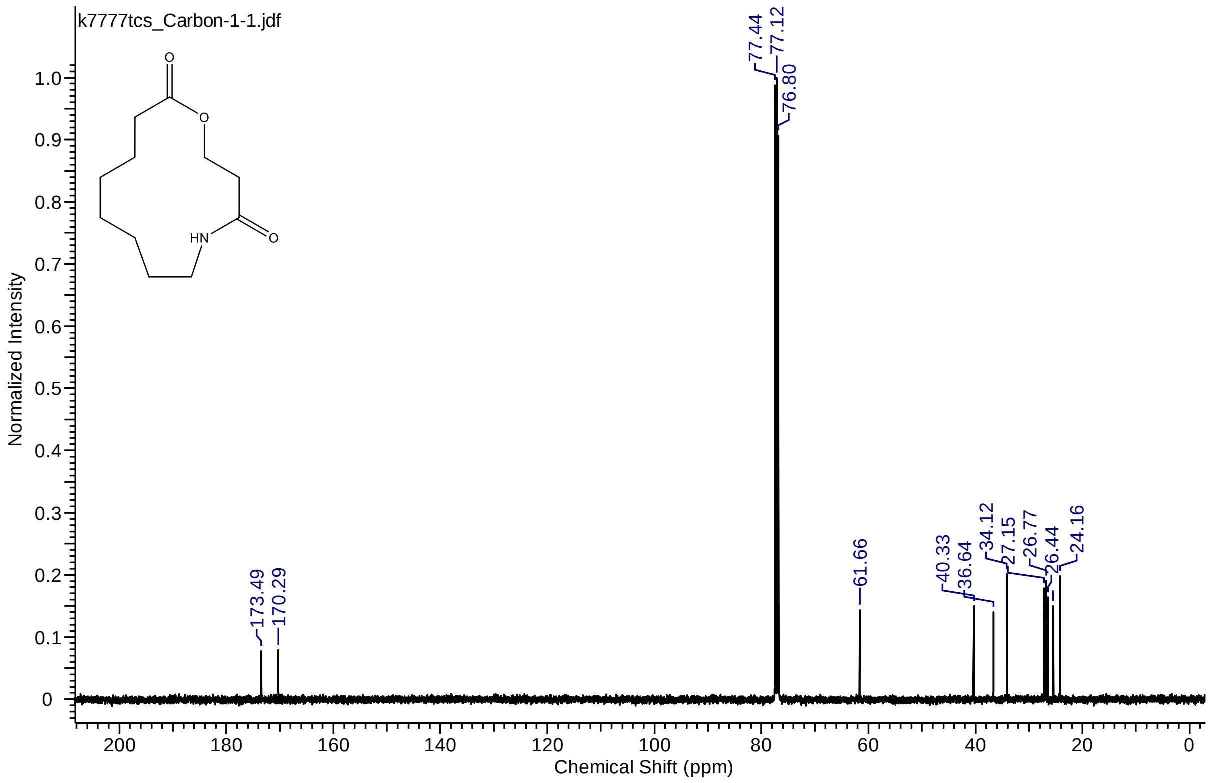

Compound 13h

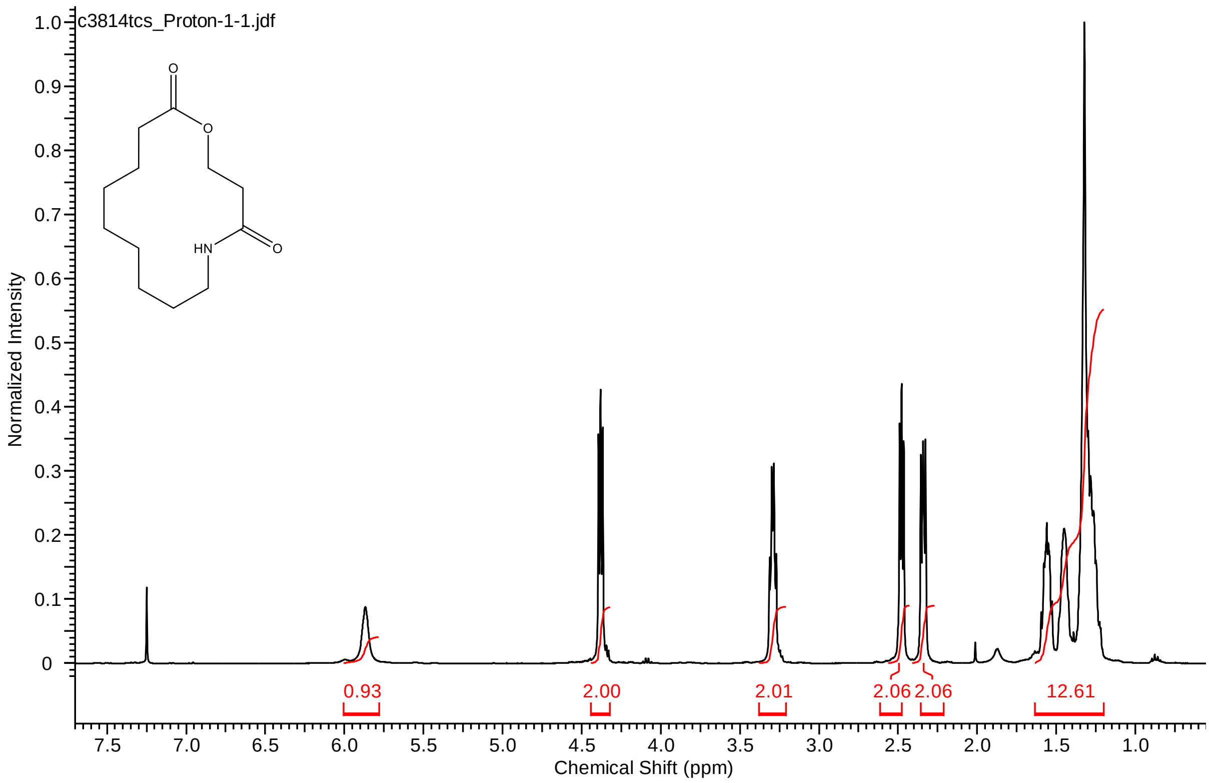

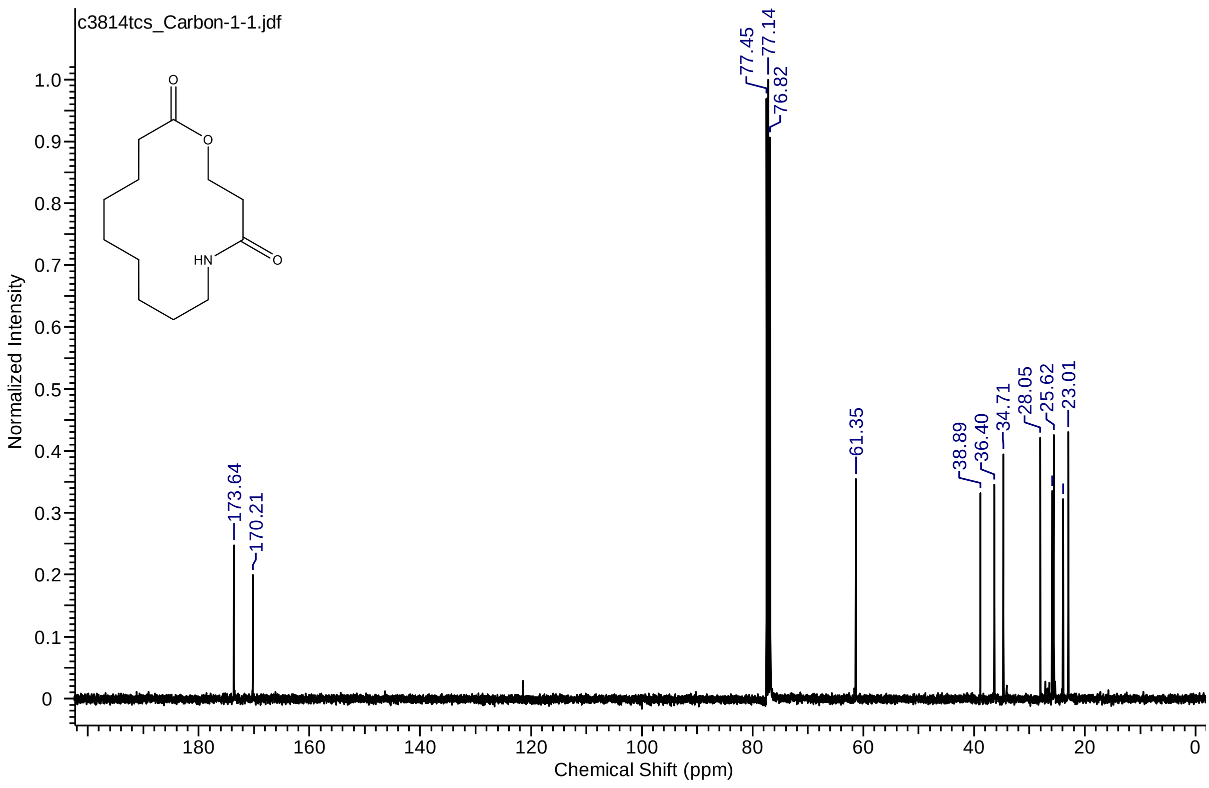

Compound 13i

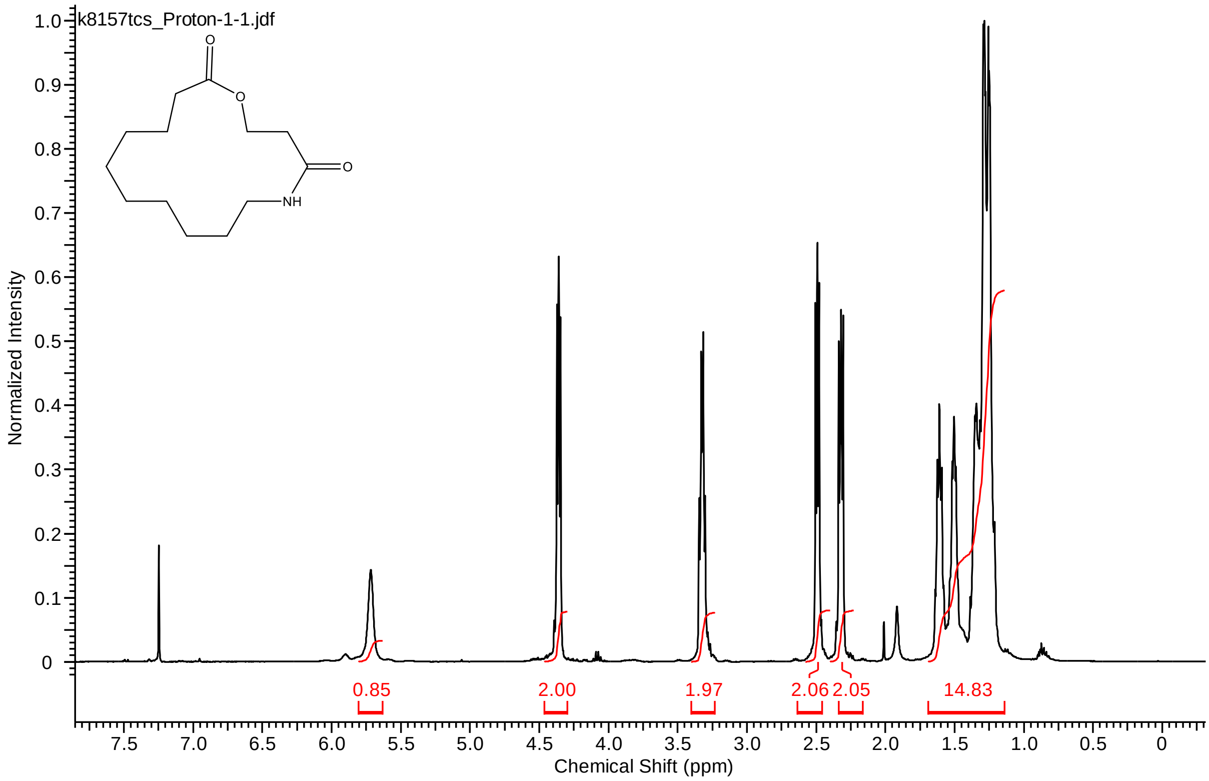

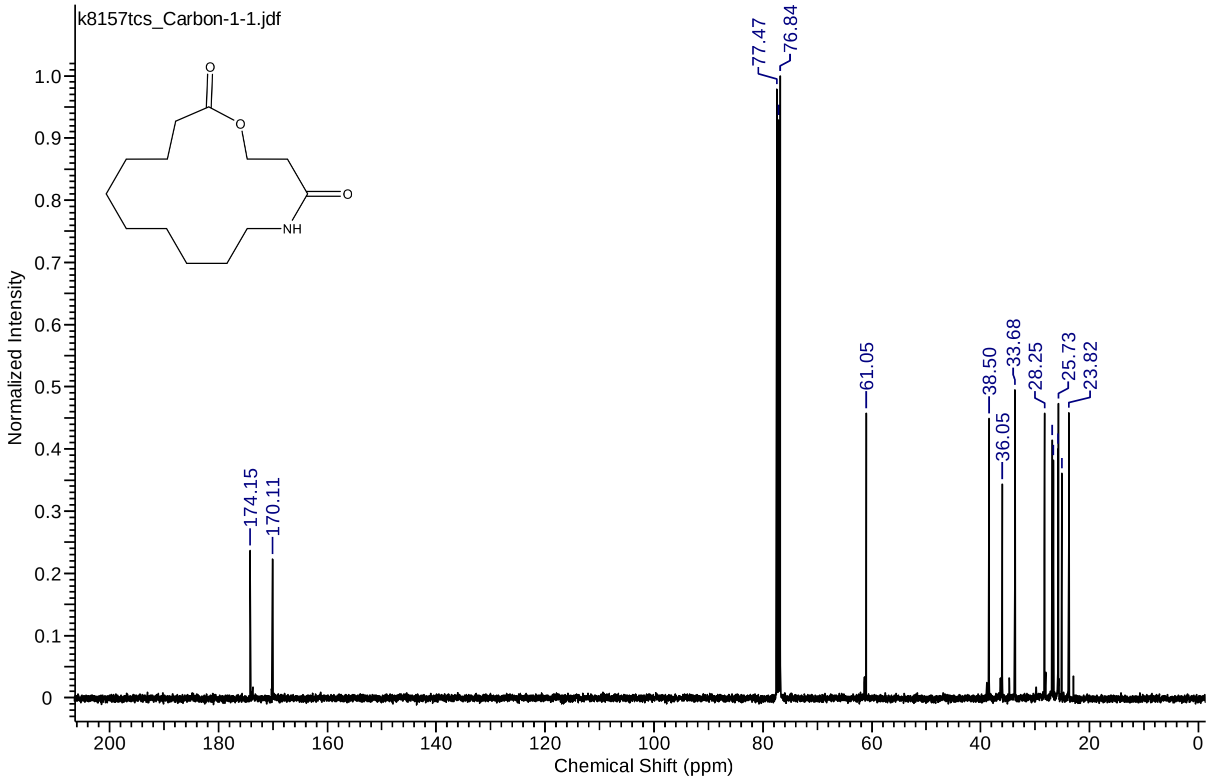

Compound 13j

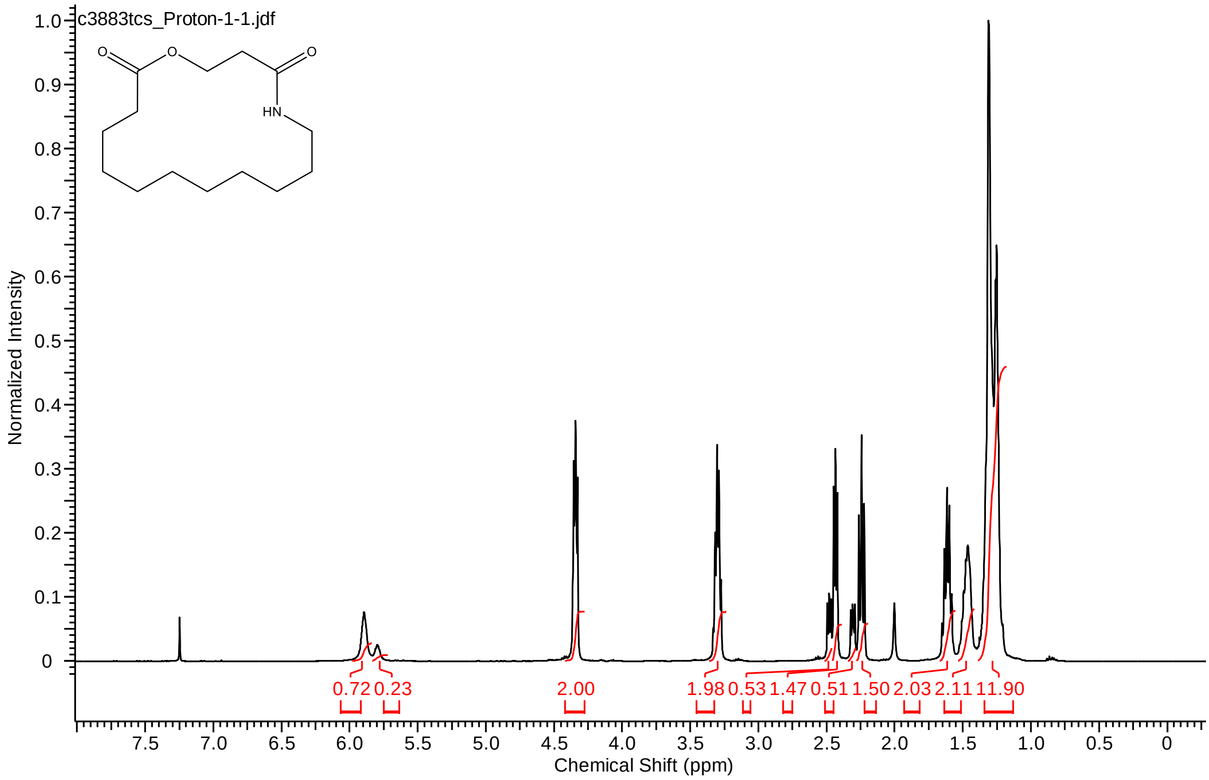

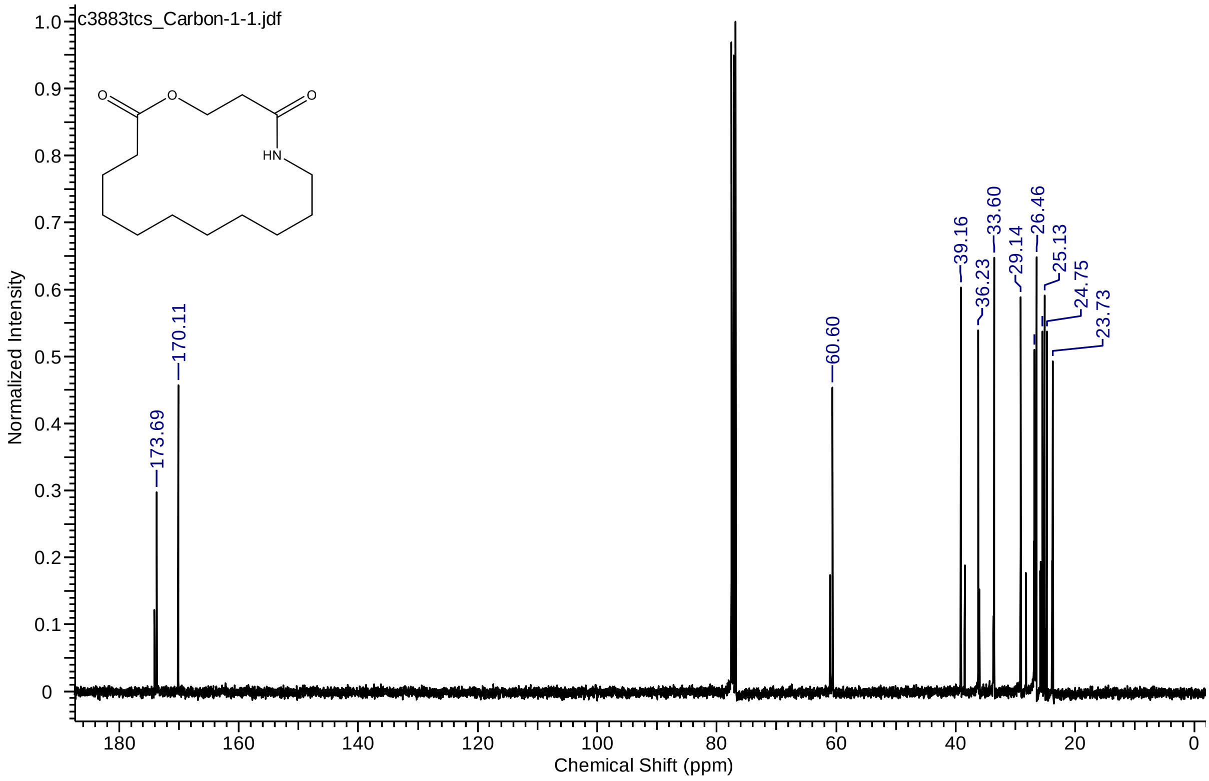

Compound 16a

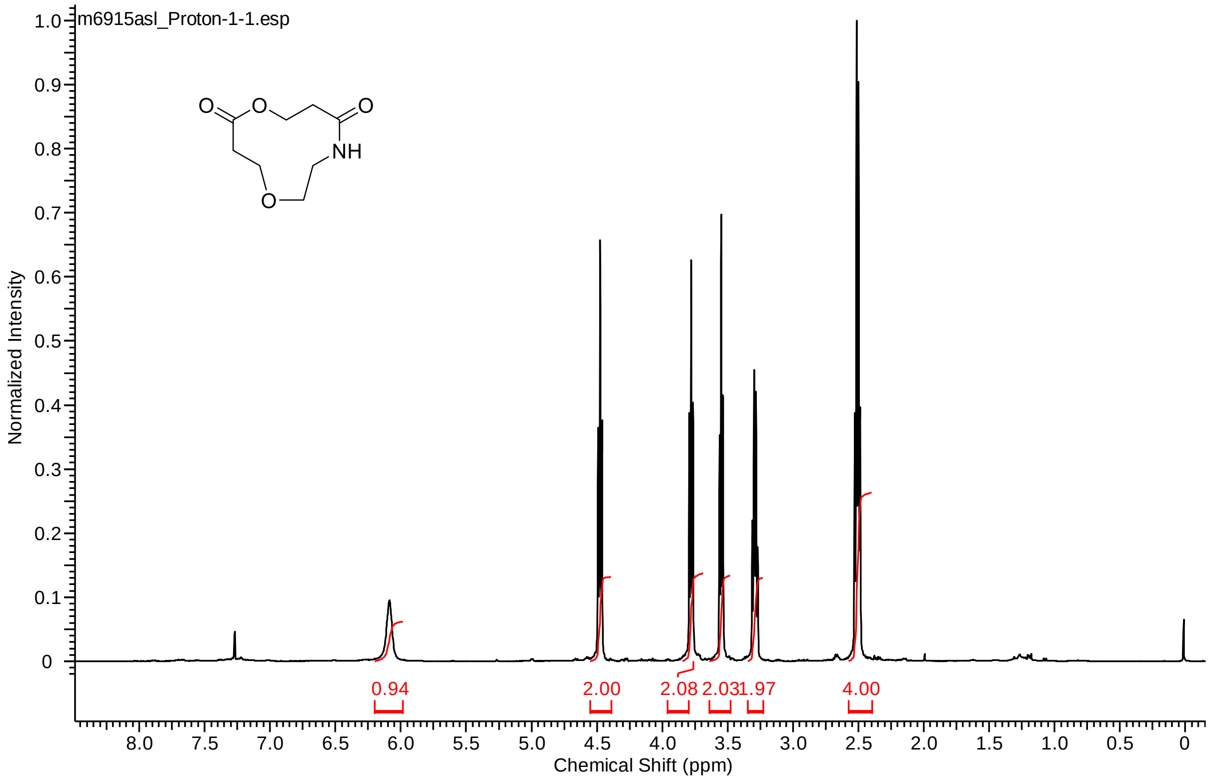

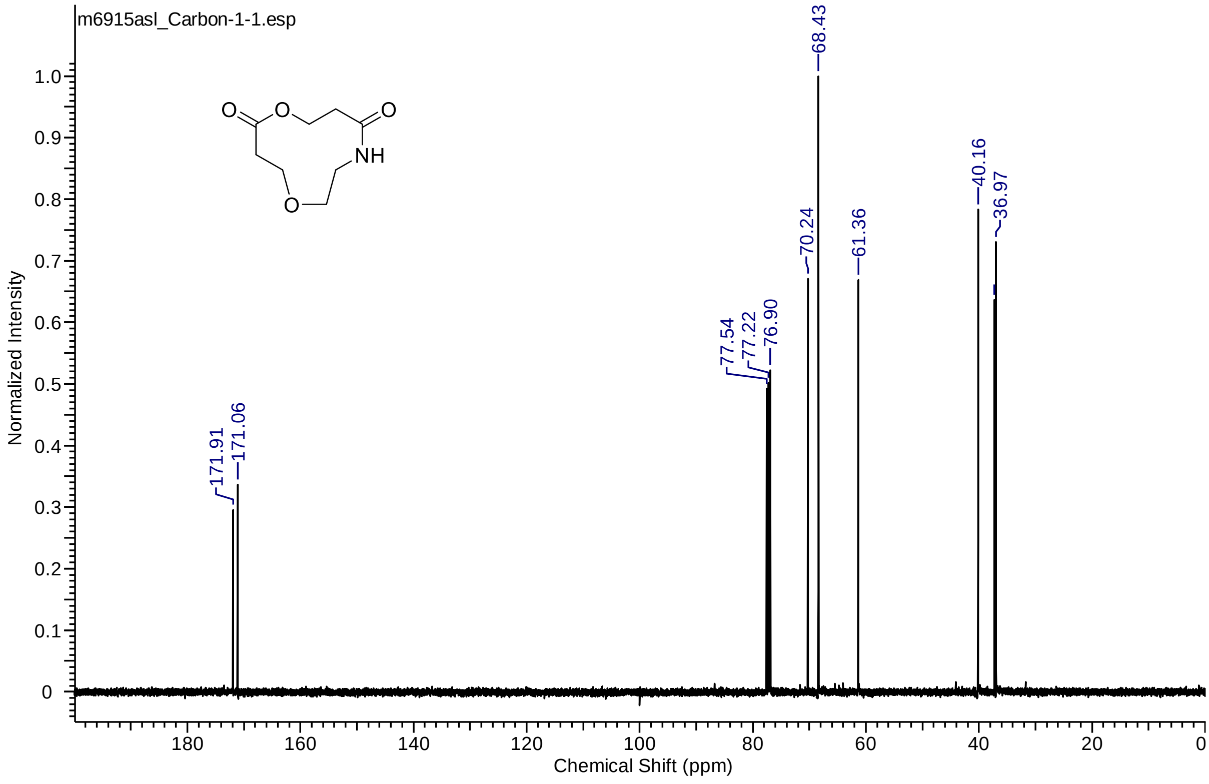

Compound 16b

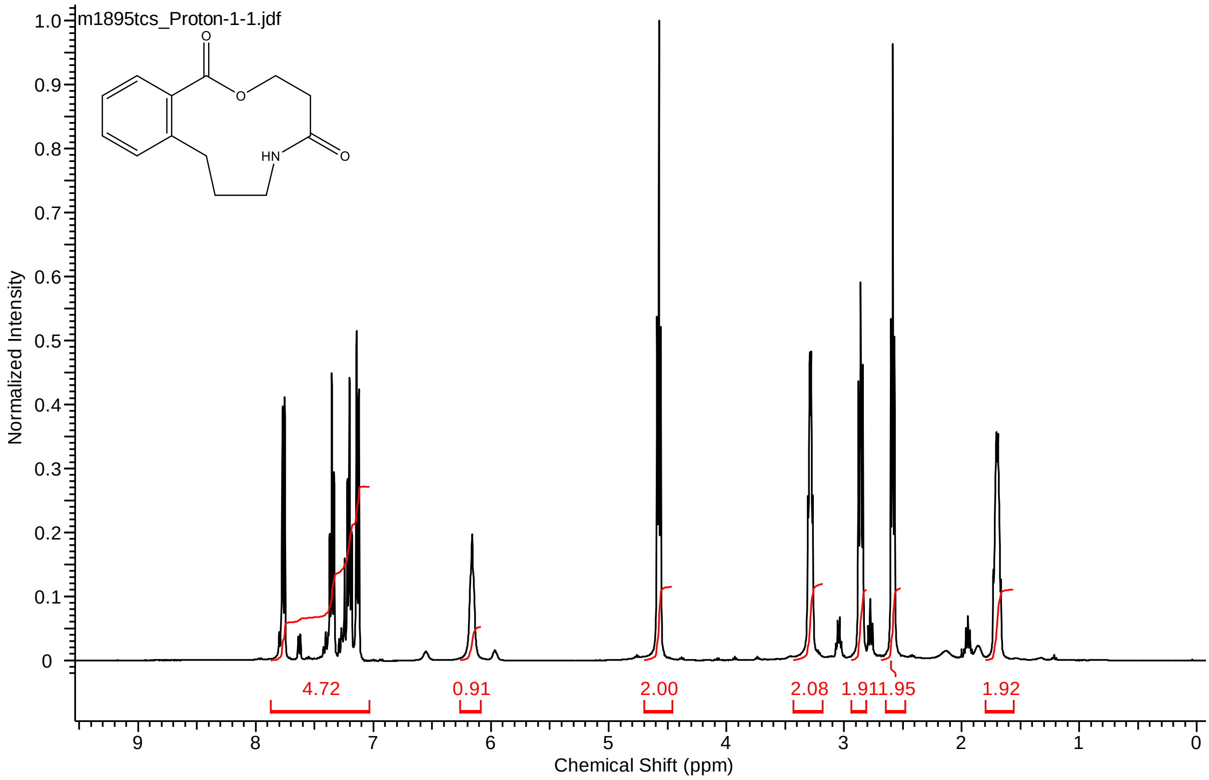

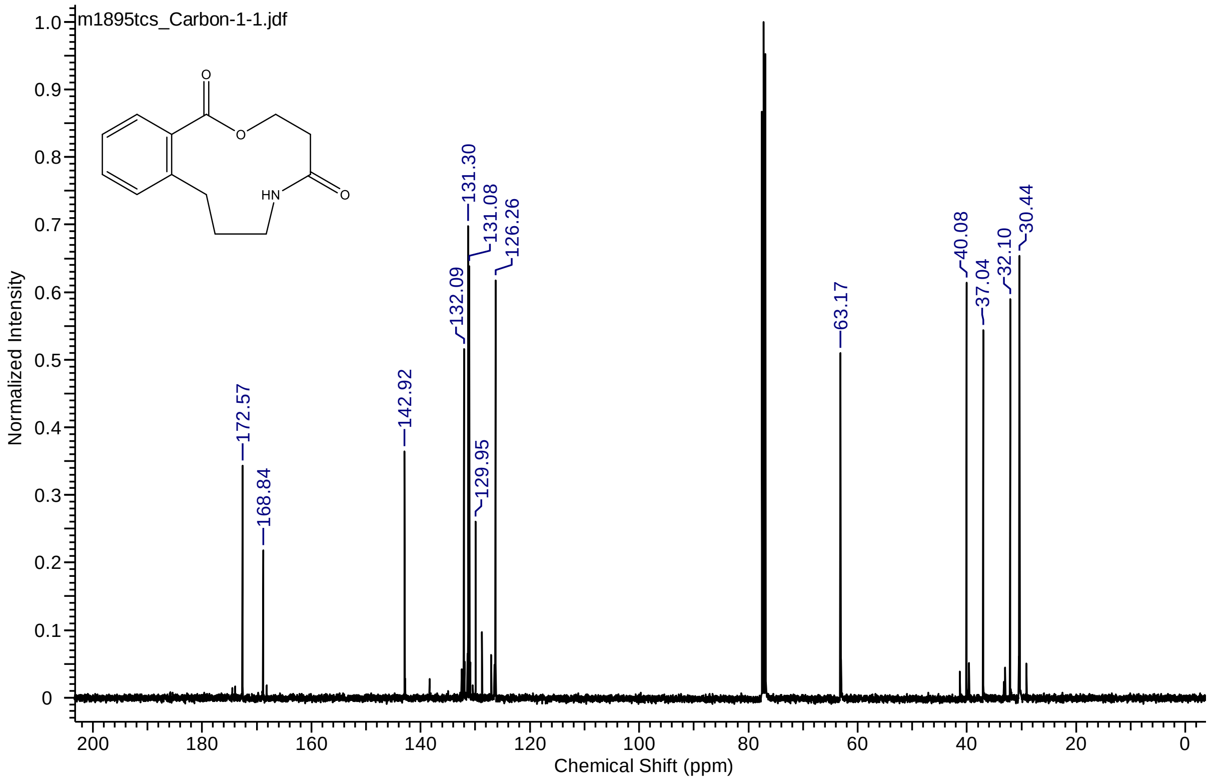

Compound 16c

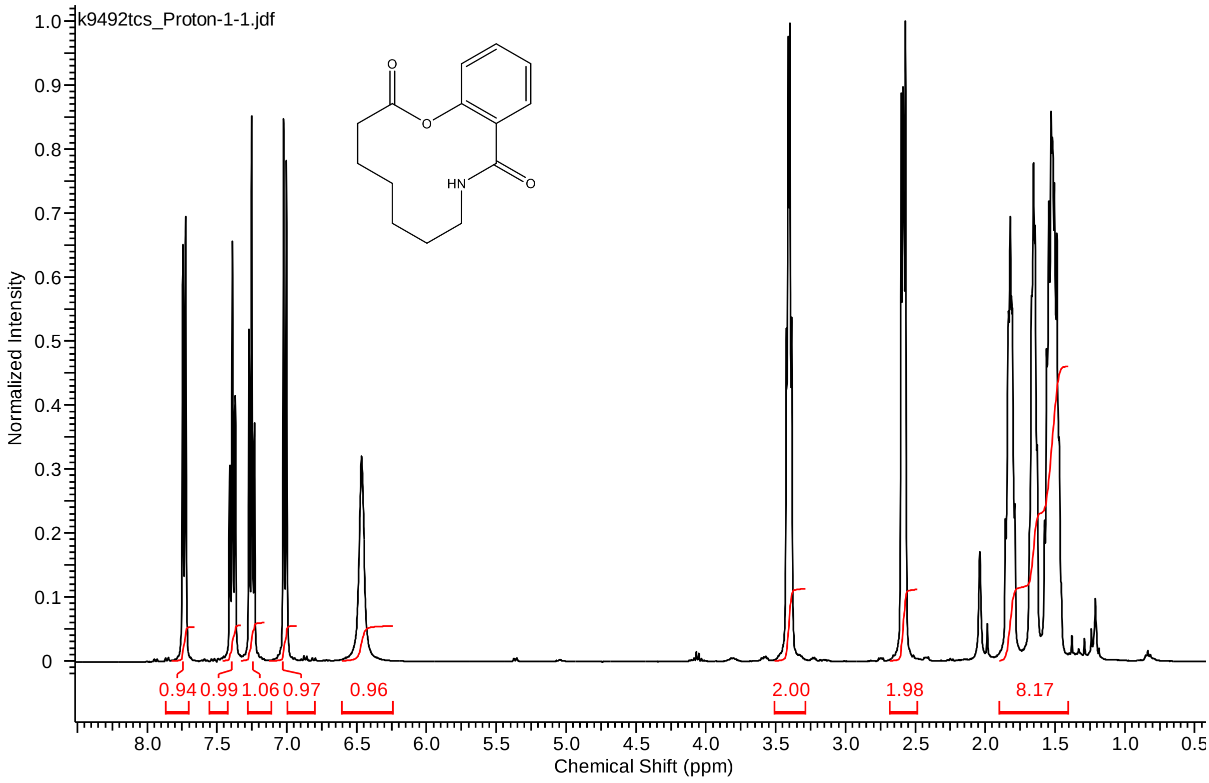

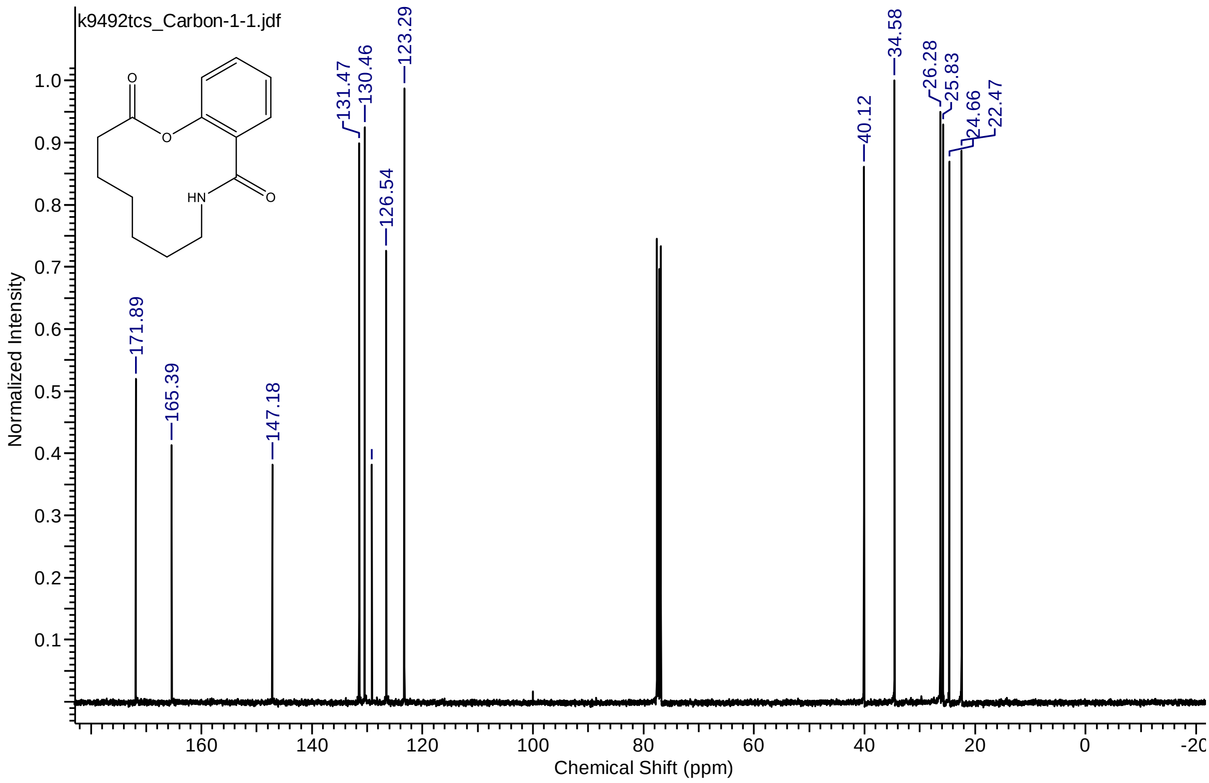

Compound 16d

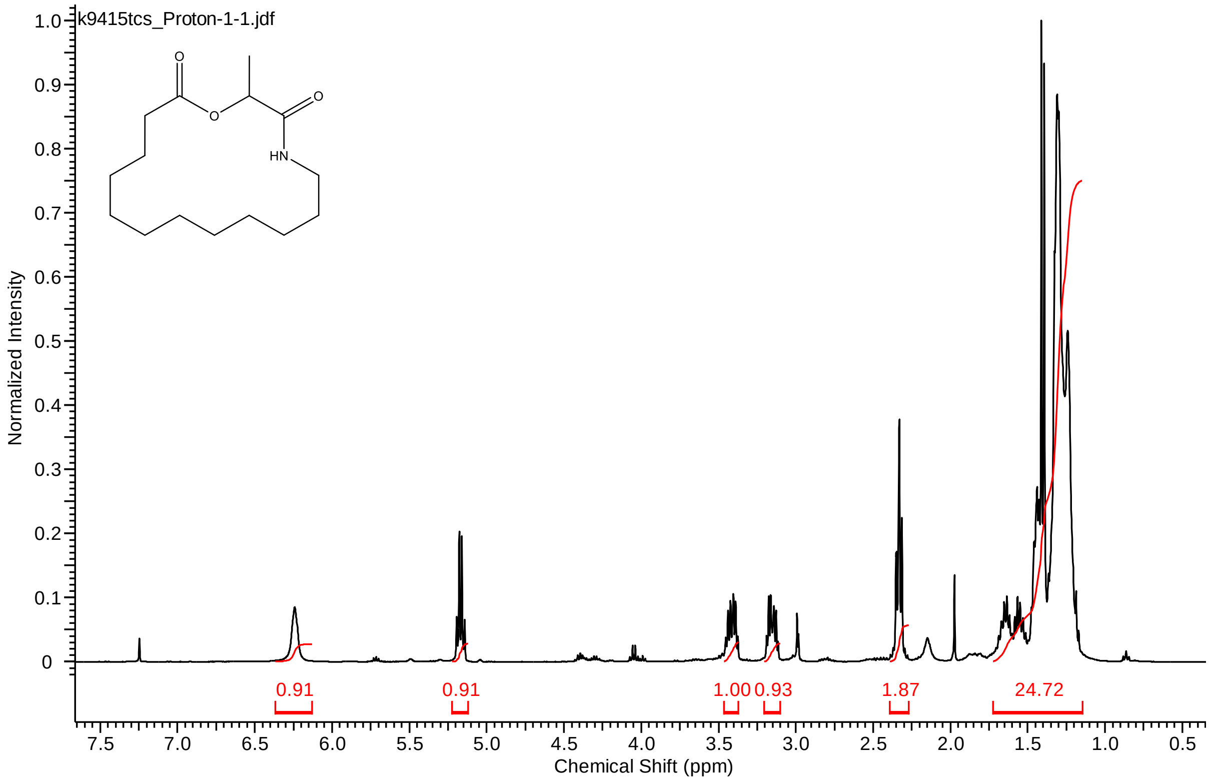

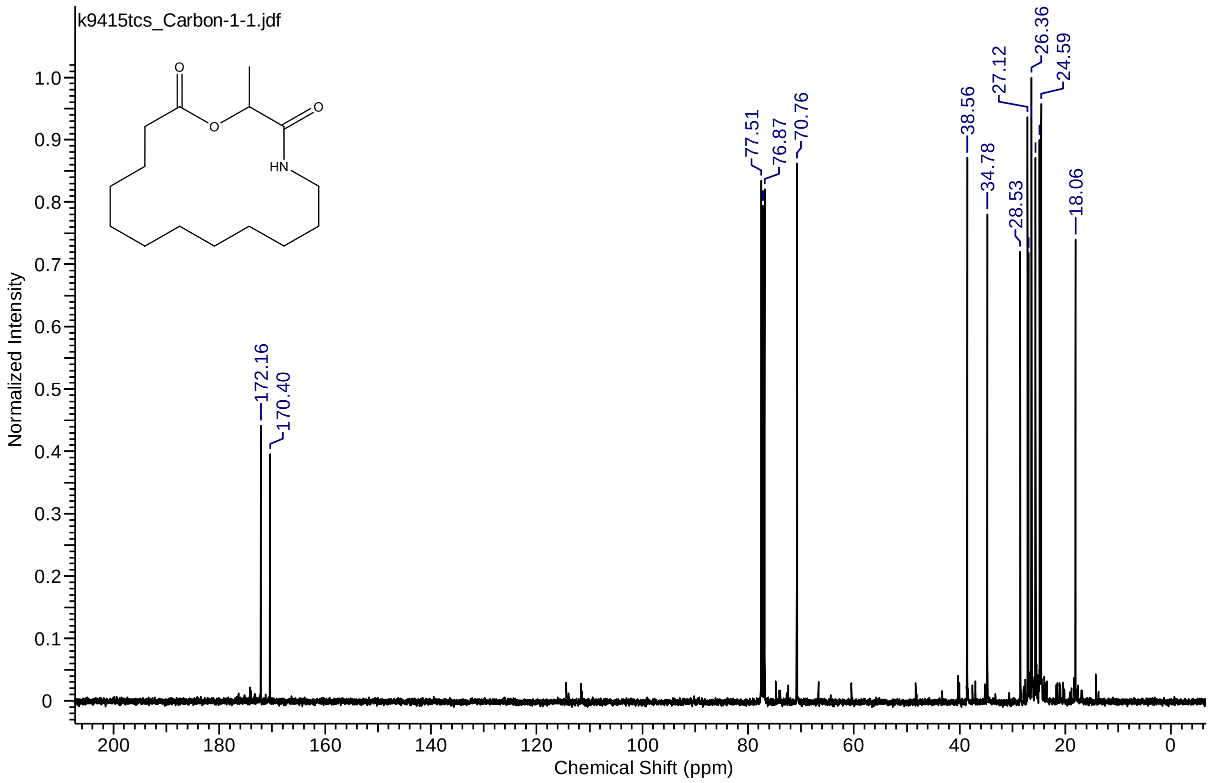

Compound 16e

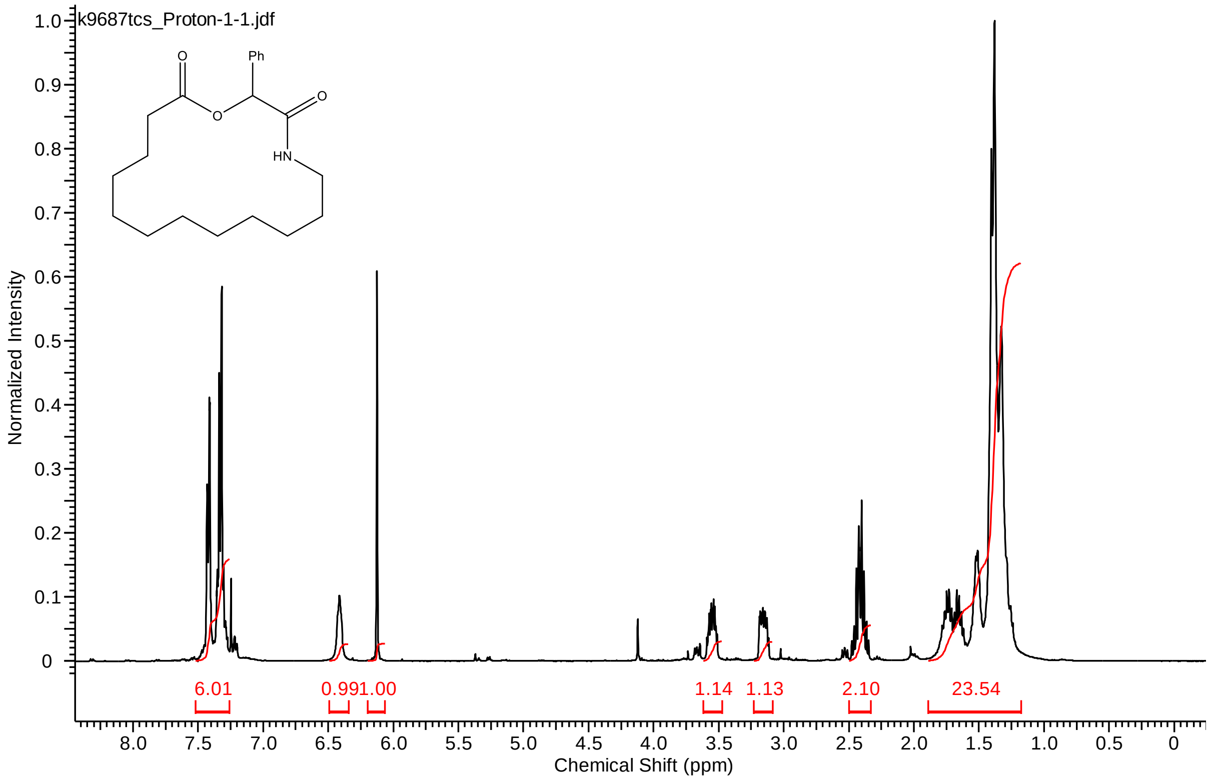

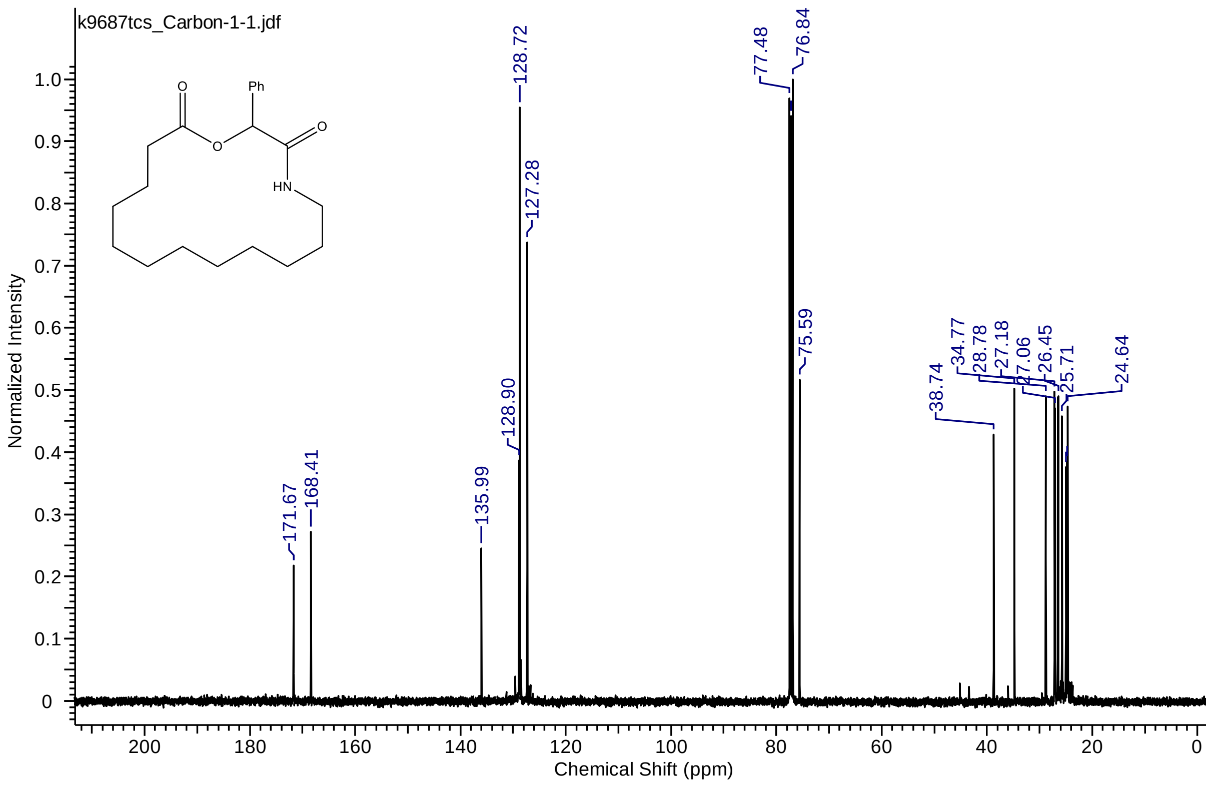

Compound 16f

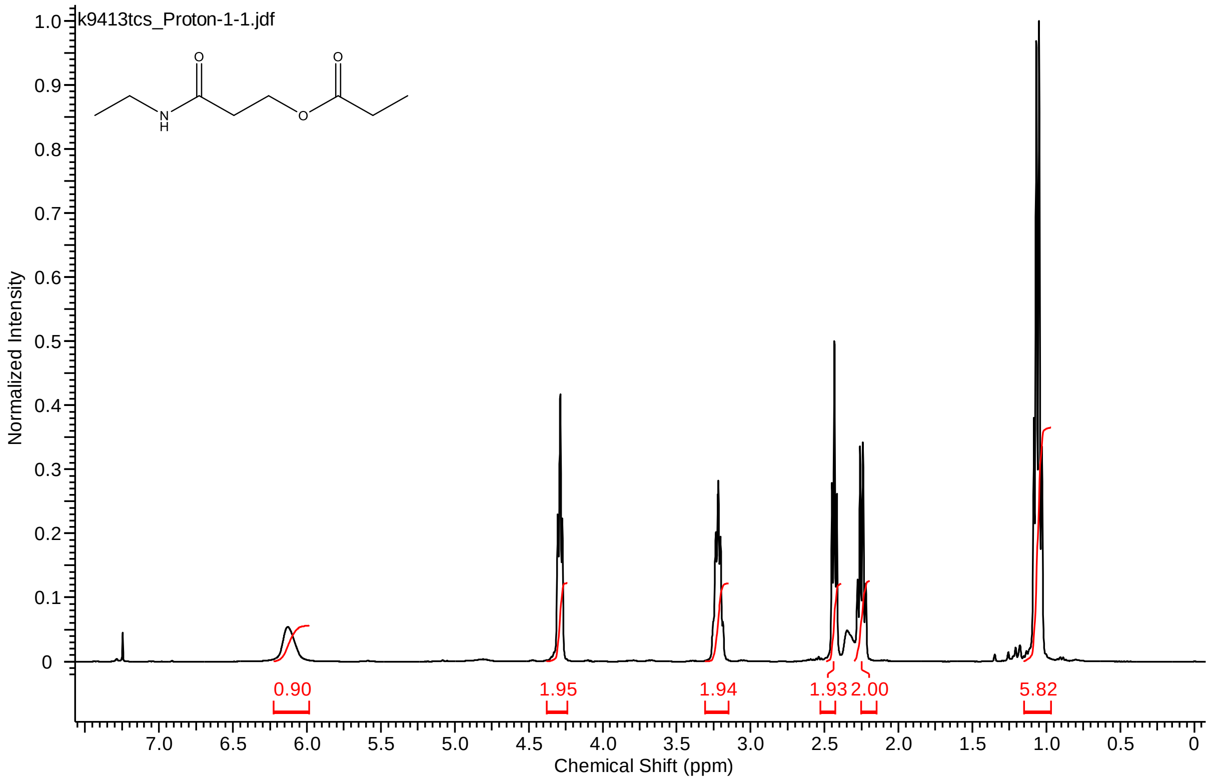

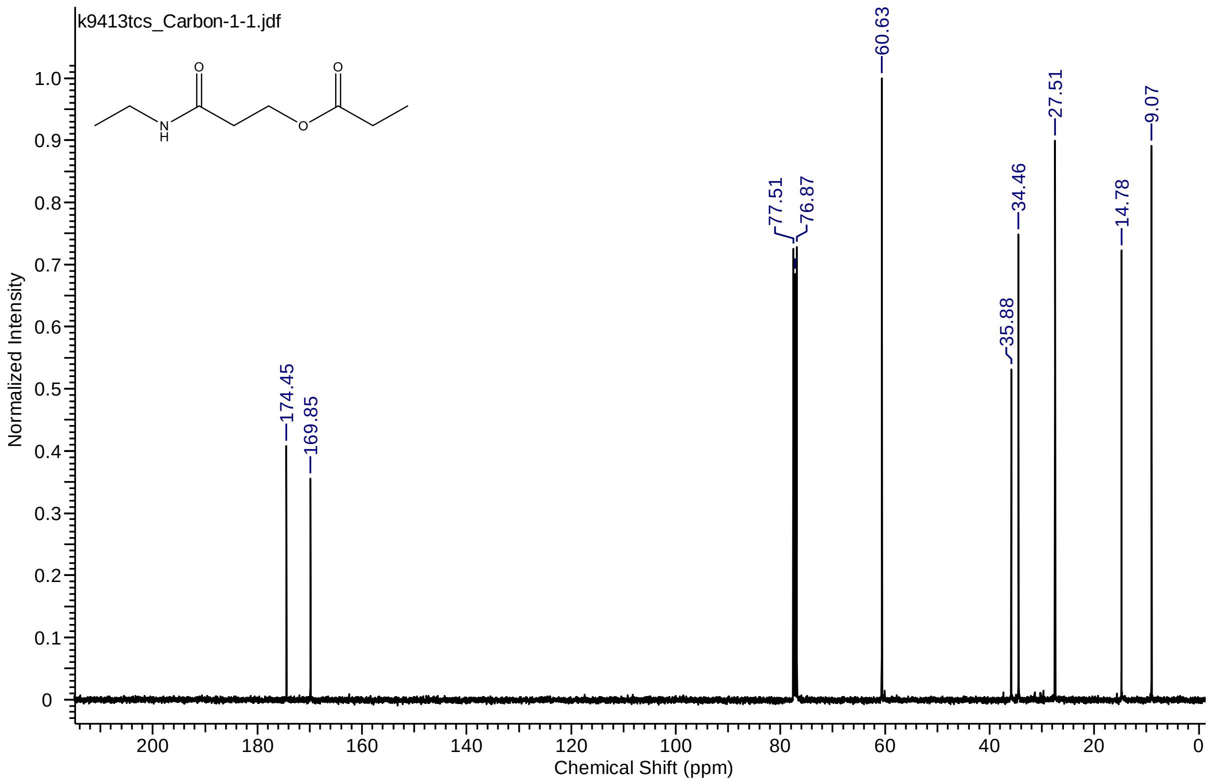

Compound 17a

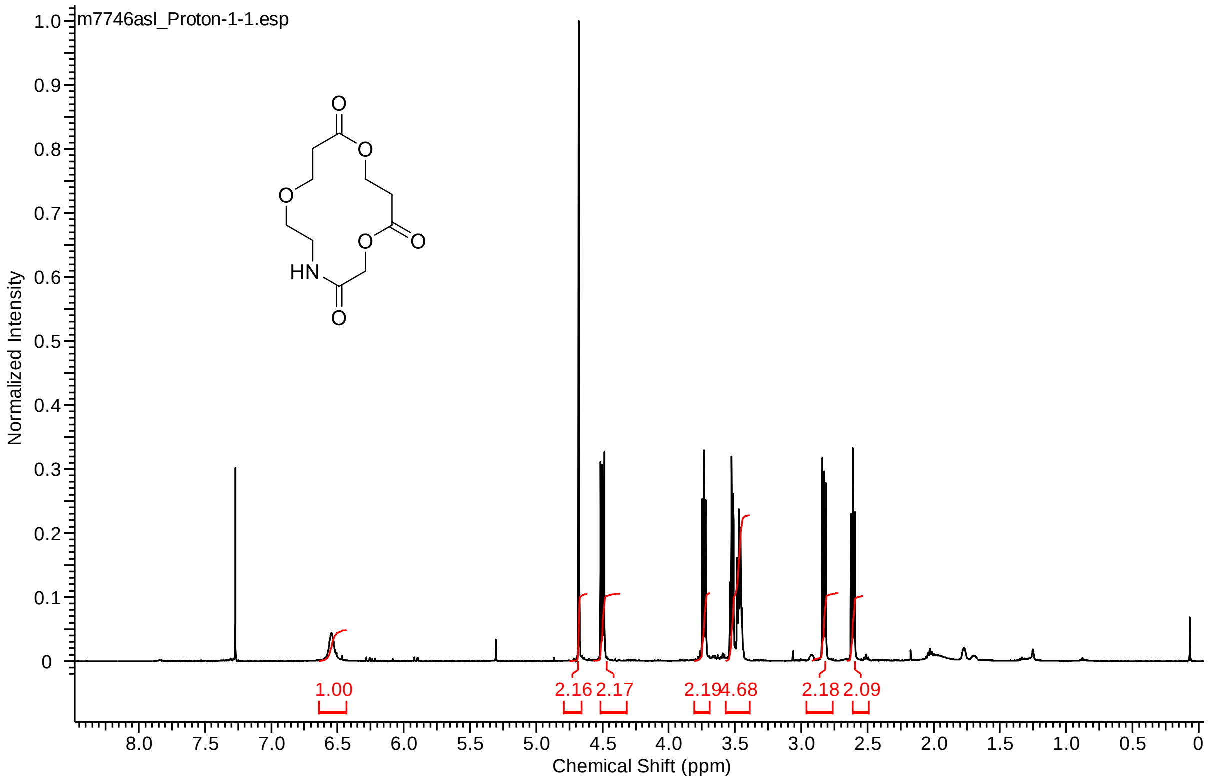

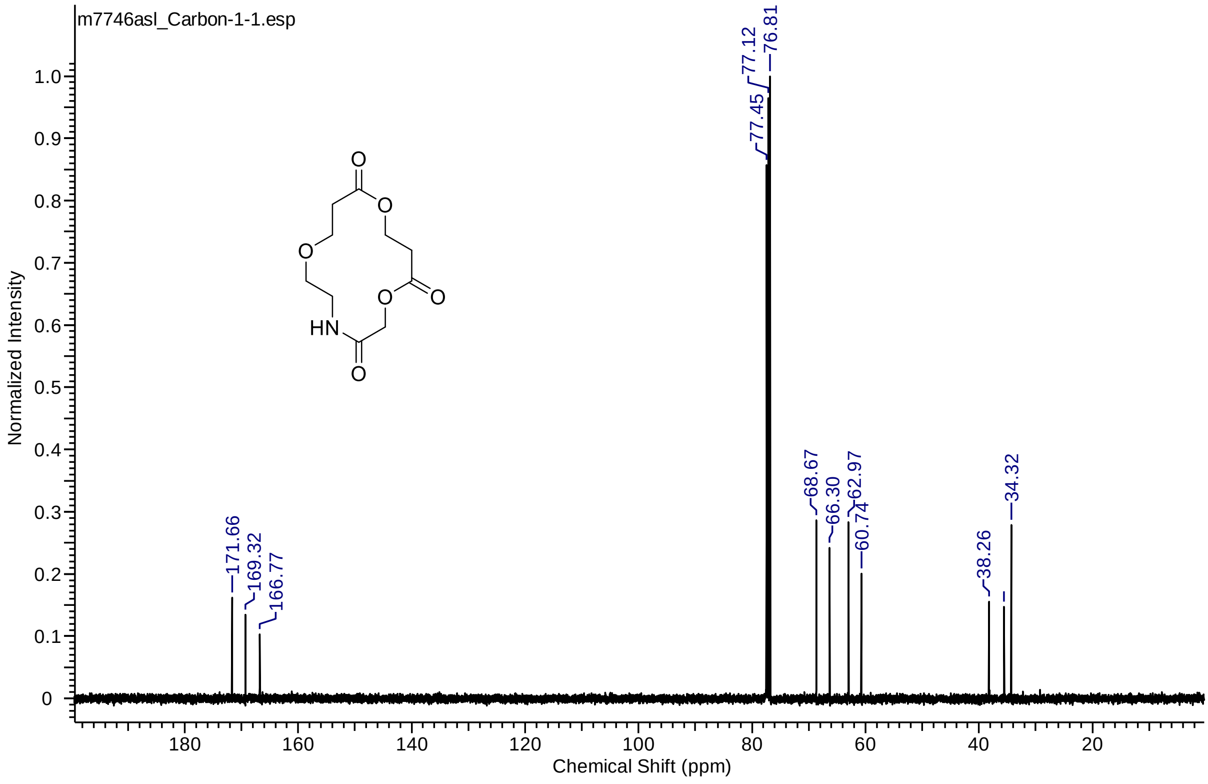

Compound 17b

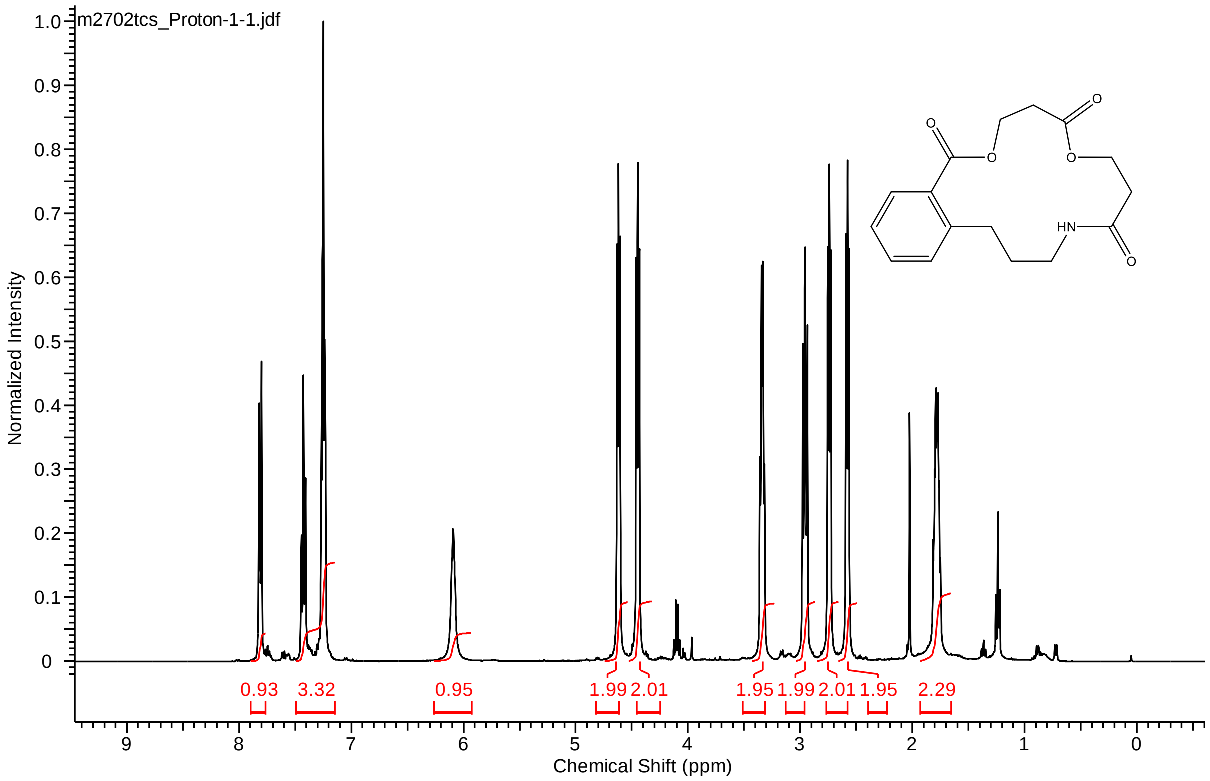

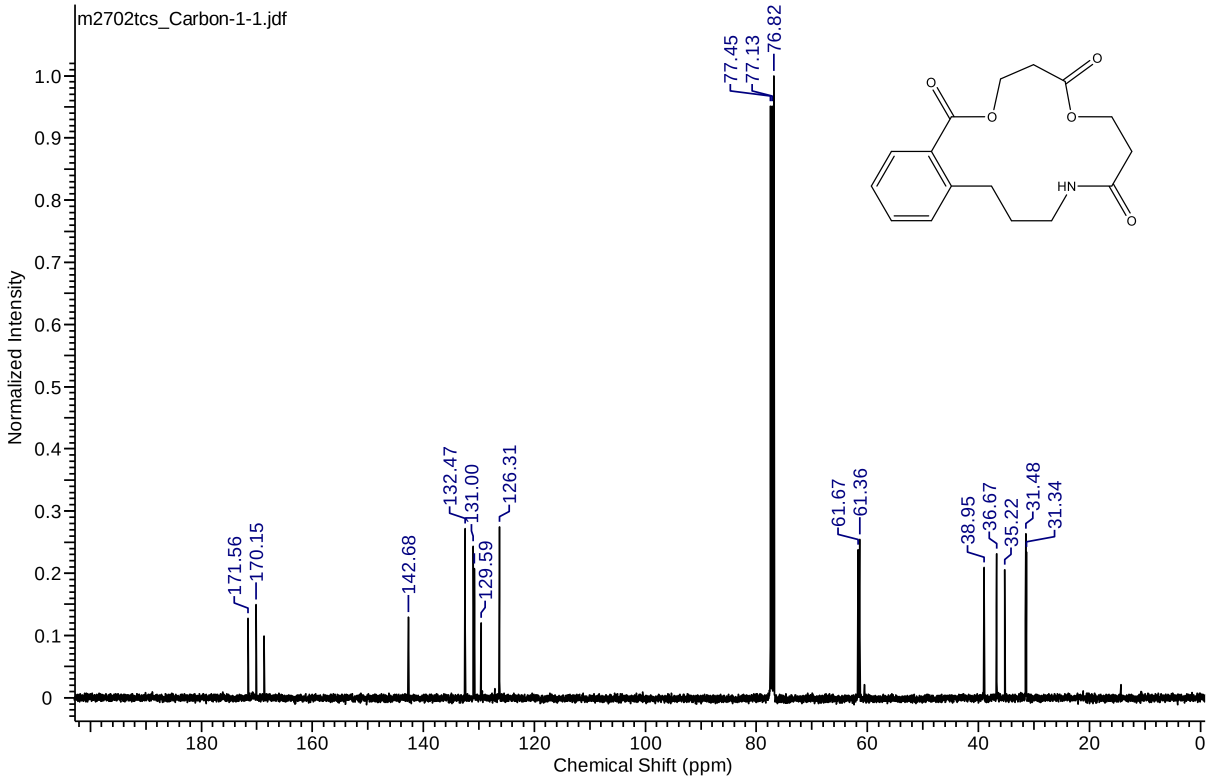

Compound 17c

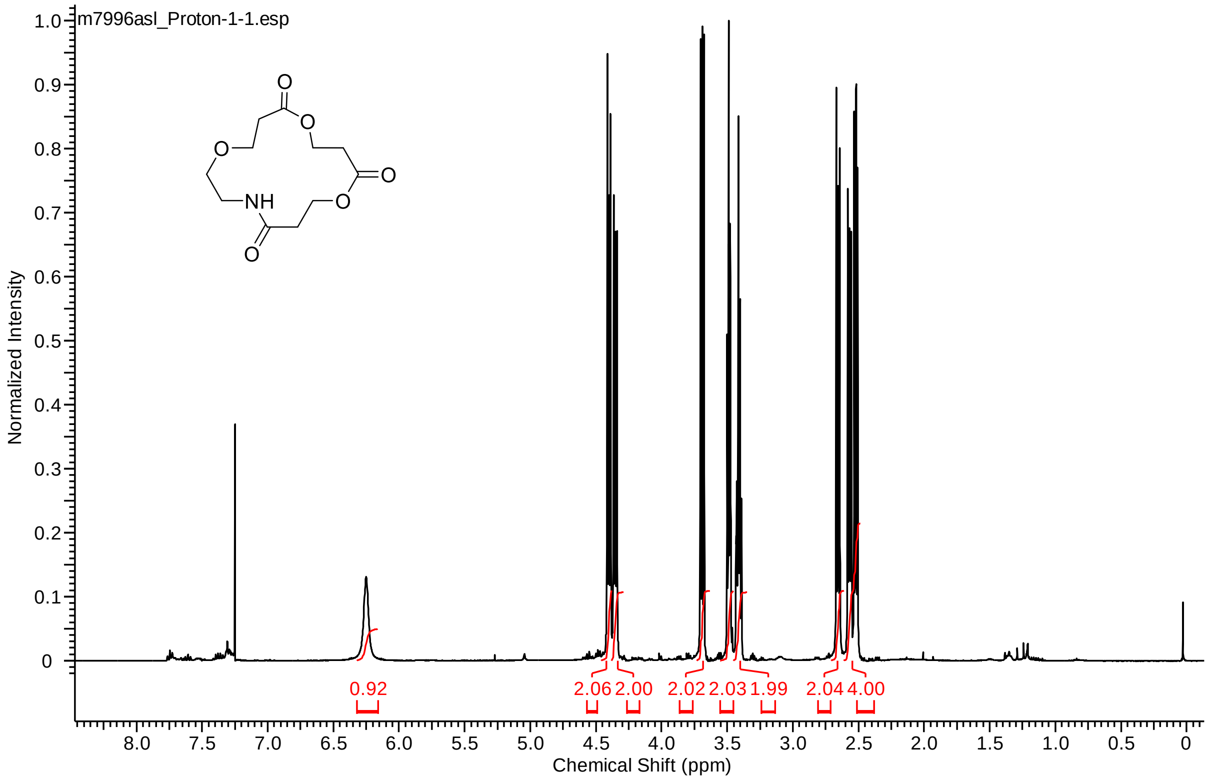

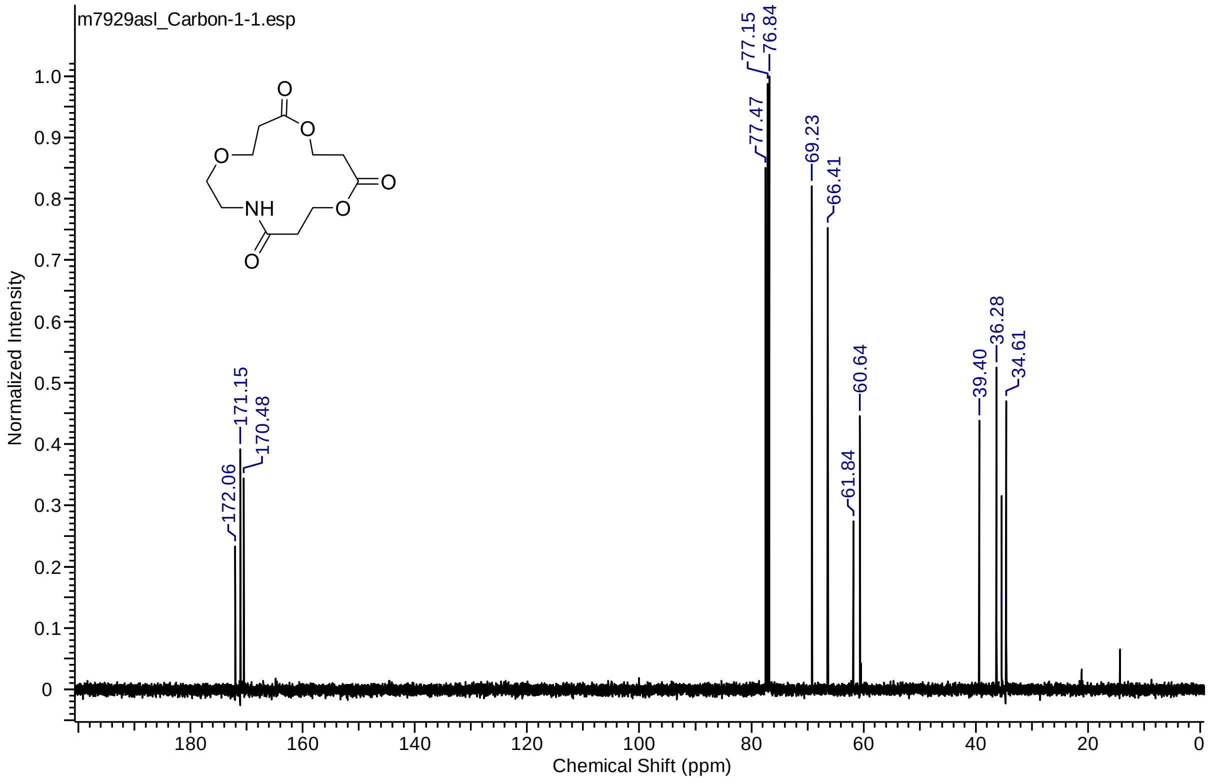

Compound 17d

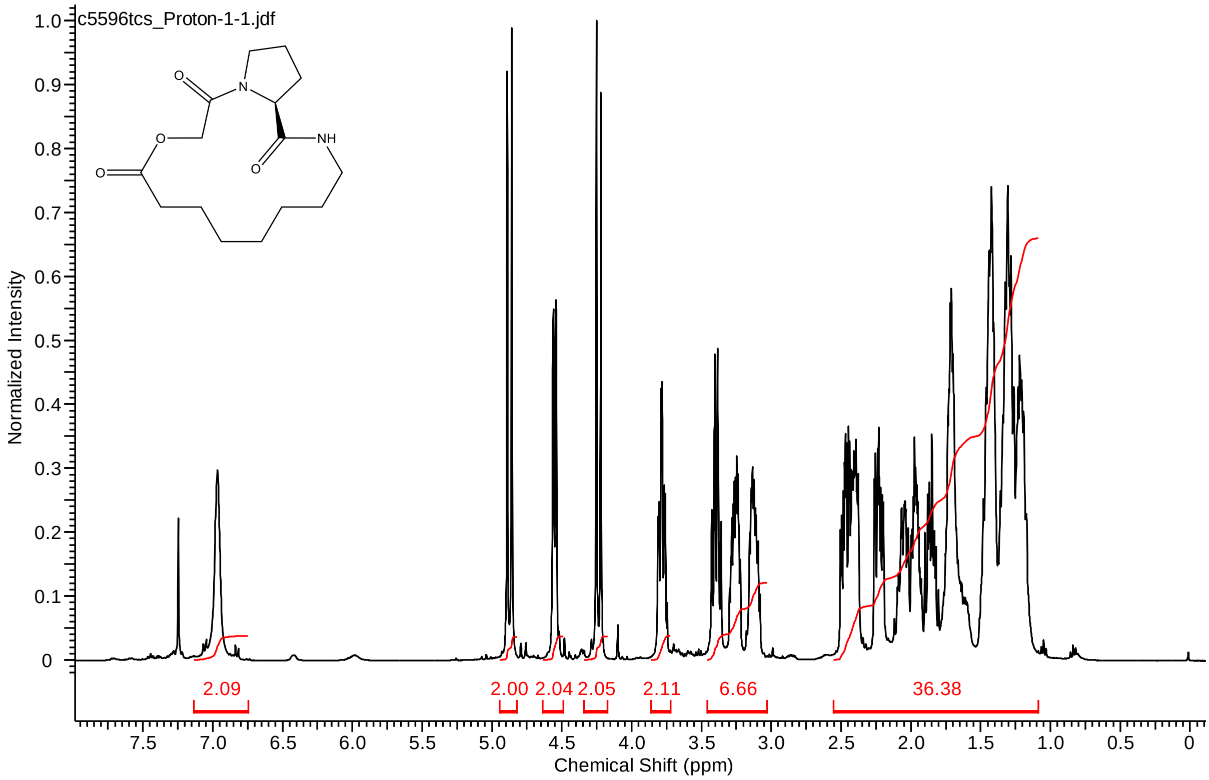

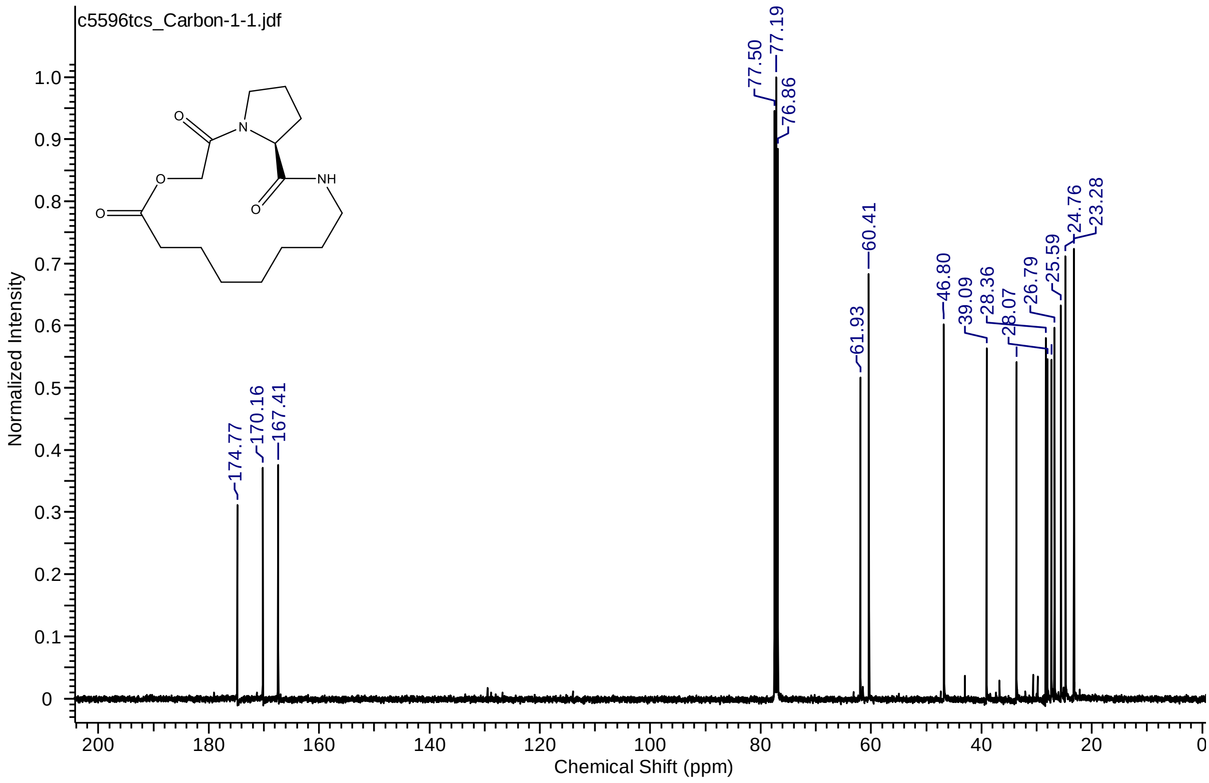

Compound 17e

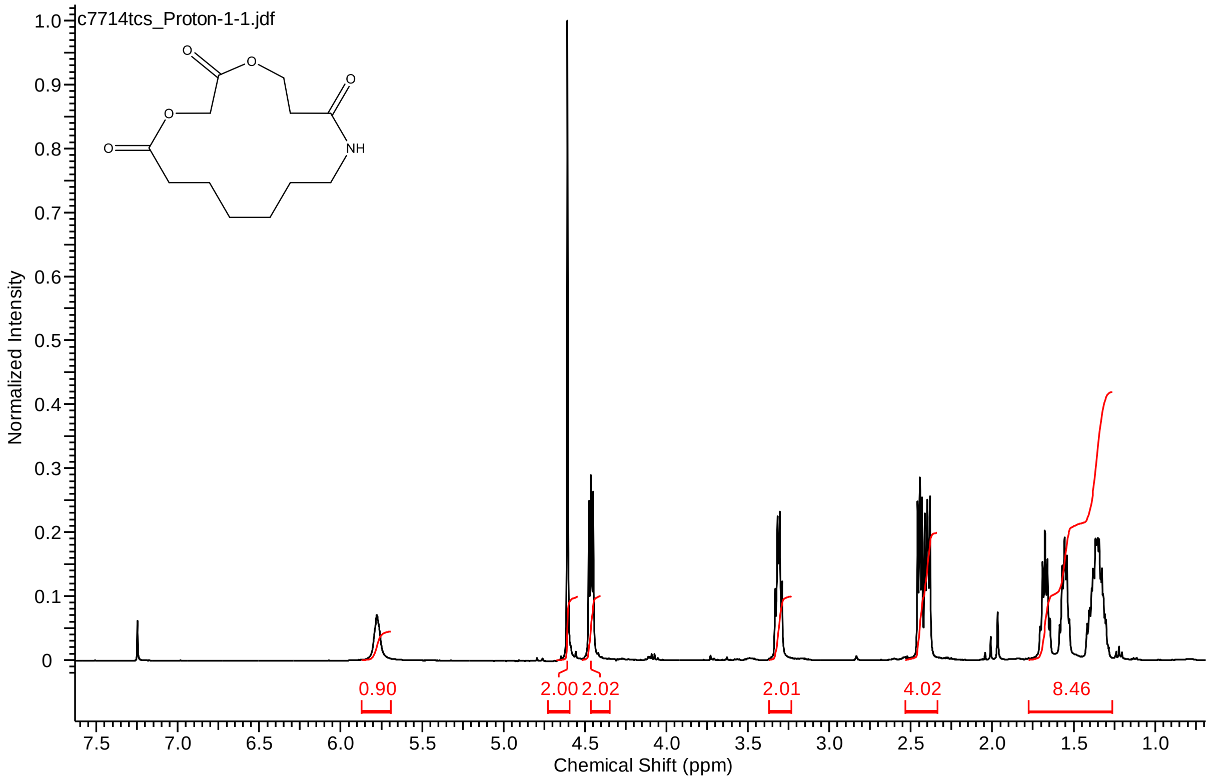

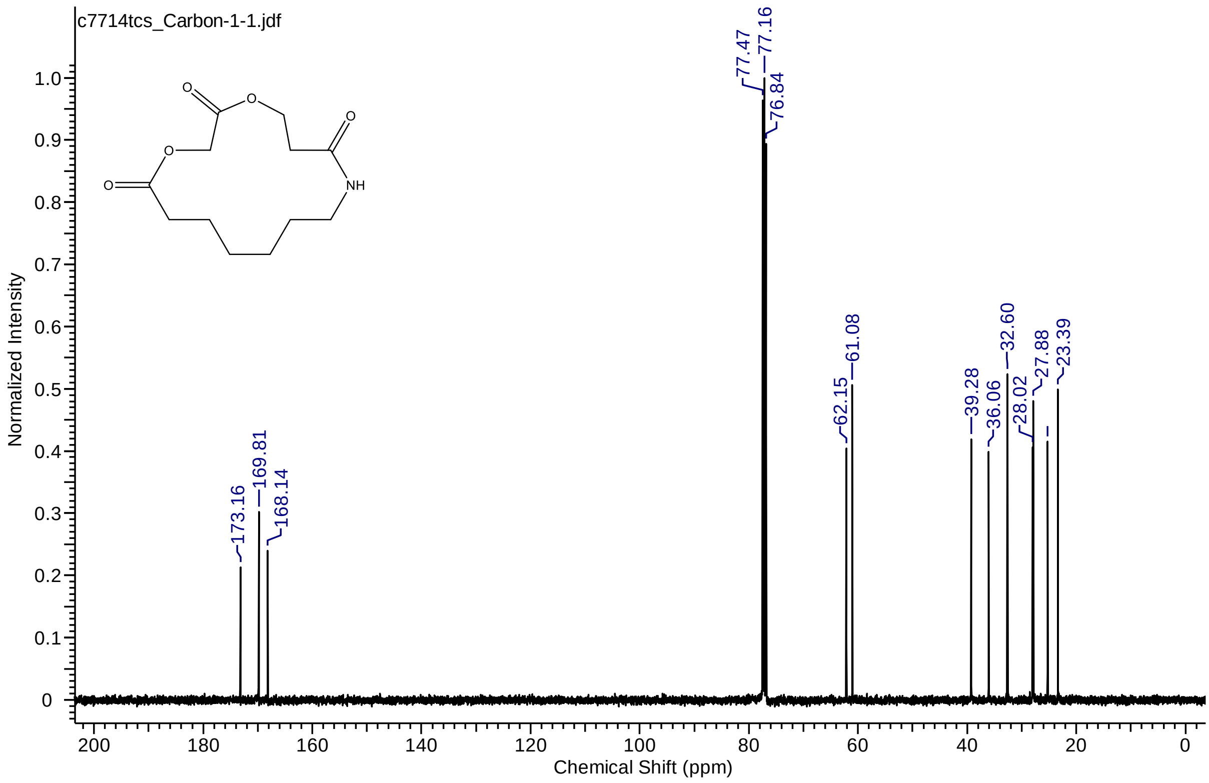

Compound 17f

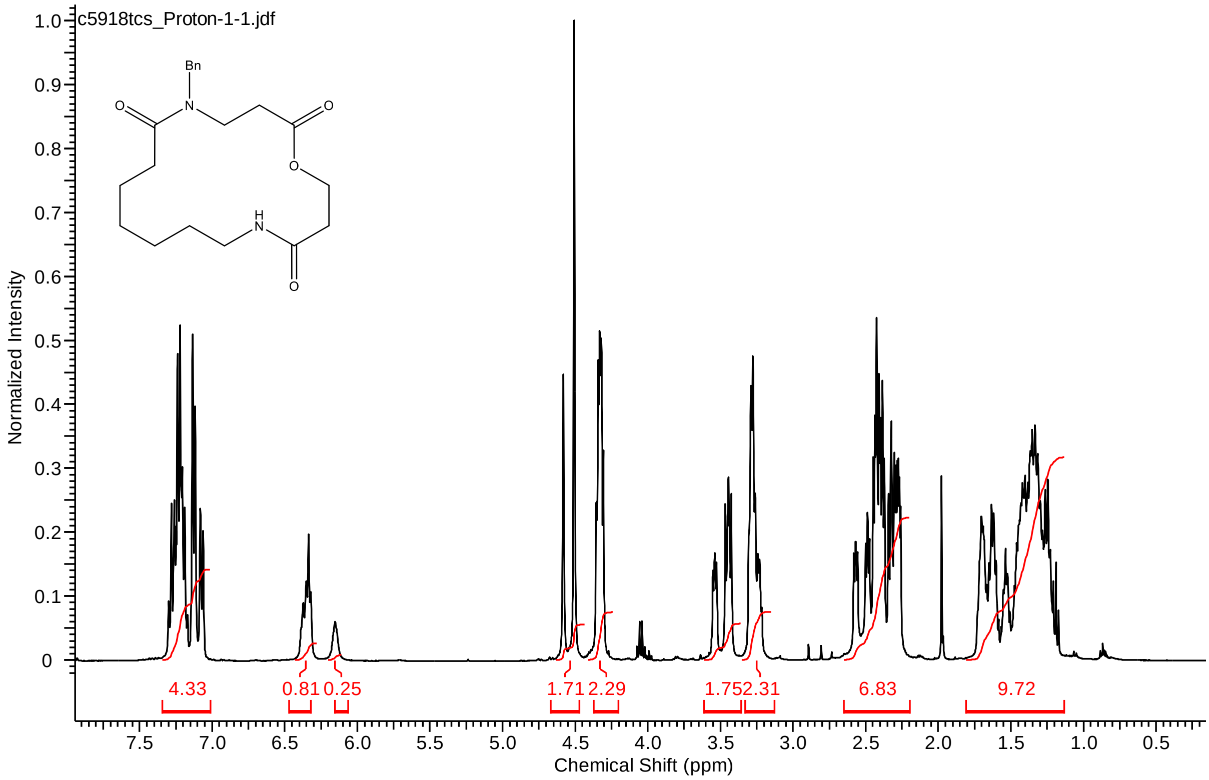

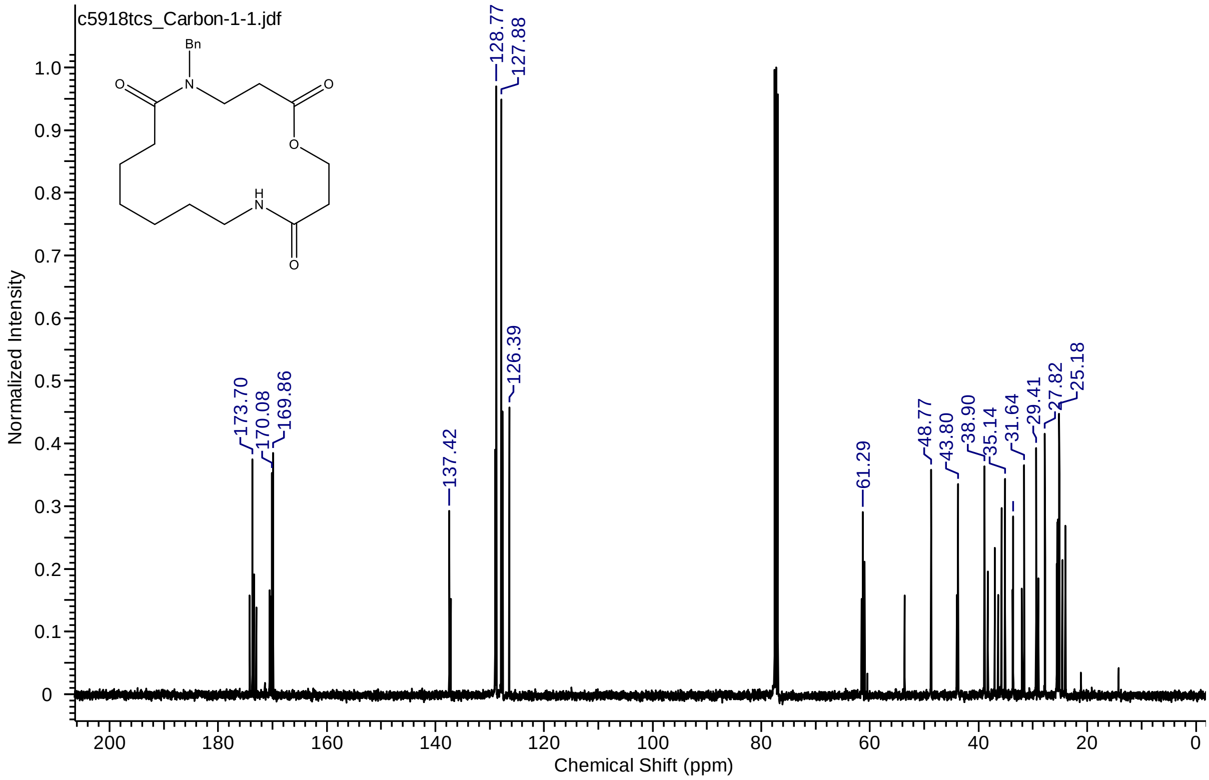

Compound 17g

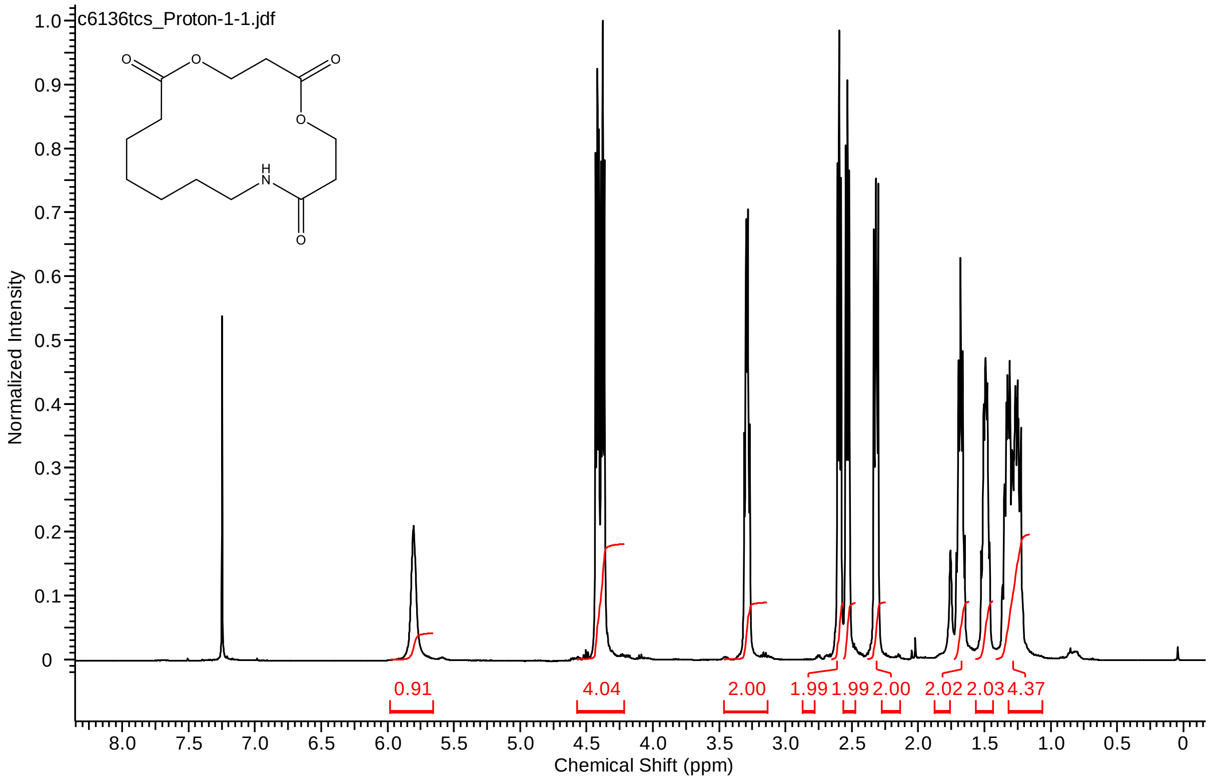

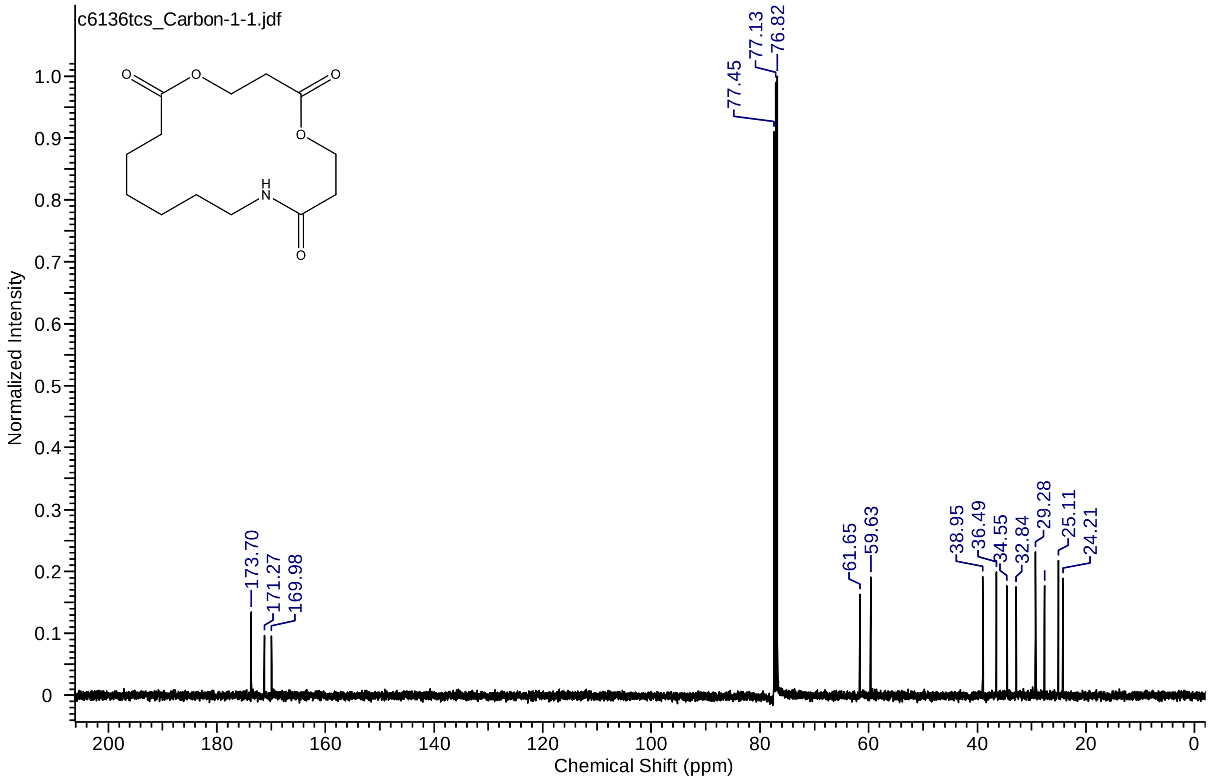

Compound 17h

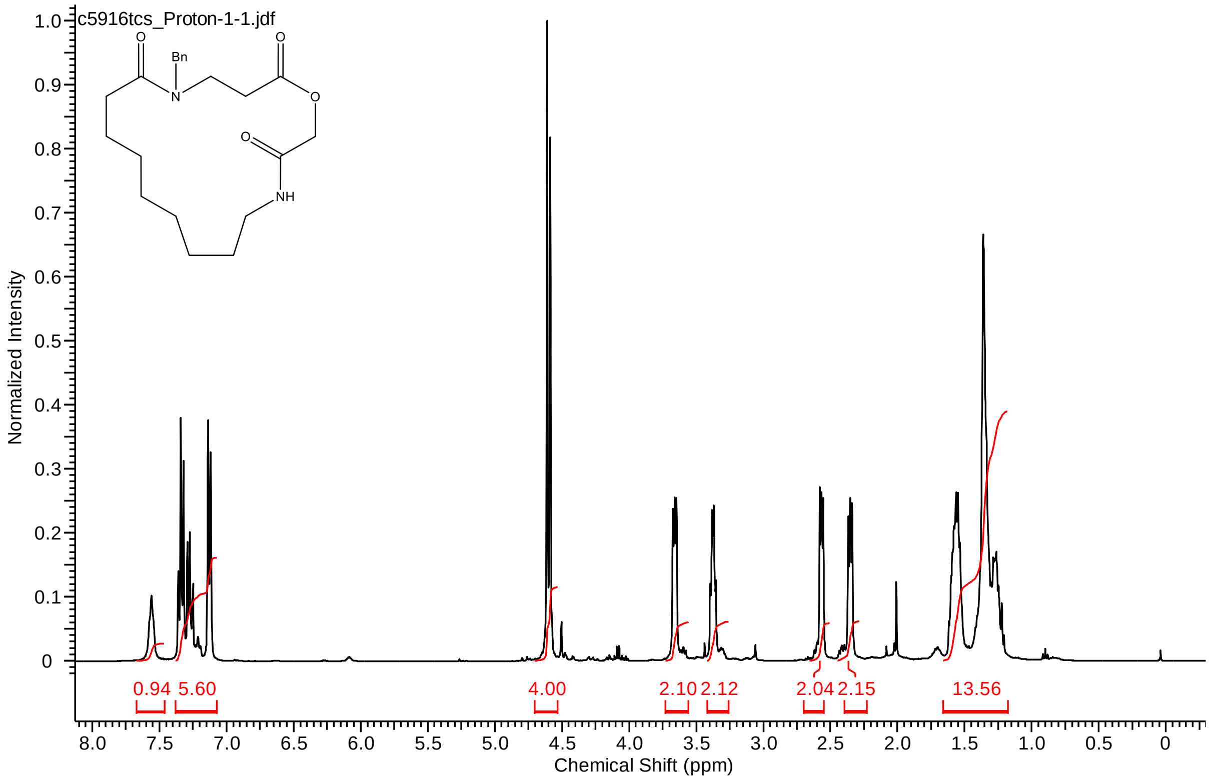

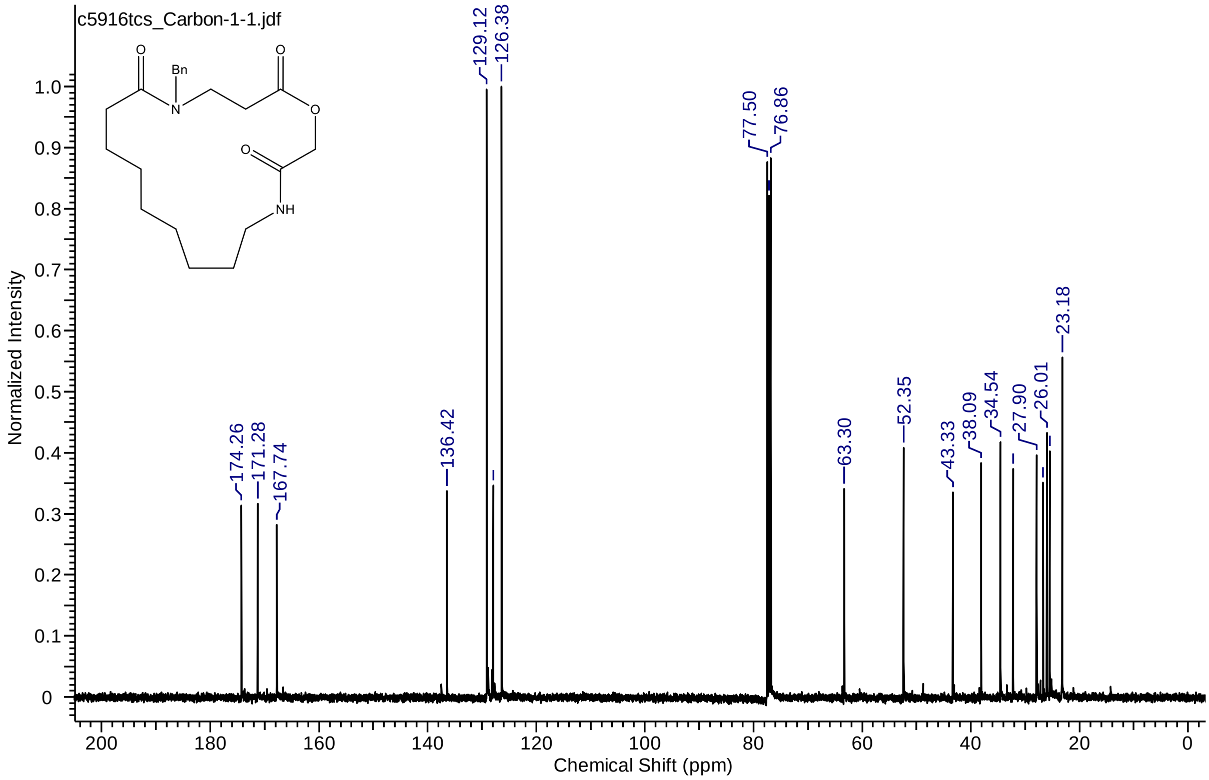

Compound 17i

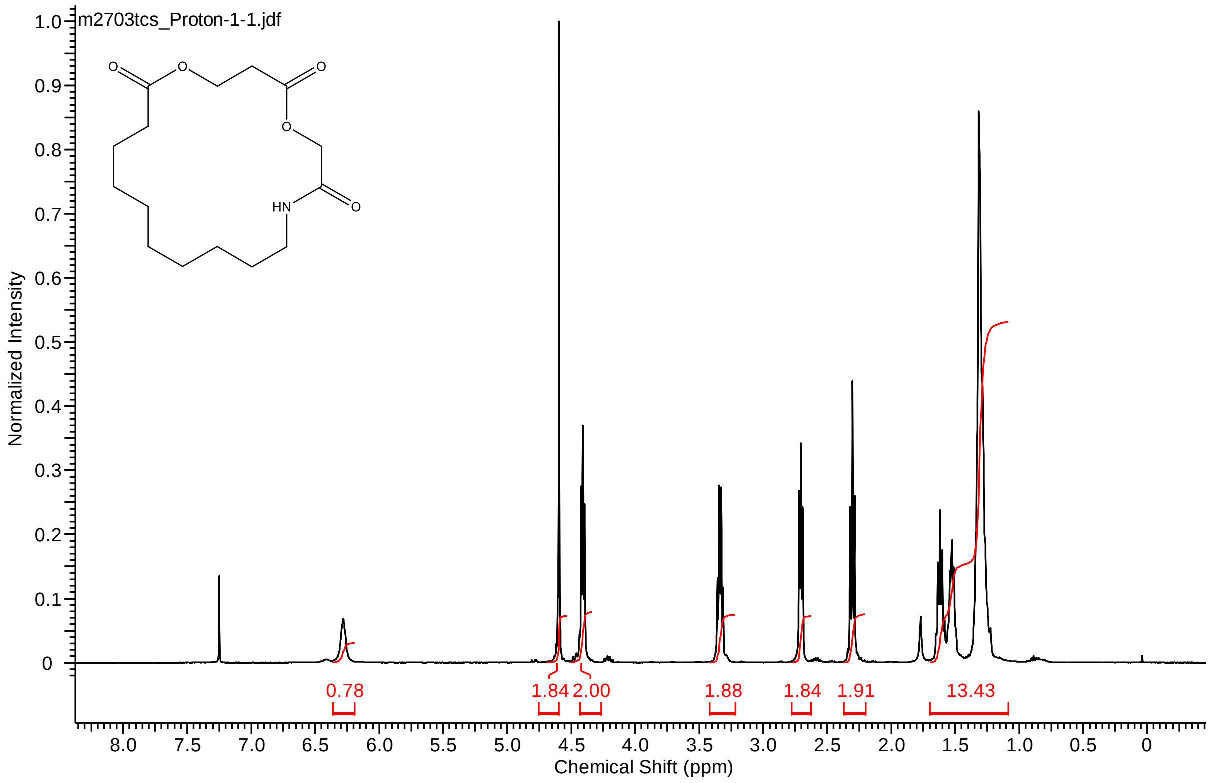

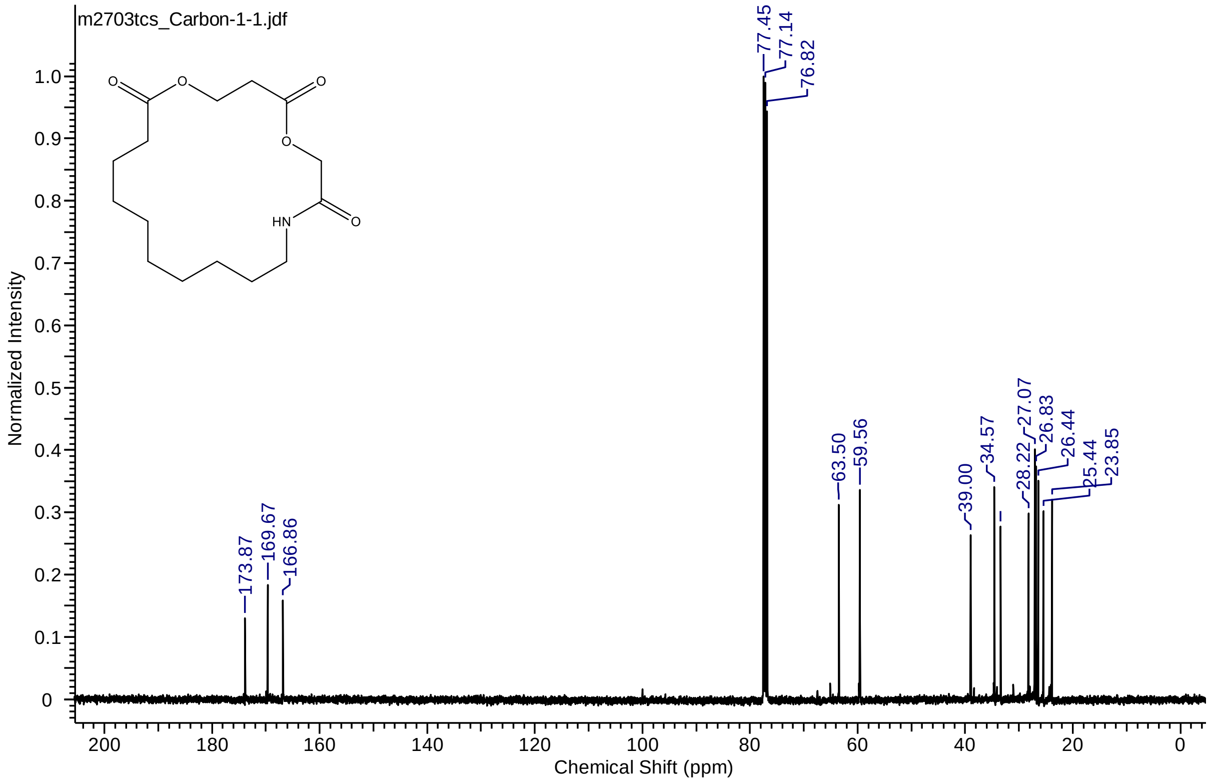

Compound 17j

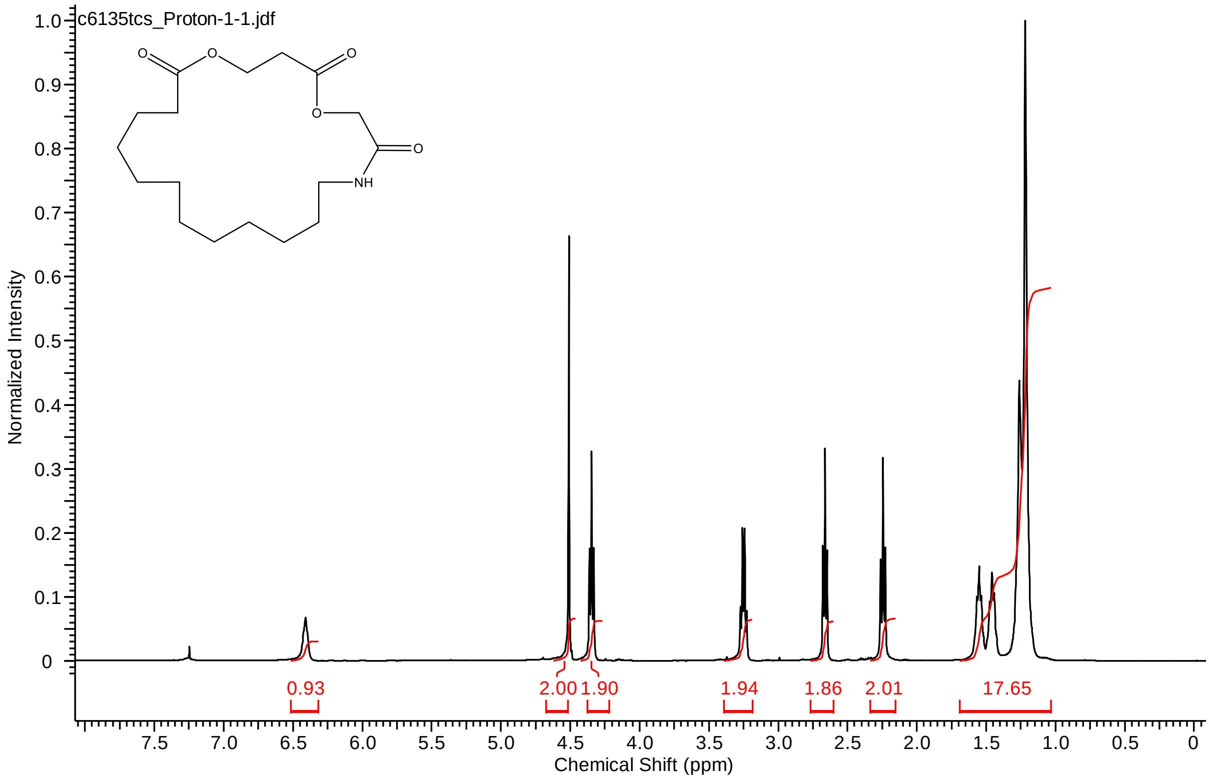

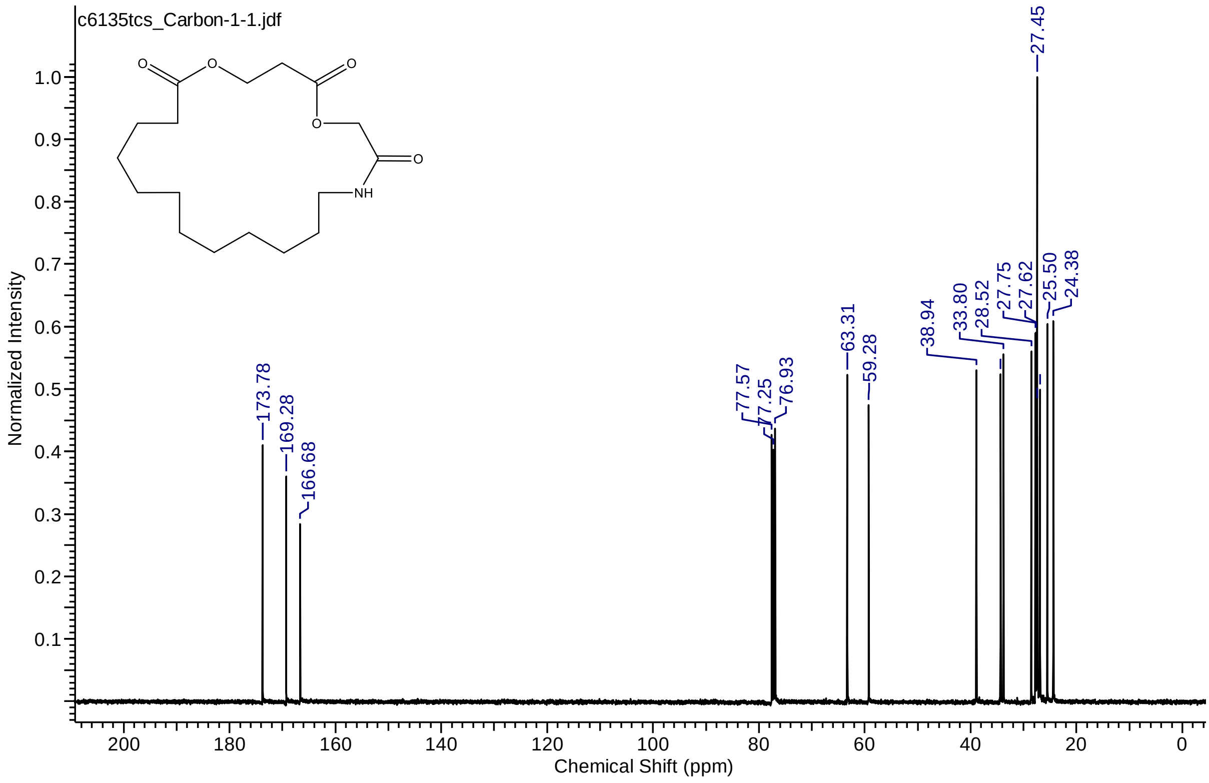

Compound 17k

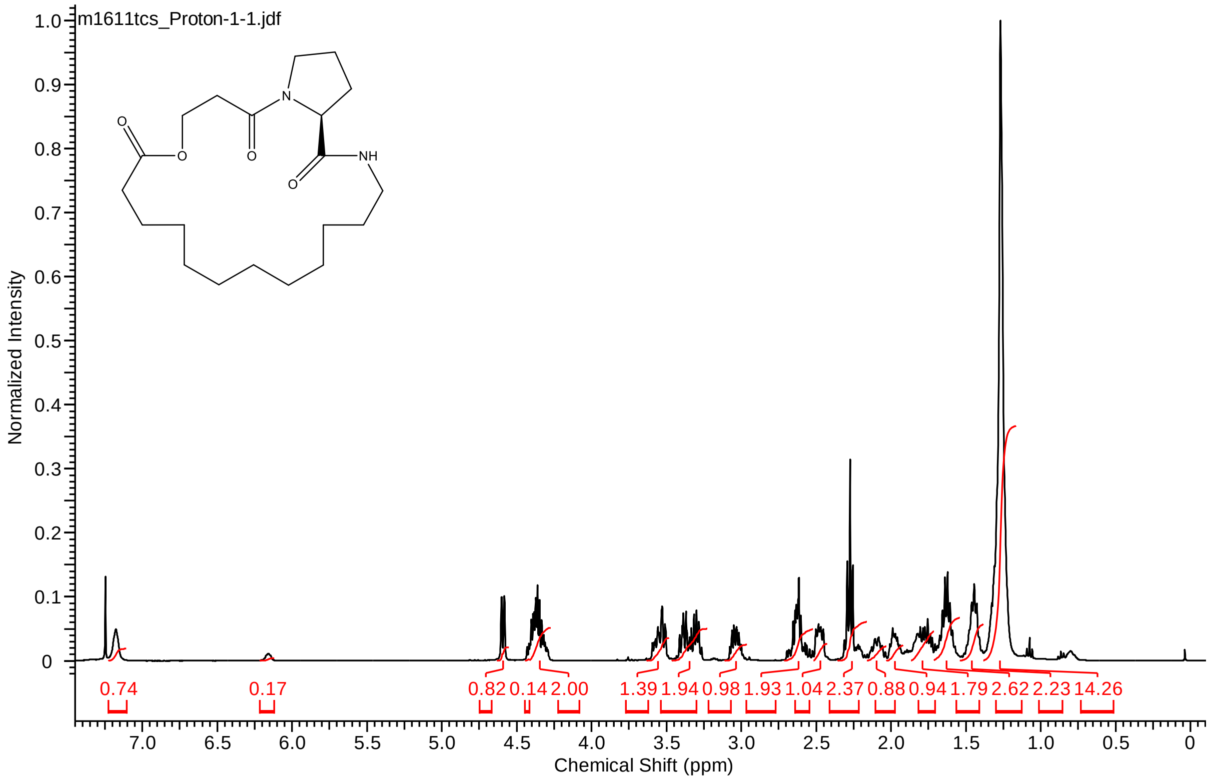

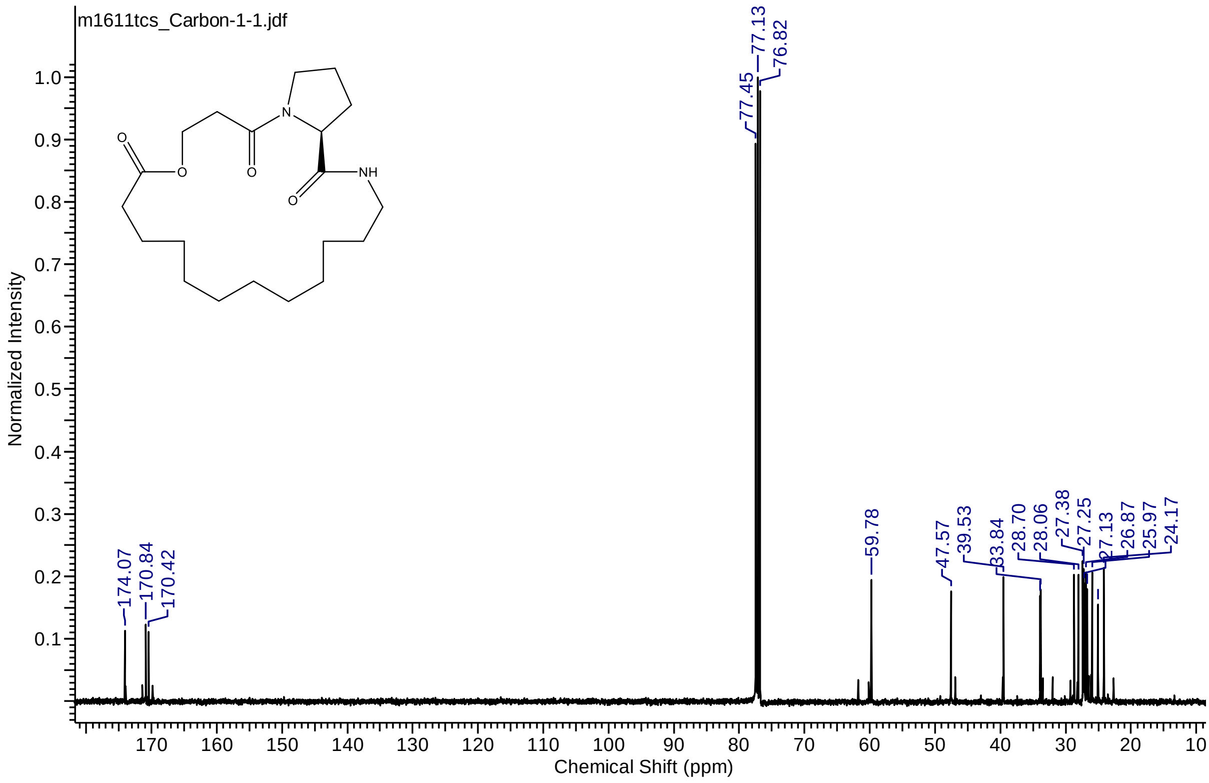

Compound 17l

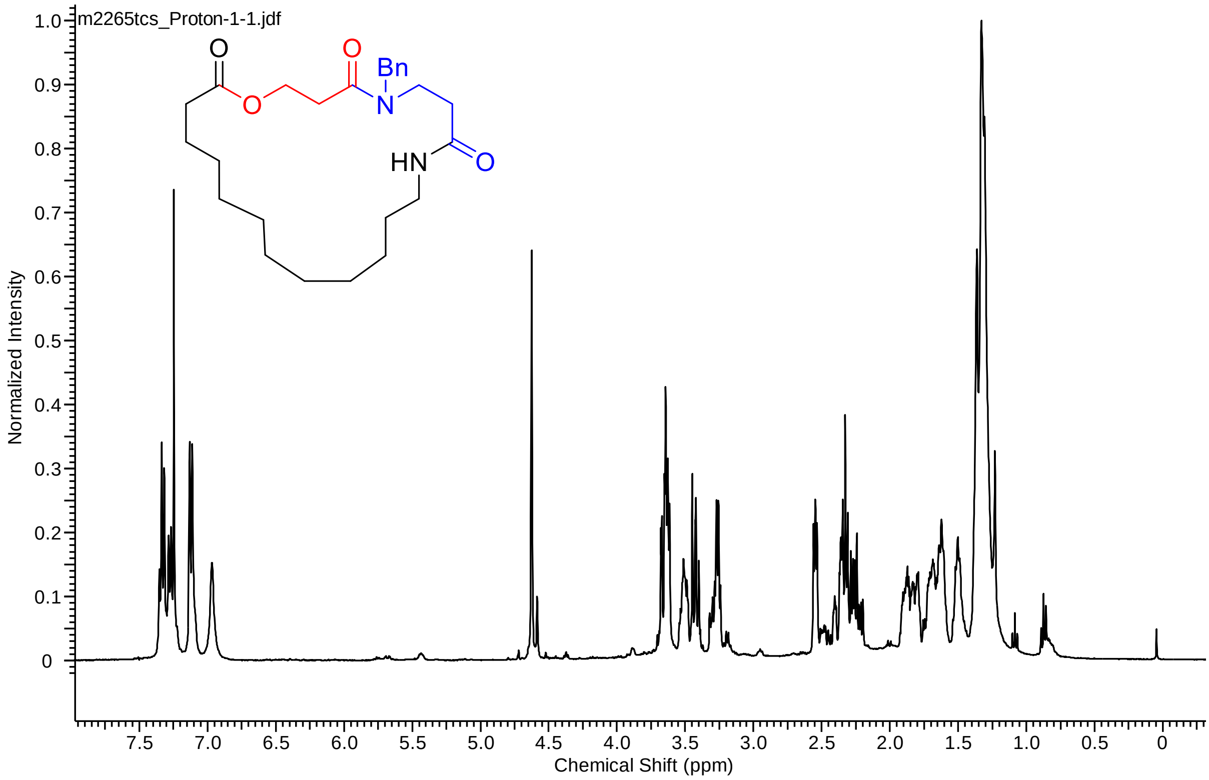

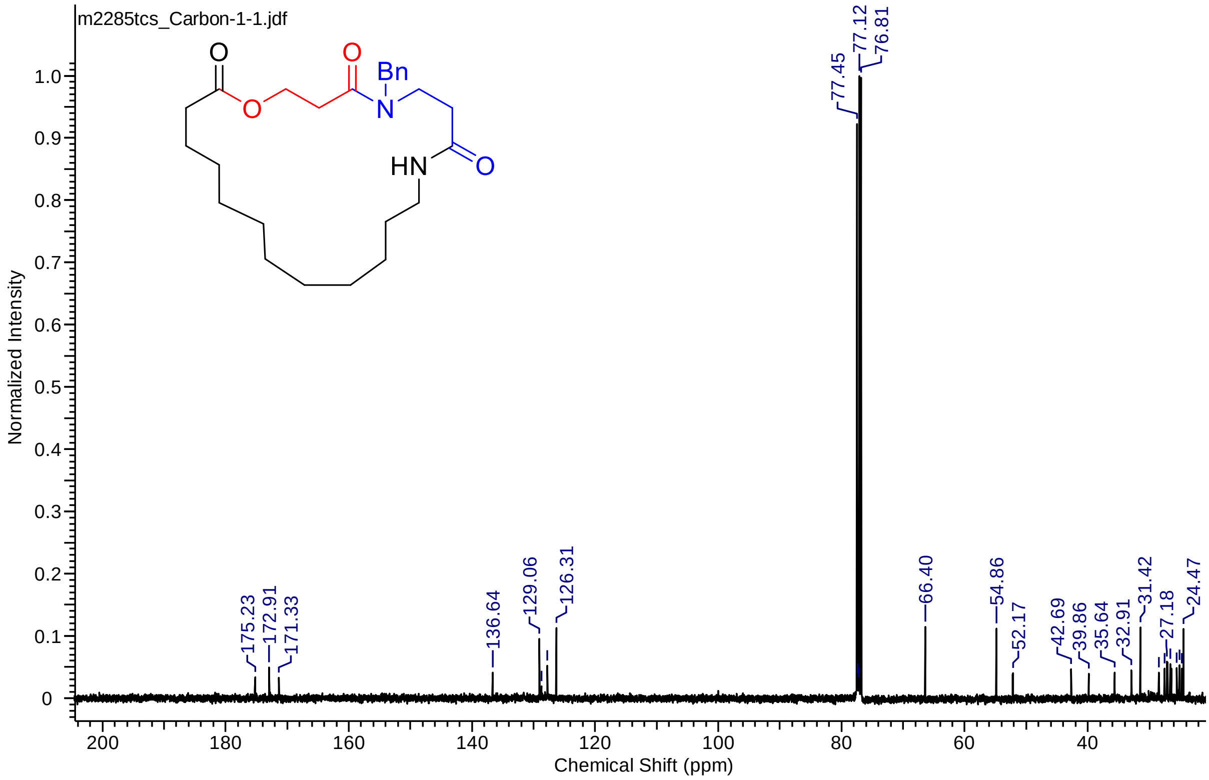

Compound 18a

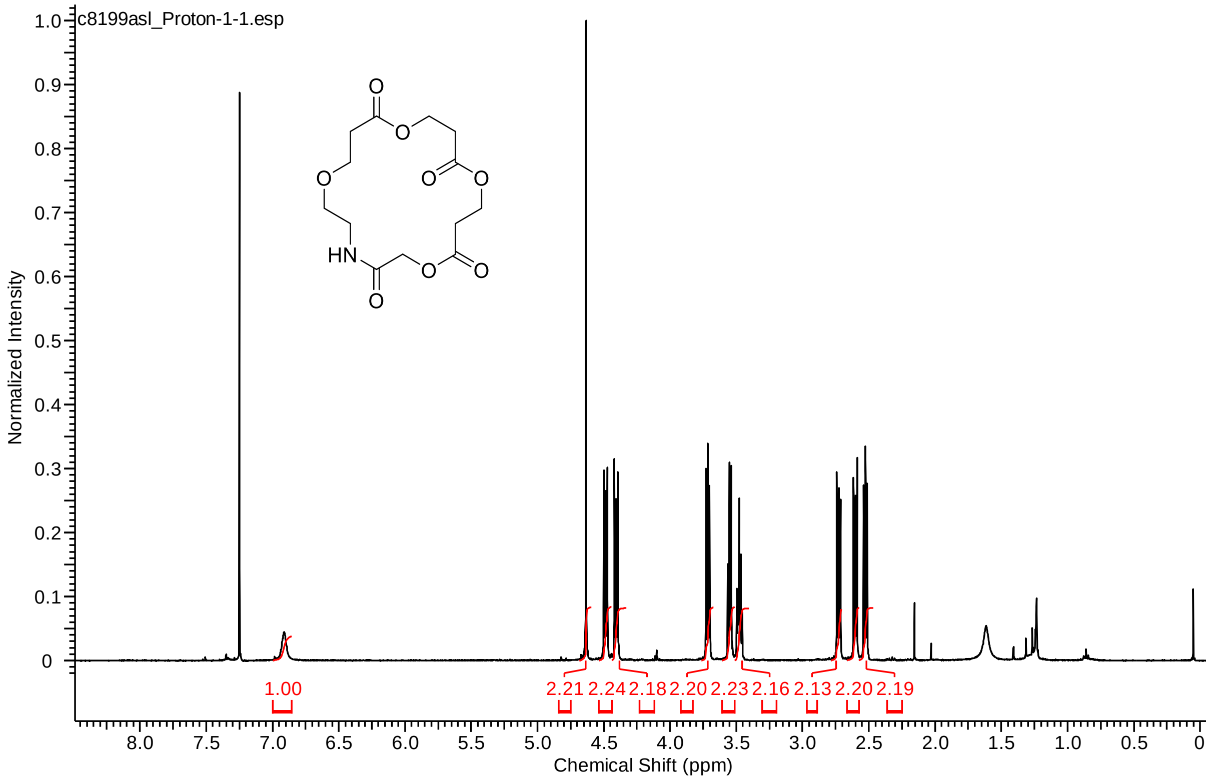

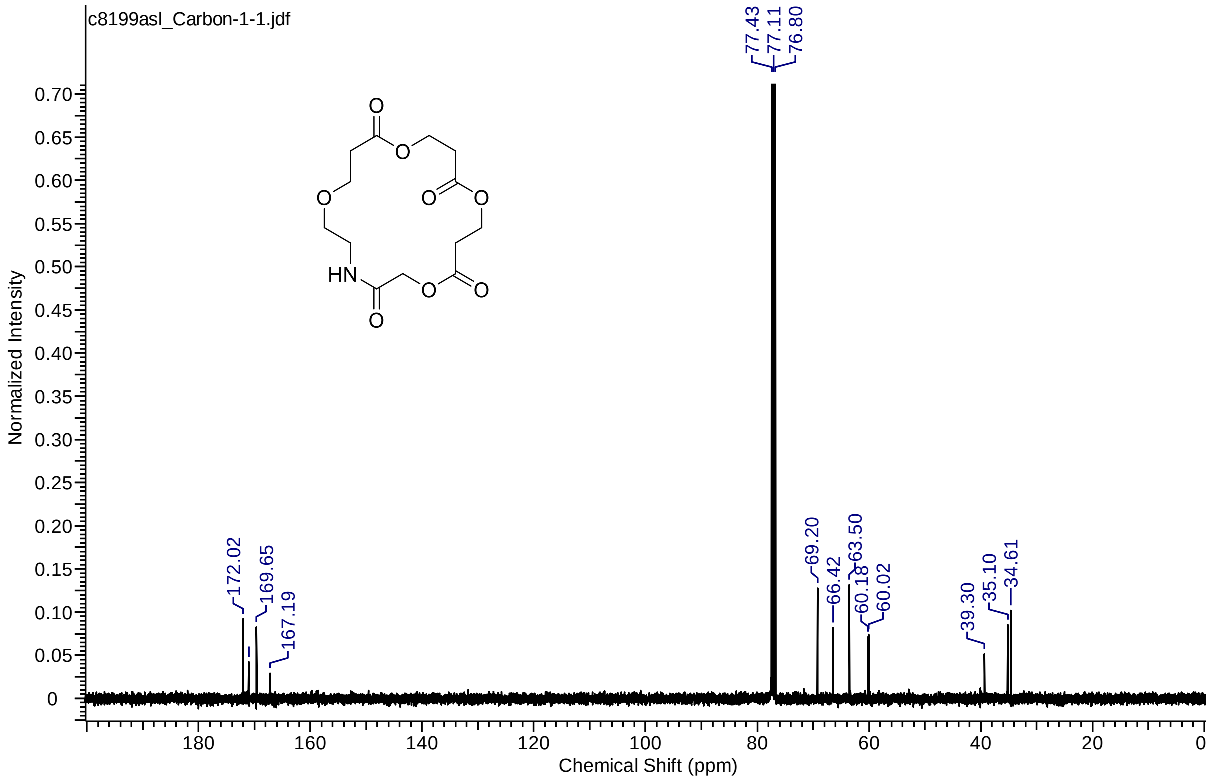

Compound 18b

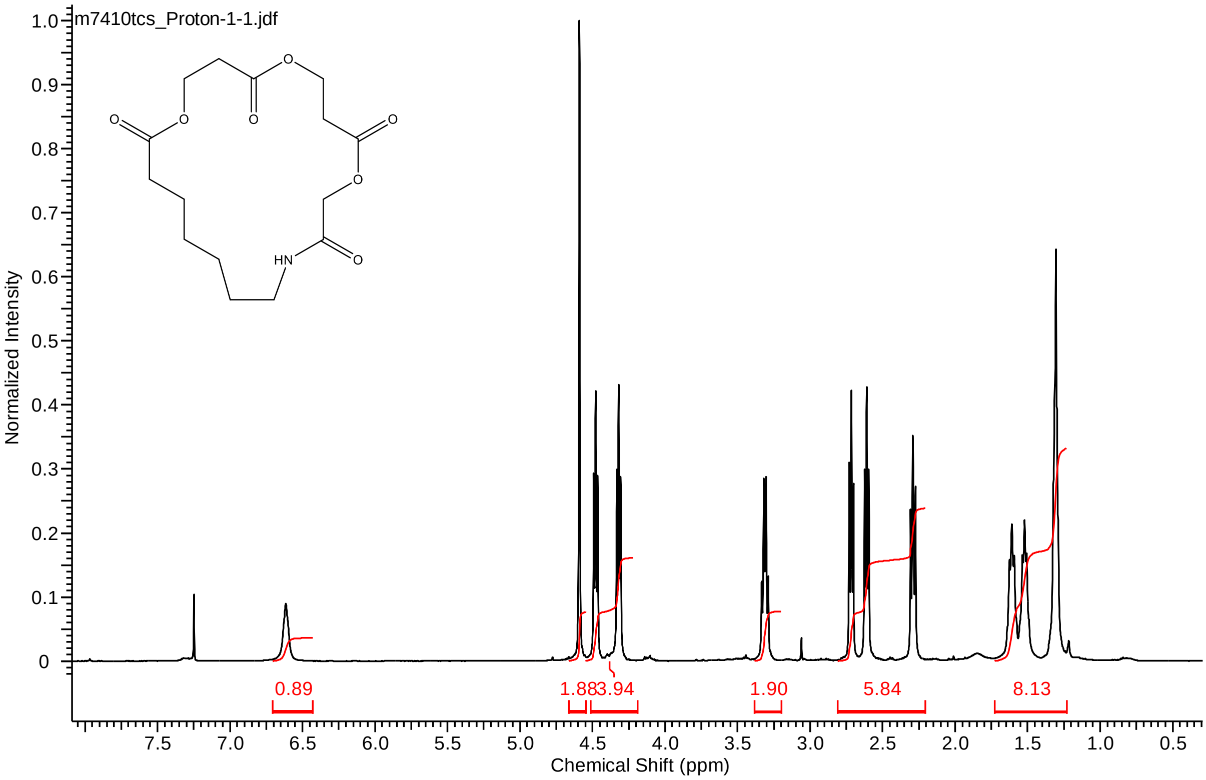

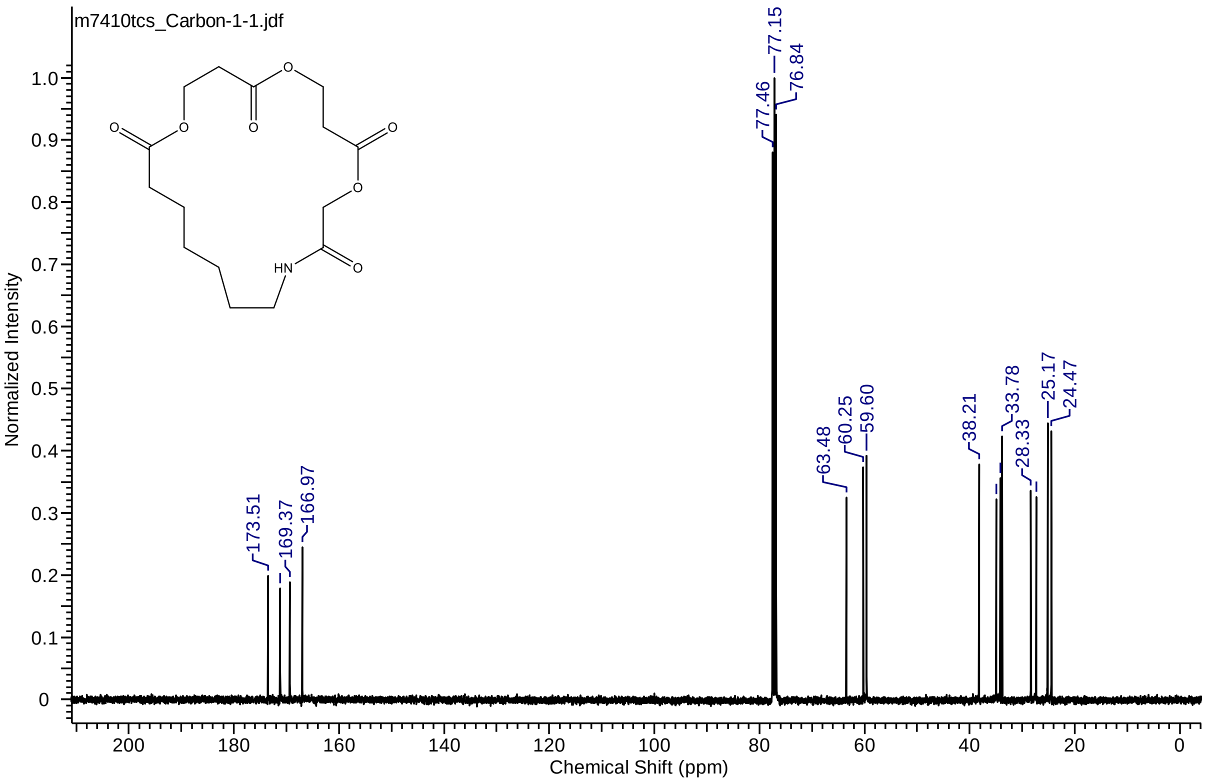

Compound 18c

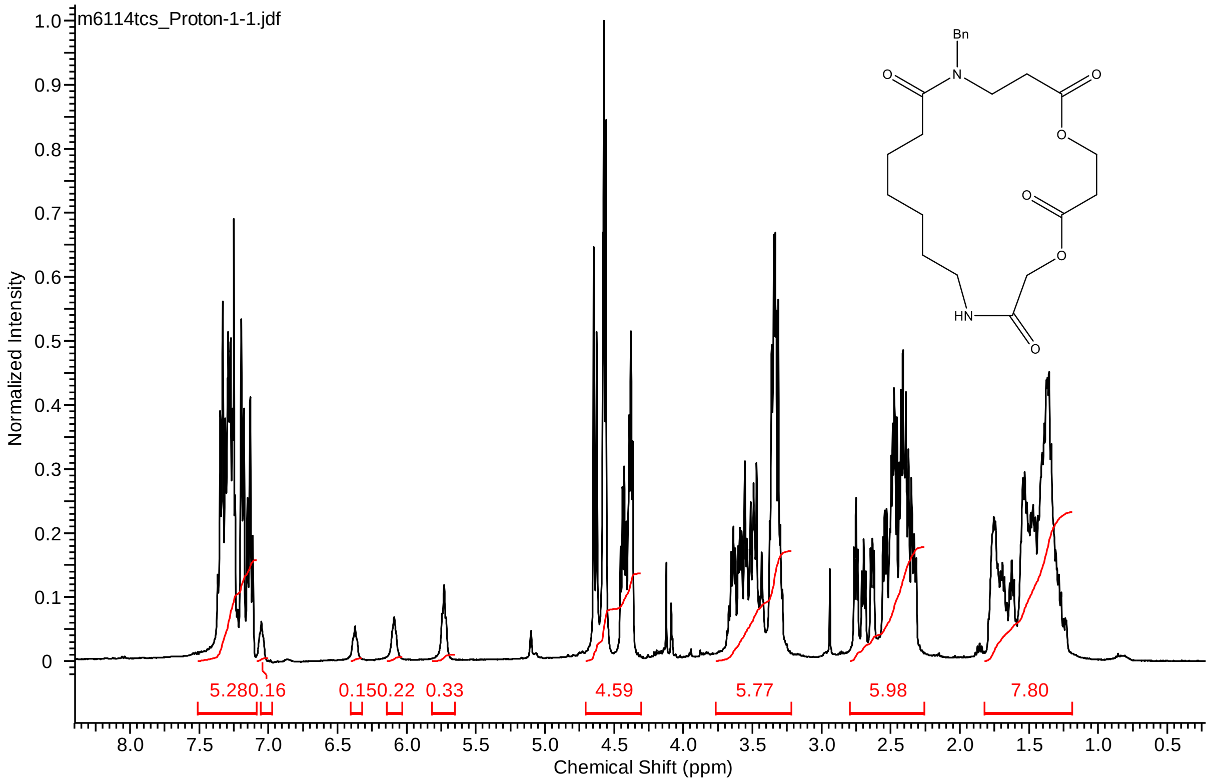

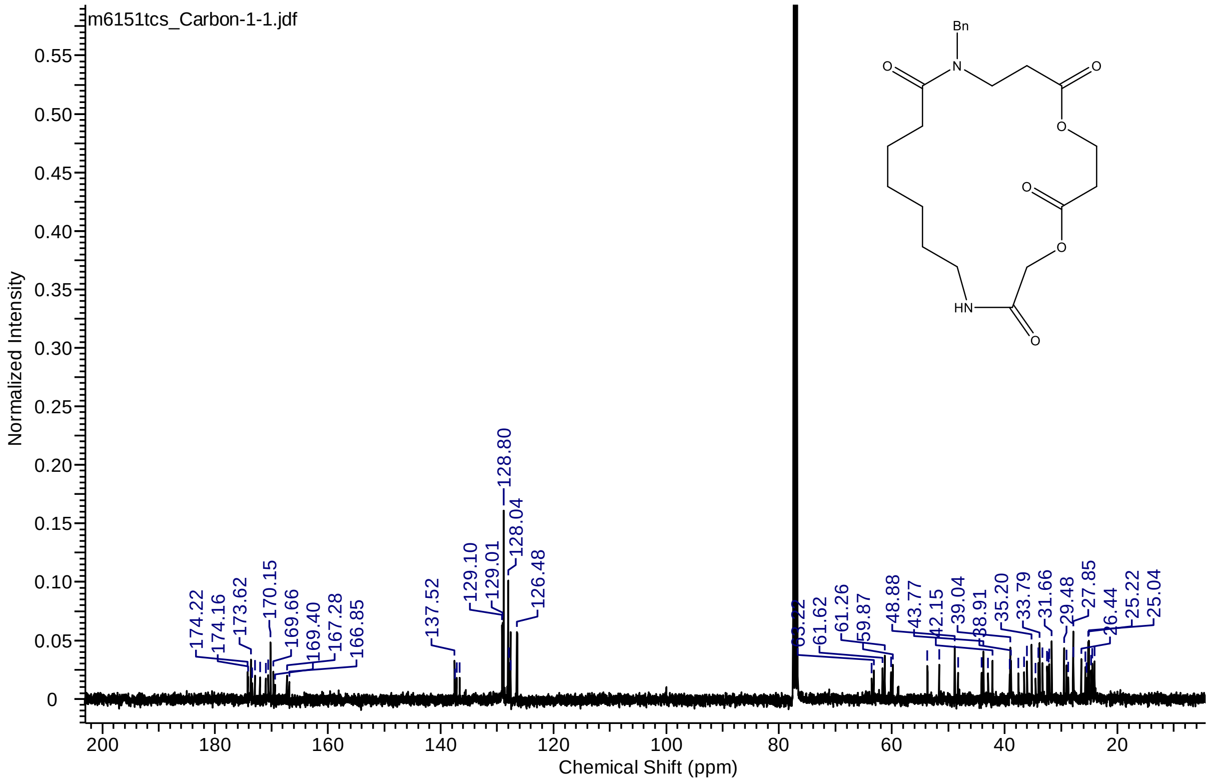

Compound 18d

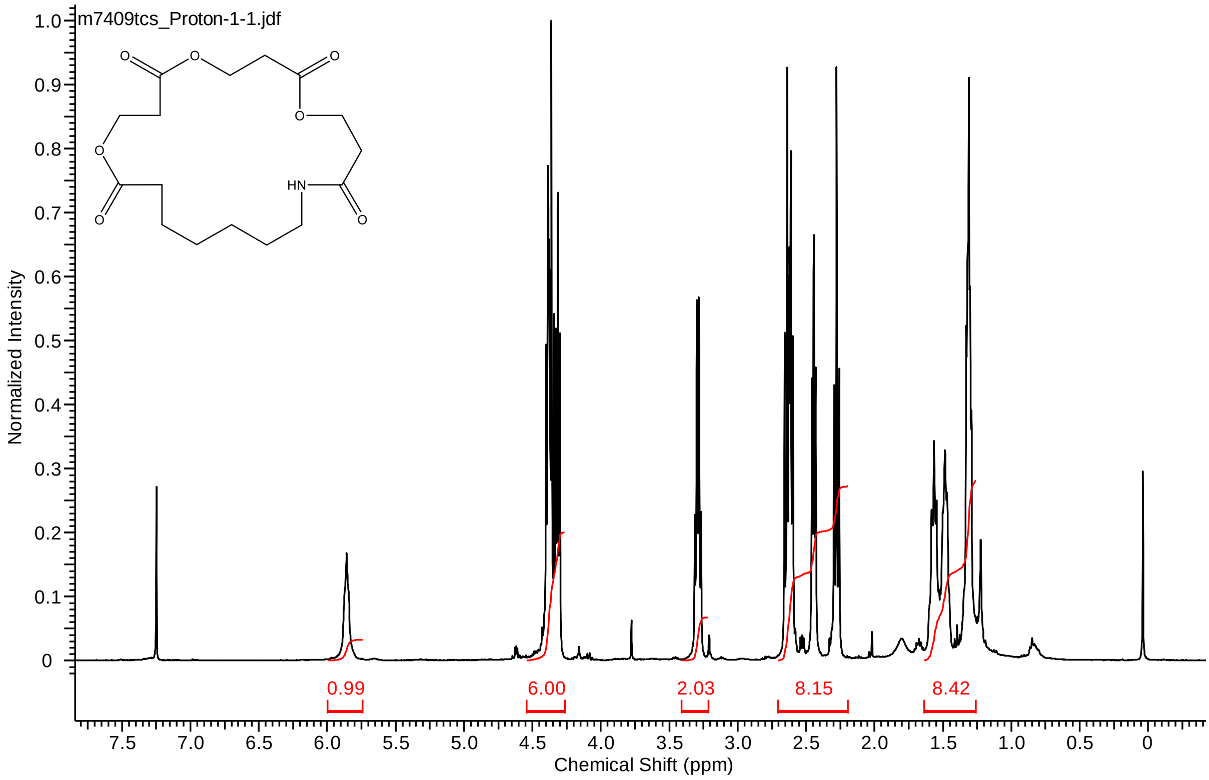

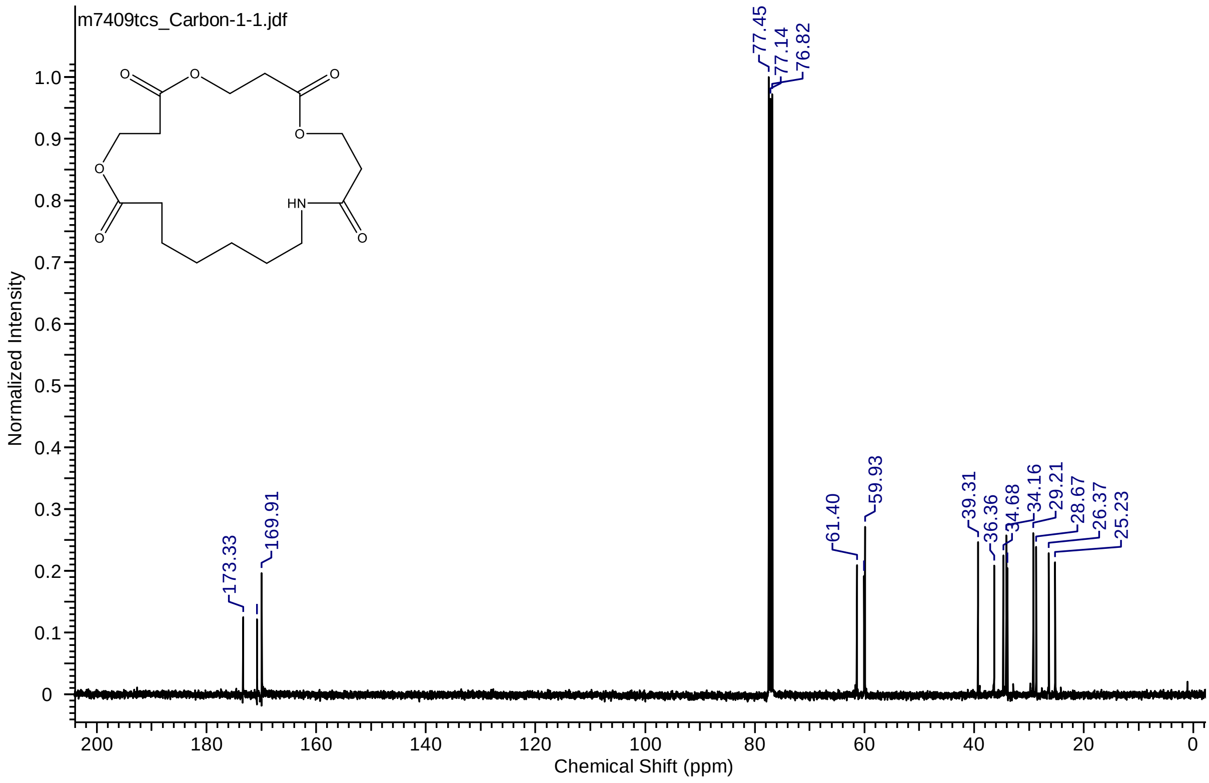

Compound 18e

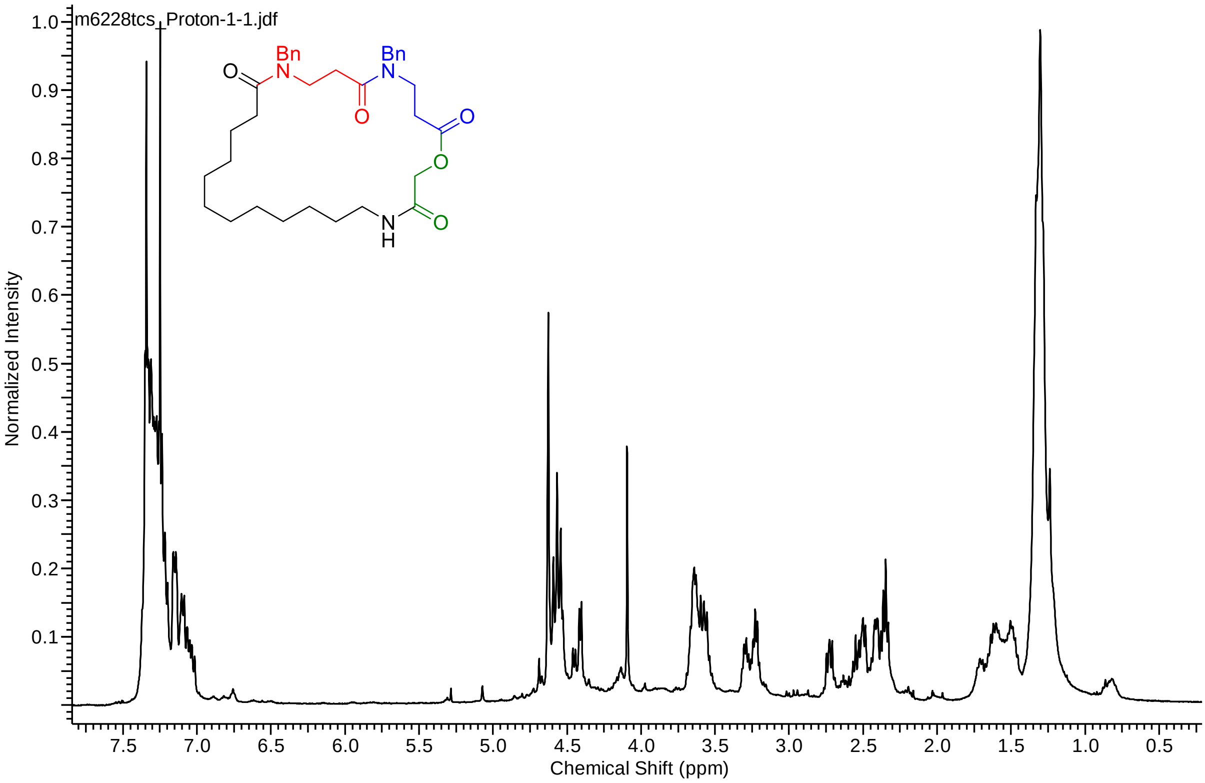

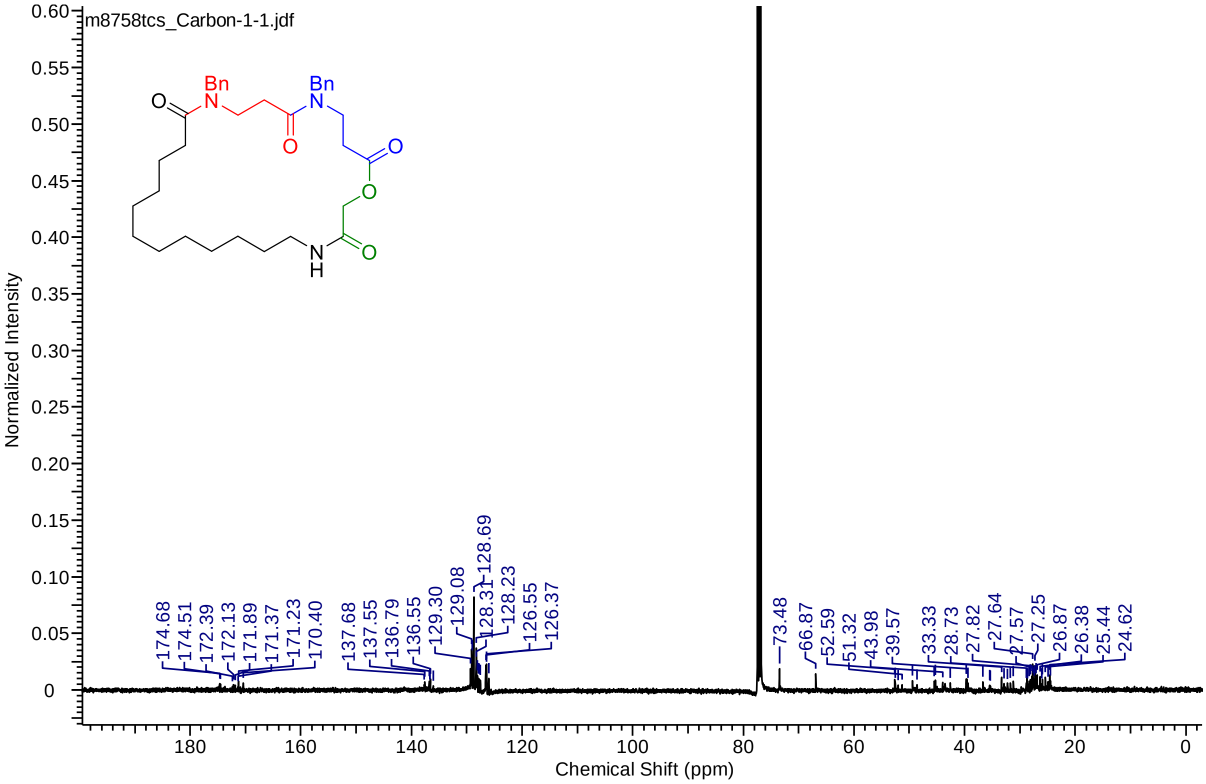

Computational studies of the selected systems

The imides, cyclols and ring expanded products in the chosen systems were initially built using either Spartan student[6] or Spartan'14[7] and optimised using Density Functional Theory (DFT)/B3LYP/6-31G*[8] in vacuum. Conformational searches of the optimised structures were performed at Molecular Mechanics Force Field (MMFF) level.[9] All the generated structures were retained and their energies were calculated using DFT/B3LYP/6-31G*. The lowest energy geometry in each case was selected, fully optimised and determined to be minima by the absence of negative vibrational modes, in vacuum using DFT/B3LYP/6-31G*. The final optimisations and frequency calculations were also done in solvated model system (non-polar solvents) using DFT/B3LYP/6-31G* and the results were similar to those performed in vacuum.

All the steps described above were also repeated using Hartree-Fock (HF)/6-31G*[10] in vacuum, with conformational searches performed at MMFF level. The final frequency calculations were done using HF/6-31G* in vacuum. All the results were in close agreement with those performed at the DFT level of theory.

Isomers 8c/10c/12c (HF/6-31G*)

Imide 8c	Calculated energy (au)	Calculated energy (kcal/mol)	Relative energy (kcal/mol)
M0001	-511.50317	-320968.24	0.00
M0002	-511.50224	-320967.65	0.58
M0003	-511.50147	-320967.17	1.07
**Cyclol 10c**	**Calculated energy (au)**	**Calculated energy (kcal/mol)**	**Relative energy (kcal/mol)**
M0002	-511.49052	-320960.30	0.00
M0001	-511.48681	-320957.97	2.33
**Lactone 12c**	**Calculated energy (au)**	**Calculated energy (kcal/mol)**	**Relative energy (kcal/mol)**
M0001	-511.49446	-320962.77	0.00
M0002	-511.48825	-320958.88	3.90
M0005	-511.48755	-320958.44	4.34
M0006	-511.48480	-320956.71	6.06
M0003	-511.48311	-320955.65	7.12
M0004	-511.48283	-320955.48	7.30

Table S1. Energies calculated at HF/6-31G* in vacuum for the conformers found for **8c/10c/12c**.

Isomers 8c/10c/12c (DFT/B3LYP/6-31G*)

Imide 8c	Calculated energy (au)	Calculated energy (kcal/mol)	Relative energy (kcal/mol)
M0002	-514.49102	-322843.12	0.00
M0001	-514.49073	-322842.93	0.18
M0003	-514.49069	-322842.90	0.21
M0004	-514.48144	-322837.10	6.01
M0005	-514.47912	-322835.65	7.47
M0007	-514.47299	-322831.80	11.32
M0006	-514.47288	-322831.73	11.38
**Cyclol 10c**	**Calculated energy (au)**	**Calculated energy (kcal/mol)**	**Relative energy (kcal/mol)**
M0002	-553.79414	-347505.83	0.00
M0003	-553.79186	-347504.39	1.44
M0001	-553.79104	-347503.88	1.95
M0006	-553.78898	-347502.59	3.24
M0005	-553.78703	-347501.36	4.47
M0004	-553.78560	-347500.46	5.36
M0008	-553.78554	-347500.43	5.40
M0007	-553.78429	-347499.64	6.18
**Lactone 12c**	**Calculated energy (au)**	**Calculated energy (kcal/mol)**	**Relative energy (kcal/mol)**
M0001	-514.48420	-322838.83	0.00
M0002	-514.48019	-322836.32	2.51
M0005	-514.47650	-322834.00	4.83
M0003	-514.47594	-322833.65	5.18
M0006	-514.47323	-322831.95	6.88
M0004	-514.47176	-322831.03	7.80

Table S2. Energies calculated at DFT/B3LYP/6-31G* in vacuum for the conformers found for **8c/10c/12c**.

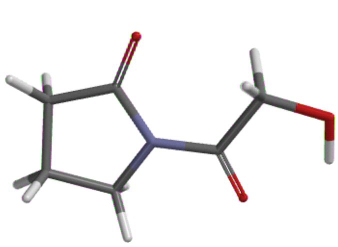

Imide **8c**

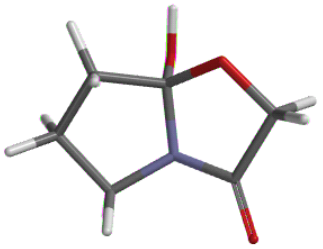

Cyclol **10c**

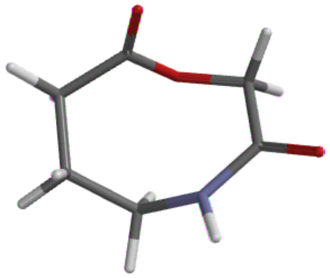

Lactone **12c**

**Isomer**	**ΔG° (kcal/mol)**		
	**DFT/B3LYP/6-31G***		**HF/6-31G***
	**Vacuum**	**Non-polar solvents**	**Vacuum**
Imide **8c**	0.0	0.0	0.0
Cyclol **10c**	13.8	13.4	12.9
Lactone **12c**	10.3	5.6	7.5

Table S3. Relative free energies (ΔG°) of the lowest energy geometries of **8c/10c/12c** at two levels of theory (DFT/B3LYP/6-31G* in a vacuum and in non-polar solvents) and HF/6-31G* in a vacuum) in kcal/mol.

Isomers 8d/10d/12d (HF/6-31G*)

**Imide 8d**	**Calculated energy (au)**	**Calculated energy (kcal/mol)**	**Relative energy (kcal/mol)**
M0001	-550.53075	-345458.04	0.00
M0002	-550.52956	-345457.30	0.75
M0003	-550.52631	-345455.26	2.78
M0004	-550.52300	-345453.18	4.86
**Cyclol 10d**	**Calculated energy (au)**	**Calculated energy (kcal/mol)**	**Relative energy (kcal/mol)**
M0002	-550.53850	-345462.91	0.00
M0003	-550.53479	-345460.58	2.33
M0001	-550.53438	-345460.32	2.59
M0005	-550.52851	-345456.64	6.27
M0006	-550.52708	-345455.74	7.17
M0004	-550.52431	-345454.00	8.91
**Lactone 12d**	**Calculated energy (au)**	**Calculated energy (kcal/mol)**	**Relative energy (kcal/mol)**
M0002	-550.52341	-345453.44	0.00
M0004	-550.52251	-345452.88	0.56
M0001	-550.52211	-345452.63	0.81
M0003	-550.52096	-345451.90	1.54
M0005	-550.51915	-345450.77	2.67
M0006	-550.51822	-345450.19	3.25

M0007	-550.51677	-345449.27	4.16
M0008	-550.51345	-345447.19	6.25
M0009	-550.51083	-345445.54	7.89
M0010	-550.50540	-345442.14	11.30

Table S4. Energies calculated at HF/6-31G* in vacuum for the conformers found for **8d/10d/12d**.

Isomers 8d/10d/12d (DFT/B3LYP/6-31G*)

Imide 8d	Calculated energy (au)	Calculated energy (kcal/mol)	Relative energy (kcal/mol)
M0002	-553.79916	-347508.97	0.00
M0001	-553.79906	-347508.91	0.06
M0003	-553.79745	-347507.90	1.07
M0004	-553.78963	-347502.99	5.98
M0007	-553.78318	-347498.95	10.02
M0005	-553.77769	-347495.50	13.47
M0006	-553.77694	-347495.03	13.94
**Cyclol 10d**	**Calculated energy (au)**	**Calculated energy (kcal/mol)**	**Relative energy (kcal/mol)**
M0002	-553.80092	-347510.08	0.00
M0001	-553.79785	-347508.15	1.93
M0004	-553.79776	-347508.10	1.98
M0003	-553.79712	-347507.69	2.38
M0006	-553.79148	-347504.15	5.92
M0005	-553.78794	-347501.93	8.14
M0007	-553.78678	-347501.20	8.87
**Lactone 12d**	**Calculated energy (au)**	**Calculated energy (kcal/mol)**	**Relative energy (kcal/mol)**
M0002	-553.79640	-347507.24	0.00
M0001	-553.79555	-347506.71	0.53
M0004	-553.79347	-347505.40	1.84
M0003	-553.79238	-347504.72	2.52
M0006	-553.79116	-347503.96	3.28
M0005	-553.79067	-347503.64	3.60
M0007	-553.79042	-347503.49	3.75
M0008	-553.78429	-347499.64	7.60
M0009	-553.78262	-347498.60	8.65
M0010	-553.78062	-347497.34	9.90
M0011	-553.78027	-347497.12	10.12
M0012	-553.71899	-347458.66	48.58
M0013	-553.71427	-347455.71	51.53

Table S5. Energies calculated at DFT/B3LYP/6-31G* in vacuum for the conformers found for **8d/10d/12d**.

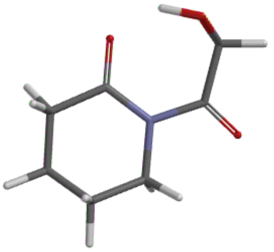

Imide **8d**

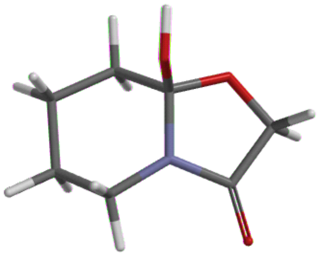

Cyclol **10d**

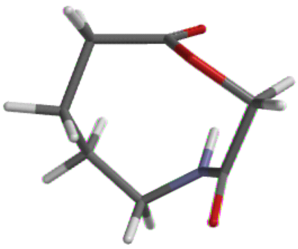

Lactone **12d**

**Isomer**	**ΔG° (kcal/mol)**		
	**DFT/B3LYP/6-31G***		**HF/6-31G***
	**Vacuum**	**Non-polar solvents**	**Vacuum**
Imide **8d**	0.0	0.0	0.0
Cyclol **10d**	2.9	2.3	1.6
Lactone **12d**	5.2	5.2	7.6

Table S6. Relative free energies (ΔG°) of the lowest energy geometries of **8d/10d/12d** at two levels of theory (DFT/B3LYP/6-31G* in a vacuum and in non-polar solvents) and HF/6-31G* in a vacuum) in kcal/mol.

Isomers 8e/10e/12e (HF/6-31G*)

**Imide 8e**	**Calculated energy (au)**	**Calculated energy (kcal/mol)**	**Relative energy (kcal/mol)**
M0001	-589.55998	-369948.89	0.00
M0003	-589.55789	-369947.57	1.32
M0002	-589.55764	-369947.42	1.47
M0004	-589.55621	-369946.52	2.37
M0006	-589.55455	-369945.48	3.41
M0007	-589.55328	-369944.68	4.21
M0005	-589.55103	-369943.27	5.61
M0010	-589.54987	-369942.55	6.34
M0008	-589.54912	-369942.07	6.82
M0009	-589.54796	-369941.34	7.55
M0012	-589.54767	-369941.17	7.72
M0011	-589.54718	-369940.85	8.04
M0014	-589.54615	-369940.21	8.68
M0013	-589.54116	-369937.08	11.81
**Cyclol 10e**	**Calculated energy (au)**	**Calculated energy (kcal/mol)**	**Relative energy (kcal/mol)**
M0002	-589.56664	-369953.07	0.00
M0006	-589.56417	-369951.52	1.55
M0007	-589.56307	-369950.83	2.24
M0004	-589.56165	-369949.93	3.13
M0005	-589.56126	-369949.69	3.38

M0012	-589.56014	-369948.98	4.08
M0011	-589.55991	-369948.84	4.22
M0001	-589.55844	-369947.92	5.14
M0003	-589.55843	-369947.92	5.15
M0008	-589.55779	-369947.51	5.55
M0009	-589.55662	-369946.78	6.29
M0016	-589.55412	-369945.21	7.85
M0013	-589.55401	-369945.14	7.92
M0010	-589.55294	-369944.47	8.60
M0014	-589.55060	-369943.00	10.06
M0015	-589.54899	-369941.99	11.07
**Lactone 12e**	**Calculated energy (au)**	**Calculated energy (kcal/mol)**	**Relative energy (kcal/mol)**
M0006	-589.56389	-369951.34	0.00
M0002	-589.56381	-369951.29	0.05
M0003	-589.56356	-369951.13	0.21
M0001	-589.56194	-369950.12	1.23
M0008	-589.56184	-369950.06	1.29
M0011	-589.55937	-369948.50	2.84
M0004	-589.55914	-369948.36	2.98
M0007	-589.55877	-369948.13	3.21
M0014	-589.55877	-369948.13	3.21
M0005	-589.55826	-369947.81	3.54
M0010	-589.55773	-369947.47	3.87
M0009	-589.55655	-369946.74	4.61
M0016	-589.55562	-369946.15	5.19
M0013	-589.55545	-369946.04	5.30
M0012	-589.55497	-369945.75	5.60
M0015	-589.55304	-369944.53	6.81
M0017	-589.55298	-369944.49	6.85

Table S7. Energies calculated at HF/6-31G* in vacuum for the conformers found for **8e/10e/12e**.

Isomers 8e/10e/12e (DFT/B3LYP/6-31G*)

Imide 10e	Calculated energy (au)	Calculated energy (kcal/mol)	Relative energy (kcal/mol)
M0001	-593.10983	-372176.42	0.00
M0003	-593.10894	-372175.86	0.56
M0002	-593.10884	-372175.79	0.62
M0004	-593.10679	-372174.51	1.91
M0006	-593.10613	-372174.10	2.32
M0007	-593.10559	-372173.76	2.66
M0005	-593.09940	-372169.87	6.54
M0010	-593.09934	-372169.83	6.58

M0008	-593.09843	-372169.26	7.16
M0009	-593.09832	-372169.19	7.22
M0015	-593.09602	-372167.75	8.67
M0012	-593.09520	-372167.24	9.18
M0018	-593.09354	-372166.20	10.22
M0011	-593.09341	-372166.12	10.30
M0016	-593.09316	-372165.96	10.46
M0013	-593.08918	-372163.46	12.96
M0014	-593.08641	-372161.72	14.69
M0019	-593.08617	-372161.57	14.84
M0017	-593.08466	-372160.63	15.79
M0021	-593.08413	-372160.29	16.12
M0023	-593.08362	-372159.97	16.45
M0022	-593.08268	-372159.38	17.04
M0025	-593.07867	-372156.87	19.55
M0020	-593.07616	-372155.29	21.13
M0024	-593.06868	-372150.59	25.82
**Cyclol 10e**	**Calculated energy (au)**	**Calculated energy (kcal/mol)**	**Relative energy (kcal/mol)**
M0003	-593.10988	-372176.45	0.00
M0005	-593.10784	-372175.17	1.28
M0007	-593.10660	-372174.39	2.06
M0006	-593.10513	-372173.47	2.98
M0001	-593.10490	-372173.33	3.12
M0009	-593.10352	-372172.46	3.99
M0002	-593.10308	-372172.18	4.27
M0004	-593.10264	-372171.91	4.54
M0008	-593.10120	-372171.00	5.45
M0012	-593.09877	-372169.48	6.97
M0010	-593.09876	-372169.47	6.98
M0011	-593.09437	-372166.72	9.73
**Lactone 12e**	**Calculated energy (au)**	**Calculated energy (kcal/mol)**	**Relative energy (kcal/mol)**
M0002	-593.11479	-372179.53	0.00
M0001	-593.11449	-372179.34	0.19
M0003	-593.11279	-372178.28	1.25
M0004	-593.11192	-372177.73	1.80
M0009	-593.11117	-372177.26	2.27
M0006	-593.11061	-372176.91	2.62
M0005	-593.10946	-372176.19	3.34
M0007	-593.10897	-372175.88	3.65
M0010	-593.10793	-372175.22	4.31
M0008	-593.10654	-372174.35	5.18
M0011	-593.10417	-372172.86	6.66
M0012	-593.10262	-372171.89	7.64
M0013	-593.09724	-372168.52	11.01

M0015	-593.06874	-372150.63	28.90
M0014	-593.06766	-372149.95	29.58

Table S8. Energies calculated at DFT/B3LYP/6-31G* in vacuum for the conformers found for **8e/10e/12e**.

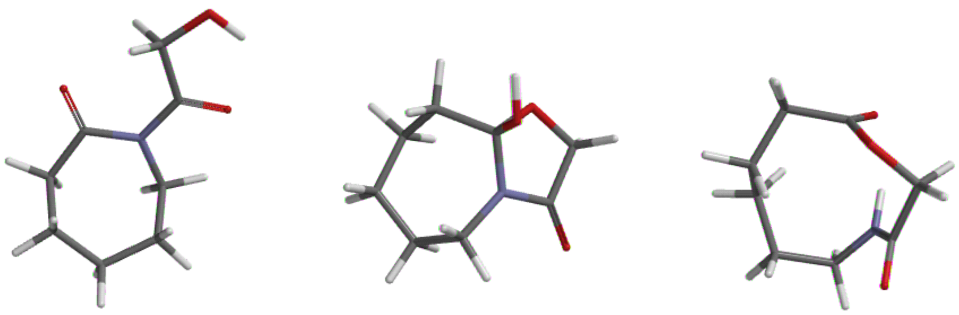

Imide **8e** Cyclol **10e** Lactone **12e**

Isomer	ΔG° (kcal/mol)		
	DFT/B3LYP/6-31G*		HF/6-31G*
	Vacuum	Non-polar solvents	Vacuum
Imide **8e**	0.0	0.3	0.0
Cyclol **10e**	4.0	4.0	1.6
Lactone **12e**	0.7	0.0	0.4

Table S9. Relative free energies (ΔG°) of the lowest energy geometries of **8e/10e/12e** at two levels of theory (DFT/B3LYP/6-31G* in a vacuum and in non-polar solvents) and HF/6-31G* in a vacuum) in kcal/mol.

Isomers 8f/10f/12f (HF/6-31G*)

Imide 8f	Calculated energy (au)	Calculated energy (kcal/mol)	Relative energy (kcal/mol)
M0001	-628.588521	-394439.30	0.00
M0004	-628.586765	-394438.20	1.10
M0002	-628.586563	-394438.07	1.23
M0003	-628.58646	-394438.00	1.29
M0005	-628.585037	-394437.11	2.19
M0006	-628.583561	-394436.18	3.11
M0014	-628.580689	-394434.38	4.91
M0011	-628.577911	-394432.64	6.66
M0007	-628.577456	-394432.35	6.94
M0008	-628.57695	-394432.04	7.26
M0013	-628.576881	-394431.99	7.30
M0019	-628.576081	-394431.49	7.81
M0009	-628.575903	-394431.38	7.92
M0012	-628.575651	-394431.22	8.08
M0015	-628.574718	-394430.64	8.66

M0010	-628.573991	-394430.18	9.12
M0027	-628.572849	-394429.46	9.83
M0024	-628.571166	-394428.41	10.89
M0023	-628.569758	-394427.52	11.77
M0020	-628.569466	-394427.34	11.96
M0018	-628.568991	-394427.04	12.26
M0016	-628.567459	-394426.08	13.22
M0022	-628.567402	-394426.04	13.25
M0017	-628.567365	-394426.02	13.28
M0030	-628.565113	-394424.61	14.69
M0021	-628.564544	-394424.25	15.05
M0025	-628.564347	-394424.13	15.17
M0026	-628.563807	-394423.79	15.51
M0028	-628.563444	-394423.56	15.74
M0029	-628.562096	-394422.72	16.58
**Cyclol 10f**	**Calculated energy (au)**	**Calculated energy (kcal/mol)**	**Relative energy (kcal/mol)**
M0002	-628.595191	-394443.48	0.00
M0004	-628.592596	-394441.85	1.63
M0009	-628.591046	-394440.88	2.60
M0001	-628.58919	-394439.72	3.77
M0005	-628.589132	-394439.68	3.80
M0013	-628.586472	-394438.01	5.47
M0003	-628.585878	-394437.64	5.84
M0008	-628.585426	-394437.35	6.13
M0006	-628.58537	-394437.32	6.16
M0010	-628.585133	-394437.17	6.31
M0012	-628.584805	-394436.97	6.52
M0007	-628.583219	-394435.97	7.51
M0011	-628.581655	-394434.99	8.49
M0014	-628.580926	-394434.53	8.95
M0015	-628.580635	-394434.35	9.13
M0016	-628.580056	-394433.99	9.50
**Lactone 12f**	**Calculated energy (au)**	**Calculated energy (kcal/mol)**	**Relative energy (kcal/mol)**
M0002	-628.601551	-394447.47	0.00
M0004	-628.601221	-394447.27	0.21
M0003	-628.600415	-394446.76	0.71
M0006	-628.599852	-394446.41	1.07
M0005	-628.599573	-394446.23	1.24
M0012	-628.598971	-394445.85	1.62
M0001	-628.598847	-394445.78	1.70
M0008	-628.59755	-394444.96	2.51
M0007	-628.597237	-394444.77	2.71
M0011	-628.597219	-394444.75	2.72
M0009	-628.597036	-394444.64	2.83

M0013	-628.595472	-394443.66	3.81
M0015	-628.594454	-394443.02	4.45
M0016	-628.594353	-394442.96	4.52
M0027	-628.593886	-394442.66	4.81
M0014	-628.593805	-394442.61	4.86
M0025	-628.593701	-394442.55	4.93
M0019	-628.593456	-394442.39	5.08
M0010	-628.593328	-394442.31	5.16
M0029	-628.593161	-394442.21	5.26
M0020	-628.593048	-394442.14	5.34
M0021	-628.592867	-394442.02	5.45
M0018	-628.592854	-394442.02	5.46
M0022	-628.592676	-394441.90	5.57
M0017	-628.592554	-394441.83	5.65
M0026	-628.592328	-394441.69	5.79
M0023	-628.591981	-394441.47	6.01
M0030	-628.591764	-394441.33	6.14
M0032	-628.591152	-394440.95	6.53
M0028	-628.591106	-394440.92	6.55
M0024	-628.590552	-394440.57	6.90
M0033	-628.590151	-394440.32	7.15
M0031	-628.589347	-394439.82	7.66

Table S10. Energies calculated at HF/6-31G* in vacuum for the conformers found for **8f/10f/12f**.

Isomers 8f/10f/12f (DFT/B3LYP/6-31G*)

Imide 8f	Calculated energy (au)	Calculated energy (kcal/mol)	Relative energy (kcal/mol)
M0001	-632.41713	-396841.75	0.00
M0002	-632.41625	-396841.20	0.55
M0003	-632.40802	-396836.03	5.71
M0004	-632.40662	-396835.16	6.59
M0005	-632.40735	-396835.61	6.13
M0006	-632.39614	-396828.58	13.17
M0007	-632.39759	-396829.49	12.26
M0008	-632.40707	-396835.43	6.31
M0009	-632.40060	-396831.38	10.37
M0010	-632.39907	-396830.41	11.33
M0011	-632.39247	-396826.27	15.47
M0012	-632.39578	-396828.35	13.40
M0013	-632.40188	-396832.18	9.57
M0014	-632.39226	-396826.14	15.60
M0015	-632.40136	-396831.85	9.89
M0016	-632.39088	-396825.28	16.47

M0017	-632.39651	-396828.81	12.94
M0018	-632.39381	-396827.12	14.63
M0019	-632.38612	-396822.29	19.45
M0020	-632.39919	-396830.49	11.25
M0021	-632.40519	-396834.26	7.49
M0022	-632.40556	-396834.49	7.26
M0023	-632.39316	-396826.70	15.04
M0024	-632.39121	-396825.48	16.26
M0025	-632.38645	-396822.50	19.25
M0026	-632.39228	-396826.16	15.59
M0027	-632.39935	-396830.59	11.15
M0028	-632.39946	-396830.66	11.08
M0029	-632.38624	-396822.37	19.38
M0030	-632.40046	-396831.29	10.46
M0031	-632.39361	-396826.99	14.75
M0032	-632.39700	-396829.12	12.63
M0033	-632.38222	-396819.84	21.91
M0034	-632.39213	-396826.06	15.69
M0035	-632.39093	-396825.31	16.44
M0036	-632.38680	-396822.72	19.03
M0037	-632.38306	-396820.37	21.38
**Cyclol 10f**	**Calculated energy (au)**	**Calculated energy (kcal/mol)**	**Relative energy (kcal/mol)**
M0002	-632.41840	-396842.54	0.00
M0004	-632.41599	-396841.03	1.51
M0009	-632.41508	-396840.46	2.08
M0005	-632.41361	-396839.54	3.00
M0001	-632.41328	-396839.33	3.21
M0012	-632.41045	-396837.56	4.98
M0008	-632.41029	-396837.45	5.09
M0003	-632.41027	-396837.44	5.10
M0006	-632.41015	-396837.37	5.18
M0010	-632.41014	-396837.36	5.18
M0011	-632.40932	-396836.85	5.70
M0007	-632.40867	-396836.44	6.10
M0013	-632.40696	-396835.37	7.18
M0014	-632.40565	-396834.54	8.00
**Lactone 12f**	**Calculated energy (au)**	**Calculated energy (kcal/mol)**	**Relative energy (kcal/mol)**
M0001	-632.43034	-396850.04	0.00
M0002	-632.42955	-396849.54	0.49
M0003	-632.42950	-396849.51	0.53
M0004	-632.42878	-396849.06	0.98
M0010	-632.42770	-396848.38	1.65
M0007	-632.42769	-396848.37	1.66
M0006	-632.42764	-396848.34	1.70

M0005	-632.42697	-396847.92	2.12
M0009	-632.42656	-396847.67	2.37
M0013	-632.42408	-396846.11	3.93
M0012	-632.42405	-396846.09	3.95
M0008	-632.42378	-396845.92	4.11
M0011	-632.42364	-396845.83	4.21
M0015	-632.42324	-396845.58	4.46
M0019	-632.42279	-396845.30	4.74
M0016	-632.42276	-396845.28	4.76
M0020	-632.42239	-396845.05	4.99
M0017	-632.42213	-396844.89	5.15
M0014	-632.42170	-396844.62	5.42
M0021	-632.42068	-396843.97	6.06
M0022	-632.42059	-396843.92	6.12
M0018	-632.41977	-396843.41	6.63
M0024	-632.41214	-396838.62	11.42
M0023	-632.41027	-396837.44	12.60
M0025	-632.39233	-396826.19	23.85

Table S11. Energies calculated at DFT/B3LYP/6-31G* in vacuum for the conformers found for **8f/10f/12f**.

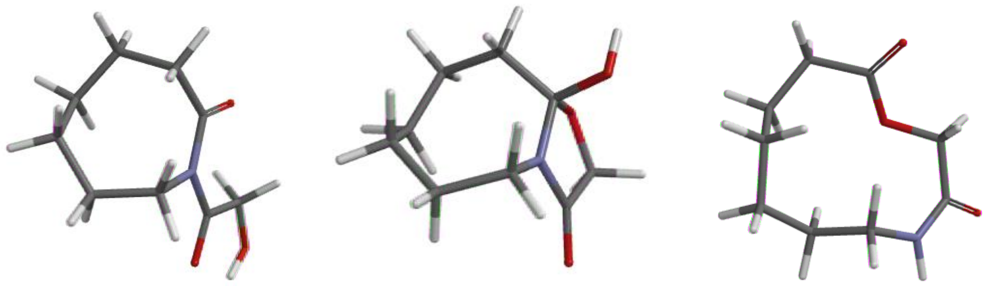

Imide **8f** Cyclol **10f** Lactone **12f**

Isomer	ΔG° (kcal/mol)		
	DFT/B3LYP/6-31G*		HF/6-31G*
	Vacuum	Non-polar solvents	Vacuum
Imide **8f**	6.3	7.1	6.3
Cyclol **10f**	8.9	9.6	7.4
Lactone **12f**	0.0	0.0	0.0

Table S12. Relative free energies (ΔG°) of the lowest energy geometries of **8f/10f/12f** at two levels of theory (DFT/B3LYP/6-31G* in a vacuum and in non-polar solvents) and HF/6-31G* in a vacuum) in kcal/mol.

Isomers 9c/11c/13c (HF/6-31G*)

Imide 9c	Calculated energy (au)	Calculated energy (kcal/mol)	Relative energy (kcal/mol)
M0002	-550.54221	-345465.24	0.00
M0001	-550.54191	-345465.05	0.19
M0010	-550.53756	-345462.32	2.92
M0007	-550.53755	-345462.31	2.92
M0008	-550.53734	-345462.18	3.05
M0004	-550.53723	-345462.11	3.12
M0003	-550.53680	-345461.84	3.40
M0009	-550.53564	-345461.11	4.12
M0012	-550.53561	-345461.09	4.14
M0011	-550.53552	-345461.04	4.20
M0014	-550.53486	-345460.62	4.61
M0013	-550.53478	-345460.57	4.66
M0017	-550.53421	-345460.22	5.02
M0016	-550.53344	-345459.74	5.50
M0023	-550.53341	-345459.72	5.52
M0020	-550.53331	-345459.65	5.58
M0019	-550.53329	-345459.64	5.60
M0022	-550.53327	-345459.63	5.61
M0021	-550.53318	-345459.57	5.66
M0015	-550.53283	-345459.35	5.89
M0025	-550.53279	-345459.33	5.91
M0024	-550.53239	-345459.07	6.17
M0018	-550.53163	-345458.60	6.64
M0006	-550.53157	-345458.56	6.68
M0005	-550.53155	-345458.55	6.69
**Cyclol 11c**	**Calculated energy (au)**	**Calculated energy (kcal/mol)**	**Relative energy (kcal/mol)**
M0001	-550.52939	-345457.19	0.00
M0003	-550.52580	-345454.94	2.26
M0002	-550.52177	-345452.41	4.79
M0004	-550.52026	-345451.46	5.73
**Lactone 13c**	**Calculated energy (au)**	**Calculated energy (kcal/mol)**	**Relative energy (kcal/mol)**
M0001	-550.53603	-345461.36	0.00
M0002	-550.53559	-345461.08	0.27
M0003	-550.53460	-345460.46	0.90
M0005	-550.52858	-345456.69	4.67
M0004	-550.52516	-345454.54	6.82
M0006	-550.52371	-345453.63	7.73
M0007	-550.52186	-345452.47	8.89

Table S13. Energies calculated at HF/6-31G* in vacuum for the conformers found for **9c/11c/13c**.

Isomers 9c/11c/13c (DFT/B3LYP/6-31G*)

Imide 9c	Calculated energy (au)	Calculated energy (kcal/mol)	Relative energy (kcal/mol)
M0002	-553.80768	-347514.32	0.00
M0001	-553.80759	-347514.26	0.06
M0004	-553.80495	-347512.60	1.72
M0003	-553.80440	-347512.26	2.06
M0008	-553.80261	-347511.14	3.18
M0010	-553.80140	-347510.38	3.94
M0012	-553.80054	-347509.84	4.48
M0009	-553.80045	-347509.78	4.54
M0007	-553.80024	-347509.65	4.67
M0011	-553.80004	-347509.53	4.79
M0015	-553.79986	-347509.41	4.91
M0021	-553.79947	-347509.17	5.15
M0006	-553.79941	-347509.13	5.19
M0020	-553.79931	-347509.07	5.25
M0005	-553.79929	-347509.05	5.27
M0014	-553.79889	-347508.80	5.52
M0019	-553.79887	-347508.79	5.53
M0018	-553.79868	-347508.67	5.65
M0017	-553.79854	-347508.58	5.74
M0023	-553.79825	-347508.40	5.92
M0013	-553.79762	-347508.00	6.32
M0022	-553.79761	-347508.00	6.32
M0016	-553.79669	-347507.42	6.90
M0024	-553.79009	-347503.28	11.04
M0026	-553.78833	-347502.18	12.14
M0027	-553.78797	-347501.95	12.37
M0025	-553.78660	-347501.09	13.23
M0029	-553.78623	-347500.86	13.46
M0031	-553.78509	-347500.14	14.18
M0032	-553.78418	-347499.57	14.75
M0030	-553.78190	-347498.14	16.18
M0028	-553.78105	-347497.61	16.71
M0033	-553.77975	-347496.79	17.53
**Cyclol 11c**	**Calculated energy (au)**	**Calculated energy (kcal/mol)**	**Relative energy (kcal/mol)**
M0002	-553.79414	-347505.83	0.00
M0003	-553.79186	-347504.39	1.44
M0001	-553.79104	-347503.88	1.95
M0006	-553.78898	-347502.59	3.24
M0005	-553.78703	-347501.36	4.47
M0004	-553.78560	-347500.46	5.36
M0008	-553.78554	-347500.43	5.40

M0007	-553.78429	-347499.64	6.18

Lactone 13c	Calculated energy (au)	Calculated energy (kcal/mol)	Relative energy (kcal/mol)
M0002	-553.80825	-347514.68	0.00
M0003	-553.80717	-347514.00	0.68
M0001	-553.80529	-347512.82	1.85
M0004	-553.80149	-347510.43	4.24
M0005	-553.79897	-347508.85	5.83
M0006	-553.79687	-347507.53	7.14
M0007	-553.78935	-347502.82	11.86
M0008	-553.78729	-347501.52	13.15

Table S14. Energies calculated at DFT/B3LYP/6-31G* in vacuum for the conformers found for **9c/11c/13c**.

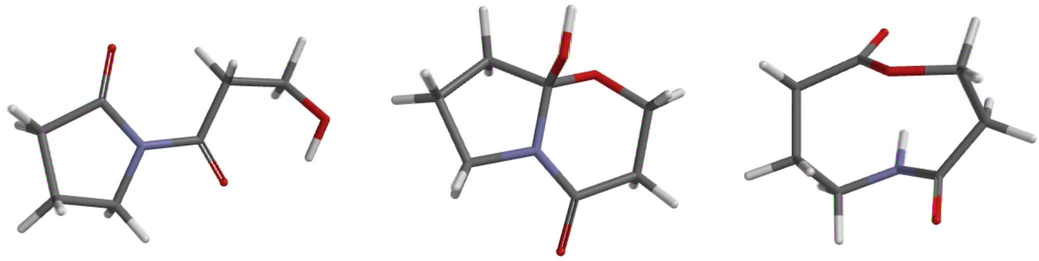

Imide **9c** Cyclol **11c** Lactone **13c**

Isomer	ΔG° (kcal/mol)		
	DFT/B3LYP/6-31G*		HF/6-31G*
	Vacuum	Non-polar solvents	Vacuum
Imide **9c**	0.0	0.0	0.0
Cyclol **11c**	11.7	11.1	12.1
Lactone **13c**	4.1	3.7	6.1

Table S15. Relative free energies (ΔG°) of the lowest energy geometries of **9c/11c/13c** at two levels of theory (DFT/B3LYP/6-31G* in a vacuum and in non-polar solvents) and HF/6-31G* in a vacuum) in kcal/mol.

Isomers 9d/11d/13d (HF/6-31G*)

Imide 9d	Calculated energy (au)	Calculated energy (kcal/mol)	Relative energy (kcal/mol)
M0002	-589.56841	-369954.18	0.00
M0001	-589.56797	-369953.90	0.28
M0009	-589.56489	-369951.97	2.21
M0008	-589.56393	-369951.37	2.81
M0006	-589.56387	-369951.33	2.85
M0011	-589.56373	-369951.24	2.94
M0005	-589.56345	-369951.07	3.11

M0010	-589.56271	-369950.60	3.58
M0012	-589.56218	-369950.27	3.91
M0004	-589.56215	-369950.25	3.93
M0014	-589.56173	-369949.98	4.20
M0020	-589.56167	-369949.95	4.23
M0017	-589.56122	-369949.67	4.51
M0015	-589.56091	-369949.47	4.71
M0013	-589.56039	-369949.14	5.04
M0016	-589.56002	-369948.91	5.27
M0018	-589.55952	-369948.60	5.58
M0024	-589.55936	-369948.50	5.68
M0022	-589.55919	-369948.39	5.79
M0003	-589.55905	-369948.31	5.87
M0027	-589.55903	-369948.29	5.89
M0021	-589.55883	-369948.16	6.02
M0025	-589.55854	-369947.99	6.19
M0023	-589.55810	-369947.71	6.47
M0007	-589.55698	-369947.00	7.18
M0019	-589.55693	-369946.97	7.21
M0026	-589.55560	-369946.14	8.04
**Cyclol 11d**	**Calculated energy (au)**	**Calculated energy (kcal/mol)**	**Relative energy (kcal/mol)**
M0001	-589.56935	-369954.77	0.00
M0002	-589.56428	-369951.59	3.18
M0003	-589.56333	-369950.99	3.78
M0005	-589.56119	-369949.64	5.13
M0006	-589.55934	-369948.48	6.29
M0004	-589.55742	-369947.28	7.49
M0008	-589.55213	-369943.96	10.81
M0007	-589.55129	-369943.43	11.34
**Lactone 13d**	**Calculated energy (au)**	**Calculated energy (kcal/mol)**	**Relative energy (kcal/mol)**
M0001	-589.57812	-369960.27	0.00
M0003	-589.57333	-369957.27	3.00
M0004	-589.57270	-369956.87	3.40
M0002	-589.57248	-369956.73	3.54
M0005	-589.57228	-369956.60	3.67
M0006	-589.57128	-369955.98	4.29
M0007	-589.57058	-369955.54	4.73
M0010	-589.56925	-369954.70	5.57
M0008	-589.56755	-369953.64	6.63
M0009	-589.56553	-369952.37	7.90
M0012	-589.56297	-369950.76	9.51
M0011	-589.56259	-369950.52	9.75
M0014	-589.56238	-369950.39	9.88
M0013	-589.56011	-369948.97	11.30

M0015	-589.55864	-369948.05	12.22

Table S16. Energies calculated at HF/6-31G* in vacuum for the conformers found for **9d/11d/13d**.

Isomers 9d/11d/13d (DFT/B3LYP/6-31G*)

Imide 9d	Calculated energy (au)	Calculated energy (kcal/mol)	Relative energy (kcal/mol)
M0001	-593.11533	-372179.87	0.00
M0002	-593.11513	-372179.74	0.12
M0010	-593.11257	-372178.14	1.73
M0009	-593.11146	-372177.44	2.43
M0011	-593.11068	-372176.95	2.92
M0004	-593.11023	-372176.67	3.20
M0008	-593.11019	-372176.64	3.23
M0006	-593.10928	-372176.07	3.80
M0005	-593.10881	-372175.78	4.09
M0017	-593.10855	-372175.62	4.25
M0012	-593.10811	-372175.34	4.53
M0003	-593.10790	-372175.21	4.66
M0023	-593.10742	-372174.91	4.96
M0015	-593.10708	-372174.69	5.17
M0018	-593.10705	-372174.67	5.20
M0014	-593.10659	-372174.39	5.48
M0007	-593.10625	-372174.17	5.70
M0013	-593.10594	-372173.97	5.89
M0021	-593.10573	-372173.84	6.03
M0022	-593.10569	-372173.82	6.05
M0016	-593.10493	-372173.34	6.53
M0020	-593.10492	-372173.34	6.53
M0019	-593.10317	-372172.24	7.63
M0024	-593.09933	-372169.83	10.04
M0026	-593.09892	-372169.57	10.30
M0025	-593.09420	-372166.61	13.26
M0027	-593.09290	-372165.79	14.07
M0028	-593.09193	-372165.18	14.68
M0031	-593.09043	-372164.25	15.62
M0029	-593.08577	-372161.32	18.55
M0032	-593.08470	-372160.65	19.22
M0030	-593.08332	-372159.79	20.08
**Cyclol 11d**	**Calculated energy (au)**	**Calculated energy (kcal/mol)**	**Relative energy (kcal/mol)**
M0001	-593.11369	-372178.84	0.00
M0002	-593.10992	-372176.47	2.37
M0004	-593.10871	-372175.71	3.12

M0003	-593.10858	-372175.63	3.21
M0007	-593.10623	-372174.16	4.68
M0005	-593.10542	-372173.65	5.19
M0008	-593.10401	-372172.76	6.07
M0011	-593.10363	-372172.53	6.31
M0006	-593.10352	-372172.46	6.38
M0010	-593.09897	-372169.61	9.23
M0014	-593.09769	-372168.80	10.04
M0009	-593.09733	-372168.58	10.26
M0012	-593.09665	-372168.15	10.69
M0013	-593.09481	-372166.99	11.84
**Lactone 13d**	**Calculated energy (au)**	**Calculated energy (kcal/mol)**	**Relative energy (kcal/mol)**
M0001	-593.12568	-372186.36	0.00
M0003	-593.12179	-372183.93	2.44
M0002	-593.12148	-372183.73	2.63
M0004	-593.11935	-372182.39	3.97
M0005	-593.11749	-372181.23	5.13
M0006	-593.11229	-372177.96	8.40
M0008	-593.11095	-372177.12	9.24
M0007	-593.10948	-372176.20	10.16
M0009	-593.10942	-372176.16	10.20
M0011	-593.10435	-372172.98	13.38
M0013	-593.10382	-372172.65	13.71
M0010	-593.10347	-372172.43	13.94
M0014	-593.10324	-372172.28	14.08
M0012	-593.10231	-372171.70	14.67
M0016	-593.10053	-372170.58	15.78
M0015	-593.09839	-372169.24	17.12
M0017	-593.09832	-372169.19	17.17
M0018	-593.09528	-372167.29	19.07
M0019	-593.09226	-372165.39	20.97
M0021	-593.07753	-372156.15	30.21
M0020	-593.07406	-372153.97	32.39
M0022	-593.07256	-372153.03	33.33

Table S17. Energies calculated at DFT/B3LYP/6-31G* in vacuum for the conformers found for **9d/11d/13d**.

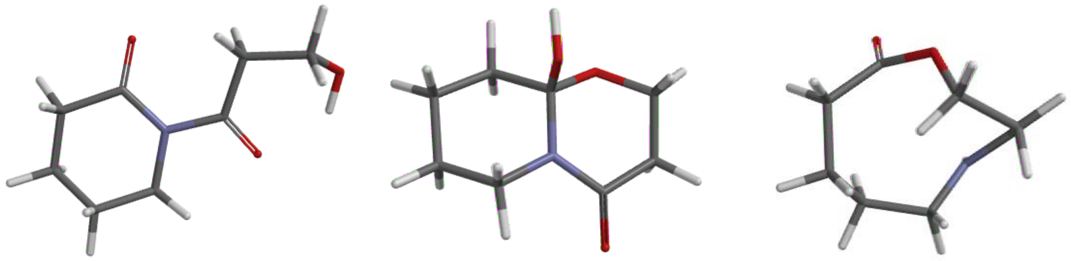

Imide **9d** Cyclol **11d** Lactone **13d**

**Isomer**	**ΔG° (kcal/mol)**		
	**DFT/B3LYP/6-31G***		**HF/6-31G***
	**Vacuum**	**Non-polar solvents**	**Vacuum**
Imide **9d**	2.4	3.6	1.8
Cyclol **11d**	8.1	8.9	6.6
Lactone **13d**	0.0	0.0	0.0

Table S18. Relative free energies (ΔG°) of the lowest energy geometries of **9d/11d/13d** at two levels of theory (DFT/B3LYP/6-31G* in a vacuum and in non-polar solvents) and HF/6-31G* in a vacuum) in kcal/mol.

References

[1] T. C. Stephens, M. Lodi, A. Steer, Y. Lin, M. Gill, W. P. Unsworth, *Chem. Eur. J.* 2017, **23**, 13314.

[2] H. F. Motiwala, M. Charaschanya, V. W. Day, J. Aube, *J. Org. Chem*, 2016, **81**, 1593.

[3] Y. Matsumura, T. Suzuki, A. Sakakura, K. Ishihara, *Angew. Chem. Int. Ed*., 2014, **53**, 6131.

[4] Y.-X. Jia, J. M. Hillgren, E. L. Watson, S. P. Marsden, E. P. Kundig *Chem.Commun*., 2008, 4040.

[5] L. G. Borboa, O. Nunez, *J. Phys. Org. Chem.*, 2006, **19**, 737

[6] Spartan Student Wavefunction, Inc. Irvine, CA.

[7] Spartan’14 Wavefunction, Inc. Irvine, CA.

[8] (a) A. D. Becke, *J. Chem. Phys*. 1992, **97**, 9173; (b) A. D. Becke, *J. Chem. Phys*., 1993, **98**, 5648; (c) C. Lee, W. Yang, R. G. Parr, *Phys. Rev. B*., 1988, **37**, 785.

[9] T. A. Halgren, *J Comput. Chem.*, 1996, **17**, 490.

[10] D. R. Hartree, *Math. Proc. Camb. Philos. Soc*., 1928, **24**, 89–132; D. R. Hartree, *Math. Proc. Camb. Philos. Soc*., 1928, **24**, 426–437.
